# Human interactions with delivery drones in public spaces: design recommendations from recipient and bystander perspectives

**DOI:** 10.3389/frobt.2025.1580289

**Published:** 2025-05-30

**Authors:** Shiva Nischal Lingam, Rutger Verstegen, Sebastiaan M. Petermeijer, Marieke Martens

**Affiliations:** ^1^ Aerospace Operations Safety and Human Performance, Royal Netherlands Aerospace Center, Amsterdam, Netherlands; ^2^ Industrial Design, Eindhoven University of Technology, Eindhoven, Netherlands; ^3^ Aerospace Operations Training and Simulation, Royal Netherlands Aerospace Center, Amsterdam, Netherlands; ^4^ Integrated Vehicle Safety, TNO, Helmond, Netherlands

**Keywords:** human-robot interaction, human-drone interaction, robot design, human-machine interface, delivery application, interview, focus group, public space

## Abstract

Drones will likely deliver packages in public spaces, where humans interact as recipients of the package and as bystanders passing by. Understanding the human needs and uncertainties that may arise during these interactions is crucial to ensure safety. This user-centered design study employed twelve interviews and four focus groups to identify key requirements for recipients and bystanders interacting with delivery drones in public spaces. Findings demonstrate different information needs and preferred interface modalities between recipients and bystanders across various interaction stages, from ordering a package to the drone’s retraction after delivery. This paper highlights essential design features and offers concrete design recommendations based on the interaction requirements. These recommendations can inform the standardization and customization of design features for each interaction stage, enhancing safety and facilitating natural human-drone interaction. Future research should build on these recommendations and validate the design concepts through experimental user studies involving human interactions with delivery drones in public spaces.

## 1 Introduction

Drones are increasingly becoming part of daily life, with the expanding global drone market creating new opportunities for consumer interactions and integration into public spaces. Consumers now receive package deliveries from drones, fostering interactions between drones and humans (Shankland, 2023). Delivery is highlighted as a major application in the Human-Drone Interaction (HDI; a sister domain to Human-Robot Interaction) literature, as reviewed by Herdel et al. (2022). An expert interview study on HDI by Lingam et al. (2024) also identifies delivery as a key application in public spaces for the coming decade. These findings suggest that delivery will be a primary use case for drones in public spaces, involving interaction with the individuals. However, designing robots (e.g., drones) to safely operate and adapt to the uncertainties of public spaces is challenging due to complexities, arising from diverse situational factors as well as the involvement of various stakeholders, including recipient, bystanders, and vulnerable populations (Yu et al., 2024).

Consideration of public needs and acceptance is crucial for the successful integration of drone delivery technology into society. Zenz and Powles (2024) examine public resistance to drone delivery services, with a particular focus on Google Wing’s operations in Canberra, Australia. The authors highlight how inadequate community engagement can lead to resistance, disrupting corporate plans and emphasizing the importance of aligning technological development with public interests. In public spaces, humans primarily interact with delivery drones in two roles: as recipients who actively interact by receiving packages or as bystanders who may be nearby but do not participate in the interaction (Lingam et al., 2024). Expectations and informational needs differ between these roles; for example, while recipients anticipate their interaction with the drone, bystanders might be unaware of the drone’s purpose and require contextual information. Prior research (Lingam et al., 2025; Obaid et al., 2015; Tan et al., 2018) has primarily focused on the perspective of recipients in public spaces, however, bystander perspectives have been less frequently studied. Previous Human-Robot Interaction (HRI) research (Nielsen et al., 2023; Pelikan et al., 2024; Yu et al., 2024) underscore the need to consider bystander roles, as delivery robots are likely to interact with bystanders more frequently than recipients (Rosenthal-von der Pütten et al., 2020) and can contribute to interaction breakdowns (Nielsen et al., 2023). The lack of consideration for both (i.e., recipient and bystander) perspectives in the HDI design space leads to feelings of uncertainty (henceforth referred to as uncertainty), which can affect their trust in automated systems, such as drones (Lee and See, 2004).

A possible approach to reducing uncertainty involves carefully designing the appearance of drones and implementing Human-Machine Interfaces (HMIs). For instance, drones could adopt visual cues similar to delivery vehicles on the road, making their purpose more evident to the public (Lingam et al., 2024). Additionally, HMIs such as speakers and ground projections can communicate specific intentions of drones to the public (Obaid et al., 2015). However, research exploring user requirements for the delivery drones in public spaces remains limited. As drones increasingly enter public spaces for deliveries, variations in current delivery drone designs (c.f., Matternet, 2024; Shankland, 2023; Vuleta, 2021) prompt questions about the impact of these designs on human perception. A lack of discussion on standardization and customisation of design elements in appearance and interfaces can lead to varied interpretations, potentially causing confusion and increasing uncertainty among the public.

The literature lacks clarity on which specific design elements need standardization to effectively communicate delivery intentions, which aspects should be customized based on the type of human role (recipient and bystander), and how these elements interrelate in an interaction space. Understanding the balance between standardization and customisation in drone design is crucial for developing user-centered design principles that address the needs of public users. Our study attempts to fill these gaps and contribute to the future development of natural interaction between public users (i.e., recipients and bystanders) and delivery drones.

This study aims to explore user requirements and offer recommendations for designing drones and their interaction spaces to address uncertainty in a public space (e.g., park) delivery context, focusing on the roles of recipients and bystanders. By investigating user needs and preferences regarding drone interactions, the study seeks to identify and discuss essential design features for each role. These insights contribute to developing design guidelines for future researchers and drone designers and improving the natural HDI experience.

The contributions of this study for the HDI and HRI community are:

• Exploration of the roles of humans, as recipients and bystanders, in interactions with delivery robots in public spaces.
• Identification of uncertainty factors and user requirements for HDI with delivery robots in public spaces.
• Highlighting essential design features for standardization and customisation, along with user reflections on current delivery drone designs.
• Provision of design recommendations for each interaction stage to reduce uncertainty and improve safety.

## 2 Background

Our research is informed by four key areas in the literature. First, we present the background on the human roles in public spaces within HDI and HRI. Next, we explore the significance of addressing human perceived uncertainty in HDI. Then, we consider the challenges and strategies for managing uncertainty through design features in drones. Finally, we discuss the background and relevance of the user-centered approach in the fields of HDI and HRI, which forms the foundation of our research methods.

### 2.1 Human roles in public space interactions

Individuals from the public interact with drones in two primary ways: actively, as recipients engaging with the drone service, or passively, as bystanders situated in the vicinity of the drone (Lingam et al., 2024). Previous HDI studies have primarily focused on the role of the recipient in public spaces. For instance, Obaid et al. (2015) evaluated the use of HMIs for drones to assist road users in discarding garbage, and Tan et al. (2018) examined the delivery of a wedding ring by a drone in a public event. However, the emerging passive role of the bystander has been rarely investigated within HDI.

The exploration of bystander interactions has been gaining attention in HRI, particularly for service robots in public spaces (e.g., Nielsen et al., 2023; Pelikan et al., 2024; Yu et al., 2024). Bystanders, though not the primary users, are likely to encounter delivery robots more often than recipients (Rosenthal-von der Pütten et al., 2020). A design probe study (Yu et al., 2024) and a video-based study (Pelikan et al., 2024) found that ground robots disrupt bystander activities when not designed to adapt to context, particularly when the robots’ objectives differ from bystander interests. The authors (Pelikan et al., 2024; Yu et al., 2024) proposed contextually adaptive robot designs that account for diverse bystander needs to facilitate natural HRI in public environments. In another video study (Nielsen et al., 2023), findings indicated that over 30% of disruptions in interactions occur between a public service robot and bystanders in an airport, in addition to challenges related to environmental disruptions and control features. Interactions with bystanders frequently broke down due to interruptions caused by children and adults, who are curious. Collectively, these studies (Nielsen et al., 2023; Pelikan et al., 2024; Yu et al., 2024) highlight the importance of considering bystander interests to ensure safe and natural HRI in public spaces.

Recipients and bystanders may share the same space during drone deliveries, but their roles differ. Recipients expect to interact with the drone to receive a package, while bystanders may be unaware of the drone’s purpose and have limited involvement (Lingam et al., 2024). These differences necessitate distinct requirements and interaction protocols for safer and more natural HDI. Addressing these differences presents challenges in drone design and interaction that are rarely covered in existing HDI literature.

### 2.2 Feelings of uncertainty in HDI

Due to the complexity of public spaces and the novelty of drone technology, uncertainty may arise during HDI. In accordance with the explanation provided by the research experts in the field of HDI across academia and industry (Lingam et al., 2024), we define uncertainty as “a state of doubt experienced by humans when interactions with drones deviate from the expected, leading to a loss of understanding of the drone’s intentions or its next actions.” Handling uncertainty is a critical challenge in integrating drones into public spaces (Lingam et al., 2024). Uncertainty may rise during the human interactions with robots, such as drones, that negatively affects the decision making of the human (Lindley, 2013) and defines the boundaries of trust in automated systems (Lee and See, 2004). Uncertainties about drone identification and purpose can cause confusion and discomfort, making it essential to address these issues to prevent miscommunication, enhance trust and safety, and ensure natural HDI. Limited familiarity with drones contributes to the uncertainty. Vuleta (2021) found that only 15% of U.S. residents have experience operating drones. Due to current safety regulations (Federal Aviation Administration, 2024), the proportion of individuals with experience interacting with delivery drones to receive packages is likely even lower. The lack of knowledge and information can lead to uncertainty and higher perception of risks towards robots, such as drones (Clothier et al., 2015; Meissner et al., 2020).

A possible direction to address uncertainty regarding interactions with delivery drones involves comprehending and designing as per the user’s expectations and needs regarding both the drone and the interaction. Fink et al. (2023) discussed the importance of understanding user requirements by involving humans in the development process of medical drone services, concerns can be addressed, and the security, usability, and acceptance of the technology can be improved. Shapira and Cauchard (2022) highlighted the significance of crafting public service drones and the elements of interaction spaces, such as drone appearance, with user experience in mind to enhance public acceptance. While it is crucial to reduce uncertainty to promote a natural HDI experience (Jane et al., 2017), there is a gap in the literature regarding the investigation of user needs for handling uncertainty, particularly in the context of delivery drones within HDI.

### 2.3 Managing uncertainty through drone design

Design elements such as flying patterns, propeller noise, drone appearance, and HMIs play a crucial role in conveying drone intentions, as highlighted in recent expert interviews and user studies (Bevins and Duncan, 2021; Lingam et al., 2024; 2025; Obaid et al., 2015; Fink et al., 2023; Tan et al., 2018), and managing user uncertainty in HDI. For instance, experts in Lingam et al. (2024) suggested that propeller noise, similar to an ambulance siren, could signal a drone’s presence and purpose, thereby reducing uncertainty in HDI. Bevins and Duncan (2021) examined how flying patterns affect user perception of drone intentions in a video-based study, observing that an undulating flight pattern and a straight descent were both interpreted as signals to avoid approaching the drone. A virtual reality experiment by Lingam et al. (2025) investigated drone flight paths and delivery methods, finding that drones approaching recipients in curved paths and delivering packages through a cable while hovering above eye level were associated with lower uncertainty and higher trust compared to drones following a straight path and landing on ground to deliver packages. Flying patterns and propeller noise serve as implicit cues for drone intentions; however, humans may interpret the same cue in different ways. For example, Bevins and Duncan (2021) found that while some participants perceived a U-shaped flight pattern as a signal to avoid approaching the drone, others interpreted it as an invitation to look at the drone.

Another approach is to explore explicit forms of communication such as drone appearance and HMIs. Such cues improve clarity and interpretability of the drone intentions by conveying explicit information (Fink et al., 2023). In Lingam et al. (2024), experts underscored the importance of purpose-reflective drone design, drawing inspiration from vehicles like delivery trucks and ambulances. On similar lines, Tan et al. (2018) explored user preferences for delivery drone design, recommending propeller guards and explicit communication of intentions to enhance safety and comfort. In contrast, Wojciechowska et al. (2019) conducted an online study where participants rated 63 static images of drones using Likert scale statements, regardless of the application area, and recommended excluding propeller guards on delivery drones to improve trustworthiness, interaction likability, and friendliness. Post-experiment interviews from Lingam et al. (2025) showed that recipients desired design features reflecting the drone’s purpose, suggesting ambulance-like features for medical drones. Recipients also expressed the need for delivery intentions to be communicated via sound and lights, particularly during package drop-offs. Experts (Lingam et al., 2024) have suggested using ground projections to indicate landing spots and implementing audio signals to signal safety warnings to nearby recipients. Additionally, Obaid et al. (2015) found that combining audio and ground projections in HMIs was more effective than audio alone in persuading recipients to clean the garbage in the public space, demonstrating greater influence. While these studies highlight the potential for managing recipient uncertainty through design elements, they offer limited insights into how these design elements compare with the needs of bystanders.

Current delivery drone models vary in terms of appearance and the availability of HMIs to communicate with users. For instance, TU Delft’s ambulance drone (Momon, 2014) includes an onboard audio interface to guide recipients during emergencies by delivering Automated External Defibrillators. In contrast, drones from Matternet (2024), Wing (2024), and Zipline (Shankland, 2023) used for medical and commercial deliveries lack both such interfaces and ambulance-like designs. The drone models also vary significantly in appearance, including colors and forms, as well as in their delivery methods. This diversity raises questions regarding the impact of appearance and HMIs on user perceptions and expectations. The appearance of drones, including their design and forms, may influence how users perceive the technology and the services provided (Wojciechowska et al., 2019). A possible direction is to identify the design elements of drone models and HMIs that require standardization according to the use case (i.e., delivery) to manage the uncertainty experienced by public users (Lingam et al., 2025).

### 2.4 User-centered design approach

The user-centered design approach has been widely applied in the fields of HRI and HDI to investigate and incorporate human needs throughout the design process of robots, ensuring that human needs are appropriately considered (Alon et al., 2021; Amiche et al., 2024; Herdel et al., 2021; Karjalainen et al., 2017; Tan et al., 2018; Yeh et al., 2017). The design process often involves iterative and multi-stage activities, such as user needs exploration and design workshops, in order to provide recommendations for future developments of robots (Amiche et al., 2024).

Previous HDI studies have conducted interviews to investigate expert user requirements (Khan and Neustaeder, 2019; Ljungblad et al., 2021). For instance, Ljungblad et al. (2021) interviewed drone pilots to identify their needs for drone applications and proposed corresponding design guidelines. Khan and Neustaeder (2021) interviewed firefighters to understand their needs and provided design recommendations for drones assisting in firefighting operations. Other studies have conducted focus groups and design workshops to explore and derive design implications for drones interacting with recipients (Herdel et al., 2021; Karjalainen et al., 2017; Tan et al., 2018; Yeh et al., 2017). For example, Herdel et al. (2021) conducted focus groups with participants, asking them to sketch potential capabilities for police drones in public spaces, considering varying levels of context severity. Karjalainen et al. (2017) organized design workshops to understand and elaborate on appearance requirements for a companion drone. Tan et al. (2018) explored recipient preferences for delivery drone design using focus groups, while Yeh et al. (2017) conducted sketching sessions to capture how recipients envision a social drone and to elaborate on preferred design features. These HDI studies provided recommendations for drone design and their integration into human environments. Our research, which aims to explore human (i.e., recipient and bystander) requirements and to offer design recommendations for drones and their interaction spaces in delivery contexts, draws inspiration from the user-centered design approach.

## 3 Methods

**FIGURE 1:**
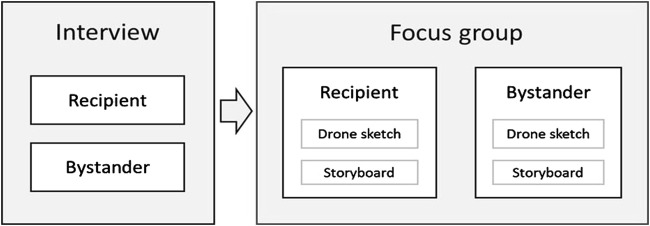
Study methods implemented to explore requirements for the recipient and bystander roles.

The study implemented a user-centered design process consisting of two stages: online interviews and focus groups with ideation sessions (see Figure 1). Online interviews were conducted to explore individual experiences, needs, and uncertainties in depth. Follow-up focus group sessions were utilized to stimulate group discussions to let participants raise issues that might not have been identified in the interviews (Lazar et al., 2017). The approach was inspired by iterative design processes in HDI literature, including interviews, surveys, and focus groups with ideation sessions (e.g., Herdel et al., 2021; Tan et al., 2018; Yeh et al., 2017). The study design was approved by the Eindhoven University Ethical Review Board.

### 3.1 Interviews

The interviews were conducted and recorded via Microsoft Teams by the first author. Before the interviews, participants were provided with a document detailing two (fictional) scenarios that illustrated the roles of recipient and bystander, to set context and expectations (Buskermolen and Terken, 2012). A semi-structured interview approach was used, with questions iteratively developed through research group discussions, insights from prior studies on HDI for delivery drones (Lingam et al., 2024; 2025), and results from the pilot study.

### 3.2 Focus groups

The first and second authors moderated the group discussions and ideation sessions, which were audio-recorded to capture humans’ perceptions and visions of interacting with delivery drones, as well as to understand the rationale behind their preferred features. Participants were initially encouraged to discuss their requirements for each role separately within their groups. They storyboarded interactions with delivery drones, a method commonly used in HCI literature (e.g., Kantola and Jokela, 2007; Truong et al., 2006), and sketched the drone (e.g., Herdel et al., 2021; Yeh et al., 2017) based on their discussions and preferences. During the pilot sessions and interviews, participants desired information on the phone. Consequently, interface cards shaped like smartphone cutouts were provided to represent mobile information requirements during storyboarding.

### 3.3 Procedure

Participants were asked to complete a questionnaire on their demographics (gender, age, ethnicity, educational background), attitudes towards technology interaction (Franke et al., 2019), experience with drones, and provide consent. They read the scenario document and were then interviewed (see Supplementary Material for the scenario description and questions) to understand the requirements for the two roles in the delivery context within a public park. Participants used the Miro board (https://miro.com/) to categorize requirements according to the MoSCoW prioritization method into four categories: 1) “Must Have,” 2) “Should Have,” 3) “Could Have,” and 4) “Won’t Have” (Clegg and Barker, 1994). The MoSCoW principle has been used previously to prioritize user requirements in the design of human-technology interactions, such as those involving autonomous vehicles (Hallewell et al., 2022; Rodak et al., 2020) and smartphones (Vos et al., 2016).

**FIGURE 2:**
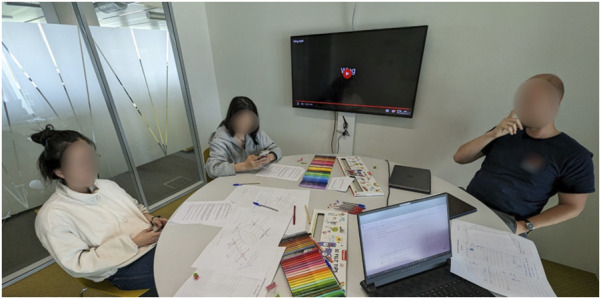
Participants in a focus group. They were given A3 papers, colored markers, and pens to create sketches.

Participants were invited to focus groups where they storyboarded interaction scenarios and sketched the drone in separate sessions for the roles of recipient and bystander. They were provided with A3 papers, colored markers, and pens (see Figure 2) for these tasks and could choose to incorporate attributes identified in the interviews or not. Additionally, participants were provided with a drone silhouette (i.e., a hybrid VTOL model of the Zipline drone) on an A3 paper to initiate their sketches, inspired by previous studies (Herdel et al., 2021; Yeh et al., 2017). They were informed that this silhouette was for inspiration and not mandatory to adopt in their designs. They could sketch HMIs directly on the drone or on interface cards, and were asked to explain their design choices while remaining open to alternative solutions.

After completing the storyboard and sketching phases, participants watched videos of existing drone models (see Supplementary Material for videos), including Amazon Prime Air, Mana, Wing, and Zipline, to prompt reflections on their preferences and perceptions. Adjustments to their sketches were permitted based on the observed features. Although the sample of drone models in the videos was not exhaustive, it served as inspiration, given participants’ limited real-world experience with delivery drones. The interviews and focus groups lasted approximately 1 hour and about 2 hours, respectively. Participants were thanked and compensated with a €35 voucher at the end of the study.

### 3.4 Participants

The study prioritized the depth and quality of qualitative data over larger sample size (Lingam et al., 2024; Ljungblad et al., 2021). Previous user-centered design studies in HDI have reached acceptable results and saturation with fewer than 10 participants (e.g., Alon et al., 2021; Herdel et al., 2022; Tan et al., 2018). Twelve participants were recruited for this study to ensure balanced focus groups and rich data collection, with sessions lasting approximately 3 hours for each participant and involving multiple methods for both recipient and bystander roles. All the participants had seen drones in the media or from a distance in reality, but none had experience piloting, owning, or seeing a delivery drone. Participants were selected based on their limited experience with drones and their background in design. Their limited prior interaction with drones could provide insights from a novice public user perspective, as most residents in the Netherlands (where the study was conducted) currently lack experience with delivery drones. This is due to the state of technology and existing regulations, which prohibit delivery drones (>500 g) from flying near humans, requiring a minimum horizontal distance of 50 m (Netherlands Enterprise Agency, 2024). During the pilot study, participants without a design background faced difficulty to provide design solutions during the focus group discussions. To facilitate a clearer articulation of complex ideas through sketches and storyboards, participants with a background in visual communication design were recruited.

Participants (five male, seven female) were aged 25–34 years (M = 29.2, SD = 3.2) and were from diverse backgrounds: five Chinese, two Dutch, two Indian, and one each from Brazil, Greece, and Indonesia. Overall, they indicated a positive attitude towards technology interaction (M = 3.8, SD = 0.6). Twelve interviews and four focus groups, each consisting of three participants, were conducted in June and July 2024.

### 3.5 Analysis methods

A thematic analysis was conducted to qualitatively identify and report patterns in participant responses (Braun and Clarke, 2006), consistent with prior HDI research (Lingam et al., 2024; 2025; Ljungblad et al., 2021). The analysis was performed on the transcriptions, sketches and storyboards. Interview and focus group recordings were automatically transcribed and then reviewed and corrected for accuracy by the first and second authors (referred to as analysts). Personal information, such as names, was removed. First, the analysts familiarized themselves with the transcriptions to extract insights from the interview data. Second, they familiarized themselves with the focus group transcriptions, sketches and storyboards to identify recurring visual motifs, narrative structures, and thematic elements. Codes were created to categorize elements, which were then organized into sub-themes and themes. For example, interview codes related to the role of external agents, the presence of multiple recipients and drones, and environmental factors contributing to user uncertainty were organized under the sub-theme, public space dynamics. The codes, sub-themes and themes were compared across the two human roles to identify similarities and differences. The analysis was conducted in a data-driven and emergent manner. The results from the analysis indicated a degree of saturation within the utilized sample, as observed by the consistency of sub-themes and themes across participants.

Information requirements and attributes mentioned by participants were categorized using the MoSCoW method and quantified.

## 4 Results

### 4.1 Interview results

The thematic analysis identified three main themes: factors contributing to user uncertainty about HDI, user requirements to feel certain during HDI, and drone design solutions to address uncertainty in HDI, along with a total of 12 sub-themes. These themes (see Table 1) are presented in the following sections, accompanied by selected participant quotes. Participant quotes from the interviews are labeled with “P,” followed by the corresponding interview number.

**TABLE 1:** Themes and corresponding sub-themes identified through thematic analysis.

Factors contributing to user uncertainty about HDI	User requirements to feel certain during HDI	Drone design solutions to address uncertainty in HDI
Differences in human roles	Tracking information for the recipient	Drone appearance
Public space dynamics	Recognition of the drone and recipient	Human-machine interfaces to communicate drone intentions
Familiarity with delivery drone technology and processes	Landing/take-off intention of the drone	Use case dependency
Criticality of the situation	Limited user intervention in drone control	
Privacy concerns		

#### 4.1.1 Factors contributing to user uncertainty about HDI

Participants expressed uncertainty when interacting with delivery drones in public spaces, primarily due to the unpredictability associated with the novelty of the technology and its autonomous functioning. They identified handling uncertainty as a significant challenge, citing factors such as *differences in human roles*, *public space dynamics*, *familiarity with delivery drone technology and processes*, *criticality of the situation*, and *privacy concerns*. These elements (see below sub-sections) collectively shaped their feelings of uncertainty toward delivery drones in public spaces like parks.

##### 4.1.1.1 Differences in human roles

The levels and types of uncertainties differed between the two human roles: recipient and bystander. As the recipient has “(...) much more of an expected engagement” (P6) with the drone than a bystander, the possible interactions and the uncertainties are different and higher for a bystander:

“(...) you can probably have like lists of possible use cases of possible archetypes [for recipients]. But for bystanders, you could have like, so many unpredictable variations, reactions and dynamics in the park” (P1).

“I will definitely feel more uncertain as [a bystander] compared to being a recipient” (P9).

Majority of the participants, as recipients, expressed feeling less uncertain during the interaction. Uncertainties regarding safety involve interactions, flying behavior, drone identification, drone and package size, and recipient positioning for package delivery. On the other hand, as a bystander, most of the participants reported feeling uncertain when a delivery drone approaches the vicinity. The bystanders expressed mixed emotions and uncertainty towards the propeller noise, the purpose of the drone, the context and the delivery location.

##### 4.1.1.2 Public space dynamics

The dynamics of a public space, such as the existence of environmental factors, the role of external agents, and the presence of multiple recipients and drones, could influence uncertainty as, “(...) there is a lot going to be happening in that [delivery] situation” (P6). These uncertainties, related to public space dynamics, might prompt recipients to alter the delivery location shortly before the drone arrives.

Environmental factors in and around the park, such as weather conditions, geographical features (e.g., park layout, rivers), cars, and trees, could contribute to feelings of uncertainty during the delivery interaction, especially when compared to drone deliveries to homes. If multiple groups are ordering deliveries in a public space, recipients might be uncertain about identifying the correct drones and could receive misplaced packages. Additionally, both recipients and bystanders could be annoyed by the presence and noise of several drones. Uncertainties caused by external agents, such as recipients and bystanders engaging in activities with friends, family (including elders and children), pets, and birds, were recognised as hindrances to the delivery process and raise safety concerns: “(...) have people running up to the drone (...) because that’s going to create chaos” (P8).

##### 4.1.1.3 Familiarity with delivery drone technology and processes

Uncertainty was expressed about “(...) how the drone will behave” (P11) due to participants’ lack of familiarity with drone technology and delivery processes. Instead, they drew comparisons with existing services, such as DHL and UberEats for package deliveries (e.g., “imagine the drone is the food delivery guy” (P3)), and ambulances for medical deliveries. Participants expressed uncertainty about the delivery methods, for instance, “I don't know if the drone would land or how are the package[s] being delivered” (P12).

##### 4.1.1.4 Criticality of the situation

Uncertainties and reactions varied depending on the criticality of the situation in which the drone delivered packages. As recipients, participants noted that critical situations increased their uncertainty regarding waiting time, delivery location, and process, stating that “when we change the scenario, there is also a change in terms of priority” (P1). As bystanders, some participants expressed that they would not intervene, others indicated they would step aside for the drone, and remaining mentioned they would follow the drone to assist the recipient in an emergency.

A majority of participants noted a potential shift in the role of bystander to recipient in critical situations. It was anticipated that a bystander might need to receive a medical package from the drone and assist the unwell patient. This role change was expected to lead to uncertainties regarding the delivery process, interactions with the drone to collect the package, and the use of medical contents to aid the patient.

##### 4.1.1.5 Privacy concerns

Both recipients and bystanders expressed privacy concerns. Recipients were primarily worried about their personal information being disclosed publicly and preferred discreet, non-intrusive communication methods (e.g., using codes instead of announcing names). Bystanders, on the other hand, were concerned about drones equipped with cameras and the possibility of being recorded. They emphasized the need for “reassurance of privacy” (P8) through data protection and ethical practices.

#### 4.1.2 User requirements to feel certain during HDI

Participants suggested that managing uncertainty could involve understanding user expectations and requirements, and proposing design solutions accordingly. Participants noted that designs aligned with user requirements could address uncertainty, promote safety, and help manage anxiety during interactions:

“As a person I have a lot of anxiety. I would like to know some stuff beforehand” (P8).

“I think drones are in general still quite dangerous that you want to make it as safe as possible. So I probably put some requirements” (P10).

User requirements included information on *tracking information for the recipient*, *recognition of* the *drone and recipient*, *landing/take-off intentions of the drone*, and *limited user intervention in drone control*. *Recognition of the drone and recipient*, *landing/take-off intentions of the drone*, and *limited user intervention in drone control* were mentioned for both roles, while *tracking information for the recipient* was specifically required for the role of the recipient. As shown in Table 2, for recipients, *recognition of the drone and recipient* was identified with the highest number of “inclusion requirements” (22), followed by *tracking information for the recipient* (17). *Limited user intervention in drone control* was regarded as the least significant “inclusion requirement” (1) and had the “exclusion requirements” (1). For the role of bystander, Table 3 shows that *recognition of the drone and recipient* was given the highest number of “inclusion requirements” (15), followed by l*anding/take-off intentions of the drone* (6). *Tracking information for the recipient* was deemed as not a requirement, while *limited user intervention in drone control* received “exclusion requirements” (2).

**TABLE 2:** Number of recipient requirements from the MoSCOW prioritization notes during the interviews.

Sub-theme	User requirements			
	Must have	Should have	Could have	Won’t have
Tracking information for the recipient	8	7	2	0
Recognition of the drone and recipient	12	6	4	0
Landing/take-off intentions of the drone	10	2	0	0
Limited user intervention in drone control	0	0	1	1

The Must Have, Should Have and Could Have requirements (green cells) are referred to as “inclusion requirements” and the Won’t Have requirements (red cells) as “exclusion requirements.”

**TABLE 3:** Number of bystander requirements from the MoSCOW prioritization notes during the interviews.

Sub-theme	User requirements			
	Must have	Should have	Could have	Won’t have
Tracking information for the recipient	0	0	0	0
Recognition of the drone and recipient	11	2	2	0
Landing/take-off intentions of the drone	5	1	0	0
Limited user intervention in drone control	0	0	0	2

The Must Have, Should Have and Could Have requirements (green cells) are referred to as “inclusion requirements” and the Won’t Have requirements (red cells) as “exclusion requirements.”

##### 4.1.2.1 Tracking information for the recipient

Participants emphasized the need for recipients to have access to timely delivery tracking information, including delivery process and live location, as, “those [pieces of information] are for the expectation and [they] will feel more transparent (...)” (P3) and allowing them to plan their time and actions in advance.

Information on the delivery process was found to provide recipients with “context on the delivery status” (P9) and to help them understand “the drone is arriving or what is happening” (P8). Participants noted the potential for inaccuracies in the estimated delivery time, drawing examples from current food delivery services. They added that receiving live updates on the drone’s location while en route would help them not only track the package and compensate for delivery time inaccuracies but also distinguish their drone from others and determine its arrival direction. This would reduce uncertainty and provide a greater sense of control.

##### 4.1.2.2 Recognition of the drone and recipient

A majority of participants highlighted the need for information to recognise both the drone and the recipient. Participants, as recipients, required details to identify and verify their drone, as well as guidance on interaction. Bystanders, on the other hand, preferred information about the recipient, the drone’s presence, and its purpose in their vicinity.

Participants highlighted the need for recipients to identify and verify their drone, in case of a scenario with multiple delivery drones and recipients in a public space were to happen. Identifying the drone allows recipients to: “(..) immediately know like, oh, this [drone] is mine, or this [drone] is not mine and you can act quickly” (P4). The verification process was found to enhance safety and reduce uncertainty, ensuring that the package was not lost and that the delivery process was complete:

“What if I didn't finish the receiving process? If it starts to fly, then I will lose my package” (P3).

“I put if I have a key to open the drone to receive my package. (...) I think it is very important to have it to avoid that somebody else will not take your package” (P11).

Especially during the early adoption phases, a tutorial on the delivery interaction, the drone and the user tasks was deemed necessary before the drone arrival to facilitate safe interaction, set expectations, save time, and reduce uncertainty.

Most participants, as bystanders, highlighted the need to understand the drone’s purpose and presence in their vicinity “so that they are informed about the [basic] intentions” (P5) and to address uncertainties, safety, and privacy concerns. They were less concerned with specific flying behavior and focusing instead on the drone’s overall activity, such as “whether it is delivering a package or emergency or involving in some of the other activities” (P2). Awareness of the drone’s presence was considered to reduce uncertainty and mitigate surprise, especially for those preoccupied in the park who might not otherwise visually notice the drone: “It’s for the person that is within the space to know what is around them. I think that is a basic—It’s a safety thing. It’s a social thing” (P5).

##### 4.1.2.3 Landing/take-off intention of the drone

Participants, as recipients, expected information on the drone’s delivery methods, and both recipients and bystanders were found to require information on the delivery location through a signal. Recipients imagined various delivery scenarios, such as the drone landing on the ground, dropping the package, hovering above and lowering the package with a cable, or even “giving it to my [recipient] hand” (P9). Recipients preferred to “(...) have more instructions” (P12) on the delivery methods. In addition, they wanted to be informed about whether they needed “to wait for the drone to land” (P8) and “how close I [recipient] can approach [the drone] or not” (P8).

In addition to the delivery methods, information on the delivery location, including a landing/take-off signal, was required for both recipient and bystander roles. This information was seen as essential for allowing recipients to prepare to collect the package and request a location change if deemed unsafe. Bystanders and external agents need to be notified that drones will be present near the delivery location, allowing them to decide whether to move to “less crowded space or empty space” (P1). Without this information, it is likely to be “hard to get people to feel happy and give them a safe feeling about the process” (P4).

##### 4.1.2.4 Limited user intervention in drone control

Overall, recipients and bystanders did not prefer to take over the control of the drone. Bystanders showed no interest in interacting with or controlling the drone and would be annoyed if asked to do so, except in safety critical situations. In contrast, recipients were interested in passive interactions for basic functions, such as verifying delivery with a QR code: “I’ll see my snacks show up, scan my QR code, and I’m done with this” (P6). They were also willing to provide directional suggestions for safe landing if needed: “You just have to tell the drone to go more left, more right, and wait for something to drop” (P8).

#### 4.1.3 Drone design solutions to address uncertainty in HDI

Design solutions aligned with user expectations were proposed to address uncertainties in interactions with delivery drones in public parks. Table 4 shows that solutions for *drone appearance* and *HMIs* (excluding phones) were mentioned almost equally for both roles. However, the phone interface was required by all participants for the recipient role but mentioned by only one for the bystander role.

**TABLE 4:** Number of participants mentioned design solutions during the interviews for the roles of recipient and bystander.

Drone design solutions to address uncertainty in HDI		Recipient	Bystander
Drone appearance	Colors and stickers	7	7
	Friendly Looks	6	6
Human-machine interfaces to communicate drone intentions	Lights	4	5
	Projection	3	4
	Display	2	2
	Non-vocal message	6	6
	Vocal message	4	2
	Phone	12	1

##### 4.1.3.1 Drone appearance

Participants expressed the need for the drone’s design to be interaction-friendly and aligned with its delivery purpose, as its appearance impacts their emotions and uncertainties. The majority suggested using colors and brand stickers on the drone’s body to indicate its delivery purpose and adopting a friendly design to enhance safety and approachability. Colors and stickers were deemed as effective for attracting attention, aiding in the identification of the drone’s purpose from a distance, and reducing uncertainty. Participants highlighted the need for intuitive color use and visibility considerations based on the delivery location’s geography: “If it is about to land in a park, it is gonna be preferable to not be green, for example, (...) [If] I see something red, maybe it is something to not approach” (P8).

Drawing from familiar food delivery services like DHL and UberEats, it was suggested that colors and brand stickers be used on both drones and packages to enhance identification and reduce uncertainty. Participants criticized “classical drones” as “very ugly” (P11) and “mechanical or masculine” (P12), recommending features like propeller guards, rounded shapes, and the avoidance of sharp edges to create a sense of safety. Some proposed that a package attached beneath the drone could signal its delivery function.

##### 4.1.3.2 Human-machine interfaces to communicate drone intentions

Participants mentioned the use of HMIs, including audio and visual elements on the drone, for communicating the drone’s intentions and establishing a connection with recipients and bystanders. A participant suggested that the use of visual and audio interfaces should depend on the distance between the drone and the user: “Maybe when it [drone] is far, it [drone] can have lights and when it [drone] is close by, it can be sound” (P12). A few participants recommended using multiple HMIs to provide redundancy and inclusivity for physically challenged users. However, one participant advised against overloading the user with too many interfaces.

The suggested visual interfaces include lights, projections, and displays attached to the drone. Lights were intended to communicate landing/take-off intentions, maintain safety distance, identify the recipient’s drone, and indicate video recording. Projections on the ground were primarily meant to signal the landing location, while displays were designed to help identify the “correct” drone by showing the recipient’s information. Visual interface advantages include: “If it is just LED lights, it is simple and it can be seen from far away” (P12), and with a display, “it is easy to change on the drone, different recipients, different names” (P11). A few participants raised practical concerns about the visibility of visual interfaces: “if it is really bright Sun and in a park, I don’t know how easy is it to see on the drone what is happening” (P8).

The audio interfaces were identified as including both vocal and non-vocal messages. Vocal messages were recommended to convey the drone’s purpose, landing/take-off intentions for safety, and to communicate with bystanders: “By language, tell everybody: I’m landing, I’m landing (...) If it [drone] needs help then I would expect voice of sentences rather than just [non-vocal] sounds” (P10). Some participants found vocal messages to be intrusive, intimate, and difficult to hear in “open environments” (P2). They recommended using “minimal” (P12) non-vocal messages to communicate basic intentions, such as the drone’s presence, landing/take-off intentions, and post-delivery acknowledgment. The library of non-vocal messages included: “(...) beeping with a low frequency” (P5), “noise that washing machine does sometimes, when it is finished” (P11), and “turning off laptop sound” (P12). While audio interfaces were found to add value by grabbing the user’s attention, especially when preoccupied, some participants “imagine [that], in public space, sound [audio] would be too intrusive” (P12).

An application on the recipient’s mobile device was a popular suggestion for receiving timely and specific information about the delivery process and drone’s intentions. Phone application (henceforth referred as phone) was considered functional and simple to use for everyone. The use of the phone was mentioned to reduce the necessity for HMIs on the drone, such as displays and vocal messages, while maintaining privacy. As recipients, participants wanted to receive information on their phones regarding tracking, identification and verification with the drone, precise landing/take-off locations, tutorials on interaction procedures and delivery methods:

“I can track on my phone or the device, how far it is and where it flies, the current location of the delivery” (P3).

“The app will tell you that the drone is like number 27. There is a number on the drones body, also showing number 27, for you to identify, and then see if it is the right one” (P10).

As bystanders, a majority of participants were not comfortable using the phone “(...) to know about the drones” (P10) and preferred to “shift most of the interaction to the drone and move away from the app” (P1). A participant, however, mentioned the need for an application to locate the drones in public space: “I would like to know, if I go somewhere and see that there are many deliveries about to happen where I am and I don’t want to have the sound, I would like to go somewhere else” (P8).

##### 4.1.3.3 Use-case dependent

Participants expressed the need for design solutions to reflect the situation criticality as the uncertainties and the expectations differed: “We should have [the emergency drone] more distinguishable, so people around also know that this is for a medical reason and it is not like a bag of chips or my Amazon [package]” (P8).

The relevance of HMIs and appearance was found to vary for emergency deliveries, in contrast to grocery package deliveries. A majority of participants suggested incorporating colors, stickers, lights, and sirens similar to those on ambulances to convey the urgency of the situation. Audio interfaces were considered less intrusive given the criticality of the situation and were deemed necessary to “notify that something [emergency] is happening” (P8) and to request bystanders to move away or assist with the emergency: “It can be like an audio announcement saying, Hey, this person [recipient name] is stuck in this [specific] place, this person [recipient name] needs help” (P7).

### 4.2 Focus group results

**FIGURE 3:**
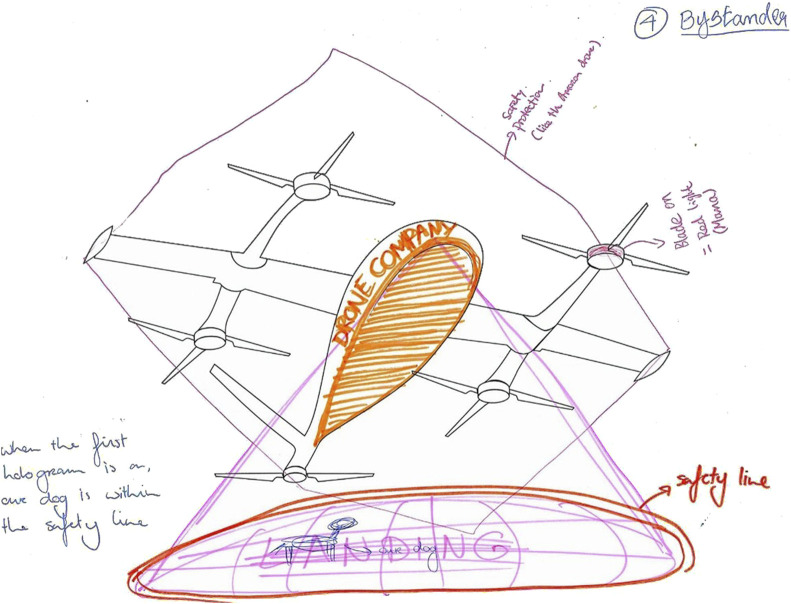
Example sketch of a drone, created by Group 4, for the bystander role.

**FIGURE 4:**
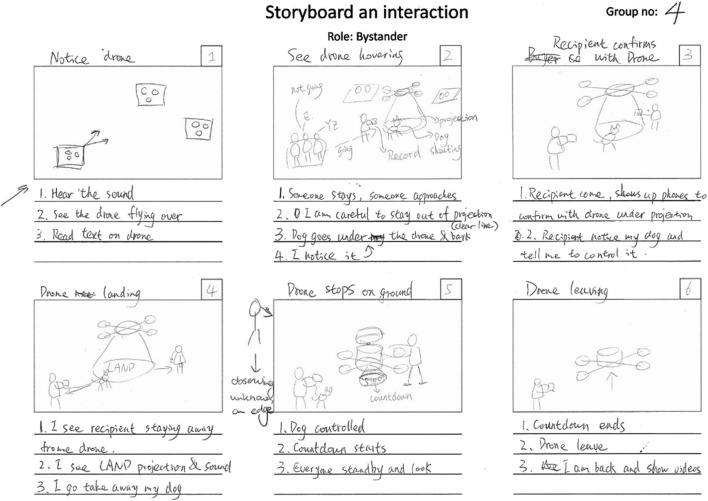
Example sketch of a storyboard, created by Group 4, for the bystander role.

The thematic analysis of drone sketches and storyboards produced design solutions and interaction procedures aligned with user requirements. Figures 3, 4 illustrate example sketches of a drone and an interaction storyboard, respectively. The drone sketches indicated the types of design solutions, while the storyboards detailed the stages of interaction and the application of the design solutions. Reflections on existing drone models were included, with focus group results labeled as “G” followed by the corresponding group number.

#### 4.2.1 Drone sketches

**FIGURE 5:**
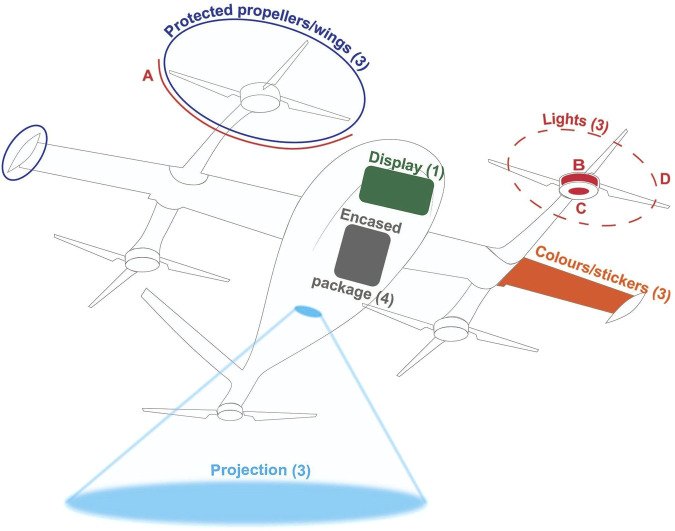
Sample display of design solutions proposed by participants using drone sketches for the recipient role. Numbers in brackets represent the number of group suggestions for each solution. For lights, **(A–D)** denote the light interfaces around the protected propellers, on the motor, on the motor mount, and on the propellers, respectively.

**FIGURE 6:**
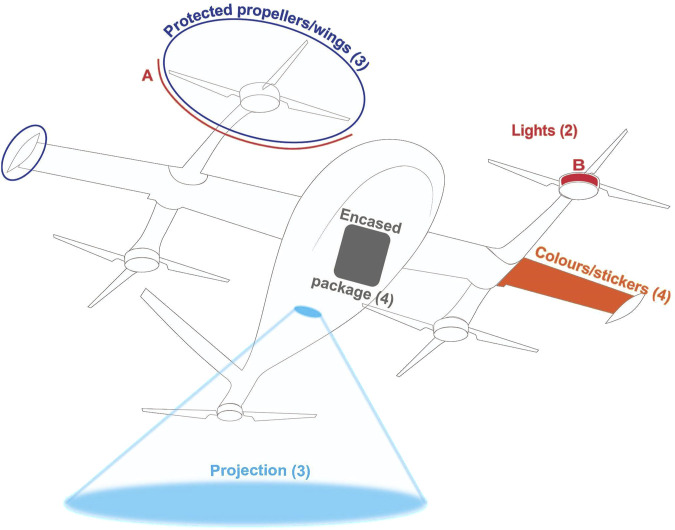
Sample display of design solutions proposed by participants using drone sketches for the bystander role. Numbers in brackets represent the number of group suggestions for each solution. For lights, **(A, B)** denote the light interfaces around the protected propellers and on the motor, respectively.

Design solutions included protected propellers/wings, encased packages, colors/stickers, lights, displays, and projections for safety, recognition and landing/take-off intentions (see Figures 5, 6).

Participants suggested using protection around the propellers and wings (G1, G2, G3) to enhance perceived safety for those in the vicinity of the drone. Encasing the package inside the drone (G1, G2, G3, G4) was recommended for package safety, while placing lights around the propellers (G2) and on the motor (G4) was advised to help recipients and bystanders identify the propellers and maintain a safe distance, reducing safety concerns when the drone enters a public park space for delivery.

Colors and (brand) stickers were suggested on the body and wings of the drone to indicate the delivery purpose, with two groups (G1, G3) recommending this for recipients and four groups (G1, G2, G3, G4) for bystanders. Lights on the propellers (G3), a display attached under the nose of the drone (G2), and ground projections (G4) were recommended to help recipients identify their respective drones. The visual interfaces were intended to work in conjunction with the phone; for example, the recipient receives a delivery-specific code on their phone that is reflected on the visual interfaces. For the package verification process, a QR code was recommended as either a sticker on the drone (G2) or a projection on the ground (G4), which recipients could scan with their phones. Ground projections (G1, G2, G4) were recommended to indicate landing/take-off intentions to both recipients and bystanders, while lights on the motor mount (G3) were suggested specifically for recipients.

#### 4.2.2 Storyboards

Based on the thematic analysis of storyboards, the stages of interaction were identified as Order and Ship, Arrival, Delivery, and Retract for recipients, and Arrival, Delivery, and Retract for bystanders. Table 5 indicates that appearance related solutions were mentioned only for the Arrival stage. Lights were noted for recipients across the Arrival, Delivery, and Retract stages, but only during the Retract stage for bystanders. Projection was identified for recipients in the Arrival and Delivery stages, while for bystanders, it was relevant only during the Delivery stage. All groups utilized phone interface cards for the recipient role, but none did so for the bystander role. The uncertainties were reported below for every stage. The following text (reported often in past tense form) outlines the interaction stages based on the storyboarding results, reflecting participants’ expectations rather than actual experiences.

**TABLE 5:** Number of groups mentioned design solutions for each human role and each stage of interaction. R and B represent recipient and bystander roles, respectively.

Design solutions		Stages of interaction and human roles							
		Order and ship		Arrival		Delivery		Retract	
		R	B	R	B	R	B	R	B
Appearance	Colors and stickers	0	0	2	4	0	0	0	0
	Friendly Looks	0	0	3	3	0	0	0	0
HMIs	Lights	0	0	1	0	1	0	1	1
	Projection	0	0	1	0	3	3	0	0
	Display	0	0	1	0	0	0	0	0
	Audio message	0	0	0	0	2	1	1	1
	Phone	4	0	2	0	4	0	1	0

During the Order and Ship stage, the recipient places the delivery order and receives tracking information.

Participants were uncertain about the delivery process and methods of interaction. The order was placed on the phone by the recipient with the required details similar to the current food delivery applications (e.g., UberEats; G1, G2, G3, G4). Once the order was accepted, the recipient received the delivery tracking information, such as order details (G1, G2, G3, G4), instructions on the interaction procedure (G3, G4), estimated time of arrival (G1, G2), flying route (G2, G3), and drone identification details (G2, G3) on the phone. While the drone flew to the delivery address (G1, G2, G3, G4), the recipient waited in anticipation.

In the arrival stage, the recipient identifies the drone, whereas the bystander identifies the recipient, drone’s presence and purpose.

Participants (G3) were uncertain about being unable to identify the drone and were concerned about the drone attracting attention and eliciting reactions from bystanders and external agents (G2, G4).

Notifications were received on the phone when the drone was close by, which helped the recipient prepare for the interaction (G1). As the drone arrived in the park, it was identified by the recipient using a code through lights on the propellers, colors/stickers, display, or projection (G2, G3, G4). If not identified, the service was contacted via phone (G3). Propeller guards/wings and lights on the propeller guards and motor were used to reflect a “safe” interaction (G1, G2, G4). Once identified, the drone either located a precise delivery location (G2, G3, G4) or the recipient selected a location from those suggested on the phone (G1) and moved to that location. The responsibility for identifying the delivery location was handed to the drone by three groups and to the recipient by one group.

Bystander identified the recipient in the vicinity and the presence of the approaching drone with propeller noise and its purpose through colors/stickers (G1, G2, G3, G4). Different reactions were observed from bystanders (and external agents): some were curious and approached the drone, some were annoyed and sought drone-free areas, and some were tempted to place an order later (G2, G4).

During the Delivery stage, the recipient identifies the delivery location, verifies with the drone and handles the delivery procedure, while the bystander observes from afar.

Participants expressed uncertainty about the delivery location and concerns regarding bystanders and external agents potentially interrupting the delivery process by entering the interaction space (G2, G4).

The delivery process was initiated by the recipient after phone confirmation (G1, G3, G4). The delivery location was highlighted by the drone through lights or a projection on the ground while it hovered above and observed by the recipient (G1, G2, G4). The package was then delivered either by landing (G1, G2, G4) or by using a cable to drop it (G3), with lights on the motor mount and audio used as a landing signal (G4). The recipient was provided the ability to terminate the delivery process via phone (G1), if deemed unsafe. Verification with the drone was performed by the recipient either before the delivery by scanning a QR code sticker on the drone (G2) or as a projection (G4), or after the delivery by confirming on their phone (G1, G3).

The delivery location was highlighted by the drone with a projection on the ground (G1, G2, G4), and the bystanders and external agents were expected to stay away or not to avoid hindering the delivery process. The bystander showed little concern about the delivery method (G1, G2, G3). The drone delivered the package with projection and audio used as a landing signal (G4). In scenarios where hindrance was possible, one group (G4) indicated that the recipient was responsible for clearing the hindrance and preventing interruption of the delivery process, while three groups (G1, G2, G3) suggested that the responsibility lay with the drone rather than the recipient.

In the retract stage, the recipient and bystander observe the drone retracting and flying away from their vicinity.

Participants were uncertain about potential interruptions to the retracting process by bystanders and external agents (G2, G4) and about the drone retracting before the recipient completed the package collection (G4).

After the delivery, the recipient scanned the area for a safe take-off and confirmed readiness via phone (G2), or the drone autonomously scanned the area for a safe take-off without recipient involvement and waited for a fixed duration for package pickup (G4). While not explicitly mentioned by two groups (G1, G3), the responsibility for ensuring a safe take-off was assigned to the drone by one group (G4) and to the recipient by another group (G2).

Before the drone retracted, the recipient and bystander observed the drone using lights on the motor or an audio message to request clearing the landing location (G2, G4). The location was vacated by recipients, bystanders, and any external agents present, and the drone retracted.

#### 4.2.3 Reflection on the existing drone models

Overall, participants found the current designs of Amazon, Mana, Wing, and Zipline drones to be more suitable for home deliveries with open spaces, where recipients are familiar with the exact delivery location and purpose, rather than for public park deliveries. The perceived differences in drone sizes led participants to associate them with different use cases; for example, the Amazon drone was seen as large and suitable for delivering bigger packages, while the smaller Mana drone was deemed more appropriate for snack deliveries (G1). The dead-drop delivery method onto designated landing pads raised safety concerns (G1, G2, G3, G4), and the necessity of carrying landing pads in parks was criticized due to the efforts required to transport the pads (G1, G2). The visible package on the Wing drone and the cable delivery methods of Wing and Mana led participants to believe that these drones would perform well in pleasant weather but could struggle in harsh wind conditions (G1, G2, G3). Participants recommended encased packages for safety, as seen in the designs of the Mana and Zipline drones (G1, G2, G3).

Participants criticized the Mana and Wing drone designs for lacking propeller and wing protection (G1, G3) and found the Amazon drone’s protective design to be sturdy and safe (G1, G2, G3, G4). Wing drone was not preferred for its unprotected propellers, intrusive, sharp-edged, and mechanical appearance (G1, G2, G3, G4). The intimidating size of the Amazon drone discouraged hovering during the delivery phase (G1, G2, G3, G4). The Zipline drone design was viewed as safe and appealing due to its futuristic design and mini-droid feature (G1, G2, G3, G4), which allowed precise package delivery and was deemed stable for windy conditions (G1, G3). Participants recommended the design focus to shift from the main drone to the mini-droid, suggesting zoomorphic features for improved user appeal (G1, G3).

## 5 Discussion

Our user-centered design study, using individual interviews and focus groups, identified key uncertainty factors, user requirements, and design solutions for delivery drones interacting with humans in public spaces like parks. Among the uncertainty factors, participants highlighted how uncertainty levels varied depending on human roles and the dynamic nature of public spaces. Bystanders, who rarely expect engagement with drones, experience higher uncertainty compared to recipients, who anticipate deliveries and face fewer uncertainties. Bystanders expressed uncertainties regarding the drone’s purpose, noise, and the delivery location. In contrast, recipient uncertainties focused on safety of themselves and bystanders, drone flying behavior, identifying their service drone, and determining their positioning for a safe pickup. While previous studies have individually studied the roles of bystanders (Pelikan et al., 2024; Yu et al., 2024) and recipients (Tan et al., 2018; Yeh et al., 2017) in HRI, our study offers a novel contribution by discussing the interplay between these roles, the differing levels of uncertainty experienced with both the roles and presenting their underlying causes. Future research in HRI should focus more on codesign that incorporates both recipient and bystander perspectives to improve public acceptance of service robots in public spaces.

Participants explained that current commercial drone designs (e.g., Amazon Prime, Mana, Wing, Zipline) are better suited for home deliveries than public spaces, where uncertainty is expected to be greater. The evolving dynamics of public spaces—such as multiple recipient groups, pets, and environmental factors—contribute to this uncertainty, complicating interactions and raising safety concerns. While Nielsen et al. (2023) found that over 60% of interaction breakdowns with public service robots are due to environmental disturbances, our study extends this by identifying the specific causes of uncertainty stemming from public space dynamics. Future research should conduct naturalistic studies involving public interactions with drones, investigating how factors like multiple recipient groups, pets, and environmental elements influence uncertainties for both recipients and bystanders. This will help guide the design of safer drones for public environments.

Uncertainties varied across different stages of interaction, including Order and ship, Arrival, Delivery, and Retract, aligning with the findings of Lingam et al. (2025) for the Arrival, Delivery, and Retract stages. Consequently, user requirements differed, prompting tailored design recommendations to manage uncertainties in each stage.

### 5.1 Order and ship stage

The interaction with the recipient begins passively even before the drone arrives, building expectations that help manage uncertainties. Recipients expressed uncertainty about the delivery methods, drone size, and interaction procedures, highlighting the need for tutorials that outline the process and align their mental models prior to the drone’s arrival. It is recommended to share standardized tutorials alongside package tracking information on the phone application where the order is placed. This underscores the need to provide information to build recipient expectations before the onset of HDI, a novel observation that adds to the current HDI studies (Bevins and Duncan, 2021; Obaid et al., 2015; Tan et al., 2018; Yeh et al., 2017) that have primarily focused on the interaction element.

Recommendation 1: Recipients should be informed on the interaction protocols via phone before the drone arrives.

### 5.2 Arrival stage

According to Lingam et al. (2025), recipients experience the most uncertainty during the Arrival stage. Our study reveals that recipients may struggle with identifying the “correct” drone, particularly in the presence of multiple recipient groups, while bystanders may feel uncertain about the drone’s presence and purpose. These uncertainties cause interruptions and safety issues, particularly with unpredictable behavior of bystanders or external agents. In order to address these concerns, drones should use visual aids like stickers and colors to indicate purpose and non-vocal audio cues, such as propeller noise, for presence. This recommendation based on our user-centered study aligns with the expert recommendations of Lingam et al. (2024). It is further recommended to draw design inspiration from delivery and taxi services, incorporating familiar elements such as bike or taxi designs and application interfaces, to reduce uncertainty.

Designs should prioritise safety and convey “friendly” intentions, incorporating protective features around propellers and wings, contrary to the recommendation by Wojciechowska et al. (2019) to exclude propeller guards. This difference arises because Wojciechowska et al. (2019) used static images of similarly sized drone models without providing context on their delivery use case, whereas our study presented information and videos that conveyed both the delivery context and an estimate of drone size. This highlights the importance of informing the public about the delivery context and drone characteristics to better understand their needs on design elements and reduce uncertainty in public spaces.

Standardized visual and audio cues can help humans identify the drone’s purpose and presence, while customized interfaces can assist recipients in distinguishing the “correct” drone. While previous studies (Tan et al., 2018; Wojciechowska et al., 2019; Yeh et al., 2017) provided design recommendations for one-to-one HDI, our study uniquely contributes by recommending customizable cues that facilitate drone identification as the scale of drones and of recipient orders increase. The above design solutions should be tested in user studies to assess how quickly the solutions instill a sense of certainty among users.

Recommendation 2: Humans should be informed about the drone’s purpose through the appearance, presence by propeller noise, and identification through appearance and visual interfaces when the drone arrives.

### 5.3 Delivery and retract stage

During this stage, uncertainty primarily stems from difficulties in identifying the delivery location and concerns about bystanders and external agents interrupting the delivery process, which can lead to safety issues and interaction breakdowns. Lingam et al. (2025) found that recipients experience high levels of uncertainty before the drone attempts to deliver and retract. It is recommended that drones use ground projections to indicate delivery locations and provide audio warnings for safety, inline with Lingam et al. (2024). Previous user studies in public spaces have used projection to receive recipient input for task execution (Cauchard et al., 2019) or to guide recipients in discarding garbage by highlighting it (Obaid et al., 2015). Our study adds value by incorporating the perspective of bystanders and recommending the use of projection to communicate drone intentions about occupying ground space (e.g., package drop-off spot), thereby reducing uncertainty and guiding humans to maintain a safe distance.

The phone application should include a customised feature that allows recipients to verify and, if necessary, interrupt deliveries for safety. This aligns with the current safety standards of global delivery companies, such as UPS (2025) and DHL (2025), which use recipient verification by delivery truck drivers to prevent misplaced deliveries. Extending this approach to the context of automated drone delivery using a mobile device for recipient verification is a novel finding. Lights and audio warnings should also be integrated into the drone’s design to inform humans to maintain a safe distance during the drone’s retraction. Standardized visual and audio interfaces for landing and take-off intentions would allow recipients, bystanders, and other external agents to quickly and accurately understand the drone’s intentions, reducing uncertainty, and enhancing safety. Future research should evaluate which interfaces (e.g., lights versus audio messages) are most effective for communicating specific landing or take-off information, including notifying recipients about package collection and alerting bystanders to keep a safe distance.

Recommendation 3: Humans should be informed of the delivery location through ground projections, and the drone should only proceed with the delivery after receiving confirmation from the recipient via phone. Additionally, lights and audio warnings should be employed to keep bystanders and external agents at a safe distance.

Participants are divided on defining control responsibilities during the Arrival, Delivery, and Retract stages. Some felt that recipients should be responsible for identifying the delivery location and ensuring safe landing and take-off, while others expressed that the drone should handle these tasks. This division may stem from a lack of familiarity with drone capabilities in public spaces. Unclear control responsibilities can increase uncertainty and miscommunication for humans, leading to interaction breakdowns during critical moments. Future studies should explore how humans respond to different control assignments, whether to the drone, the recipient, or shared between them, examine the resulting uncertainties, and work towards standardizing control responsibilities to enhance safety.

### 5.4 Limitations and future research

Although the authors in our study made efforts to minimize confirmation bias and enhance diversity in the interpretation by involving multiple coders, thematic analysis inherently involves subjective interpretation of the data (Braun and Clarke, 2006). The qualitative nature of the research introduces potential biases, which could influence the results and limit the generalizability of the findings. Future research should examine the extent to which the viewpoints presented in our study can be generalized to the broader public by conducting large-scale studies on HDI in simulated environments. Additionally, employing mixed methods, involving Likert scale statements or behavioral observations around drones, could provide objective measures to complement the qualitative insights.

Interviews and focus groups required participants to envision drone interactions, and their limited experience with drones may have influenced their ability to propose effective design solutions. Additionally, the proposed design solutions need empirical validation. Future research should refine these solutions into prototypes or conceptual models and evaluate them through user testing in virtual environments or real-world experiments, for delivery scenarios involving both recipients and bystanders.

While our study reflects the limited exposure to drone deliveries, representing the perspective of novice users in public spaces, it is limited in considering the perspective of experienced users, such as drone pilots. Such users could deepen the observations by reflecting on their experiences with interaction scenarios, challenges, HMI requirements, and their implications. Future research should extend our work by including participants with experience flying drones.

The participants’ design background enabled them to formulate concrete design solutions with rationale; however, it might have biased their suggestions toward existing design principles. To minimize this bias and improve the validity, future research should develop concepts based on our design recommendations and evaluate them with participants from non-design backgrounds.

Participants in our interviews stressed designing systems based on situation criticality and transitions from bystander to recipient roles, as user needs vary with urgency. While our study offered preliminary design directions, the focus groups did not comprehensively address this aspect due to time constraints. It is recommended to explore how varying levels of situation criticality impact user requirements for both recipients and bystanders, and develop design recommendations for HDI in public spaces.

## 6 Conclusion

Our user-centered design study, conducted through interviews and focus groups, identified key uncertainty factors and user requirements, providing design recommendations for interactions between delivery drones and humans (recipients and bystanders) in public parks. It is crucial to address these aspects for the effective integration of delivery drones into public environments. The study identified that information needs and preferred interface modalities vary between recipients and bystanders and across different interaction stages. The study highlighted the necessary design features that require standardization and customisation to support the development of effective design guidelines and improve natural HDI in public spaces. Drone designers may face the challenge of implementing these features and addressing human requirements, particularly related to uncertainty and safety concerns. Prior to the implementation, future research should validate the proposed recommendations through experimental studies involving interactions between different human roles and delivery drones. Furthermore, research is necessary to identify which interfaces are most effective for conveying specific types of information and facilitating communication.

## Data Availability

The data analysed in the study are included in the Supplementary Material. Further inquiries can be directed to the corresponding author.
