# Supplementary material for: Human interactions with delivery drones in public spaces: design recommendations from recipient and bystander perspectives
Source: Front Robot AI. 2025 May 30;12:1580289. doi: 10.3389/frobt.2025.1580289 (PMC12162322; doi:10.3389/frobt.2025.1580289)
Supplement: Supplementary file 1 [file DataSheet1.zip › Data_&_results/Focus_groups/Reflections_on_existing_drone_models/FG_reflection_codes.pdf]

02-09-2024

Codes for 'reflections on exisiting drone models'

Table of contents

1. Documents 3
2. Design considerations of mini-droid 4
3. Wing's poor design 5
4. Zipline minin-droid advantages 7
5. Discouraged hovering 9
6. Encased package safety 10
7. Evironmental effects on task/package safety 11
8. Landing pads criticism 13
9. Protection propellers & sturdy Amazon drone 14
10. Reflection on size 16
11. Safety concerns with dead drop 18

1. Documents

No.	Document (format: G(group_number))	Created by
1	G1	Analyst1
2	G2	Analyst1
3	G3	Analyst1
4	G4	Analyst1

Note: Participant quotes in each group are represented by “P”, followed by an (anonymized) ID number. Moderator quotes are represented by “M”, followed by an ID number. These representations are mentioned where applicable (e.g., discussion between multiple attendees).

2. Design considerations of mini-droid

1.

"Or the capsules could have a company logo.

P3 1:00:19

Or you can give it eyes."

Code: ● Design considerations of mini-droid Weight score: 0
G1, 582 - 584
Created: 29-08-2024 16:44 by Analyst1, Modified: 29-08-2024 16:44 by Analyst1
Area: 80 0.22%

2.

"The design of the drone or design of the pod inside the drone?

P2 1:26:35

That's not the inside, that's what I saw. It didn't open really which was the whole thing.

P3 1:26:45

I think, for instance, the drone that we draw could be this part.

M1 1:26:50

For the bystander or recipient?

P3 1:26:55

Maybe both, like the drone that we draw could be the pod and the main drone could be like industrial or whatever. But the thing that comes down that could be smarter.

P2 1:27:20

The flying part can just be the mechanical structure, standard design, but then you can change the shape itself."

Code: ● Design considerations of mini-droid Weight score: 0
G2, 792 - 802
Created: 29-08-2024 17:07 by Analyst1, Modified: 29-08-2024 17:07 by Analyst1
Area: 596 1.37%

3.

"It's a bit like, a cat was about to get out, honestly."

Code: ● Design considerations of mini-droid Weight score: 0
G3, 956 - 956
Created: 29-08-2024 17:30 by Analyst1, Modified: 29-08-2024 17:30 by Analyst1
Area: 54 0.08%

3. Wing's poor design

1.

"M1 56:29
And do you think it's a good idea for public spaces?

P2 56:33
That shape feels more intrusive actually.

M1 56:38
Why?

P2 56:40
it has a lot of many sharp points and the other one's (Mana) feels rounder and safer."

Code: ● Wing's poor design   Weight score: 0
G1, 543 - 550
Created: 29-08-2024 16:42 by Analyst1, Modified: 29-08-2024 16:42 by Analyst1
Area: 264   0.73%

2.

"The design looks very mechanical or industrial."

Code: ● Wing's poor design   Weight score: 0
G2, 750 - 750
Created: 29-08-2024 17:04 by Analyst1, Modified: 29-08-2024 17:04 by Analyst1
Area: 47   0.11%

3.

"In this specific one that the drone itself, pulling a gripper, I can imagine like in the future that oh, it's stuck. I will have to find a way, I cut the rope?"

Code: ● Wing's poor design   Weight score: 0
G2, 754 - 754
Created: 29-08-2024 17:04 by Analyst1, Modified: 29-08-2024 17:04 by Analyst1
Area: 159   0.37%

4.

"It looks industrial for delivering food, I expect more friendly, round."

Code: ● Wing's poor design   Weight score: 0
G2, 768 - 768
Created: 29-08-2024 17:06 by Analyst1, Modified: 29-08-2024 17:06 by Analyst1
Area: 71   0.16%

5.

"It looks like a toy plane.

P2 1:31:17
Yeah. It looks very plastic.

P3 1:31:22
I dont trust this drone (to do its job)."

Code: ● Wing's poor design   Weight score: 0
G3, 898 - 902
Created: 29-08-2024 17:26 by Analyst1, Modified: 29-08-2024 17:26 by Analyst1
Area: 122   0.19%

6.

"I feel like it's like most fragile"

Code: ● Wing's poor design   Weight score: 0
G3, 924 - 924

Created: 29-08-2024 17:26 by Analyst1, Modified: 29-08-2024 17:26 by Analyst1
Area: 34   0.05%

7.

"But the drone I don't like how it looks and"

Code: ● Wing's poor design   Weight score: 0
G3, 926 - 926
Created: 29-08-2024 17:26 by Analyst1, Modified: 29-08-2024 17:26 by Analyst1
Area: 43   0.07%

8.

"what do you think about the drone by Wing?

P2 1:30:31
I hate the design of this thing.

M1 1:30:34
Why?

P2 1:30:43
For the size of machine that it is, it looks like not enough material, because it's very thin elements that are just connected together. We all really like the encasing of the Amazon drone, there's something in that, consistency and the size versus how much material is there actually, that makes this look very flimsy. And honestly, my first association was, oh, it's two hockey sticks with, like, a connecting thing between them, and, like, if you also see it, it's tilting around a bit. I'm like, Oh, it doesn't look like it's actually a secure, safe thing to see flying around, especially at the sizes and speeds that we are talking about with these."

Code: ● Wing's poor design   Weight score: 0
G4, 876 - 882
Created: 29-08-2024 17:40 by Analyst1, Modified: 29-08-2024 17:40 by Analyst1
Area: 774   1.15%

4. Zipline minin-droid advantages

1.

"Because it has a capsule dropping. I think they consider the safety let this one (drone) stay in the air and dropping that capsule and for landing the package safe, I think that's very nice. Instead of just like with a rope to hang the package. I think this way is better."

Code: ● Zipline minin-droid advantages   Weight score: 0
G1, 568 - 568
Created: 29-08-2024 16:43 by Analyst1, Modified: 29-08-2024 17:31 by Analyst1
Area: 272   0.75%

2.

"Yeah, and even I think the next step could be to even make this capsule, it now looked a bit like a funny boat or something, look dome kind of avatar. You make it more human or like more fun instead of like these corporate drones that deliver boxes."

Code: ● Zipline minin-droid advantages   Weight score: 0
G1, 580 - 580
Created: 29-08-2024 16:44 by Analyst1, Modified: 29-08-2024 16:44 by Analyst1
Area: 249   0.69%

3.

"M1 1:01:34
Okay, yeah. And with regards to the logo being on the drone or or on the capsule, what do you think is a better solution for if the drone is being used in a public space where there is recipient and also the bystander?

P2 1:01:57
For both (it works). I think it's gonna land with that high distance, I prefer on the capsule."

Code: ● Zipline minin-droid advantages   Weight score: 0
G1, 591 - 594
Created: 29-08-2024 16:45 by Analyst1, Modified: 29-08-2024 17:31 by Analyst1
Area: 356   0.99%

4.

"P3 1:25:36
A small drone inside of a drone, nice."

Code: ● Zipline minin-droid advantages   Weight score: 0
G2, 775 - 776
Created: 29-08-2024 17:06 by Analyst1, Modified: 29-08-2024 17:06 by Analyst1
Area: 50   0.12%

5.

"the design of the whole carriage is kinda cute again"

Code: ● Zipline minin-droid advantages   Weight score: 0
G2, 782 - 782
Created: 29-08-2024 17:06 by Analyst1, Modified: 29-08-2024 17:06 by Analyst1
Area: 52   0.12%

6.

"Nice design in the first place."

Code: ● Zipline minin-droid advantages   Weight score: 0
G2, 790 - 790
Created: 29-08-2024 17:07 by Analyst1, Modified: 29-08-2024 17:07 by Analyst1
Area: 31   0.07%

7.

"M1 1:33:50
Okay, we go to the last one which is by zipline.

P2 1:33:55
Oh wow, this was quick.

P3 1:34:04
This looks stable.

P1 1:34:12
Too much future haha."

Code: ● Zipline minin-droid advantages   Weight score: 0
G3, 943 - 950
Created: 29-08-2024 17:28 by Analyst1, Modified: 29-08-2024 17:28 by Analyst1
Area: 164   0.25%

8.

"How it was delivered. Like if you compare the simplicity towards the other two videos we saw. This might work for like fragile deliveries, like I had concerns earlier or hard conditions, like stormy day."

Code: ● Zipline minin-droid advantages   Weight score: 0
G3, 982 - 982
Created: 29-08-2024 17:29 by Analyst1, Modified: 29-08-2024 17:29 by Analyst1
Area: 203   0.32%

9.

"The way that it has like an extra casing to deliver the goods looks quite futuristic in a way. It's quite different. It is good because you feel that your goods are protected.

P2 1:33:24
What I like about that is, a lot of the others we just saw, use some form of extra packaging, or specific packaging, to actually get it down safely, which we're also looking from a sustainability point of view, you're just adding a bunch of extra packaging materials, whereas here it's just an extra casing, but the package itself isn't altered or made sturdy or changed in any way whatsoever."

Code: ● Zipline minin-droid advantages   Weight score: 0
G4, 892 - 894
Created: 29-08-2024 17:42 by Analyst1, Modified: 29-08-2024 17:42 by Analyst1
Area: 582   0.86%

5. Discouraged hovering

1.

"No, I thought they will actually land."

Code: ● Discouraged hovering   Weight score: 0
G1, 446 - 446
Created: 29-08-2024 16:59 by Analyst1, Modified: 29-08-2024 16:59 by Analyst1
Area: 38   0.11%

2.

"Analyst1 47:06
And do you want to see such a drone in public park?

P2 47:11
It's too big.

P1 47:13
It's too big.

P3 47:15
Feels like an airplane in the park."

Code: ● Discouraged hovering   Weight score: 0
G1, 457 - 464
Created: 29-08-2024 17:00 by Analyst1, Modified: 29-08-2024 17:00 by Analyst1
Area: 176   0.49%

3.

"I don't want it to drop on my head."

Code: ● Discouraged hovering   Weight score: 0
G3, 764 - 764
Created: 29-08-2024 17:17 by Analyst1, Modified: 29-08-2024 17:17 by Analyst1
Area: 35   0.05%

4.

"I don't want it to hover above my head anymore because it looks a bit more intimidating than I thought the size and scale of it. So if it just suddenly runs out of fuel on top of my head, and not going to make it out alive."

Code: ● Discouraged hovering   Weight score: 0
G3, 796 - 796
Created: 29-08-2024 17:18 by Analyst1, Modified: 29-08-2024 17:18 by Analyst1
Area: 223   0.35%

5.

"P2 1:28:20
I think both for me are too big that I want them to hover above me."

Code: ● Discouraged hovering   Weight score: 0
G3, 865 - 866
Created: 29-08-2024 17:24 by Analyst1, Modified: 29-08-2024 17:24 by Analyst1
Area: 79   0.12%

6. Encased package safety

1.

"Actually (package) staying inside (the drone), makes more sense. What if it's bad weather and it rains. It's paper package and it gets wet easily."

Code: ● Encased package safety   Weight score: 0
G1, 542 - 542
Created: 29-08-2024 17:02 by Analyst1, Modified: 29-08-2024 17:02 by Analyst1
Area: 146   0.41%

2.

"I still go to the idea that it's inside of the drone and can open."

Code: ● Encased package safety   Weight score: 0
G2, 718 - 718
Created: 29-08-2024 17:03 by Analyst1, Modified: 29-08-2024 17:03 by Analyst1
Area: 66   0.15%

3.

"I mean when when my food is delivered, the compartment opens and then I pick my food."

Code: ● Encased package safety   Weight score: 0
G2, 722 - 722
Created: 29-08-2024 17:03 by Analyst1, Modified: 29-08-2024 17:03 by Analyst1
Area: 85   0.20%

4.

"P1 1:27:23
Both of them have things inside the drone, so it feels more protected.

P3 1:27:33
Interesting that it opened up like this and the package was not outside"

Code: ● Encased package safety   Weight score: 0
G3, 853 - 856
Created: 29-08-2024 17:23 by Analyst1, Modified: 29-08-2024 17:23 by Analyst1
Area: 167   0.26%

5.

"With that size, it makes to be inside and to not drop somewhere.

P3 1:29:12
Else the package breaks."

Code: ● Encased package safety   Weight score: 0
G3, 870 - 872
Created: 29-08-2024 17:25 by Analyst1, Modified: 29-08-2024 17:25 by Analyst1
Area: 102   0.16%

7. Evironmental effects on task/package safety

1.

"P3 51:43
That's why they stick in the air.

P2 51:45
But it's very shaky. Imagine, if they order a soup.

P2 51:49
It will be shaken too.

P2 51:53
If it's like a restaurant delivery and what if it is a birthday cake, then the cake is damaged."

Code: ● Evironmental effects on task/package safety Weight score: 0
G1, 503 - 510
Created: 29-08-2024 16:41 by Analyst1, Modified: 29-08-2024 16:41 by Analyst1
Area: 247 0.69%

2.

"M1 55:18
Yeah, what do you think about the video by Wing? What do you think about the way it appears or the whole interaction itself?

P2 56:17
Actually (package) staying inside (the drone), makes more sense. What if it's bad weather and it rains. It's paper package and it gets wet easily."

Code: ● Evironmental effects on task/package safety Weight score: 0
G1, 539 - 542
Created: 29-08-2024 16:42 by Analyst1, Modified: 29-08-2024 16:42 by Analyst1
Area: 310 0.86%

3.

"it brings confusion and also about the rope sometimes it can be tangled. In the video it was mostly smooth but if there is some tree so there is reason to be tangled."

Code: ● Evironmental effects on task/package safety Weight score: 0
G1, 556 - 556
Created: 29-08-2024 16:42 by Analyst1, Modified: 29-08-2024 16:42 by Analyst1
Area: 166 0.46%

4.

"I think I cannot ordering if it's raining or windy. This would be a problem.

M1 1:18:21
And why?

P3 1:18:24
Because the thing that is carrying my foods, the cable, I don't know if it's strong enough to keep my food safe on a windy day."

Code: ● Evironmental effects on task/package safety Weight score: 0
G2, 714 - 718
Created: 29-08-2024 17:02 by Analyst1, Modified: 29-08-2024 17:03 by Analyst1
Area: 239 0.55%

5.

"I would also agree with the point, P3 made, that if it is just a string, I am uncertain. If it is two or other strings like holding it like on four corners or something I would prefer that but with

one string and if it's the windy path or something and if it's like even an emergency situation then I am not certain."

Code: ● Evironmental effects on task/package safety   Weight score: 0
G2, 734 - 734
Created: 29-08-2024 17:03 by Analyst1, Modified: 29-08-2024 17:03 by Analyst1
Area: 316   0.73%

6.

"In this specific one that the drone itself, pulling a gripper, I can imagine like in the future that oh, it's stuck. I will have to find a way, I cut the rope?

P2 1:23:23
That's true.

P3 1:23:25
I can predict some problems.

P1 1:23:30
I'm really unlucky with some aspects. If the gripper doesn't work, it's like boom (destruction) for me. If the gripper doesn't open, you cannot do anything or you need to cut it off or do you need to do it."

Code: ● Evironmental effects on task/package safety   Weight score: 0
G2, 754 - 760
Created: 29-08-2024 17:04 by Analyst1, Modified: 29-08-2024 17:05 by Analyst1
Area: 447   1.03%

7.

"it's in the Netherlands, maybe we need to worry about the wind and everything but in other places, for example, in California, I would imagine this could work."

Code: ● Evironmental effects on task/package safety   Weight score: 0
G2, 762 - 762
Created: 29-08-2024 17:06 by Analyst1, Modified: 29-08-2024 17:06 by Analyst1
Area: 159   0.37%

8.

"Is it just me that it seems wobbly."

Code: ● Evironmental effects on task/package safety   Weight score: 0
G3, 896 - 896
Created: 29-08-2024 17:26 by Analyst1, Modified: 29-08-2024 17:26 by Analyst1
Area: 35   0.05%

9.

"Don't you feel like, it could be a big wind and it just swings and puff (breaks)."

Code: ● Evironmental effects on task/package safety   Weight score: 0
G3, 934 - 934
Created: 29-08-2024 17:27 by Analyst1, Modified: 29-08-2024 17:27 by Analyst1
Area: 81   0.13%

8. Landing pads criticism

1.

"I'm also not a fan of dropping the package on the landing pad."

Code: ● Landing pads criticism   Weight score: 0
G1, 472 - 472
Created: 29-08-2024 16:35 by Analyst1, Modified: 29-08-2024 16:35 by Analyst1
Area: 62   0.17%

2.

"I didn't like, that I need to have something (landing pad) also with me. For instance, if thing flies away, I don't have any space to receive my delivery."

Code: ● Landing pads criticism   Weight score: 0
G2, 660 - 660
Created: 29-08-2024 16:53 by Analyst1, Modified: 29-08-2024 16:53 by Analyst1
Area: 154   0.35%

3.

"but that's for package delivery at home. So I can imagine that's okay. If you're at home.

P3 1:11:59
Yeah, but me I have to have.

P2 1:12:01
But if you're somewhere else.

P3 1:12:04
Yeah. Or if it flys away, I don't have the space anymore to receive my package."

Code: ● Landing pads criticism   Weight score: 0
G2, 662 - 668
Created: 29-08-2024 16:53 by Analyst1, Modified: 29-08-2024 16:54 by Analyst1
Area: 267   0.61%

9. Protection propellers & sturdy Amazon drone

1.
"I like how the drone designed. It's very safe. Like the outer fence (and the package is) inside just like these drones (from the sketches)"
Code: ● Protection propellers & sturdy Amazon drone Weight score: 0
G1, 454 - 454
Created: 29-08-2024 16:28 by Analyst1, Modified: 29-08-2024 16:28 by Analyst1
Area: 138 0.38%

2.
"the shape or the fillings (protective gaurds) is good."
Code: ● Protection propellers & sturdy Amazon drone Weight score: 0
G1, 468 - 468
Created: 29-08-2024 16:34 by Analyst1, Modified: 29-08-2024 16:34 by Analyst1
Area: 54 0.15%

3.
"I don't like, so their fans have no protection. So it's very dangerous if they actually land."
Code: ● Protection propellers & sturdy Amazon drone Weight score: 0
G1, 502 - 502
Created: 29-08-2024 16:36 by Analyst1, Modified: 29-08-2024 16:36 by Analyst1
Area: 93 0.26%

4.
"I think the drone itself should be protected. So it can actually land as well. Then the children wont accidentally touch the fan."
Code: ● Protection propellers & sturdy Amazon drone Weight score: 0
G1, 518 - 518
Created: 29-08-2024 16:39 by Analyst1, Modified: 29-08-2024 16:39 by Analyst1
Area: 129 0.36%

5.
"the surrounding for example, like it already covers part of the thing. Indeed, and makes it look more safer or daily use friendly"
Code: ● Protection propellers & sturdy Amazon drone Weight score: 0
G2, 692 - 692
Created: 29-08-2024 16:55 by Analyst1, Modified: 29-08-2024 16:55 by Analyst1
Area: 129 0.30%

6.
"I like how it looks though, sturdy. The shape I think."
Code: ● Protection propellers & sturdy Amazon drone Weight score: 0
G3, 812 - 812
Created: 29-08-2024 17:20 by Analyst1, Modified: 29-08-2024 17:20 by Analyst1
Area: 54 0.08%

7.
"think in my, its safety if something goes wrong but looks safer I think when it's like covered."
Code: ● Protection propellers & sturdy Amazon drone Weight score: 0
G3, 864 - 864
Created: 29-08-2024 17:24 by Analyst1, Modified: 29-08-2024 17:24 by Analyst1
Area: 95 0.15%

8.
"in the other two (Mana and Wing), you have to be protected rather"
Code: ● Protection propellers & sturdy Amazon drone Weight score: 0
G3, 928 - 928
Created: 29-08-2024 17:35 by Analyst1, Modified: 29-08-2024 17:35 by Analyst1

Area: 65   0.10%

9.

"Positive thing is that they have, like the thing, whole wrapped so you barely see the blades."

Code: ● Protection propellers & sturdy Amazon drone   Weight score: 0
G4, 814 - 814
Created: 29-08-2024 17:37 by Analyst1, Modified: 29-08-2024 17:37 by Analyst1
Area: 93   0.14%

10.

"For both, they (propellers and wings) should be wrapped."

Code: ● Protection propellers & sturdy Amazon drone   Weight score: 0
G4, 820 - 820
Created: 29-08-2024 17:37 by Analyst1, Modified: 29-08-2024 17:37 by Analyst1
Area: 56   0.08%

10. Reflection on size

1.

"It can have functions to deliver, like the large package. But like for personal use, it can have like different types of drones, maybe the most times like 80% needs, small drones can deliver the small goods. And maybe this is only for the the rest of the 20% (large goods)."

Code: ● Reflection on size   Weight score: 0
G1, 468 - 468
Created: 29-08-2024 16:35 by Analyst1, Modified: 29-08-2024 16:35 by Analyst1
Area: 273   0.76%

2.

"I think this is pretty nice for big packages, like the sizing of it. If this would deliver like a water bottle and a bag of chips, I think it would be a bit overkill."

Code: ● Reflection on size   Weight score: 0
G1, 472 - 472
Created: 29-08-2024 16:35 by Analyst1, Modified: 29-08-2024 16:35 by Analyst1
Area: 166   0.46%

3.

"I think the use cases are already different, right? Because we are thinking about snacks and Amazon was thinking about big packages."

Code: ● Reflection on size   Weight score: 0
G1, 512 - 512
Created: 29-08-2024 16:37 by Analyst1, Modified: 29-08-2024 16:37 by Analyst1
Area: 132   0.37%

4.

"It's bigger than we expected.

P1 1:13:44
It feels like a man standing inside the hood."

Code: ● Reflection on size   Weight score: 0
G2, 688 - 690
Created: 29-08-2024 16:55 by Analyst1, Modified: 29-08-2024 16:55 by Analyst1
Area: 88   0.20%

5.

"Maybe its good for the residential deliveries of huge packages. Something like stuff, I don't bother. But if it is like within the residential parks and you're delivering it within the public space, I don't prefer."

Code: ● Reflection on size   Weight score: 0
G2, 700 - 700
Created: 29-08-2024 16:57 by Analyst1, Modified: 29-08-2024 16:57 by Analyst1
Area: 214   0.49%

6.

"I'm imagining that delivering some food or something in a park in a given scenario, I don't want the drone to be some kind of mechanical robot."

Code: ● Reflection on size   Weight score: 0
G2, 704 - 704
Created: 29-08-2024 16:58 by Analyst1, Modified: 29-08-2024 16:58 by Analyst1
Area: 143   0.33%

7.

"it goes against the whole point of delivery then. Like when you order a delivery, you expect it to come to you. But because of the size and everything. But what really stood out for me which didn't work was how it was delivered. That yeah, it's just rough. And I've seen some

videos on Amazon warehouses and yes, they're the delivery like there the movement of packages is also not very delicate. It is like this, but I don't know it's just different to see it happened to the end user. That doesn't work."

Code: ● Reflection on size   Weight score: 0
G3, 804 - 804
Created: 29-08-2024 17:20 by Analyst1, Modified: 29-08-2024 17:20 by Analyst1
Area: 505   0.78%

8.

"Okay, it's quite big."

Code: ● Reflection on size   Weight score: 0
G4, 796 - 796
Created: 29-08-2024 17:35 by Analyst1, Modified: 29-08-2024 17:35 by Analyst1
Area: 21   0.03%

11. Safety concerns with dead drop

1.

"They just throw the package. They don't even like softly landed. What if there is a glass. What the hell? That's really high [P3 repeats]."

Code: ● Safety concerns with dead drop Weight score: 0
G1, 442 - 442
Created: 29-08-2024 16:26 by Analyst1, Modified: 29-08-2024 16:26 by Analyst1
Area: 138 0.38%

2.

"I think the drone should be able to go down instead of just dropping things. What if there is an animal under then the animal already dies."

Code: ● Safety concerns with dead drop Weight score: 0
G1, 450 - 450
Created: 29-08-2024 16:27 by Analyst1, Modified: 29-08-2024 16:27 by Analyst1
Area: 139 0.39%

3.

"t think it is a good solution to like, drop off a package from the distance, especially like a lot. Right now, it is liquid in a plastic box. What if it is glass, and then the package requirements should be really high to be able to drop from the distance. I don't think it's good."

Code: ● Safety concerns with dead drop Weight score: 0
G1, 454 - 454
Created: 29-08-2024 16:27 by Analyst1, Modified: 29-08-2024 16:27 by Analyst1
Area: 281 0.78%

4.

"If something is really sensitive objects inside the box I ordered, and if they are dropping from that height, if it is food, no (I dont like it)."

Code: ● Safety concerns with dead drop Weight score: 0
G2, 682 - 682
Created: 29-08-2024 16:54 by Analyst1, Modified: 29-08-2024 16:54 by Analyst1
Area: 145 0.33%

5.

"There could also be packages that are fragile."

Code: ● Safety concerns with dead drop Weight score: 0
G3, 788 - 788
Created: 29-08-2024 17:18 by Analyst1, Modified: 29-08-2024 17:18 by Analyst1
Area: 46 0.07%

6.

"I guess the way that it drops things, we probably want it to be more delicate. Because there's food and there's like things that you don't want to be scrambled."

Code: ● Safety concerns with dead drop Weight score: 0
G3, 796 - 796
Created: 29-08-2024 17:18 by Analyst1, Modified: 29-08-2024 17:18 by Analyst1
Area: 160 0.25%

7.

"With that size, it makes to be inside and to not drop somewhere.

P3 1:29:12
Else the package breaks."

Code: ● Safety concerns with dead drop Weight score: 0
G3, 870 - 872
Created: 29-08-2024 17:25 by Analyst1, Modified: 29-08-2024 17:25 by Analyst1
Area: 102 0.16%

8.

"The whole dropping thing looks quite brutal.

P2 1:22:42

Yeah. I mean, you order glass bottles"

Code: ● Safety concerns with dead drop   Weight score: 0
G4, 806 - 808
Created: 29-08-2024 17:37 by Analyst1, Modified: 29-08-2024 17:37 by Analyst1
Area: 95   0.14%
