# Supplementary material for: Human interactions with delivery drones in public spaces: design recommendations from recipient and bystander perspectives
Source: Front Robot AI. 2025 May 30;12:1580289. doi: 10.3389/frobt.2025.1580289 (PMC12162322; doi:10.3389/frobt.2025.1580289)
Supplement: Supplementary file 1 [file DataSheet1.zip › Data_&_results/Focus_groups/Storyboards_sketches/FG1.pdf]

Storyboard an interaction

Group no: 1

Role: Recipient

1.

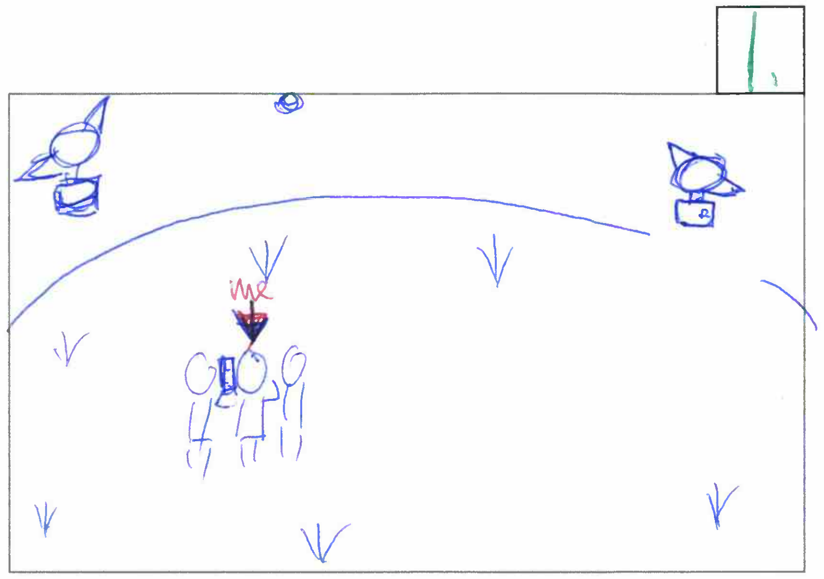

The order via phone, delivering by a drone, estimated EAT is 30 mins later, so you keep chat with friends while waiting.

2.

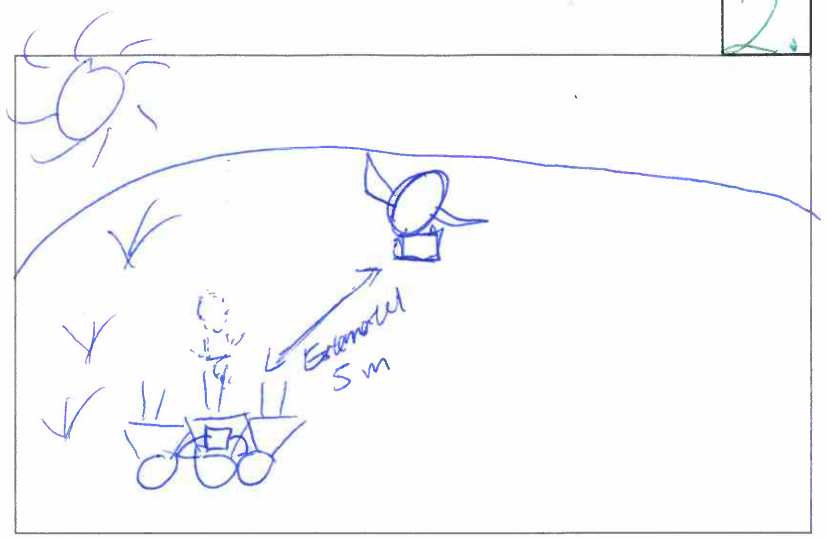

Receiving the package: get notification when the drone is 5m-10m away from you, Then you can press "Ready to receive" button on your phone.

3.

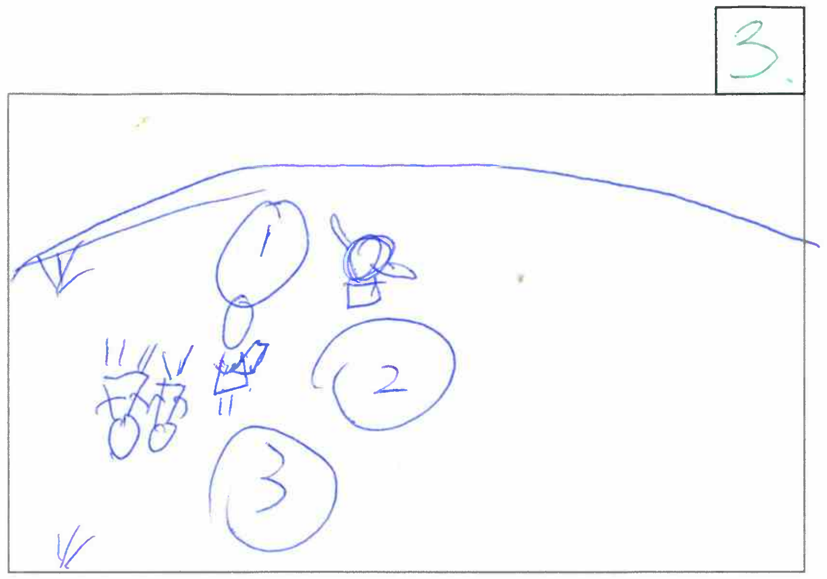

After press "Ready" button, the drone will give a few options for landing zones. then you choose which zone or any other place to land. finally, you choose zon[illegible] 2 to land.

4

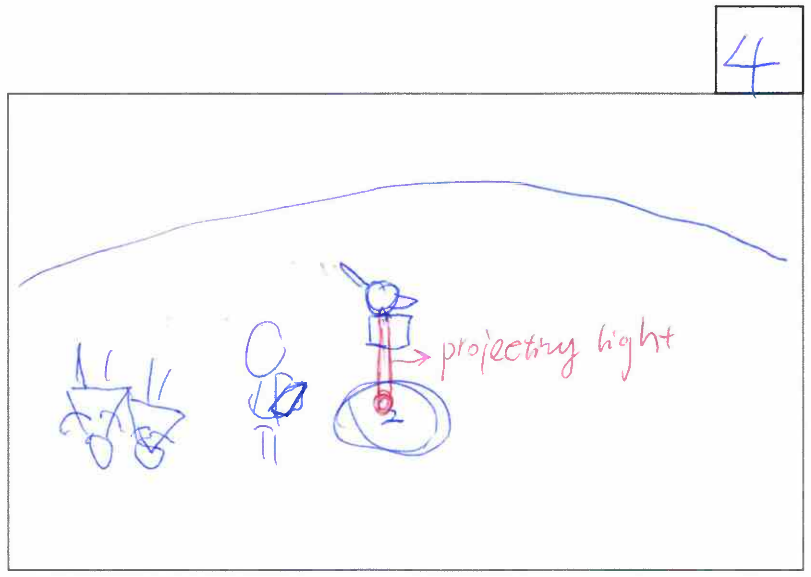

The drone is flying above the zone2. and the drone projects light on the ground for indicating the landing spot. The recipient should be able to terminate the landing process any time. The drone is going down...

5.

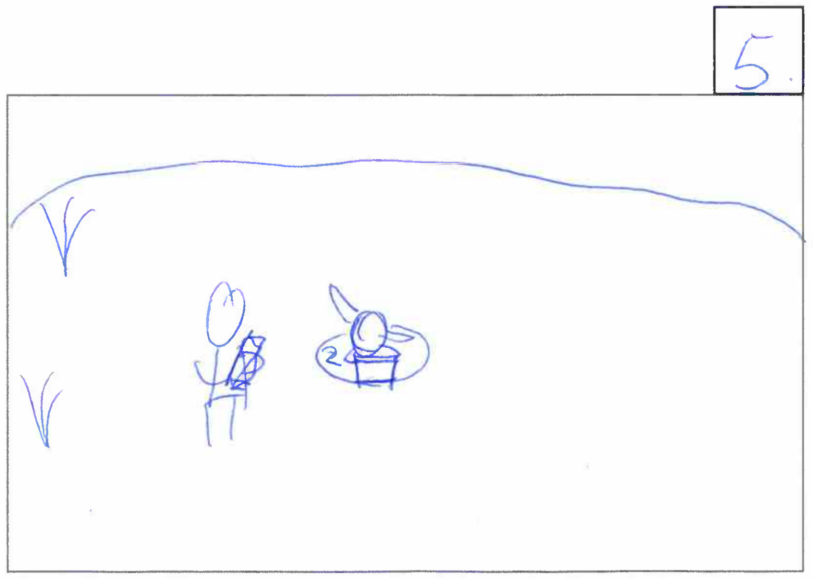

The drone dropped the package on the spot. and you can confirm you have received package The drone has to wait above the spot. till you pressed the confirm button.

6

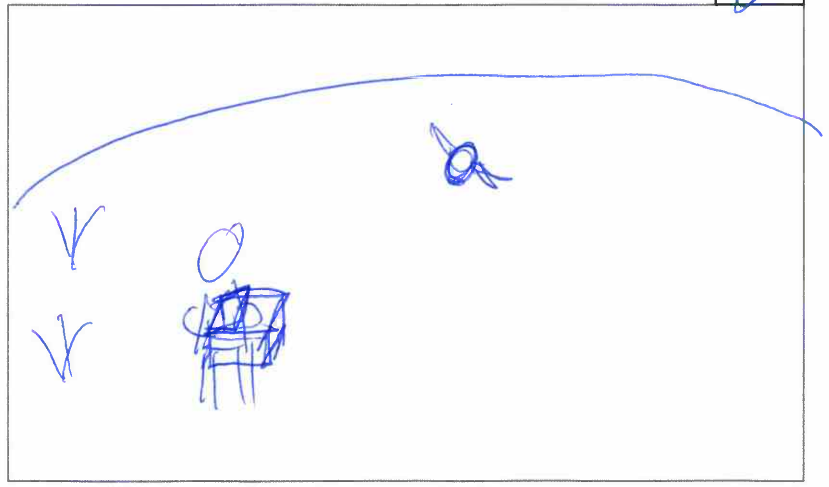

The drone is flying away.

1

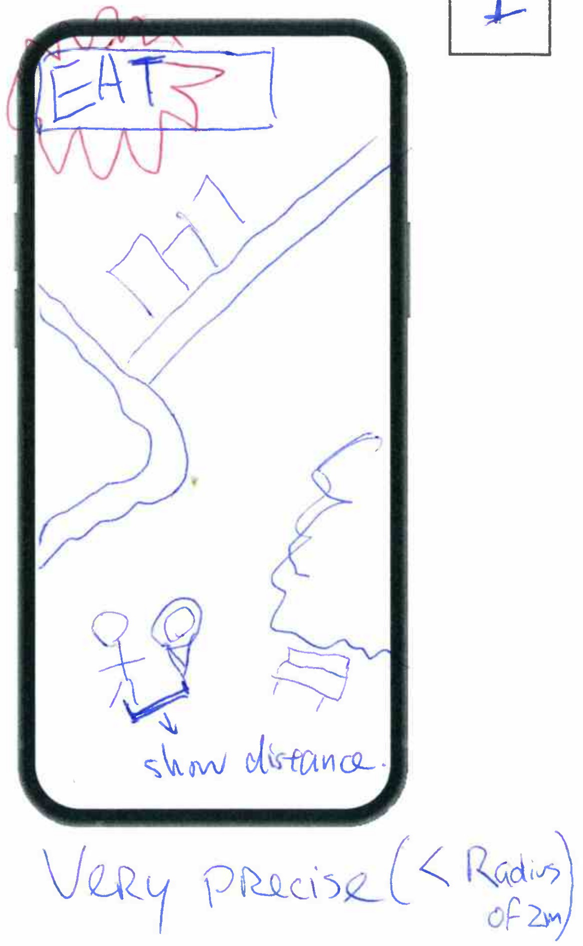
EAT
show distance.
Very precise (< Radius of 2m)

2

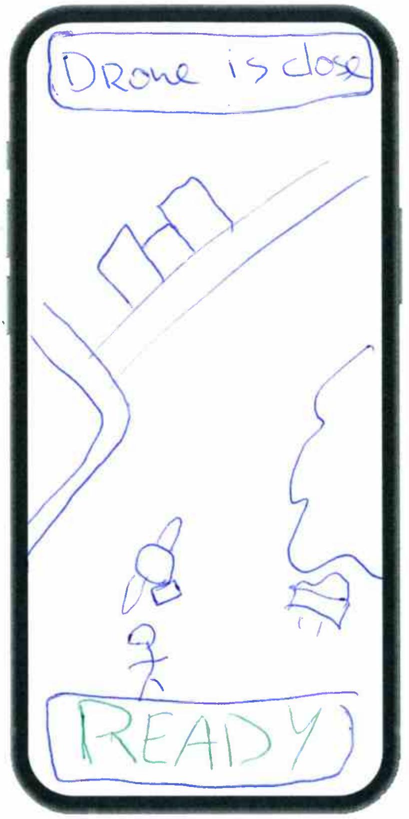
Drone is close
READY

3

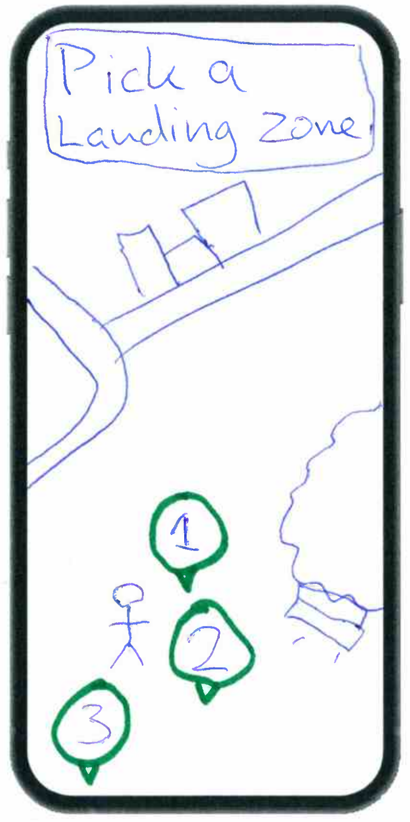
Pick a Landing zone
1
2
3

4

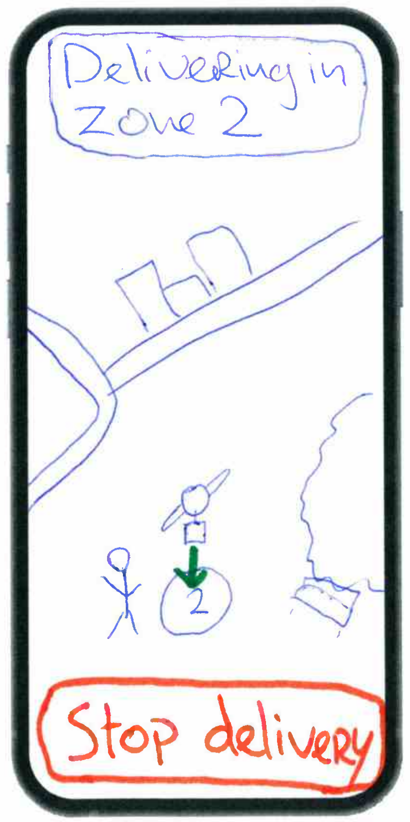
Delivering in zone 2
2
Stop delivery

5

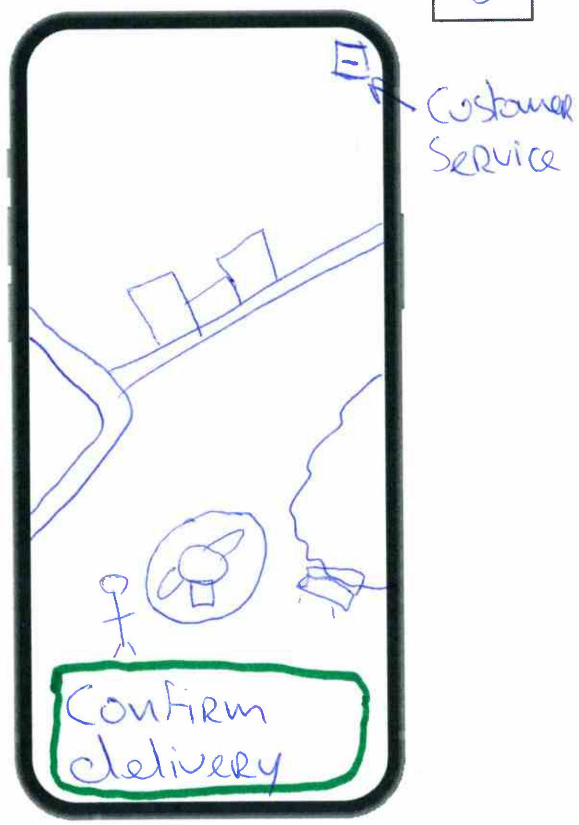
Customer Service
Confirm delivery

Recipient ①

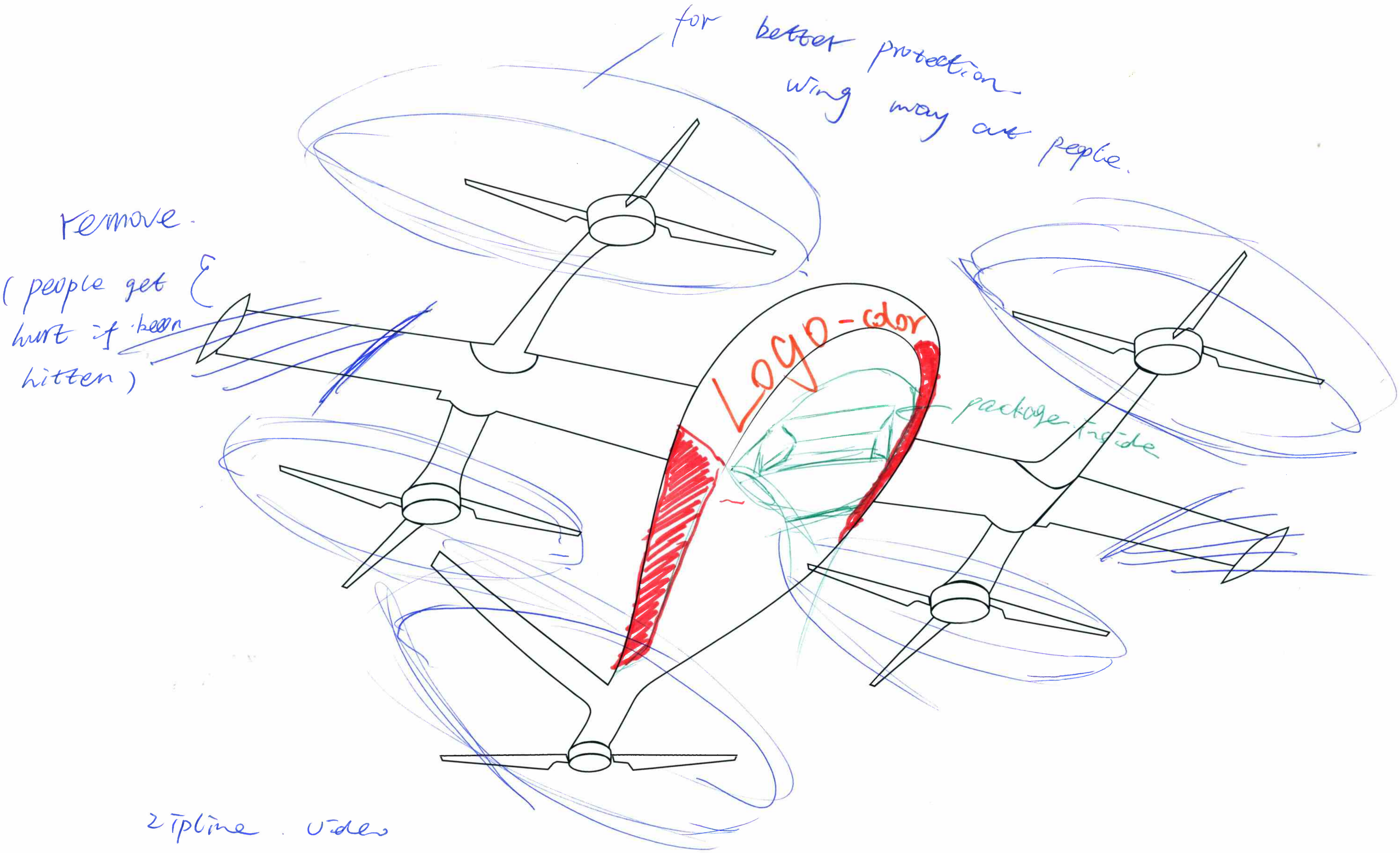

Zipline video

Logo in both main body and capsule

Storyboard an interaction

Group no: 1

Role: Bystander

1

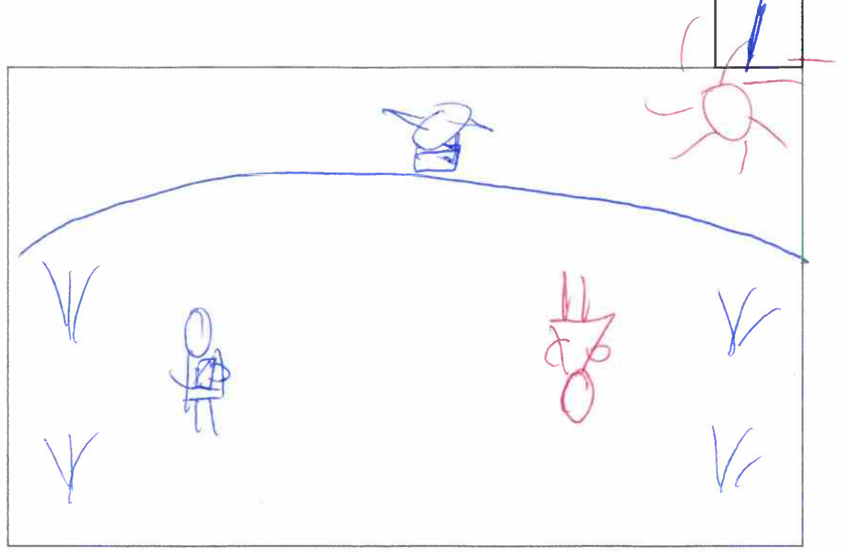

I see a drone coming ....

2.

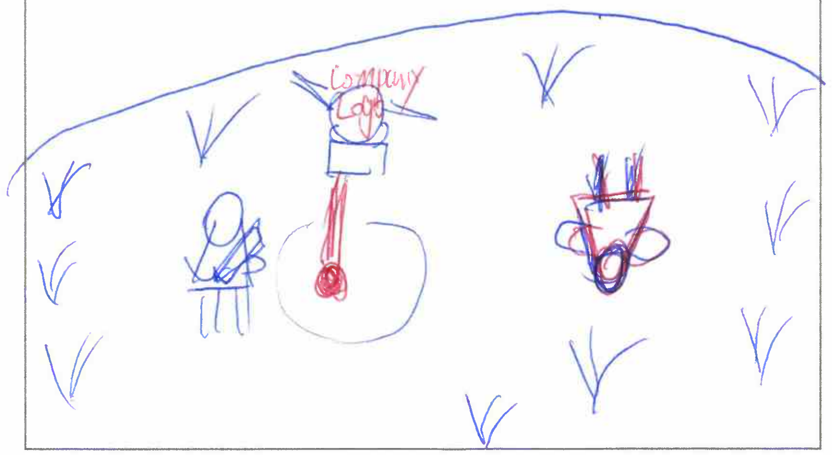

I can see where it will be land ....

3

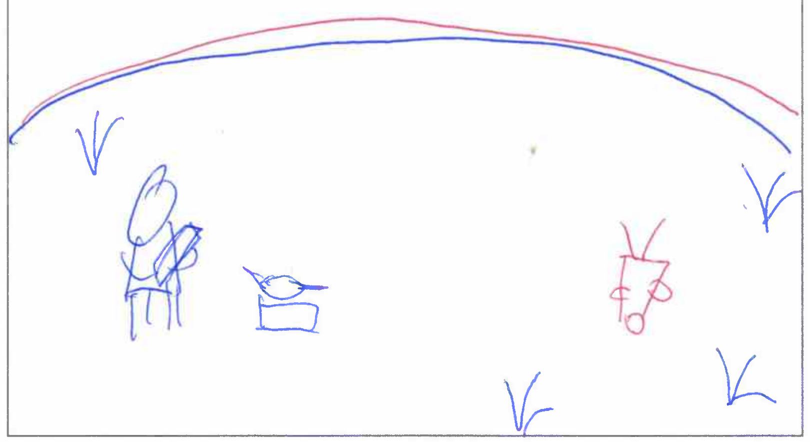

I notice the drone dropped the package

4

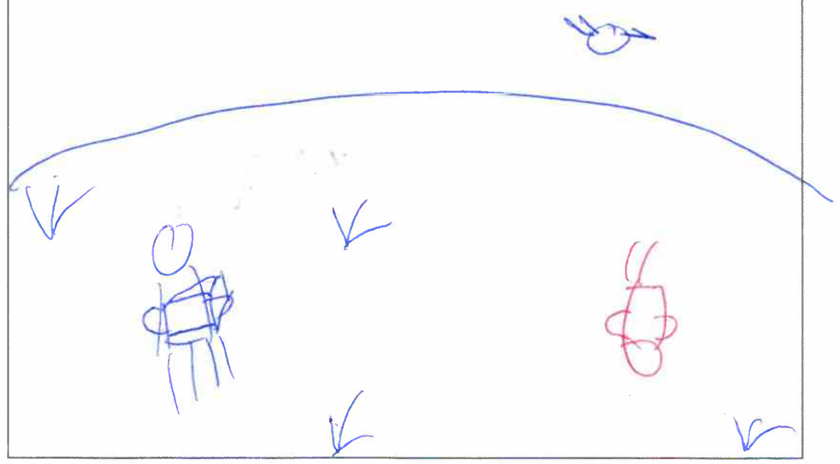

The drone is flying away....

Bystander (1)

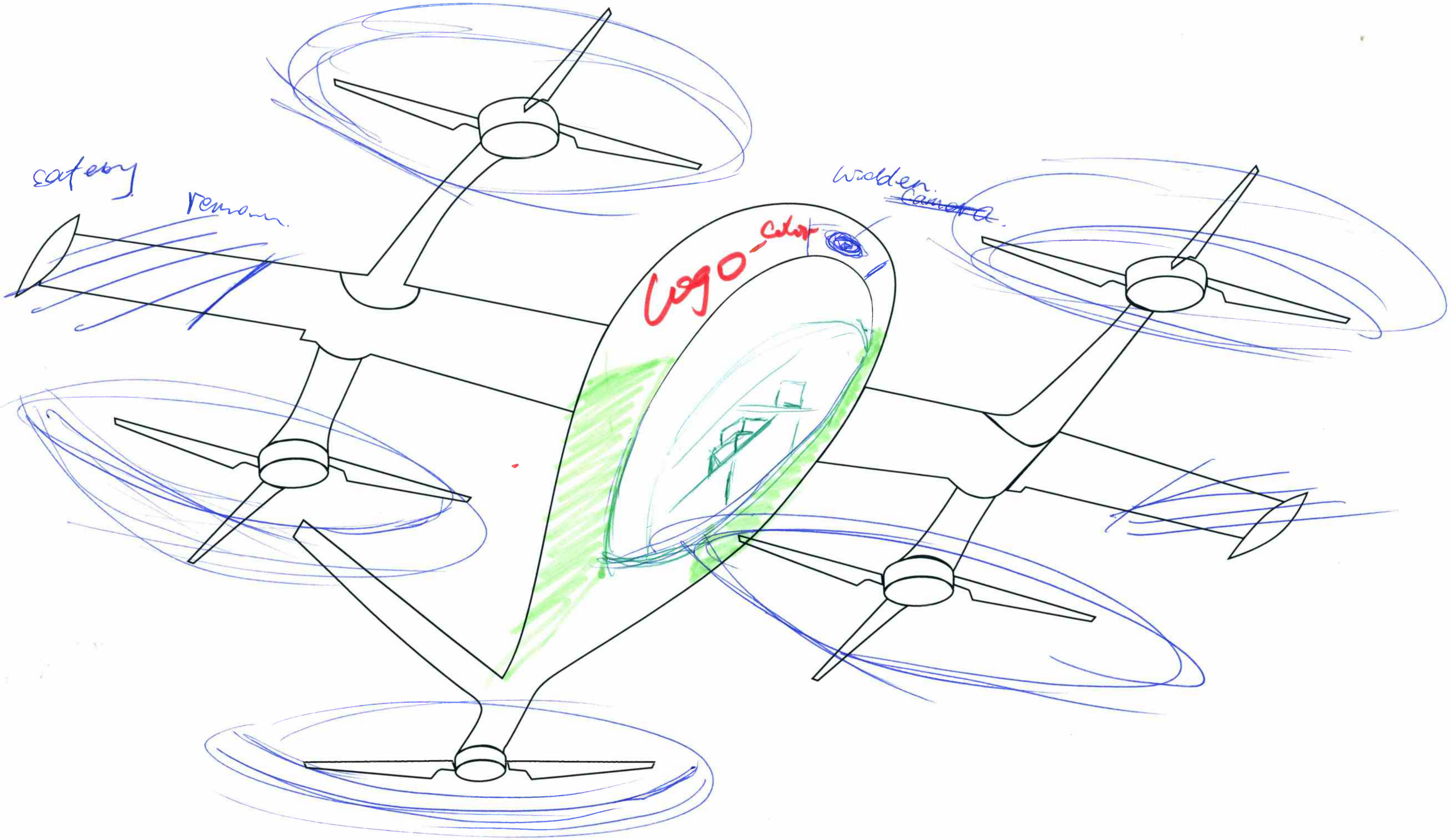

From zipline, the logo can be printed on the capsule x drone
