# Supplementary material for: Human interactions with delivery drones in public spaces: design recommendations from recipient and bystander perspectives
Source: Front Robot AI. 2025 May 30;12:1580289. doi: 10.3389/frobt.2025.1580289 (PMC12162322; doi:10.3389/frobt.2025.1580289)
Supplement: Supplementary file 1 [file DataSheet1.zip › Data_&_results/Focus_groups/Storyboards_sketches/FG2.pdf]

Storyboard an interaction

Group no: 2

Role: Recipient

1

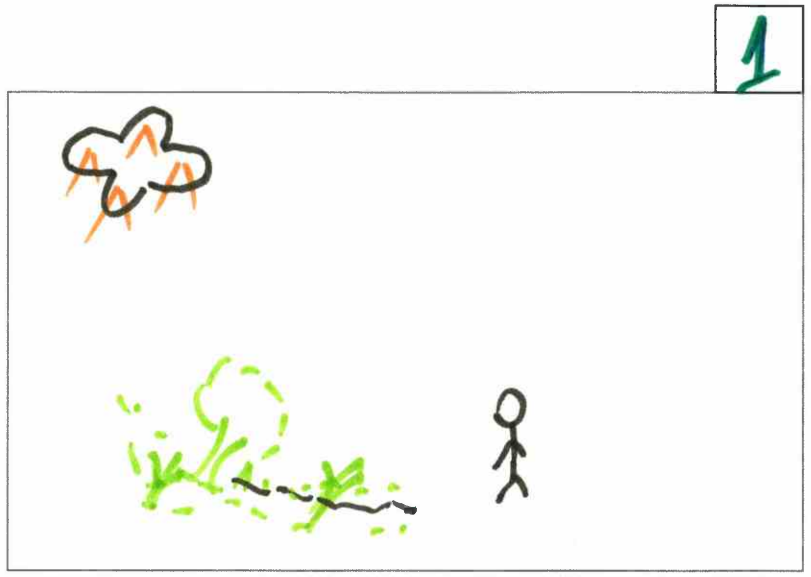

Colour identification from the drone.

2

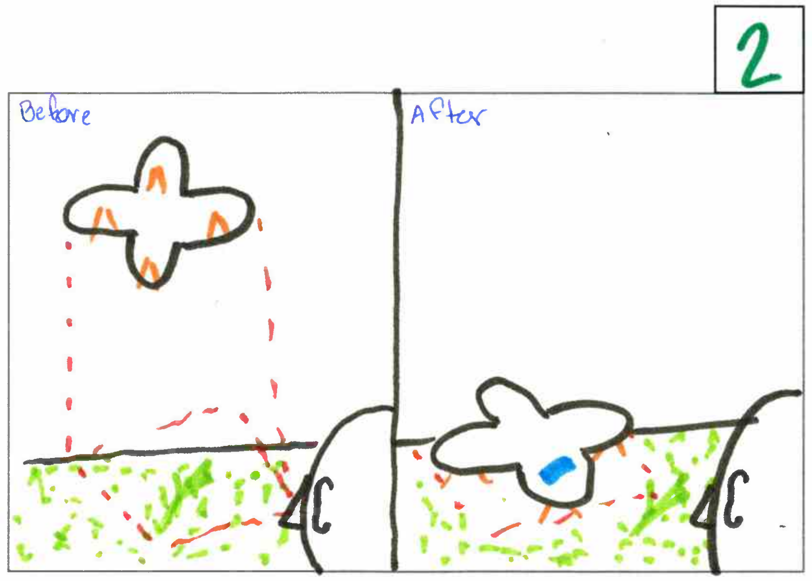

Landing marks on the graund. Laser lights.
You scan from your mobile the code.

3

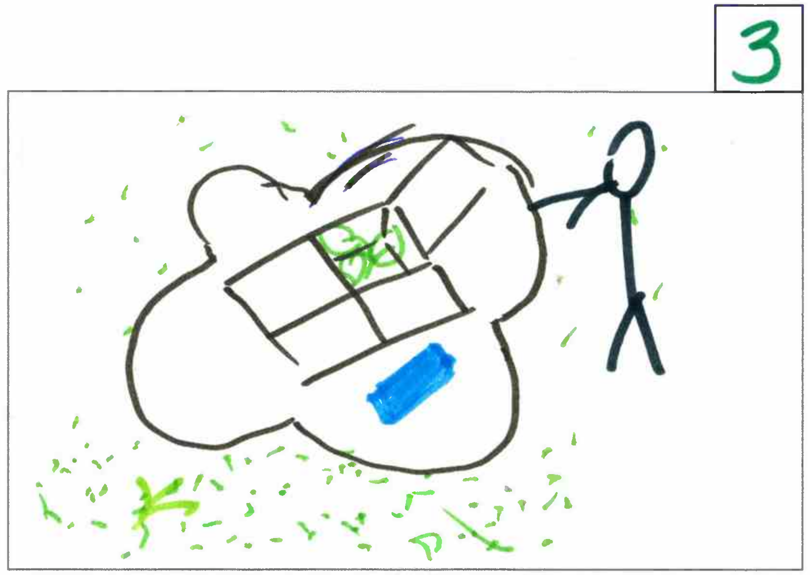

When you scan only your compartment opens.

4

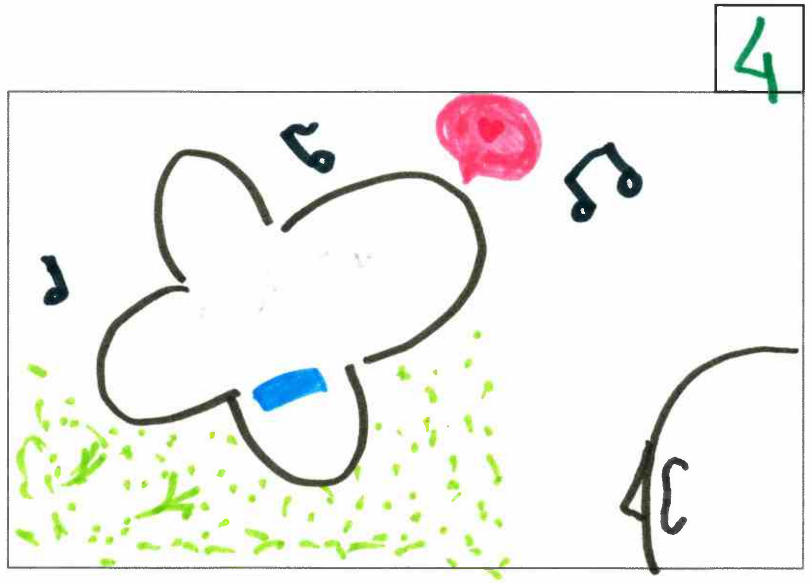

It plays a song + customized message (optional > "Happy Birthday!")

5

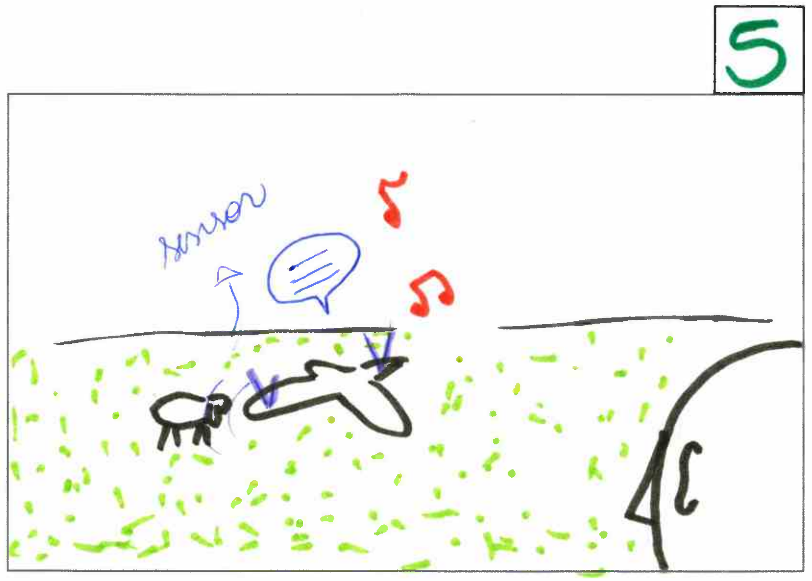

Warning song - an animal is around.
Confirmation > clear area on the phone.
Blink Blink (→ side of the drone), and it flyes away.

6

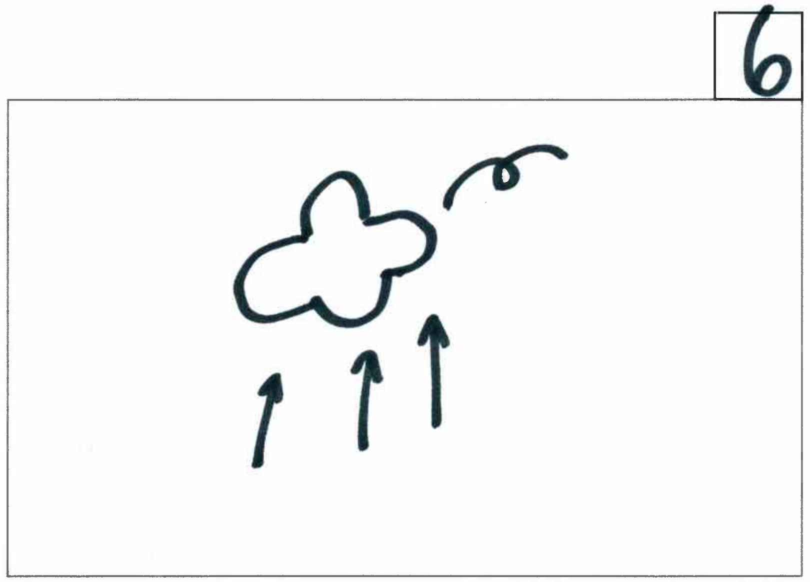

It flyes away.
Mobile message: "Delivered!"
Rating the delivery service.

6

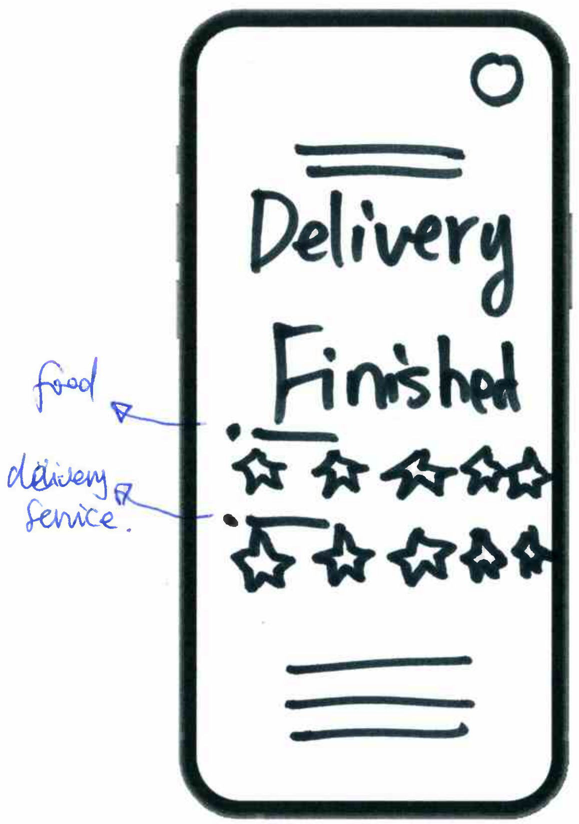
Delivery
Finished
food
delivery service

2

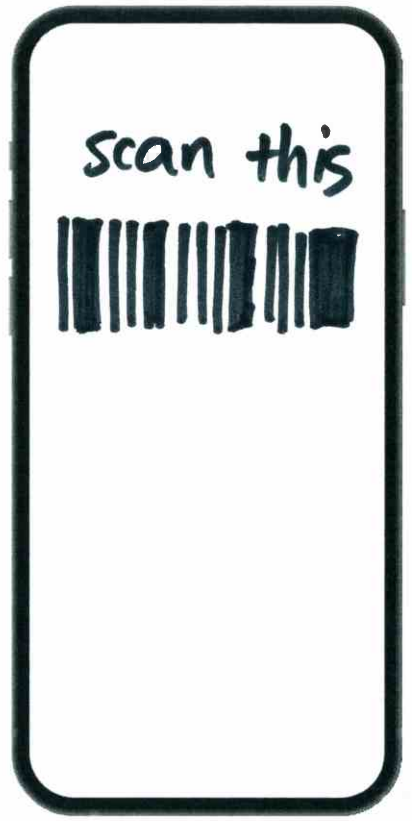
scan this

1

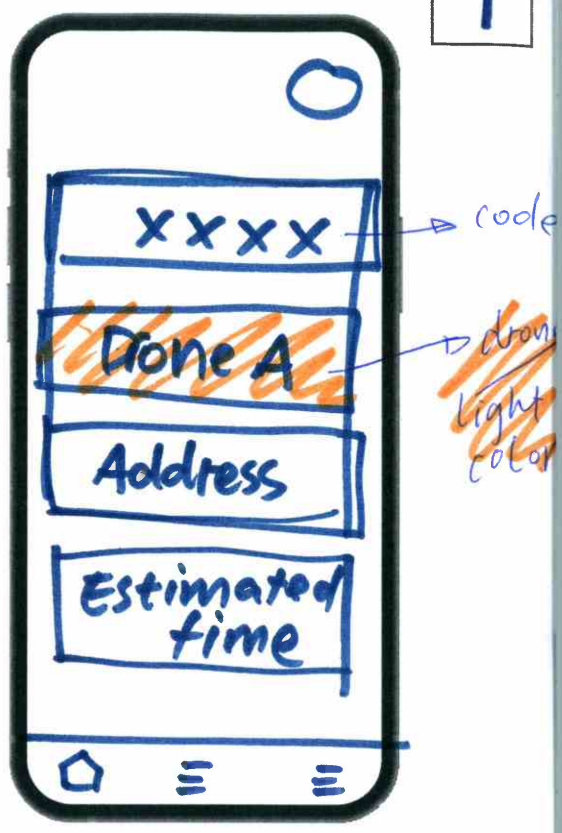
XXXX
code
Drone A
light
Address
Estimated time

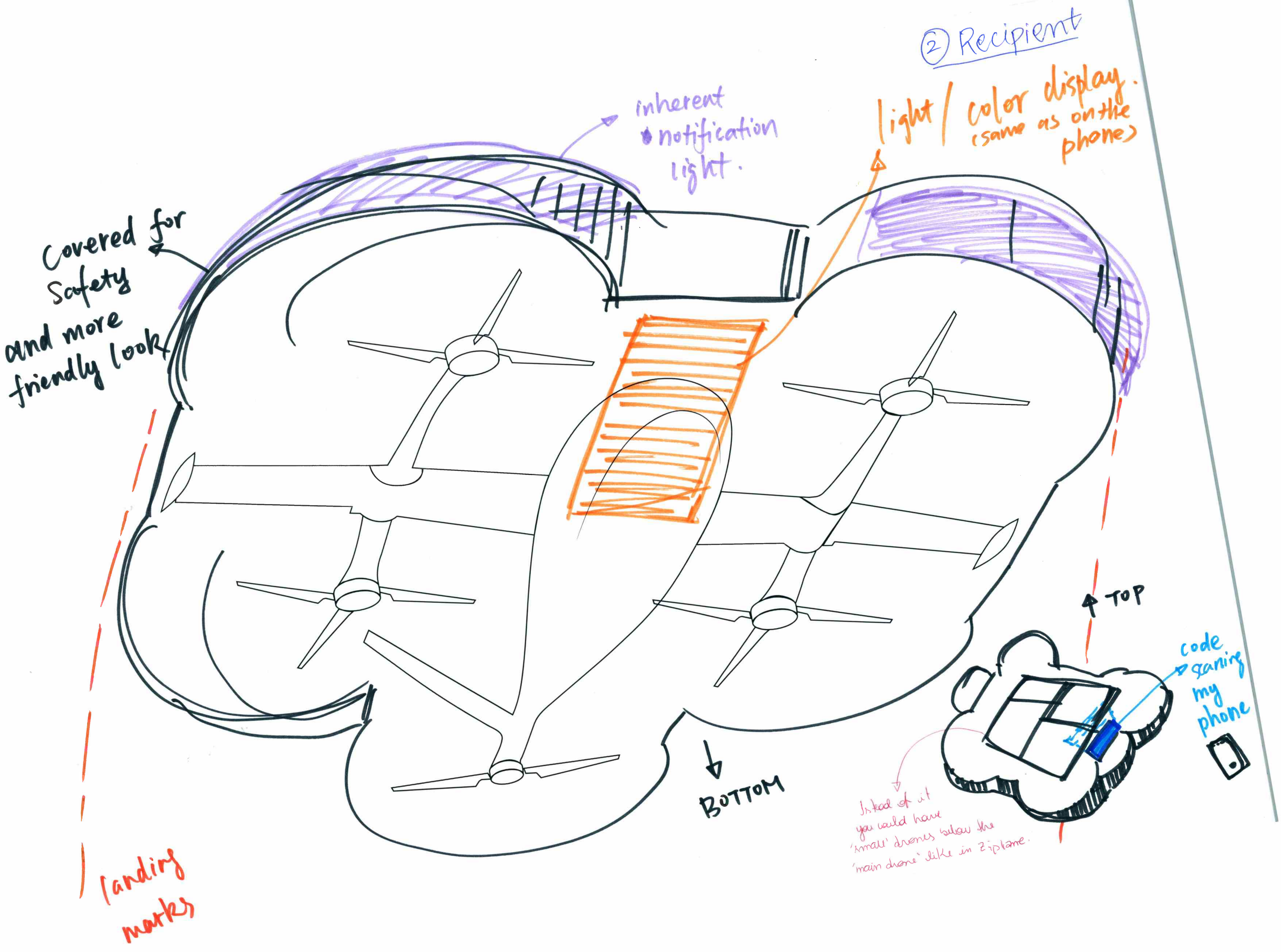
② Recipient
inherent notification light.
light / color display.
(same as on the phone)
Covered for Safety and more friendly look
TOP
BOTTOM
code scaning my phone
Instead of it you could have 'small' drones below the 'main drone' like in Ziplane.
landing marks

Storyboard an interaction

Group no: 2

Role: Bystander

1

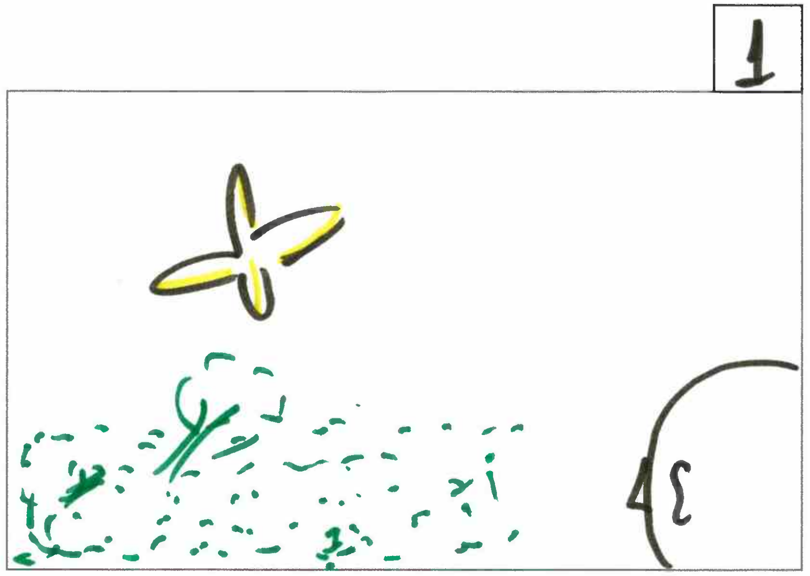

2

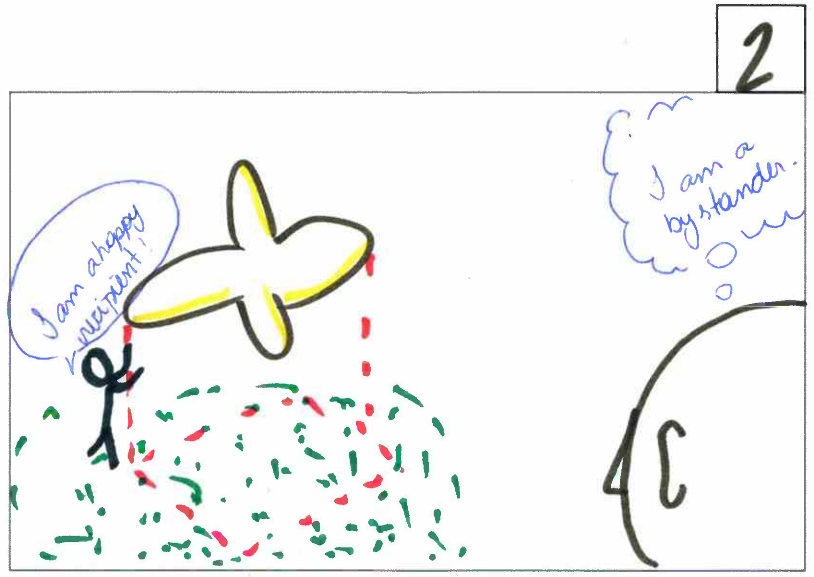

Landing marks on the ground.

3

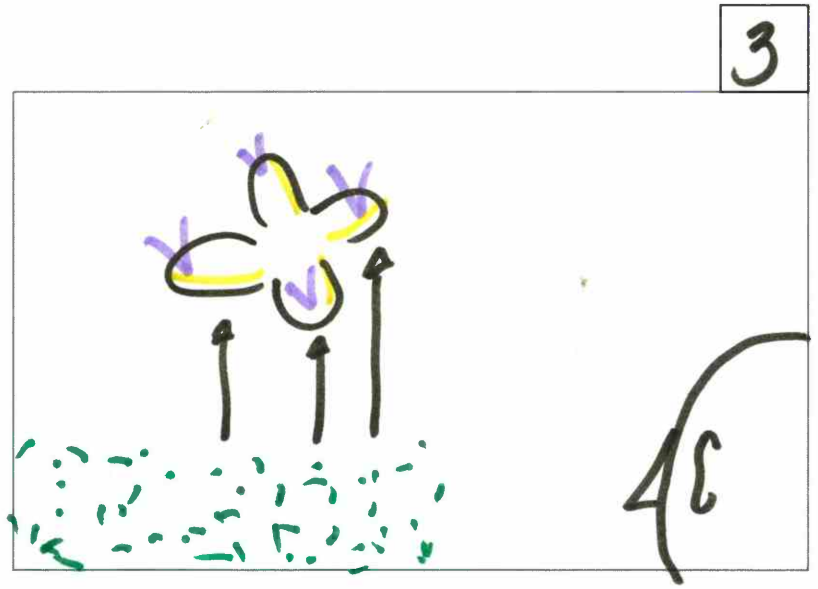

Blinking lights > flying away.

Just to make sure that my beloveds are safe (child, dog).

② Bystander

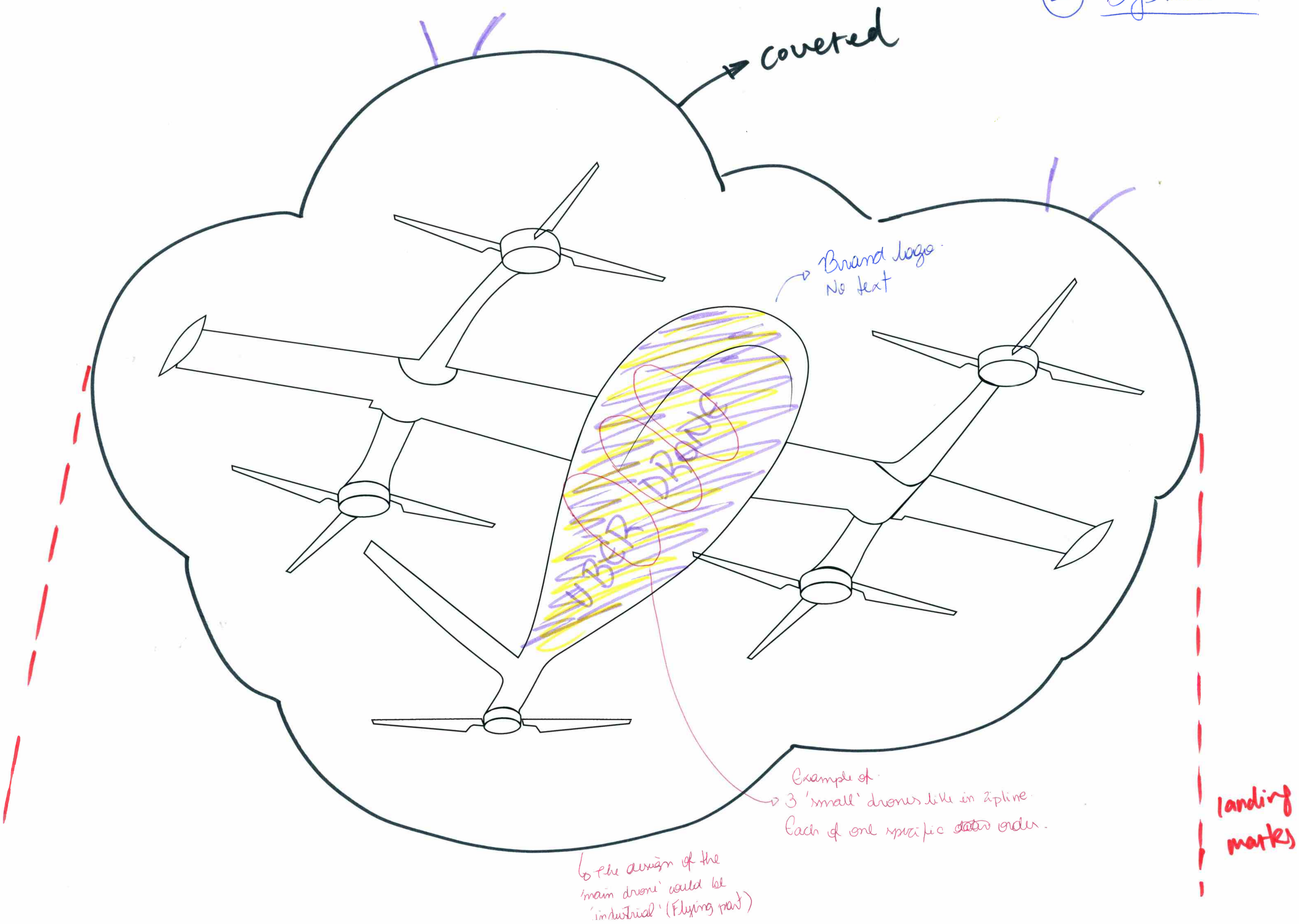
covered
Brand logo
No text
UBER DRONE
Example of
3 'small' drones like in zipline
Each of one specific order.
the design of the 'main drone' would be 'industrial' (Flying part)
landing marks
