# Supplementary material for: Human interactions with delivery drones in public spaces: design recommendations from recipient and bystander perspectives
Source: Front Robot AI. 2025 May 30;12:1580289. doi: 10.3389/frobt.2025.1580289 (PMC12162322; doi:10.3389/frobt.2025.1580289)

Storyboard an interaction

Group no: 3

Role: Recipient

1

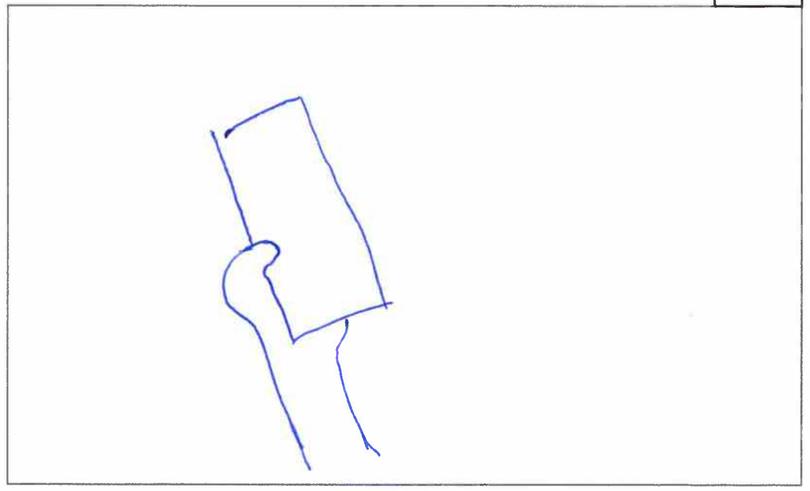

comfirming on the order.

2

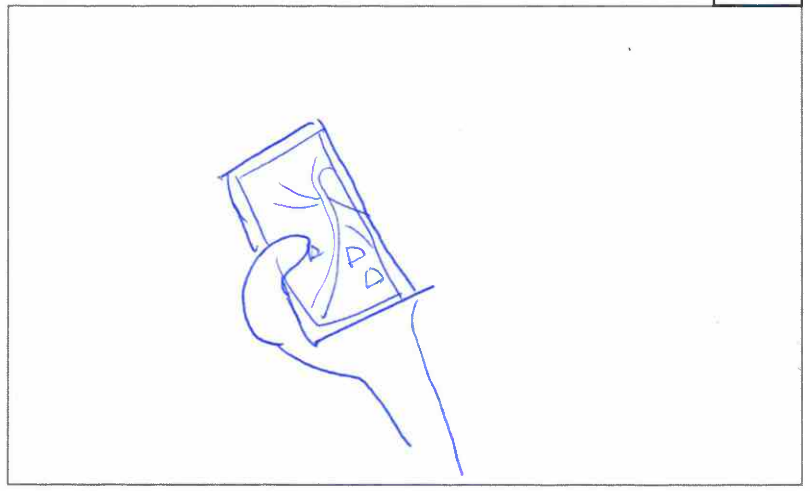

The drone left the warehouse, the apps tell us the delivery route, estimated time, visible range.

3

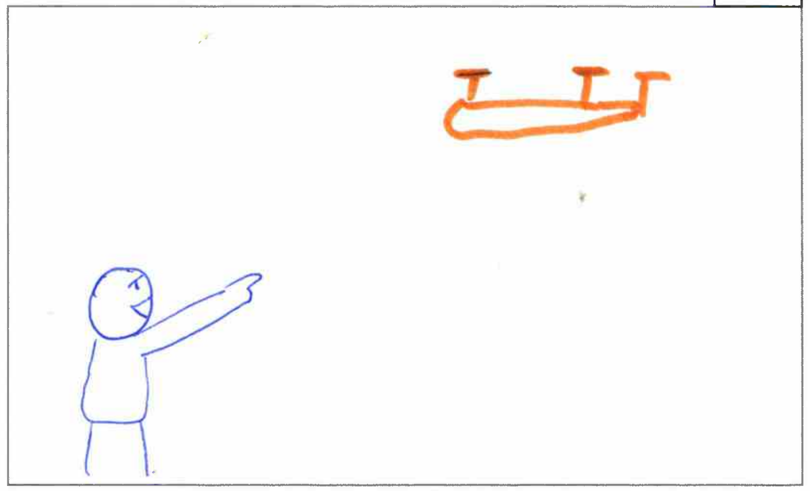

Identify drone purpose with colour code.

4

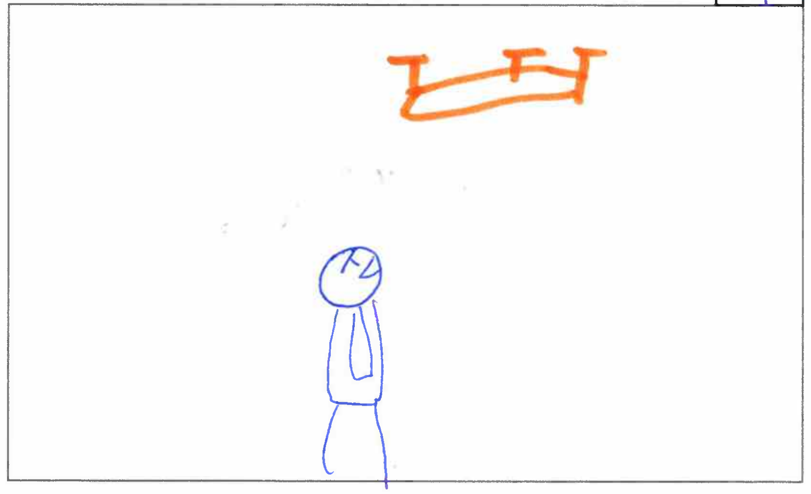

"The drone is ready to deliver, please confirm."

5

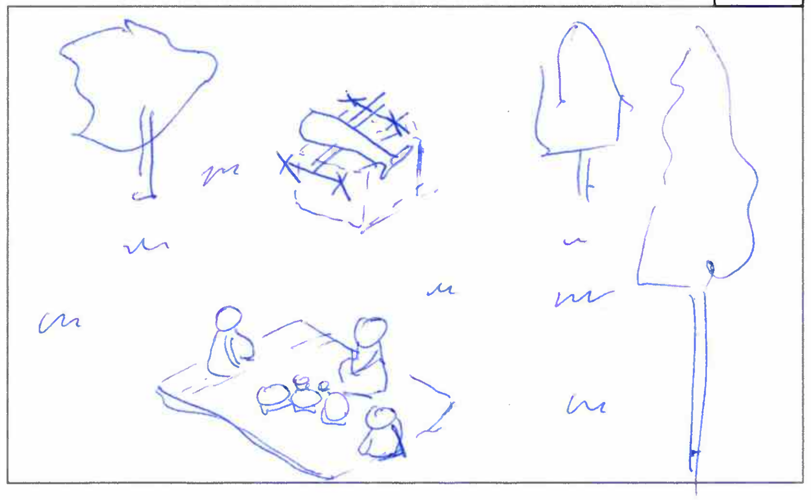

The drone hovers on our head and drops the goodies

16 or 36

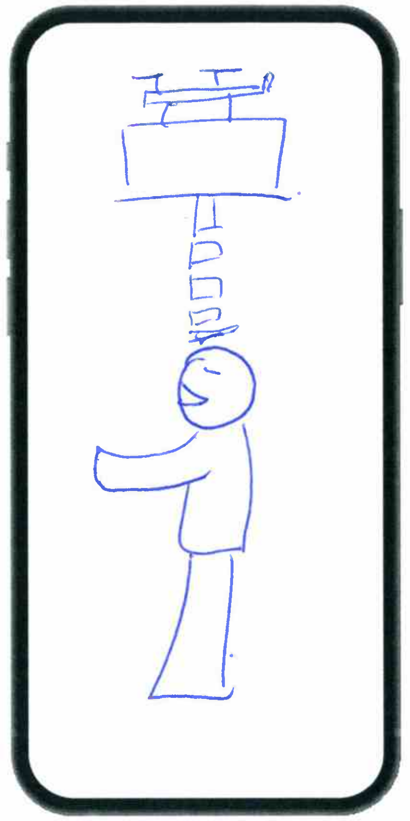

instructions of what will happen.

2-3

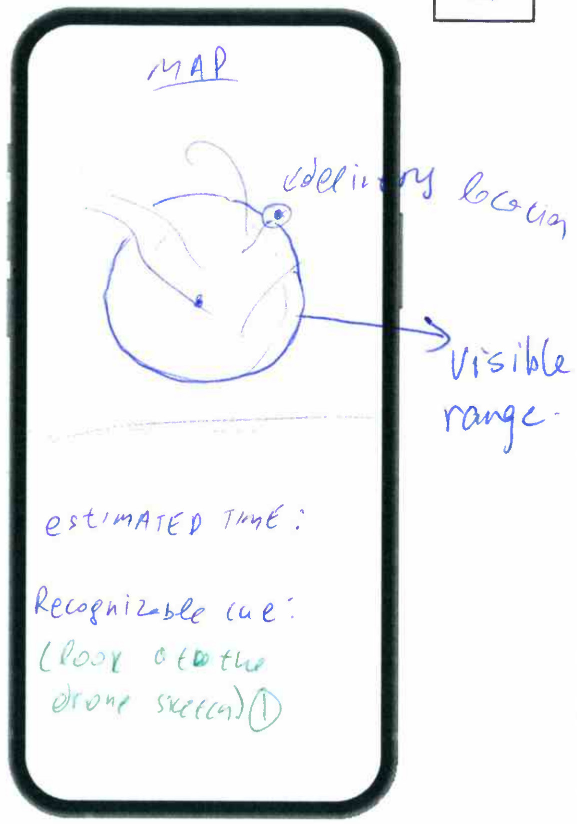

1

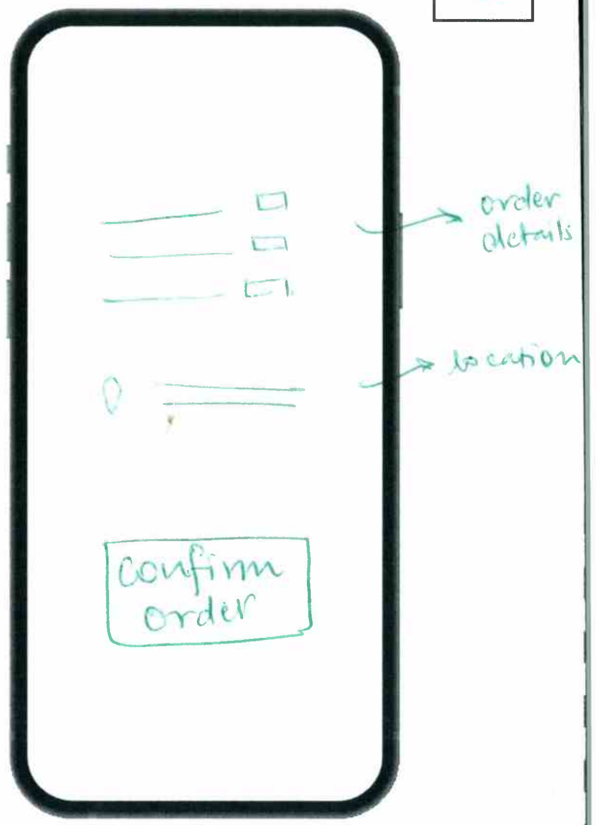

4-5

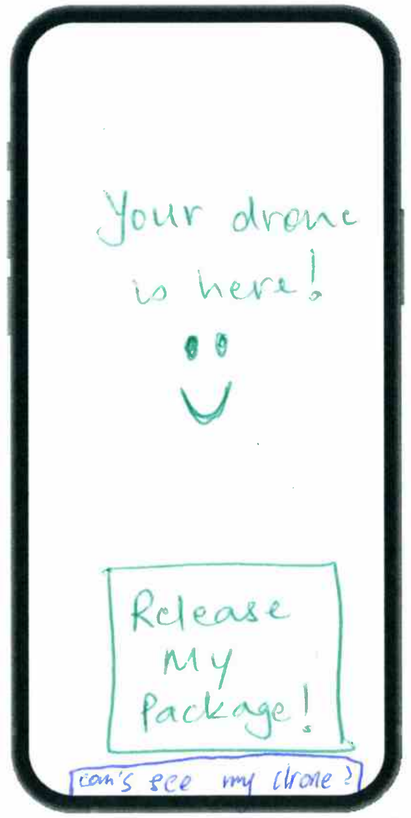

① Drone sketch (recipient)

③ Recipient

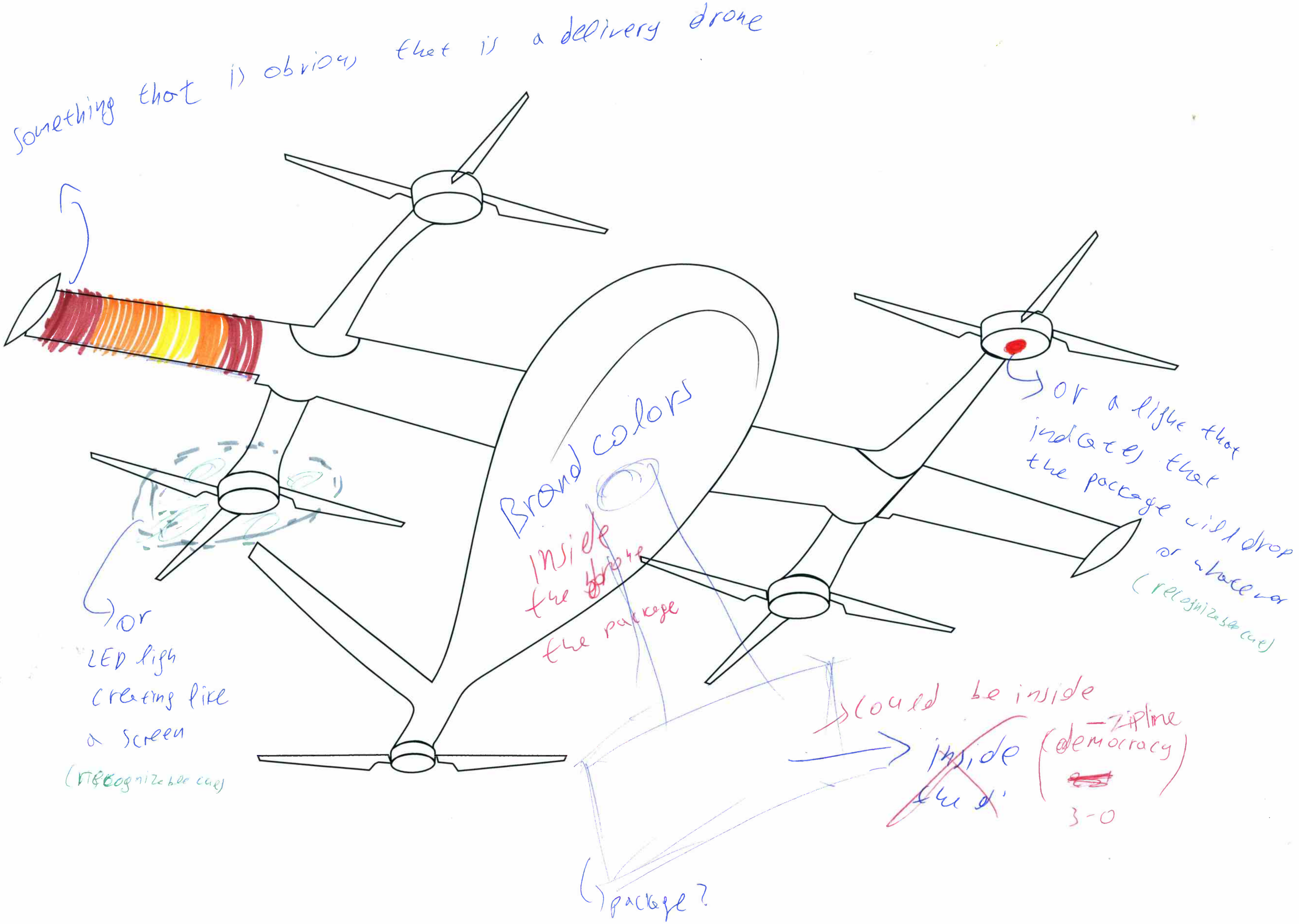

Storyboard an interaction

Group no: 3

Role: Bystander

1

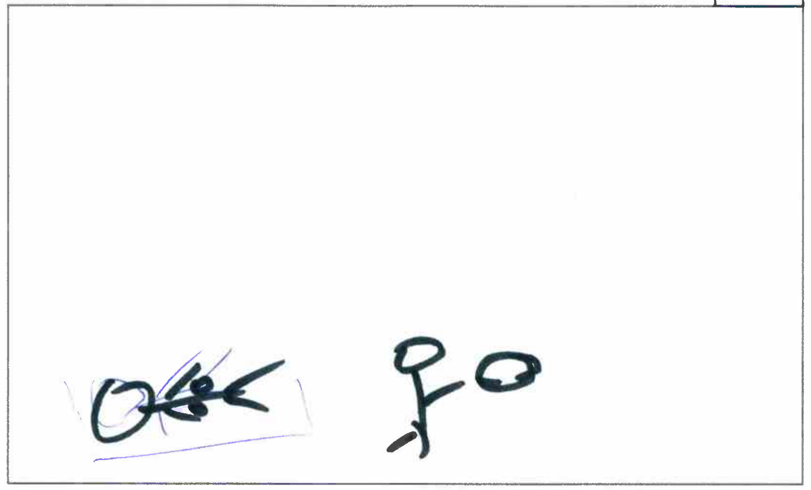

Chilling with friends
doing funny and relaxing
stuff.

2

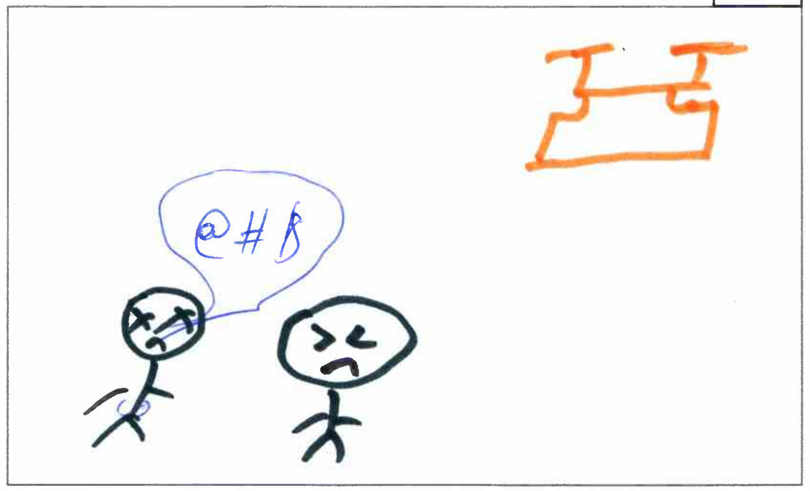

Annoying sound
drone approaching

3

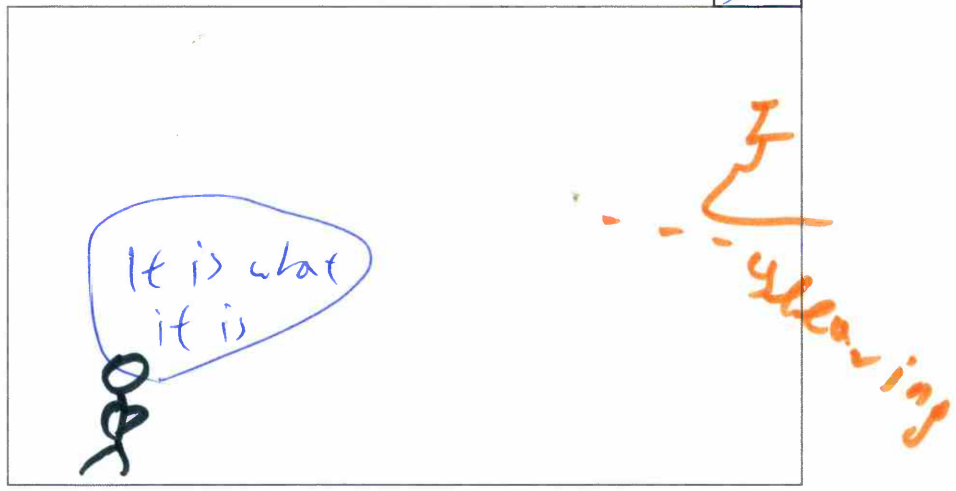

Identify if it is
a delivery drone?

4

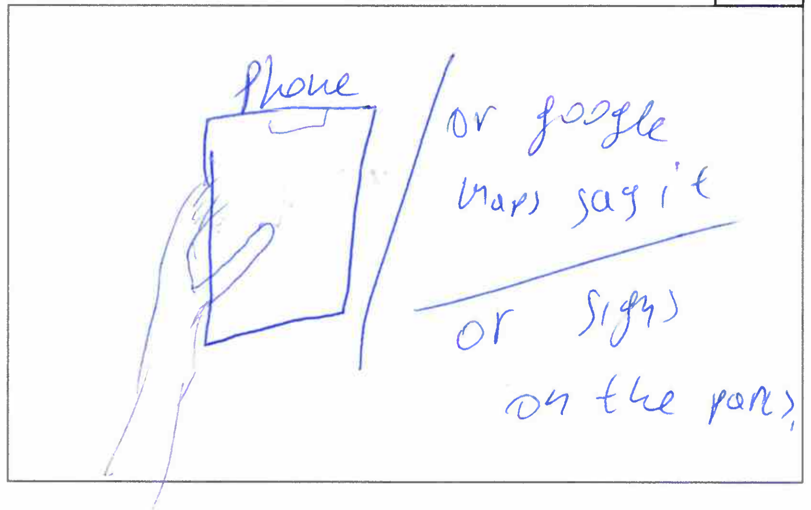

if it is really frequent
the deliveries
I want to find drone free
zones - areas

OR.

4

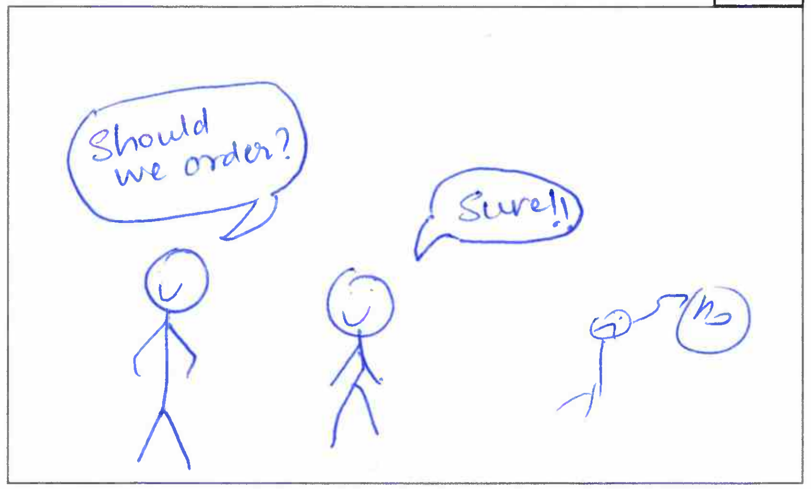

Bystanders get tempted into
ordering (eg. beers on a hot
summer afternoon).

③ Bystander

② Drone sketch (bystander)

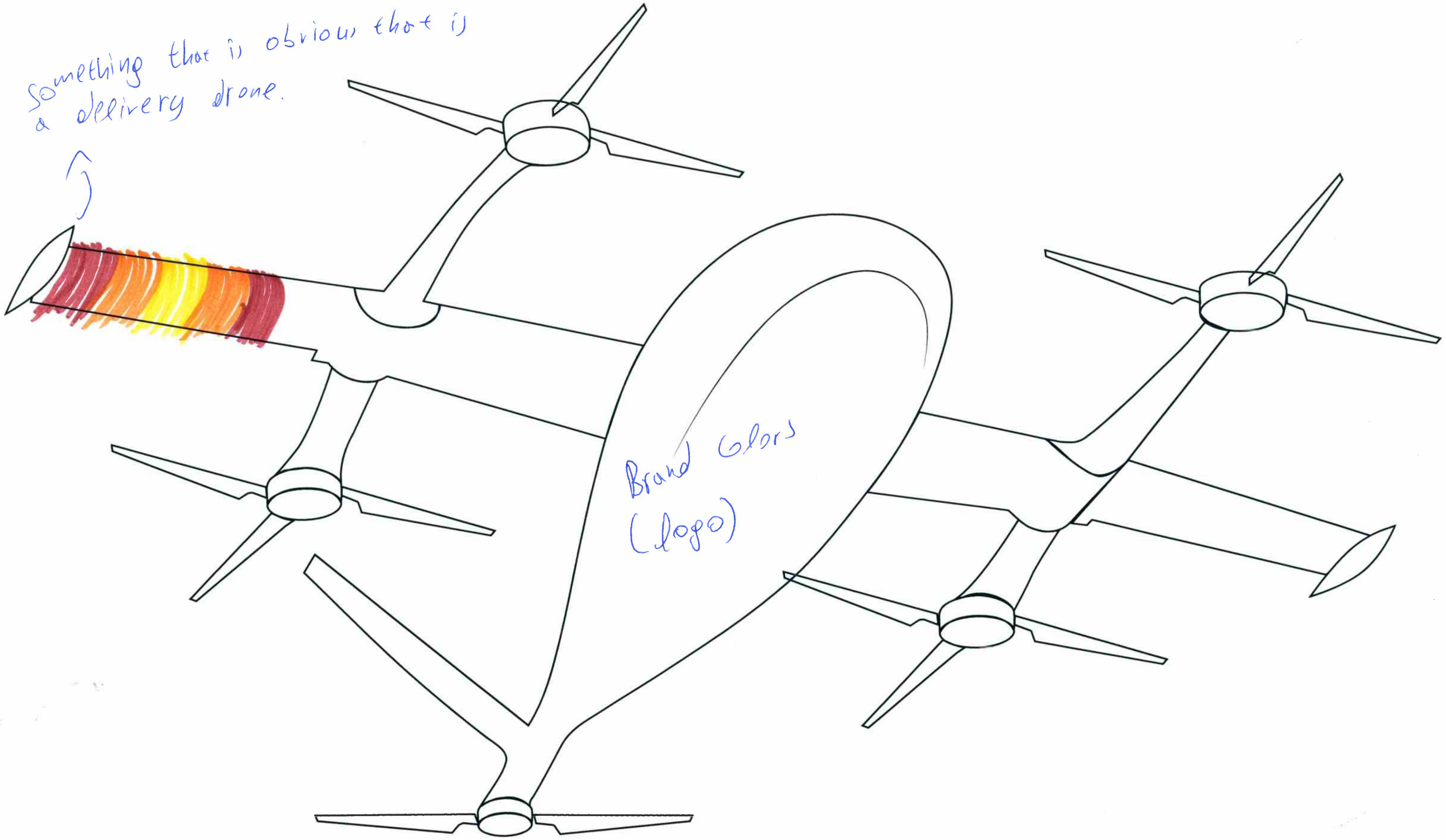

Supplement: Supplementary file 1 [file DataSheet1.zip › Data_&_results/Focus_groups/Storyboards_sketches/FG3.pdf]
