# Supplementary material for: Human interactions with delivery drones in public spaces: design recommendations from recipient and bystander perspectives
Source: Front Robot AI. 2025 May 30;12:1580289. doi: 10.3389/frobt.2025.1580289 (PMC12162322; doi:10.3389/frobt.2025.1580289)
Supplement: Supplementary file 1 [file DataSheet1.zip › Data_&_results/Focus_groups/Storyboards_sketches/FG4.pdf]

Storyboard an interaction

Group no: 4

Role: Recipient

ordering. **1**

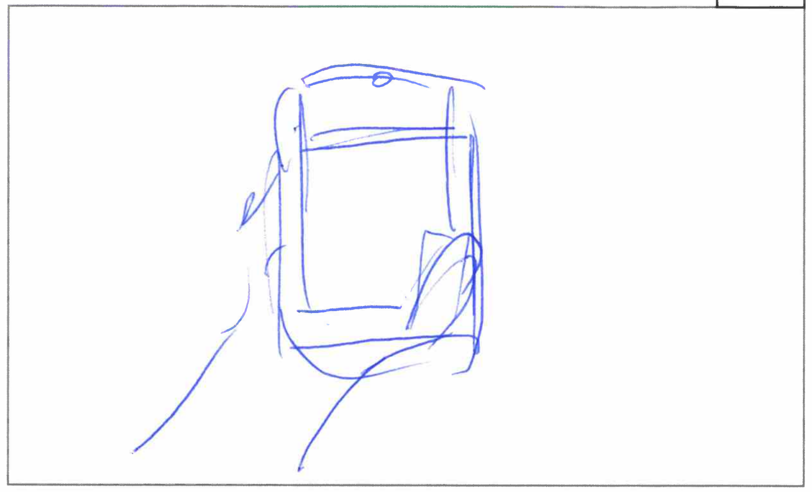

1. Open the app
2. Add food to cart (Discuss with friends)
3. Check Delivery time
4. Get the summary & wait

Wrong Drone ~~passing~~ nearby **2**

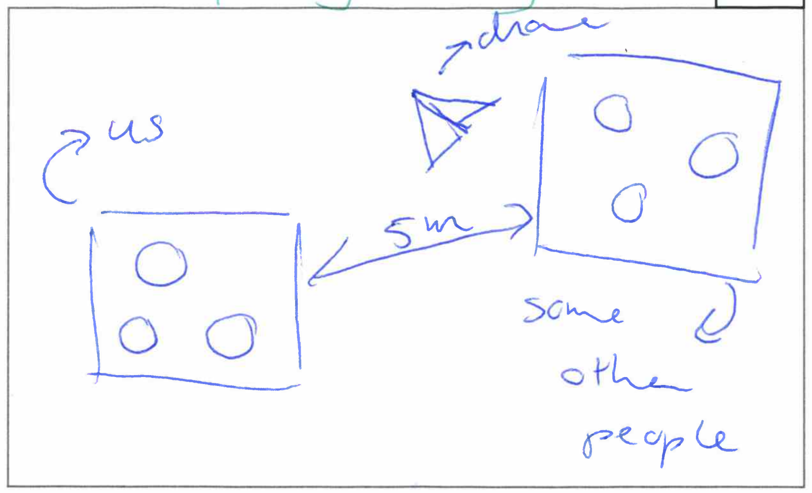

1. Drone ~~fly~~ hover nearby between 2 group
2. Drone project color code
3. The other group go check, we see the color and not going to pick

Right Drone Nearby **3**

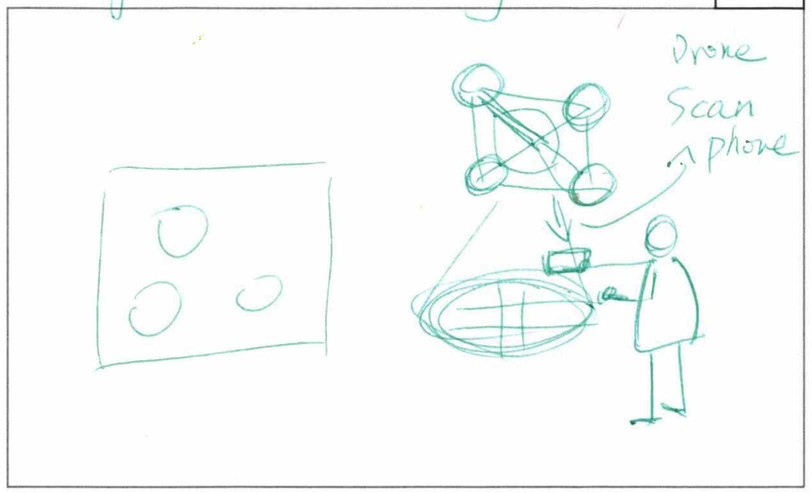

1. Drone hover nearby
2. Project Right color code
3. We go check, right order,
4. Confirm ~~in~~ on the app (Drone scan phone)

Drone land **4**

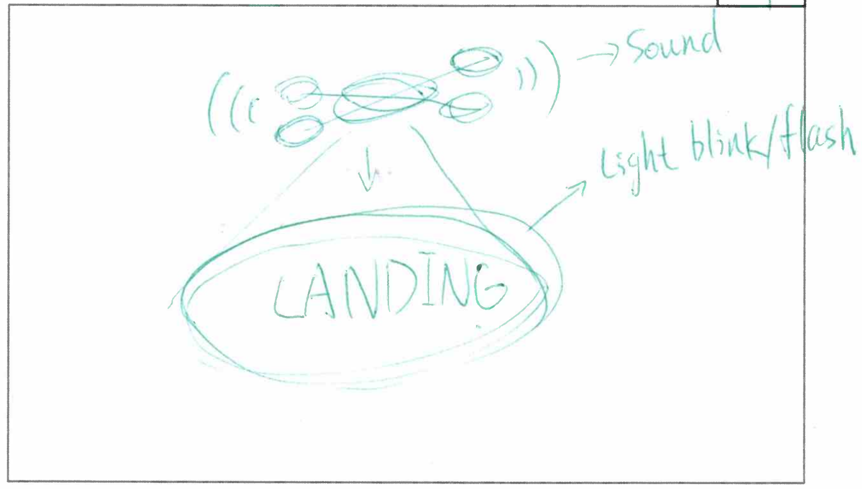

1. Project Landing area
2. Light blink, Sounds on
3. Land slow down on ground
4. Drone stops totally (Blade stops)

Get our food **5**

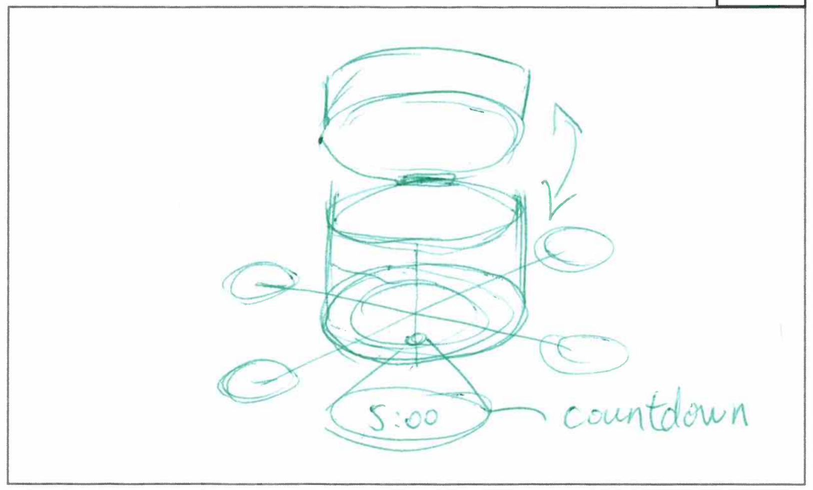

1. Drone open by itself, count down starts
2. Max. waiting time 5min. (After it, close, go away by itself)
3. I pick up the food (confirm on phone)

Drone leaving **6**

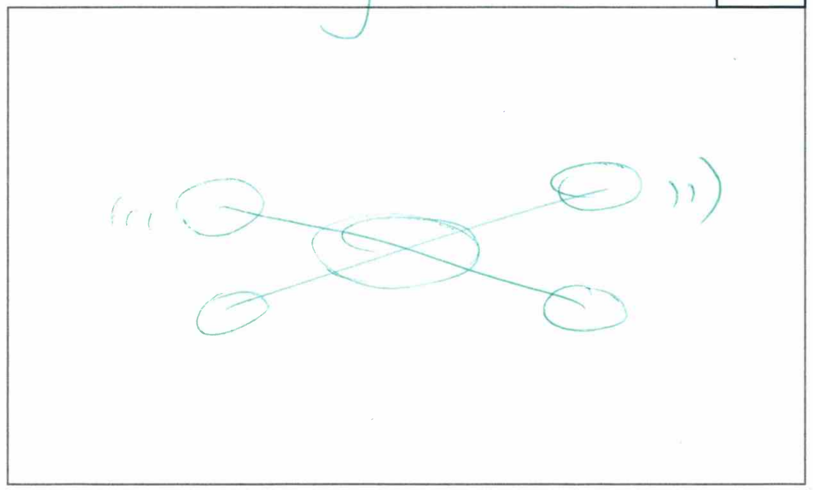

1. Sounds on first (Beep, Engine,)
2. ~~Blades on later~~ Self-scan around
3. If not ~~saft~~ safe, stay on ground (Recipient not responsible on drone leaving)
4. If safe, blade on, ~~fl~~ fly up slowly

1 A

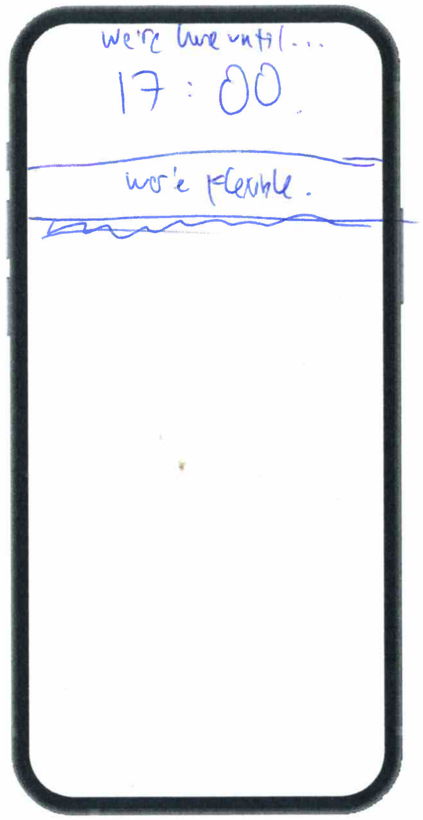
we're here until...
17 : 00
we're flexible.

1 B

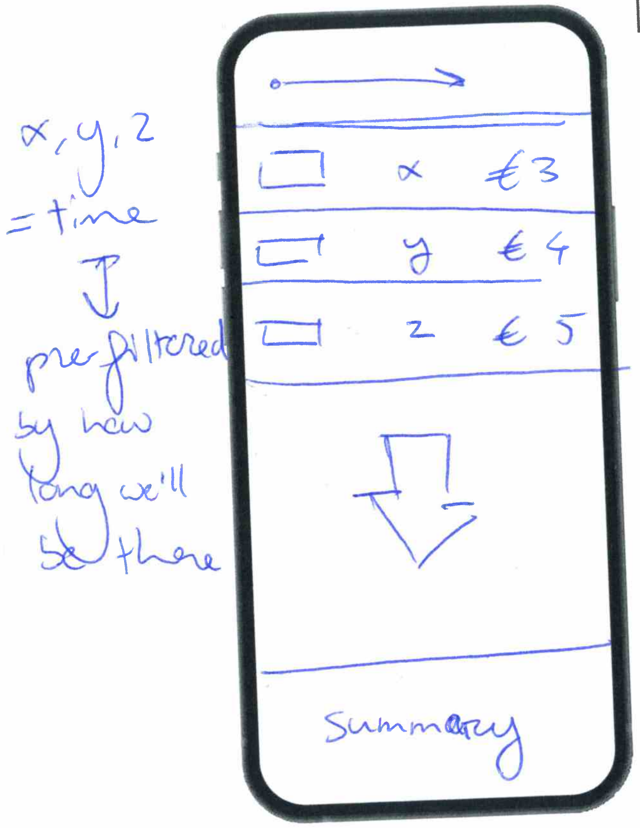
x, y, z
= time
pre-filtered
by how
long we'll
be there
x €3
y €4
z €5
Summary

1 C

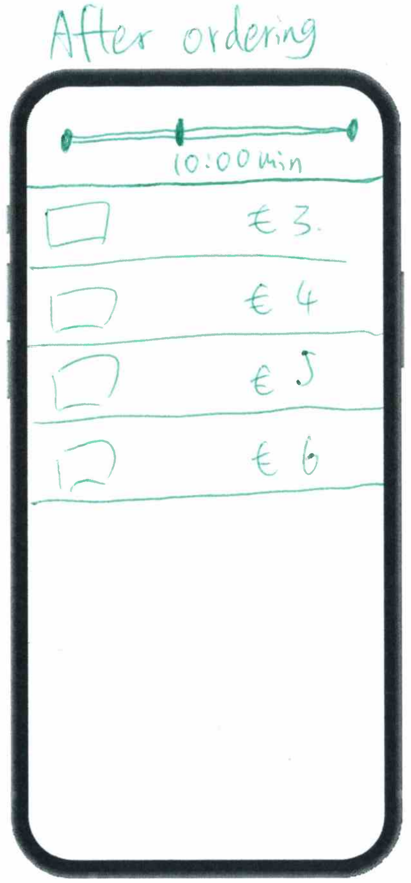
After ordering
10:00 min
€ 3.
€ 4
€ 5
€ 6

3

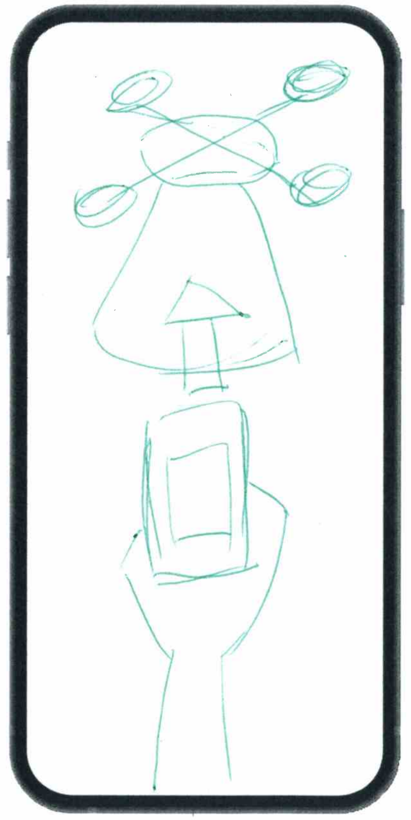

4

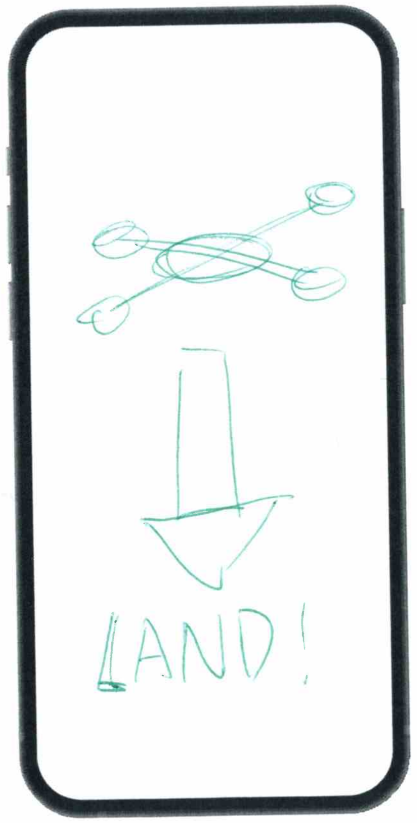
LAND!

5

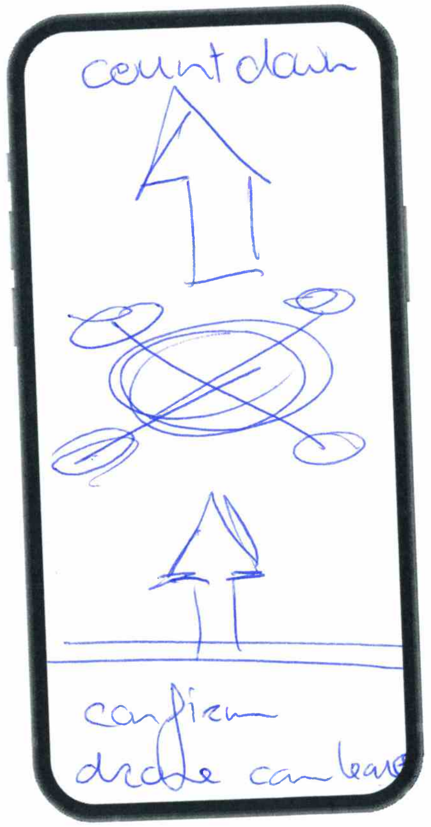
countdown
confirm
drone can leave

④ Recipient

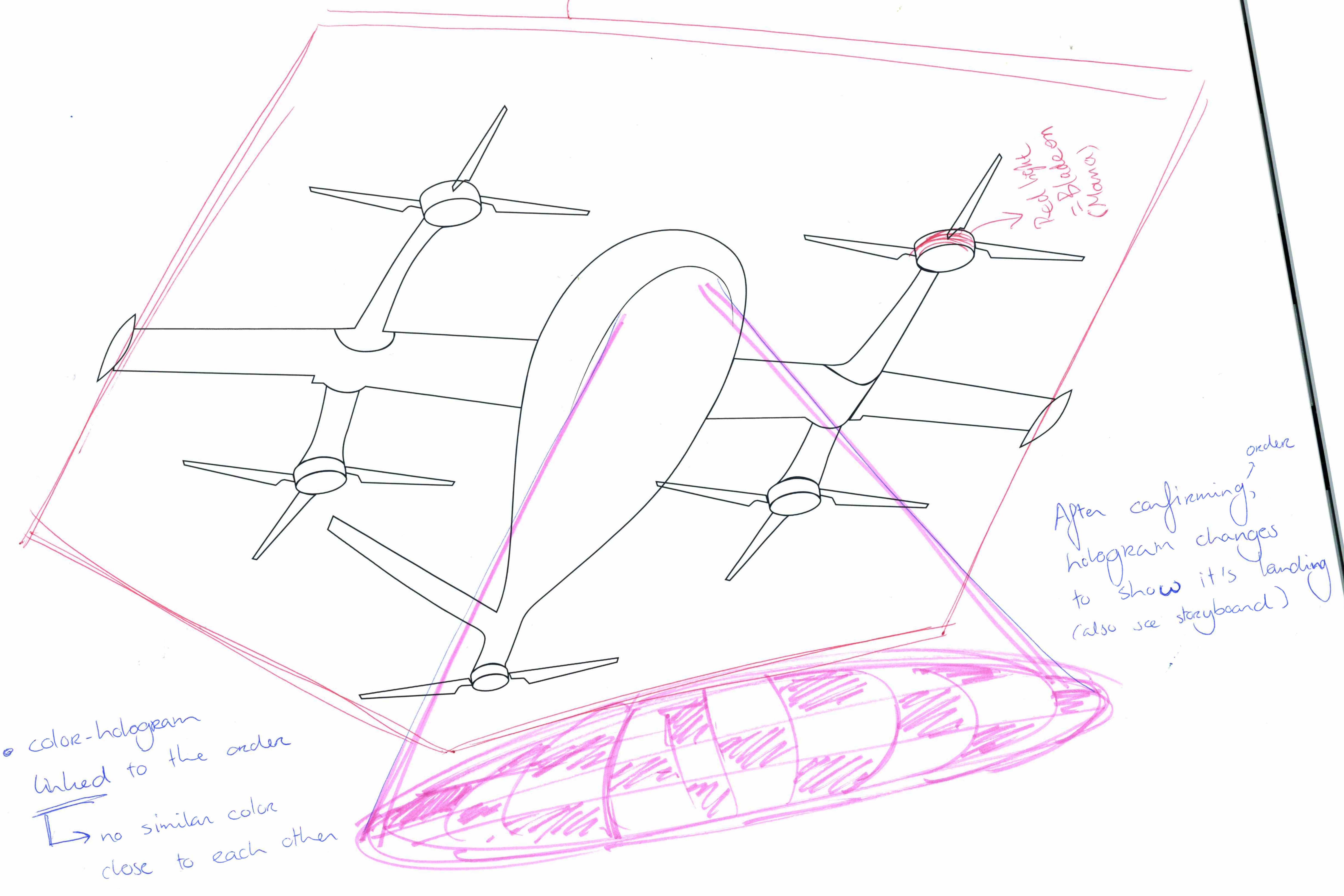
safety shield Amazon drone
Red light = Bladeon (Mano)
After confirming order, hologram changes to show it's landing (also see storyboard)
• color-hologram linked to the order
no similar color close to each other

Storyboard an interaction

Group no: 4

Role: Bystander

Notice drone (1)

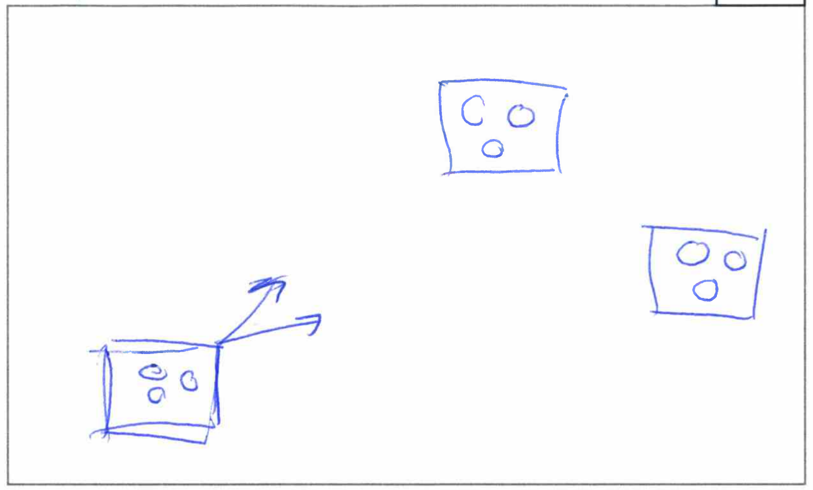

1. Hear the sound
2. See the drone flying over
3. Read text on drone

See drone hovering (2)

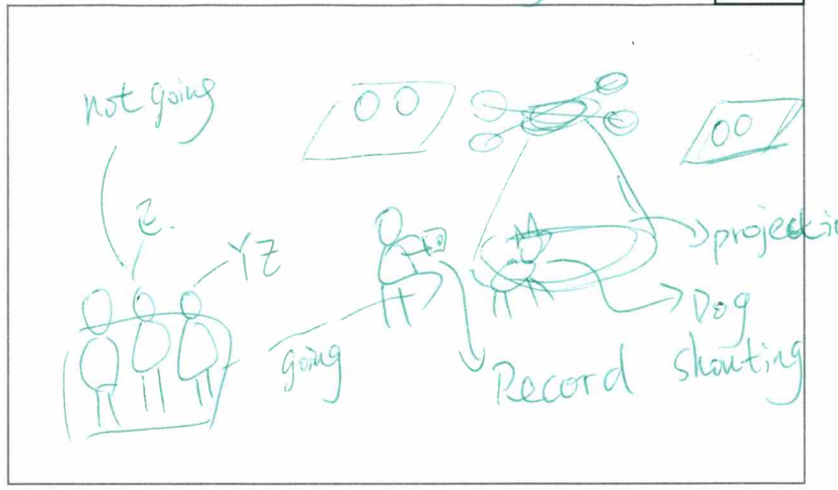

1. Someone stays, someone approaches
2. ~~0~~ I am careful to stay out of projection (clear line)
3. Dog goes under ~~bay~~ the drone & bark
4. I notice it

Recipient confirms ~~Buyer~~ Co with Drone (3)

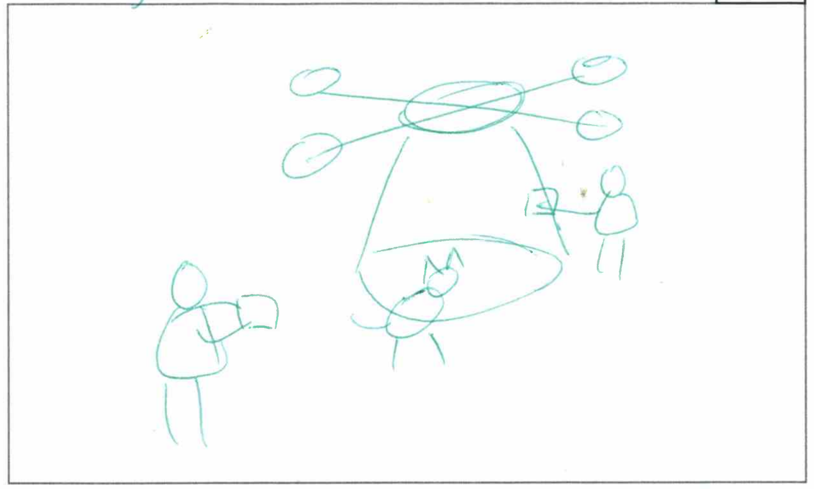

1. Recipient come, shows up phones to confirm with drone under projection
~~3~~. 2. Recipient notice my dog and tell me to control it.

Drone ~~nee~~ landing (4)

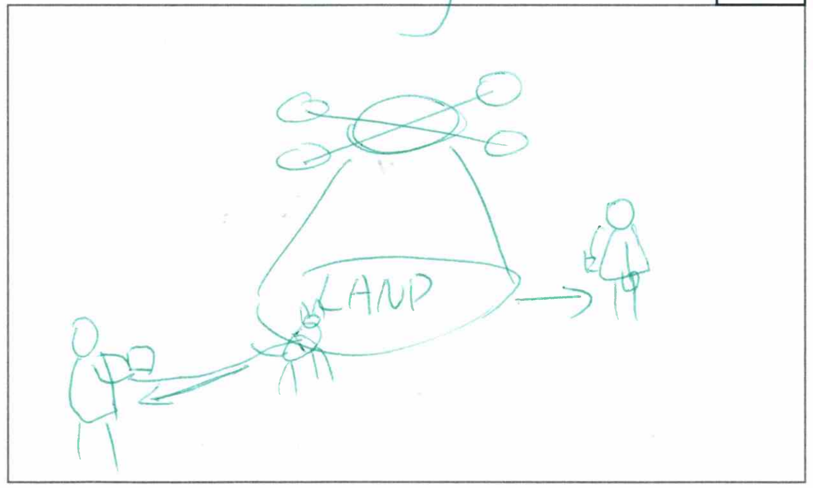

1. I see recipient staying away frome drone.
2. I see LANP projection & sound
3. I go take away my dog

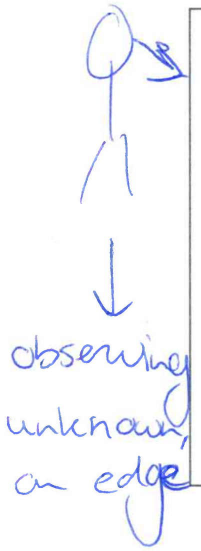

Drone stops on ground (5)

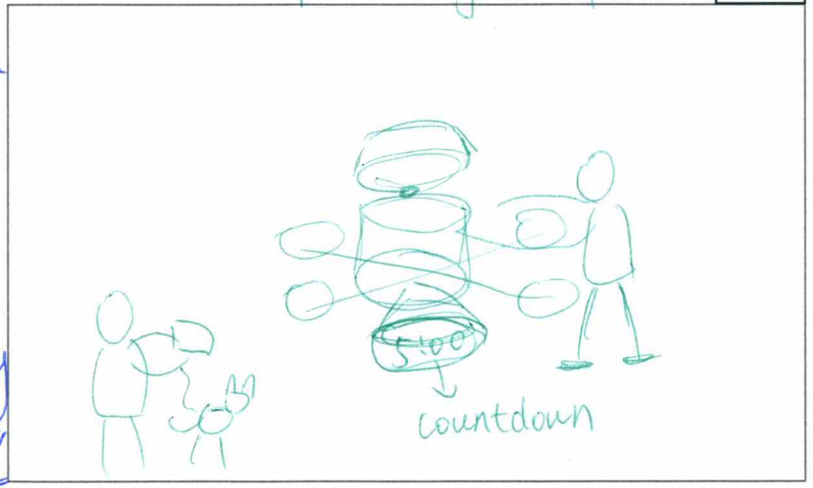

1. Dog controlled
2. Countdown starts
3. Everyone standby and look

Drone leaving (6)

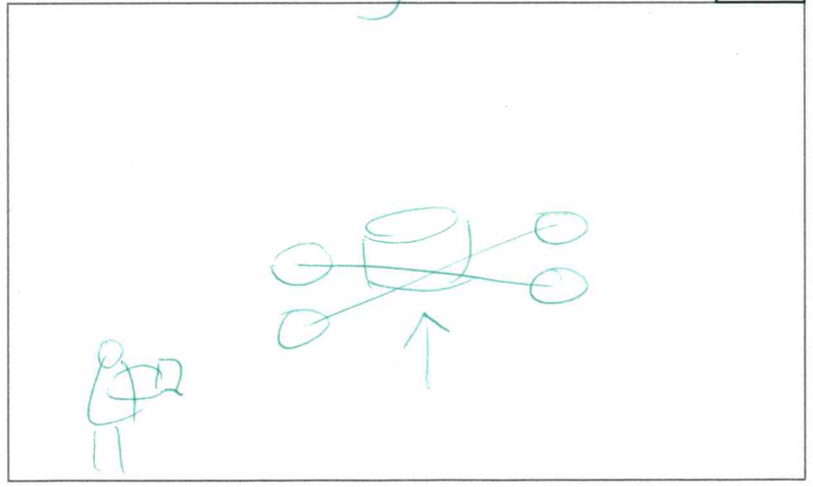

1. Countdown ends
2. Drone leave
3. ~~We~~ I am back and show videos

④ Bystander

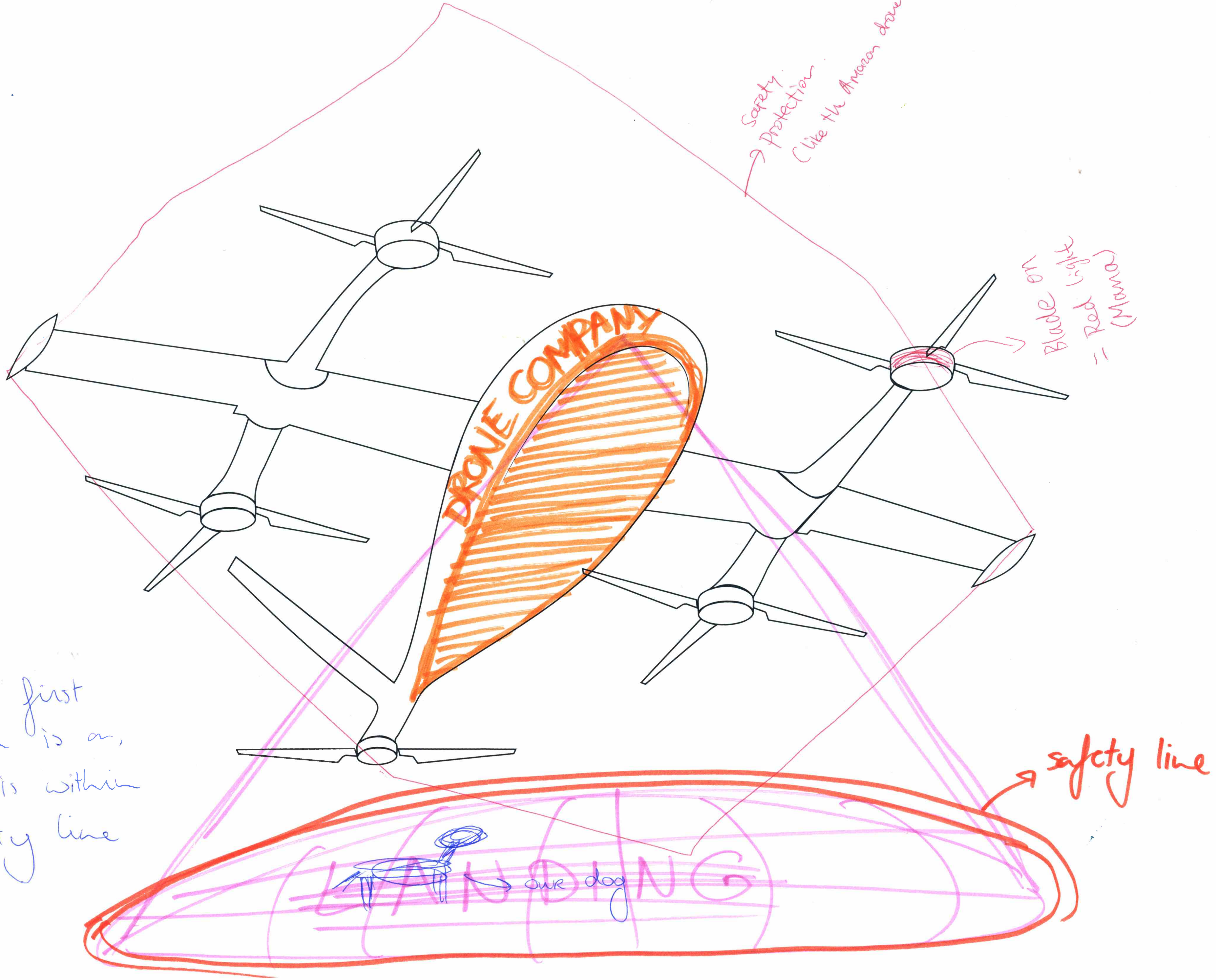

when the first hologram is on, our dog is within the safety line
