# Supplementary material for: Human interactions with delivery drones in public spaces: design recommendations from recipient and bystander perspectives
Source: Front Robot AI. 2025 May 30;12:1580289. doi: 10.3389/frobt.2025.1580289 (PMC12162322; doi:10.3389/frobt.2025.1580289)
Supplement: Supplementary file 1 [file DataSheet1.zip › Data_&_results/Interviews/Interview_codes_MaxQDA.pdf]

01-04-2025

Themes, codes and quotations from the interview transcriptions

Table of contents

1. Documents 4
2. Factors contributing to user uncertainty about HDI 5
   2.1. Criticality of the situation 5
      2.1.1. Role transition: bystander to recipient 5
      2.1.2. Criticality effect 7
   2.2. Familiarity with delivery drone technology and processes 10
      2.2.1. Technology 10
      2.2.2. Process 12
   2.3. Privacy concerns 16
   2.4. Public space dynamics 23
      2.4.1. Role of external agents 24
      2.4.2. Environmental factors 26
      2.4.3. Presence of multiple recipients and drones 27
   2.5. Differences in human roles 28
      2.5.1. Recipient 29
      2.5.2. Bystander 33
3. User requirements to feel certain during HDI 39
   3.1. Tracking information for the recipient 39
      3.1.1. Precision 40
      3.1.2. Time details 42
      3.1.3. Process tracker 46
      3.1.4. Live location 47
   3.2. Limited human intervention in drone control 50
      3.2.1. Level of participation 50
      3.2.2. Control 52
   3.3. Landing/take-off intention of the drone 58
      3.3.1. Signal of landing and take-off 58
      3.3.2. Location 60
      3.3.3. Delivery method 63
   3.4. Recognition of the drone and recipient 66
      3.4.1. Details of recipient 66
      3.4.2. Tutorial 67
      3.4.3. Presence 70
      3.4.4. Verification 72
      3.4.5. Recognise own drone 76
      3.4.6. Purpose 78

4. Drone design solutions to address uncertainty in HDI 83
4.1. Use case dependent 83
4.1.1. Appearance - non-emergency 83
4.1.2. HMIs - emergency 85
4.1.3. Appearance - emergency 90
4.2. Human-machine interfaces to communicate drone intentions 96
4.2.1. Phone 96
4.2.2. Non-semantic audio 108
4.2.3. Semantic audio 114
4.2.4. Display 117
4.2.5. Ground projection 118
4.2.6. Lights 120
4.3. Drone appearance 123
4.3.1. Colours/stickers 124
4.3.2. Friendly/rounded looks 127

1. Documents

No.	Document name (Format : I(participant_ID))	Created by
1	I12	Analyst1
2	I11	Analyst1
3	I10	Analyst1
4	I9	Analyst1
5	I8	Analyst1
6	I7	Analyst1
7	I6	Analyst1
8	I5	Analyst1
9	I4	Analyst1
10	I3	Analyst1
11	I2	Analyst1
12	I1	Analyst1

2. Factors contributing to user uncertainty about HDI

1.
"Because of unpredictability."
Code: ● Uncertainty factors   Weight score: 0
I9 , 19 - 19
Created: 26-07-2024 14:24 by Analyst1, Modified: 08-08-2024 9:17 by Analyst1
Area: 28   0.11%

2.
"It feels like a piece of technology that you are not controlling. With the human, you kind of expect a certain behavior, but now (with the drone) you don't know who is deciding and you're definitely not deciding."
Code: ● Uncertainty factors   Weight score: 0
I9 , 19 - 19
Created: 26-07-2024 14:25 by Analyst1, Modified: 08-08-2024 9:17 by Analyst1
Area: 212   0.81%

2.1. Criticality of the situation

2.1.1. Role transition: bystander to recipient

1.
"if somebody who has experience with medical (emergency), they could potentially go see if there's anything they can do."
Code: ● Role transition: bystander to recipient   Weight score: 0
I12 , 175 - 175
Created: 29-07-2024 12:14 by Analyst1, Modified: 02-09-2024 16:25 by Analyst1
Area: 119   0.56%

2.
"For instance, if the person is not able to pick the medicine, I can deliver (it) to the person. If I am taking care of someone that ordered this, I can go there and pick. So I think it could be interesting."
Code: ● Role transition: bystander to recipient   Weight score: 0
I11 , 174 - 174
Created: 26-07-2024 16:18 by Analyst2, Modified: 08-08-2024 9:53 by Analyst1
Area: 206   1.03%

3.
"If the recipient is not able to pick the package. It's like I can deliver for the person (needing it). I think would be interesting to know who is the recipient."
Code: ● Role transition: bystander to recipient   Weight score: 0
I11 , 190 - 190
Created: 26-07-2024 16:20 by Analyst2, Modified: 08-08-2024 9:53 by Analyst1
Area: 161   0.80%

4.
"If it is for emergencies, it could happen on streets, I will imagine that it is could happen for people I know. If I want to save these people"
Code: ● Role transition: bystander to recipient   Weight score: 0
I10 , 75 - 75
Created: 26-07-2024 16:43 by Analyst2, Modified: 02-09-2024 16:23 by Analyst1
Area: 142   0.65%

5.
"If someone ordered emergence medicine in public area, he might still be conscious, when they receive the medicine so he can do it by himself. But if he lost his consciousness, it will be

like they have diabetes and they already fainted and but they need insulin (immediately). In that case, the bystander will be the one who actually becomes the recipient."

Code: ● Role transition: bystander to recipient   Weight score: 0
I10 , 148 - 148
Created: 26-07-2024 16:58 by Analyst2, Modified: 02-09-2024 16:23 by Analyst1
Area: 356   1.62%

6.

"If someone is a medical student or nurse within the park, they can rush over and can do the first aid and help them."

Code: ● Role transition: bystander to recipient   Weight score: 0
I7 , 185 - 185
Created: 26-07-2024 12:37 by Analyst1, Modified: 02-09-2024 16:31 by Analyst1
Area: 116   0.45%

7.

"when it's an emergency drone, I think there's also a certain sense of urgency. Of also being able to support whoever is having that interaction with that interaction. I'm not quite sure how that will work, but I do feel like there's some involvement I would want to be able to have as a bystander there because someone apparently is in need of emergency medication. That also means that it's a scenario where it is actually nice to have some involvement from someone else who is there, but at the same time you don't want everyone around there to be involved"

Code: ● Role transition: bystander to recipient   Weight score: 0
I6 , 177 - 177
Created: 29-07-2024 11:33 by Analyst2, Modified: 01-08-2024 13:52 by Analyst1
Area: 558   1.37%

8.

"The only case that came to my mind is like an allergy to food. So they're swelling, and they're airway is closing up. And I'm hoping that they have friends around to order it for them. It's not just themselves. And if they want attention from other people, I think they can"

Code: ● Role transition: bystander to recipient   Weight score: 0
I5 , 175 - 175
Created: 25-07-2024 16:33 by Analyst1, Modified: 02-09-2024 16:02 by Analyst1
Area: 273   0.67%

9.

"If I'm a doctor, maybe I want to know what's the situation, because maybe I could be of help"

Code: ● Role transition: bystander to recipient   Weight score: 0
I5 , 244 - 244
Created: 25-07-2024 16:43 by Analyst1, Modified: 02-09-2024 16:02 by Analyst1
Area: 92   0.23%

10.

"I say, this person maybe is already laying on the ground then I'm pretty sure this is the person who needs the medicine. I will then assist him or them to get the package. If it is a person who ordered the medicine but still have capability to receiving it, I shouldn't distract them."

Code: ● Role transition: bystander to recipient   Weight score: 0
I3 , 217 - 217
Created: 02-08-2024 10:35 by Analyst1, Modified: 02-09-2024 15:55 by Analyst1
Area: 284   1.56%

11.

"if it's going to land and provide this stuff, the other people can take care of or they can move away or they can just understand what the drone is doing and they can provide more support"

Code: ● Role transition: bystander to recipient   Weight score: 0
I2 , 62 - 62
Created: 29-07-2024 14:49 by Analyst2, Modified: 02-09-2024 16:26 by Analyst1
Area: 187   0.79%

2.1.2. Criticality effect

1.
"Yeah. So I imagine for this kind of design, maybe there should be the ethical designer as well. There is somebody who, from medical background, could predefine such thing (reflection on criticality)."

Code: ● Criticality effect   Weight score: 0
I12 , 179 - 179
Created: 07-08-2024 16:43 by Analyst1, Modified: 02-09-2024 16:25 by Analyst1
Area: 199   0.94%

2.
"(It helps me) to not block (the course of the drone) or to know not to engage, or maybe someone wants to steal. Not everyone is thinking of the same."

Code: ● Criticality effect   Weight score: 0
I8 , 166 - 166
Created: 26-07-2024 14:05 by Analyst1, Modified: 02-09-2024 16:30 by Analyst1
Area: 149   0.55%

3.
"I'm the recipient and if I'm in a rush of getting kind of medicine or something, I need to know when it can be delivered. If it is like an emergency, I can go through or something. So delivery timing is important.

Analyst1 05:40
It's not an emergency, it's like a grocery like a snack. And you think it's still relevant?

Participant 05:51
In a park, maybe no. Then it gonna differ between what is the outer product (or how the package looks like). For example, for medicine, the timing is important. If they need some EpiPen kind of thing you need to know like, it's an emergency. If it is, like snacks, or kids something. I think it's not a Must Have."

Code: ● Criticality effect   Weight score: 0
I7 , 31 - 35
Created: 29-07-2024 10:37 by Analyst2, Modified: 02-09-2024 16:31 by Analyst1
Area: 668   2.61%

4.
"when I imagine a scenario was describing earlier for the snack, like if it takes some time for it to actually find the right person, ohh, that would bother me a lot when it happens in the emergency medicines"

Code: ● Criticality effect   Weight score: 0
I6 , 100 - 100
Created: 25-07-2024 18:33 by Analyst1, Modified: 01-08-2024 13:50 by Analyst1
Area: 207   0.51%

5.
"It's the same for me. I would just keep this the same because I think that is a very logical interaction. I would just be more frustrated if it takes longer with the medication scenario. So there's a bit of the: is there a more optimal way to do it question, that pops up when I'm thinking about the second scenario."

Code: ● Criticality effect   Weight score: 0
I6 , 104 - 104
Created: 29-07-2024 11:18 by Analyst2, Modified: 01-08-2024 13:50 by Analyst1
Area: 316   0.77%

6.

"for example, I would see after being used to this seeing one of those emergency medicines drones for the first time, I would be like, wait, what is going on here? So this is a different situation that would bring quite a bit of uncertainty, because that doesn't fade to the background because it's not something we're used to."

Code: ● Criticality effect   Weight score: 0
I6 , 149 - 149
Created: 26-07-2024 11:27 by Analyst1, Modified: 01-08-2024 13:50 by Analyst1
Area: 326   0.80%

7.

"it's a scenario where it is actually nice to have some involvement from someone else who is there, but at the same time you don't want everyone around there to be involved"

Code: ● Criticality effect   Weight score: 0
I6 , 177 - 177
Created: 26-07-2024 11:34 by Analyst1, Modified: 01-08-2024 15:42 by Analyst1
Area: 171   0.42%

8.

"with the signaling of it being an emergency drone (Note: signaling of emergency drone0 does also come a certain: What can you expect from it? So it does mean that culture-wise as well, you need to know, right that's an emergency drone, when one lands, there is someone around looking for medication"

Code: ● Criticality effect   Weight score: 0
I6 , 185 - 185
Created: 26-07-2024 11:36 by Analyst1, Modified: 01-08-2024 13:50 by Analyst1
Area: 298   0.73%

9.

"If you hear sirens going and you're on the road, you know you make way for them. There's a certain---. It's not necessarily a rule, but it is something you know that you do and there is certain code of conduct in that that does need to happen to actually get into the interactions that I'm describing. Especially with for example, the pop up of: Hey, someone around you is in need of help and signaling of"

Code: ● Criticality effect   Weight score: 0
I6 , 185 - 185
Created: 26-07-2024 11:36 by Analyst1, Modified: 01-08-2024 13:50 by Analyst1
Area: 405   0.99%

10.

"Oh wow yeah. I mean it's different because while you're in the emergency situation, right? So you want to know if help is there or not and this is more important in emergency situation. You have to know if it's here or if it's just someone else's snack because you're going to be ready for it then. I think it's important for people to know that help is already there. Than if it's a snack, I think it's less important for people to know, like is it my snack or not like it could be good, but it's not extremely necessary. But for emergencies, I think it is."

Code: ● Criticality effect   Weight score: 0
I5 , 179 - 179
Created: 29-07-2024 11:55 by Analyst2, Modified: 02-09-2024 16:02 by Analyst1
Area: 558   1.37%

11.

"'m more just thinking of from a bystander perspective, I feel like I do not need to participate in this in whatever way, so I don't really care."

Code: ● Criticality effect   Weight score: 0
I5 , 243 - 243
Created: 25-07-2024 16:41 by Analyst1, Modified: 02-09-2024 16:02 by Analyst1
Area: 144   0.35%

12.

"I hate the kind of people that see something is happening. They kind of just like run to it without thinking if they're gonna help or not, they end up causing more problems. I just hate to be that kind of people. So like I usually approach very cautiously."

Code: ● Criticality effect   Weight score: 0
I5 , 244 - 244
Created: 25-07-2024 16:43 by Analyst1, Modified: 02-09-2024 16:02 by Analyst1
Area: 256   0.63%

13.

"Participant 18:26
It's urgent, I need to know where it flies. Otherwise, I can think about the other solution. Or do I need to have an alternative plan? I can call the ambulance somewhere else, right. If the drone comes out? Yeah, so basically all the information matters. It matters more. My waiting time is more urgent than the food delivery or grocery delivery. So I can prepare the alternative plan."

Code: ● Criticality effect   Weight score: 0
I3 , 110 - 111
Created: 29-07-2024 12:23 by Analyst2, Modified: 02-09-2024 15:55 by Analyst1
Area: 404   2.22%

14.

"people will also understand this; if it's going to land and provide this stuff, the other people can take care of or they can move away or they can just understand what the drone is doing and they can provide more support."

Code: ● Criticality effect   Weight score: 0
I2 , 62 - 62
Created: 24-07-2024 14:22 by Analyst1, Modified: 02-09-2024 16:26 by Analyst1
Area: 222   0.94%

15.

"when we change the scenario, there is also a change in terms of priority"

Code: ● Criticality effect   Weight score: 0
I1 , 122 - 122
Created: 24-07-2024 13:12 by Analyst1, Modified: 02-09-2024 16:29 by Analyst1
Area: 72   0.29%

16.

"It is really different with the first case (snacks delivery) where even if the drone arrived five minutes late, that's fine. But now (with the medical emergency), I'm really like waiting for the drone. Like, come on."

Code: ● Criticality effect   Weight score: 0
I1 , 122 - 122
Created: 24-07-2024 13:14 by Analyst1, Modified: 02-09-2024 16:29 by Analyst1
Area: 216   0.87%

17.

"In this emergency situation, we are always optimizing our actions every minute, always dynamic, always changing. The location of pickup probably, like also dynamic and also changes."

Code: ● Criticality effect   Weight score: 0
I1 , 138 - 138
Created: 24-07-2024 13:21 by Analyst1, Modified: 02-09-2024 16:29 by Analyst1
Area: 181   0.73%

18.

"there's an emergency, there's a drone bringing medicine packages, like, either you stay away and the professionals can help. I guess that's the best thing you can do in here. But if it's in Indonesia, everyone will try to help anyway. So it really depends on the culture."

Code: ● Criticality effect   Weight score: 0
I1 , 178 - 178

Created: 24-07-2024 13:29 by Analyst1, Modified: 02-09-2024 16:29 by Analyst1
Area: 271   1.09%

2.2. Familiarity with delivery drone technology and processes

1.
"I would say keep the interaction way simple. I would say not make it too complex (as) it's harder for human to adopt, as well. I think just keep it as neutral and no more interaction than humans has with (traditional delivery)."
Code: ● Familiarity   Weight score: 0
I3 , 233 - 233
Created: 24-07-2024 15:05 by Analyst1, Modified: 02-09-2024 15:55 by Analyst1
Area: 227   1.24%

2.
"For example, if it is for food delivery, then imagine the drone is the food delivery guy, and what you expect from them. But if it's for emergency, you can think about an ambulance, what it is supposed to have. The basic necessities for those two elements, like food delivery and emergency things."
Code: ● Familiarity   Weight score: 0
I3 , 233 - 233
Created: 24-07-2024 15:05 by Analyst1, Modified: 02-09-2024 15:55 by Analyst1
Area: 297   1.63%

2.2.1. Technology

1.
"I could imagine people having curiosity about high technology because this would be something new."
Code: ● Technology   Weight score: 0
I12 , 125 - 125
Created: 29-07-2024 12:10 by Analyst1, Modified: 02-09-2024 16:25 by Analyst1
Area: 98   0.46%

2.
"If we're talking about a scenario that everybody getting used to the delivery drone, I probably won't feel uncertain. But if it's something new to me, I saw it the first time, I will be like, Oh, I don't know what is this? Then that's more uncertain."
Code: ● Technology   Weight score: 0
I12 , 129 - 129
Created: 29-07-2024 12:11 by Analyst1, Modified: 02-09-2024 16:25 by Analyst1
Area: 250   1.17%

3.
"If it is my first time, I will feel uncertain about it. As a second time, it's like, I already know what will happen."
Code: ● Technology   Weight score: 0
I11 , 18 - 18
Created: 29-07-2024 11:26 by Analyst1, Modified: 08-08-2024 9:53 by Analyst1
Area: 117   0.58%

4.
"I don't know how the drone will behave as I never saw it before."
Code: ● Technology   Weight score: 0
I11 , 26 - 26
Created: 29-07-2024 11:28 by Analyst1, Modified: 08-08-2024 9:53 by Analyst1
Area: 64   0.32%

5.

"it will be weird. If I'm expecting something smaller, and it turns out to be way bigger"

Code: ● Technology   Weight score: 0
I9 , 73 - 73
Created: 26-07-2024 14:32 by Analyst1, Modified: 08-08-2024 9:17 by Analyst1
Area: 87   0.33%

6.

"Because of the newness like, you don't know how does it (drone delivery) happen? There is no previous context that you have as a user."

Code: ● Technology   Weight score: 0
I9 , 147 - 147
Created: 26-07-2024 14:53 by Analyst1, Modified: 08-08-2024 9:17 by Analyst1
Area: 134   0.51%

7.

"the first time (I am) using, I would have some kind of uncertainty. I can imagine that I'm not going to be the only person receiving some things. If out of the sudden I start seeing the drones around me (Id feel uncertain)."

Code: ● Technology   Weight score: 0
I8 , 14 - 14
Created: 26-07-2024 13:12 by Analyst1, Modified: 02-09-2024 16:30 by Analyst1
Area: 223   0.82%

8.

"I think it would also take some time to actually know that I can trust this."

Code: ● Technology   Weight score: 0
I6 , 108 - 108
Created: 29-07-2024 11:20 by Analyst2, Modified: 01-08-2024 13:50 by Analyst1
Area: 76   0.19%

9.

"I think the first summer that you have these delivery drones up and running, there is going to be some uncertainty because you're not quite sure what's happening. You need to see these interactions actually happening in your vicinity. But when we get to the second summer, you've seen them, we've all seen them around. We all know what to expect from the drones, you know."

Code: ● Technology   Weight score: 0
I6 , 129 - 129
Created: 26-07-2024 11:20 by Analyst1, Modified: 01-08-2024 13:50 by Analyst1
Area: 372   0.91%

10.

"over time it starts fading into the background, the more they start becoming part of that environment. So it's not necessarily the uncertainty that's necessarily becoming ----. Overtime, if you would graph out your uncertainty after that first week"

Code: ● Technology   Weight score: 0
I6 , 133 - 133
Created: 26-07-2024 11:24 by Analyst1, Modified: 01-08-2024 13:50 by Analyst1
Area: 248   0.61%

11.

"I was also thinking in the future, in the sense of, do we already know that there are drones that are delivering food for people because knowing that or not, I think makes a difference for how do you react to what you see when the drone is flying"

Code: ● Technology   Weight score: 0
I5 , 4 - 4
Created: 25-07-2024 15:45 by Analyst1, Modified: 02-09-2024 16:02 by Analyst1
Area: 246   0.60%

12.
"I'm kind of just like trying to create two scenarios where people have some knowledge on delivery drones and not having knowledge on delivery drones."
Code: ● Technology Weight score: 0
I5 , 5 - 5
Created: 25-07-2024 15:47 by Analyst1, Modified: 02-09-2024 16:02 by Analyst1
Area: 149 0.36%

13.
"it's our first time ordering this, considering if we know very little about these kind of things, then we would probably feel excited and be like: Well, is it gonna fall?"
Code: ● Technology Weight score: 0
I5 , 25 - 25
Created: 25-07-2024 16:06 by Analyst1, Modified: 02-09-2024 16:02 by Analyst1
Area: 170 0.42%

14.
"drones make quite a bit of noise. I think that's something more maybe emotional or more primal, but that wouldn't help with making me feel like safe or like comfortable with situation. At least not the first times (very begining stages)."
Code: ● Technology Weight score: 0
I4 , 11 - 11
Created: 25-07-2024 11:34 by Analyst1, Modified: 02-09-2024 15:58 by Analyst1
Area: 237 1.05%

15.
"I would say too much signaling will make me uncertain, because I don't know what to do. If I am the new user, or people who are not familiar with drone, I don't know what to expect."
Code: ● Technology Weight score: 0
I3 , 237 - 237
Created: 24-07-2024 15:05 by Analyst1, Modified: 02-09-2024 15:55 by Analyst1
Area: 181 0.99%

16.
"not many people have experiences like receiving something from drones"
Code: ● Technology Weight score: 0
I1 , 10 - 10
Created: 24-07-2024 12:21 by Analyst1, Modified: 02-09-2024 16:29 by Analyst1
Area: 69 0.28%

17.
"the factor from the delivery methods itself, because I'm unfamiliar with drones."
Code: ● Technology Weight score: 0
I1 , 38 - 38
Created: 24-07-2024 12:52 by Analyst1, Modified: 02-09-2024 16:29 by Analyst1
Area: 80 0.32%

2.2.2. Process

1.
"I don't know how they deliver it. I don't know if the drone would land or how are the package being delivered. Is it gonna land or not, like the more detailed behavior."
Code: ● Process Weight score: 0
I12 , 33 - 33
Created: 29-07-2024 11:55 by Analyst1, Modified: 02-09-2024 16:25 by Analyst1
Area: 168 0.79%

2.

"how are the package being delivered (flying route, gonna land or not)), again, I don't know how this drone works. It's a Must Have. If it happened already once then I know, it becomes a Could Have."

Code: ● Process   Weight score: 0
I12 , 49 - 49
Created: 29-07-2024 12:00 by Analyst1, Modified: 02-09-2024 16:25 by Analyst1
Area: 197   0.93%

3.
"But if it's something new to me, I saw it the first time, I will be like, Oh, I don't know what is this? Then that's more uncertain."

Code: ● Process   Weight score: 0
I12 , 129 - 129
Created: 26-07-2024 13:08 by Analyst2, Modified: 02-09-2024 16:25 by Analyst1
Area: 132   0.62%

4.
"If it is my first time, I will feel uncertain about it. As a second time, it's like, I already know what will happen."

Code: ● Process   Weight score: 0
I11 , 18 - 18
Created: 26-07-2024 13:44 by Analyst2, Modified: 08-08-2024 9:53 by Analyst1
Area: 117   0.58%

5.
"Because of unpredictability. It feels like a piece of technology that you are not controlling. With the human, you kind of expect a certain behavior, but now (with the drone) you don't know who is deciding and you're definitely not deciding."

Code: ● Process   Weight score: 0
I9 , 19 - 19
Created: 29-07-2024 8:17 by Analyst2, Modified: 08-08-2024 9:17 by Analyst1
Area: 241   0.92%

6.
"Assuming that this is my first time doing a delivery like this, I don't know where this drone is going to land, and how is this delivery going to happen."

Code: ● Process   Weight score: 0
I9 , 41 - 41
Created: 26-07-2024 14:27 by Analyst1, Modified: 08-08-2024 9:17 by Analyst1
Area: 153   0.58%

7.
"s. It's also something to do with the number of times this delivery is happening, right?"

Code: ● Process   Weight score: 0
I9 , 57 - 57
Created: 29-07-2024 8:45 by Analyst2, Modified: 08-08-2024 9:17 by Analyst1
Area: 88   0.34%

8.
"Analyst1 34:08
Yeah. And this is something else that also makes you are uncertain?

Participant 34:14
About the delivery location also. Where, when and how is it going to place the parcel, like drop it off.

Analyst1 34:23
Yeah. Why?

Participant 34:27
Because of the newness like, you don't know how does it (drone delivery) happen? There is no previous context that you have as a user."

Code: ● Process Weight score: 0
I9 , 140 - 147
Created: 29-07-2024 9:04 by Analyst2, Modified: 08-08-2024 9:17 by Analyst1
Area: 415 1.58%

9.
"Maybe in the beginning, the first time (I am) using, I would have some kind of uncertainty"

Code: ● Process Weight score: 0
I8 , 14 - 14
Created: 29-07-2024 9:41 by Analyst2, Modified: 02-09-2024 16:30 by Analyst1
Area: 90 0.33%

10.
"As I told you, I believe if there is a service like that (drones delivering packages) more people are going to use it. My point is that I don't know what is coming, (if) it's my delivery, or maybe it's for someone else. If I am in a park, and just see drones flying around, maybe I'm not gonna be sure if that's going to be my package or if I have to go to stand somewhere specifically (to receive the package)."

Code: ● Process Weight score: 0
I8 , 18 - 18
Created: 29-07-2024 9:41 by Analyst2, Modified: 02-09-2024 16:30 by Analyst1
Area: 411 1.52%

11.
"How used are you to the situation to actually know what you can expect and also take away some of the uncertainty of when and a drone is actually flying out to you."

Code: ● Process Weight score: 0
I6 , 17 - 17
Created: 01-08-2024 9:48 by Analyst1, Modified: 01-08-2024 13:50 by Analyst1
Area: 164 0.40%

12.
"Well, let's say time wise we're tracking over time a bit. These drones are becoming more mainstream. I think the first summer that you have these delivery drones up and running, there is going to be some uncertainty because you're not quite sure what's happening. You need to see these interactions actually happening in your vicinity. But when we get to the second summer, you've seen them, we've all seen them around. We all know what to expect from the drones, you know. And then the moment you just see something flying, even just the main color you all together think ohh, right, another one from that company. Oh, they're really making a marketing stunt for company number two. Is the same now also kind of happens when you look at delivery bikes and stuff. They kind of blend into the environment and you just notice the color and you already know what is happening. So that takes some time. But I think, to have that---."

Code: ● Process Weight score: 0
I6 , 129 - 129
Created: 29-07-2024 11:27 by Analyst2, Modified: 01-08-2024 13:50 by Analyst1
Area: 928 2.27%

13.
"Well, let's say it's the first week, I'm a bystander sitting in the park and first I'm going to hear this sound, you know, because I can hear this drone approaching, and I'm going to be looking around because that's one thing we tend to do if we're sitting somewhere and we hear a sound, we try to locate the sounds. So okay, I'm already distracted. Excuse me, I was having a nice picnic here. I'm distracted now, so I'm looking around and seeing what's going on and I spot a drone. I'm like: what is it drone doing here? I'm sitting here in the park. There's no one I would

check: Okay, there's no one actively controlling it, it seems because everyone is just also either in their own group, or also looking at this drone trying to figure out what's happening. And then it actually approaches a specific group of people. Okay, it's actually meant to be here. There's this confirmation of: Okay wait, it's not just randomly flying, it has a purpose here, somehow, for whatever reason. Then I see someone interact with that drone. It's like: Okay, so apparently there is something happening. All right, they get snacks from it. Okay, wait. Seriously, are people not even walking to the store anymore? Now they're just ordering their snacks by drone. What is happening here? And then as the weeks move by and it really becomes summer. Everyone is in the park and then it's just not that one color anymore. But those different companies start showing up and you're like: Oh, god, the sound I can't even focus anymore here I'm trying to enjoy a picnic. But all these different drones are flying around here, because all these people can't just walk through the store anymore. They're all just ordering. And then over time it starts fading into the background, the more they start becoming part of that environment. So it's not necessarily the uncertainty that's necessarily becoming ----. Overtime, if you would graph out your uncertainty after that first week, I would not say it changes that much. Once you know what to expect, it doesn't change that much. What mostly changes is the extent to which it actually fades into the background and to which it becomes a normalized part of what you see in your environments."

Code: ● Process Weight score: 0
I6 , 133 - 133
Created: 29-07-2024 11:28 by Analyst2, Modified: 01-08-2024 13:50 by Analyst1
Area: 2216 5.43%

14.

"If I am the new user, or people who are not familiar with drone, I don't know what to expect. If there's an extra interface on the drone, I don't know. I will say not to over design the interaction."

Code: ● Process Weight score: 0
I3 , 237 - 237
Created: 29-07-2024 12:30 by Analyst2, Modified: 02-09-2024 15:55 by Analyst1
Area: 198 1.09%

15.

"how can I like take something from drones? Do I need to wait? Do drones give signals? Is it safe to take the snacks? Or what do I need to do after the drones landed?"

Code: ● Process Weight score: 0
I1 , 10 - 10
Created: 24-07-2024 12:21 by Analyst1, Modified: 02-09-2024 16:29 by Analyst1
Area: 165 0.66%

16.

"Do they just throw my snacks around the sky and say bye? Do they actually fly around or hover like, two meters from the ground? Do I need to like, reach the packet or do they actually need to land on the grass?"

Code: ● Process Weight score: 0
I1 , 14 - 14
Created: 24-07-2024 12:32 by Analyst1, Modified: 02-09-2024 16:29 by Analyst1
Area: 210 0.85%

17.

"I'll compare this drone as an alternative methods of, like delivery, you could have bike delivery or a motorcycle delivery or car. There you have people. They usually have uniforms, orange, or whatever. First you can recognize the organization or the task. That is something that you are already familiar of like, you're taking something from human. We are used to getting like posts from the post officer packages from the DHL or post NL. Sometimes we need to sign and sometimes we don't. Sometimes they say that we are not at home, even though we are at home. So you already have some kind of anchor or reference point. But for drones, how do I react and what do I need to do when something goes wrong?"

Code: ● Process   Weight score: 0
I1 , 18 - 18
Created: 24-07-2024 12:37 by Analyst1, Modified: 02-09-2024 16:29 by Analyst1
Area: 704   2.83%

2.3. Privacy concerns

1.
"If the drone is not carrying anything, so I would think it is a personal drone. If it's approaching me, then probably (I am afraid that it is going to) shoot (a video of) me or it could be something from other people that I'm not expecting."
Code: ● Privacy   Weight score: 0
I10 , 15 - 15
Created: 26-07-2024 16:27 by Analyst2, Modified: 02-09-2024 15:53 by Analyst1
Area: 240   1.09%

2.
"Participant 02:40
The impression that I've with the drone is that it is a video shooting tool. If they are approaching you, most possibly they are shooting you and you won't feel comfortable because there's someone else hiding somewhere that you don't know. But they know where you are, and they are shooting videos of you without (your) permission. That's my first impression in drones in general, because I got this impression that drones are for video shooting."
Code: ● Privacy   Weight score: 0
I10 , 18 - 19
Created: 26-07-2024 16:28 by Analyst2, Modified: 02-09-2024 15:53 by Analyst1
Area: 465   2.11%

3.
"On the other hand, my impression to the drone is either a weapon or they are shooting videos. Both of them are quite offensive to privacy and to people (safety). You know that this drone is not yours."
Code: ● Privacy   Weight score: 0
I10 , 103 - 103
Created: 26-07-2024 16:49 by Analyst2, Modified: 02-09-2024 16:23 by Analyst1
Area: 200   0.91%

4.
"As a bystander, I just want to know, is it recording as a video or something? I don't want to be in the video. If it is recording of any kind even if the other recipient who is getting delivered by drones is next to me, I don't want. (Note: Is there a video capture or not)"
Code: ● Privacy   Weight score: 0
I7 , 137 - 137
Created: 29-07-2024 10:48 by Analyst2, Modified: 02-09-2024 16:31 by Analyst1
Area: 273   1.07%

5.
"usually people's first instinct is to, when people see a drone, typically they would think of, OK, well, is it something that is used for spying on people? Or are people just recording footage? Am I being recorded? Who is watching?"
Code: ● Privacy   Weight score: 0
I5 , 4 - 4
Created: 25-07-2024 15:44 by Analyst1, Modified: 02-09-2024 16:02 by Analyst1
Area: 231   0.57%

6.
"if you don't know that was the case and you think about like, okay, well, what is going on? And like, am I being recorded or anything? I think usually when we see drones like the ones that we have been exposed to the most are the ones like we also see on TV. Like your

annoying neighbour had a drone that is flying around their pool and then like people have, you know, like Modern Family, like discourse about it"

Code: ● Privacy   Weight score: 0
I5 , 4 - 4
Created: 25-07-2024 15:46 by Analyst1, Modified: 02-09-2024 16:02 by Analyst1
Area: 413   1.01%

7.

"they feel like, oh, am I being recorded because that I feel like it's going to stir a lot of negative reactions. So because we don't want to hate the drones if we want to use them."

Code: ● Privacy   Weight score: 0
I5 , 120 - 120
Created: 25-07-2024 16:21 by Analyst1, Modified: 02-09-2024 16:02 by Analyst1
Area: 180   0.44%

8.

"Uh, well, the same thing. I think it's for privacy reasons. I just want to know if I'm being recorded for something that I did, that I did not give consent for.
And also if it's going to like drop on my head and give me a concussion. Well, I don't know if it's that heavy, but you know, just safety and privacy I guess."

Code: ● Privacy   Weight score: 0
I5 , 201 - 202
Created: 29-07-2024 11:57 by Analyst2, Modified: 02-09-2024 16:02 by Analyst1
Area: 319   0.78%

9.

"I'm guessing the drone will atmost have maybe a camera on it, and maybe that will help a bit in assuring me because then I know it can detect people or faces. If that's not there, then I would be wondering like, how is it gonna know that it is me and how is it gonna do the delivery properly?"

Code: ● Privacy   Weight score: 0
I4 , 19 - 19
Created: 25-07-2024 11:39 by Analyst1, Modified: 02-09-2024 15:58 by Analyst1
Area: 292   1.29%

10.

"In society right now, in general, we have a bit of a fear of like cameras and like smart AI. Some people even have on their phone or laptops, when they don't want they block the camera. I think in general, there's a bit of fear for surveillance. If we have these drones flying around everywhere, and the cameras are always on, I think that will be a bit tough for people or some people might even get aggressive. They will try to shoot the drone or whatever, or harm it."

Code: ● Privacy   Weight score: 0
I4 , 103 - 103
Created: 25-07-2024 12:44 by Analyst1, Modified: 02-09-2024 15:58 by Analyst1
Area: 470   2.08%

11.

"If it would have a live camera on it, I would feel a bit watched, a bit uneasy, maybe because I'm being filmed, or whatever."

Code: ● Privacy   Weight score: 0
I4 , 127 - 127
Created: 29-07-2024 12:14 by Analyst2, Modified: 02-09-2024 15:58 by Analyst1
Area: 124   0.55%

12.

"This is my privacy. Do they record video? Take photo of my leisure time in the park? Or who places this drone in this park?"

Code: ● Privacy   Weight score: 0

I1 , 10 - 10
Created: 24-07-2024 12:22 by Analyst1, Modified: 02-09-2024 16:29 by Analyst1
Area: 123   0.50%

13.
"Even if you are used to like people flying drones in the touristic areas, you probably also be annoyed because you know if it's like tourists then they will probably turn on the video recording or take photo of you lying on the grass. That's not good for privacy."
Code: ● Privacy   Weight score: 0
I1 , 150 - 150
Created: 24-07-2024 13:24 by Analyst1, Modified: 02-09-2024 16:29 by Analyst1
Area: 263   1.06%

14.
"Yeah, basically, I'm relaxing on the park and suddenly (I see) flying object (and think) like what is this thing and what can it do? I am curious and it also concerns about privacy as drones flying above your head. It will be good if like... but I don't know how to communicate, this is really hard."
Code: ● Privacy   Weight score: 0
I1 , 158 - 158
Created: 29-07-2024 15:37 by Analyst2, Modified: 02-09-2024 16:29 by Analyst1
Area: 299   1.20%

15.
"I was also thinking, if you can even show what type of medicine. It depends, for example, I know if people have heart attack, then that really depends on time. If somebody is around closeby, if they can do something, that's very important. In this case, I would imagine if the drone would also serve as some kind of a sign, for people to know something's happening, to ask for help. If it's other things, maybe it's just very personal, then it's about privacy and ethical consideration, then maybe not."
Code: ● Privacy   Weight score: 0
I12 , 175 - 175
Created: 26-07-2024 13:27 by Analyst2, Modified: 02-09-2024 16:25 by Analyst1
Area: 502   2.36%

16.
"I don't want that everybody around me knows that I'm receiving something. So if there is a light or sound or something, maybe I can feel like I am disturbing people. So I just want my stuff."
Code: ● Privacy   Weight score: 0
I11 , 78 - 78
Created: 12-08-2024 14:05 by Analyst1, Modified: 12-08-2024 14:05 by Analyst1
Area: 190   0.95%

17.
"I think it (Note: package details) Won't Have. I think it's more related to privacy. As a bystander, I don't want to know what is the medicine for? I think it's very personal (for the recipient), the medicine (information). I think it's not important to know what is inside of the package, but only the name of the recipient. I think it's good."
Code: ● Privacy   Weight score: 0
I11 , 186 - 186
Created: 26-07-2024 16:19 by Analyst2, Modified: 08-08-2024 9:53 by Analyst1
Area: 344   1.72%

18.
"It's probably critical medicine for my personal use. It's just like a diabetes shot that I've forgotten at home, and I need it. I don't want to announce it to the world like, hey, that's why it's arriving. If it's for an accident scenario, where this critical medicine arriving for someone

to be used, then maybe yes, because everyone around is aware of it. But the use case of critical medicine is very different in both these scenarios."

Code: ● Privacy Weight score: 0
I9 , 123 - 123
Created: 26-07-2024 14:51 by Analyst1, Modified: 08-08-2024 9:17 by Analyst1
Area: 438 1.67%

19.

"If it's for critical medicine for personal use, I could also choose a good landing spot where I'm more comfortable receiving it."

Code: ● Privacy Weight score: 0
I9 , 131 - 131
Created: 26-07-2024 14:52 by Analyst1, Modified: 08-08-2024 9:17 by Analyst1
Area: 128 0.49%

20.

"Analyst1 12:25
Yeah, and next one reassurance of privacy?

Participant 12:28
I don't know if the drones are gonna have cameras, what's the way that they're gonna deliver (Note: reassurance of privacy). I want to believe that if you have cameras and you scan everything going around, I want to be a little bit sure what kind of data is going to be shared with who. Maybe the food (data) that's gonna deliver is going to get deleted, like it's not (just) about what I'm going to order but maybe where the drone is reaching or with who I am around.

Participant 12:29
Yeah, and why do you want to know this information?

Participant 13:09
Right now all the devices are collecting a lot of personal data. I would like to know, what I'm using at least, they're trying to maintain some kind of data privacy. Also for me with the camera, even you know with the laptops and everything, I'm trying to cover the cameras.

Analyst1 13:32
Okay, and how do you want to know this information?

Participant 13:36
I think for that information has to do first how the drone operates. I don't know if they need to have a camera to operate or not. That's only based on how the company, having these drones, are gonna reassure us. I'm not gonna read you know, the 100 pages of data privacy. But to have some key points about how my data is collected and used, I would like to have it in some bullet points to say this is the main key points for example."

Code: ● Privacy Weight score: 0
I8 , 59 - 70
Created: 29-07-2024 9:48 by Analyst2, Modified: 02-09-2024 16:30 by Analyst1
Area: 1463 5.40%

21.

"someone does not want (others) to know that they need medicines. Maybe you can select if you're in a good situation. So that is something of additional feature, (like) you know how much you are in need or the privacy."

Code: ● Privacy Weight score: 0
I8 , 116 - 116
Created: 26-07-2024 13:50 by Analyst1, Modified: 02-09-2024 16:30 by Analyst1
Area: 217 0.80%

22.

"Participant 28:39
It's (Note: reassurance of privacy) like I said before, with the privacy, to know if the data is going to be used, how many times maybe something was delivered, and just to know that the data is not going to any weird places."

Code: ● Privacy Weight score: 0
I8 , 127 - 128
Created: 29-07-2024 10:01 by Analyst2, Modified: 02-09-2024 16:30 by Analyst1
Area: 244 0.90%

23.

"if I want to have some kind of silence in and not have that sound (drone noise) all the time around me, things (packages) dropping, or, seeing drones left and right, I would like to know, in case I want to avoid that space to go."

Code: ● Privacy Weight score: 0
I8 , 150 - 150
Created: 26-07-2024 14:03 by Analyst1, Modified: 02-09-2024 16:30 by Analyst1
Area: 229 0.85%

24.

"With the privacy (Note: privacy), I don't know if it can be communicated, but I would like to know the access to (information) like where I live or my face because I'm a neighbour or whatever, and this is not going to be used somehow against me."

Code: ● Privacy Weight score: 0
I8 , 166 - 166
Created: 29-07-2024 10:31 by Analyst2, Modified: 02-09-2024 16:30 by Analyst1
Area: 245 0.90%

25.

"Analyst1 13:20
Is there something that you don't want to have?

Participant 13:23
My (personal) data. If I'm the recipient, I will give all my data and my location access. I don't want it to be like recorded, noted, taken into consideration in giving me notification, hey you did this and so on. I'm more like European in this part. I'll write data breach (Note: Data breach) [after thinking].

Analyst1 14:17
How do you want to know this data breach whether there is data breach or there will be no data breach?

Participant 14:37
Just like, allow essential cookies (on phone) representing whenever we download some app or something. Because within the app, it can say that we are taking into consideration apart from the privacy settings that we want to access your location or something. We can just say that allow access only for the time being, fitting the delivery part. Something like that."

Code: ● Privacy Weight score: 0
I7 , 60 - 67
Created: 29-07-2024 10:40 by Analyst2, Modified: 02-09-2024 16:31 by Analyst1
Area: 925 3.61%

26.

"If it is recording of any kind even if the other recipient who is getting delivered by drones is next to me, I don't want."

Code: ● Privacy Weight score: 0
I7 , 137 - 137
Created: 26-07-2024 12:32 by Analyst1, Modified: 02-09-2024 16:31 by Analyst1

Area: 122   0.48%

27.
"Analyst1 37:18
Okay. Is it something you want to know, from the way the drone looks? Or some elements of the drone? Or is it something like you want to see on your mobile or on the ground?

Participant 37:45
Even on my mobile, I think if I'm not the recipient, I'm not gonna get any data regarding anything delivered. So I think it's not the case. But if there is a camera, I think it would be good that letting people know that it's green or red (visual interface on the drone). If it is green, and it's recording, people will know that they are recorded."

Code: ● Privacy   Weight score: 0
I7 , 144 - 147
Created: 29-07-2024 10:49 by Analyst2, Modified: 02-09-2024 16:31 by Analyst1
Area: 570   2.22%

28.
"Maybe Could Have is something like blurring the face, if it is recording (Note: Blur the face or objects). It's easier for the privacy."

Code: ● Privacy   Weight score: 0
I7 , 155 - 155
Created: 07-08-2024 16:36 by Analyst1, Modified: 02-09-2024 16:31 by Analyst1
Area: 135   0.53%

29.
"ou want to know this as an announcement or displays?

Participant 43:43
Display is fine. I think if I'm the bystander and if it is announcing in a way that because if it lost its way, it's gonna announce like three to four times it gets annoyed by the other, not just to me but the rest of the people in the zone. So if it is just a name display, everyone can understand okay, this person or this drone has lost and looking for this person. So it can just check by, instead of getting annoyed. For example, if someone is playing speaker, when you are like working in the library or something, it annoys you."

Code: ● Privacy   Weight score: 0
I7 , 177 - 179
Created: 12-08-2024 14:32 by Analyst1, Modified: 02-09-2024 16:31 by Analyst1
Area: 608   2.37%

30.
"here's a tricky balance there that I don't actually want it to track too much of what's happening, but I do want it to be able to find me and check in with me that I am the recipient"

Code: ● Privacy   Weight score: 0
I6 , 57 - 57
Created: 29-07-2024 11:10 by Analyst2, Modified: 01-08-2024 15:55 by Analyst1
Area: 182   0.45%

31.
"So I'm thinking about the data privacy part, which is a bit tricky here because, when it comes to a snack, it doesn't matter that much who I am as a recipient. But when we're talking about emergency medicine, there's also this personal involvement in the product that is being delivered, so there's automatically a more personal link already to what is being delivered, which I have a feeling that I have an opinion about. What are those privacy-wise? But I'm not quite sure what that opinion is. Say that drone stops for the person next to me with my emergency medicine. That's something I would not want to happen. That's an interaction I don't want to see happening, especially when next to me, because I also don't want to have a system where we can, say, have people running up to the drone if they see their own, because

that's going to create chaos. So you want it to be smooth enough so that it actually tracks properly, but when we're talking about having that little check in and having that work smoothly enough for my emergency medicine, I think it would also take some time to actually know that I can trust this."

Code: ● Privacy Weight score: 0
I6 , 108 - 108
Created: 29-07-2024 11:20 by Analyst2, Modified: 01-08-2024 13:50 by Analyst1
Area: 1126 2.76%

32.

"Are you here for me? This should be done with. Ambience and with some privacy. Like I don't want my name to be on the drone. It's like you should not say I'm here for [paticipant says their name]. Like do everybody in the park know [name]. Like I don't want it to be like that."

Code: ● Privacy Weight score: 0
I5 , 90 - 90
Created: 25-07-2024 16:15 by Analyst1, Modified: 02-09-2024 16:02 by Analyst1
Area: 277 0.68%

33.

"people should not be able to see a camera like so in their face. And they feel like, oh, am I being recorded because that I feel like it's going to stir a lot of negative reactions. So because we don't want to hate the drones if we want to use them."

Code: ● Privacy Weight score: 0
I5 , 120 - 120
Created: 29-07-2024 11:49 by Analyst2, Modified: 02-09-2024 16:02 by Analyst1
Area: 249 0.61%

34.

"protect people's privacy even though they are having an emergency medical situation in the public space. They should also be given the choice of if they want to announce it or not."

Code: ● Privacy Weight score: 0
I5 , 171 - 171
Created: 29-07-2024 11:54 by Analyst2, Modified: 02-09-2024 16:02 by Analyst1
Area: 180 0.44%

35.

"I think it shouldn't have my personal information (Note: Private information - Names, condition, content of the delivery), I just don't know why, but I feel like it's going to happen. Like in an emergency situation, I just somehow imagine people would be like, well, it's a good idea to put the people's name and their condition on the voice announcement or something. So I want to like stress that over here. Okay, stop."

Code: ● Privacy Weight score: 0
I5 , 190 - 190
Created: 29-07-2024 11:56 by Analyst2, Modified: 02-09-2024 16:02 by Analyst1
Area: 421 1.03%

36.

"I also don't want it to have visible cameras (Note: Visible camera)"

Code: ● Privacy Weight score: 0
I5 , 210 - 210
Created: 29-07-2024 12:00 by Analyst2, Modified: 02-09-2024 16:02 by Analyst1
Area: 67 0.16%

37.

"in general, there's a bit of fear for surveillance. If we have these drones flying around everywhere, and the cameras are always on, I think that will be a bit tough for people or some people might even get aggressive. They will try to shoot the drone or whatever, or harm it."

Code: ● Privacy Weight score: 0

I4 , 103 - 103
Created: 29-07-2024 12:12 by Analyst2, Modified: 02-09-2024 15:58 by Analyst1
Area: 276   1.22%

38.
"on a broader level, some people might get aggressive with drones. It may be good if it has some indication for them."
Code: ● Privacy   Weight score: 0
I4 , 163 - 163
Created: 29-07-2024 12:16 by Analyst2, Modified: 02-09-2024 15:58 by Analyst1
Area: 116   0.51%

39.
"I wouldn't (want) to know who is the person because it's privacy. If that person just ordered the condom, for example, I don’t want to know if they are getting the condom or who is getting the condom. If the drone points out that person to everybody, it is not good."
Code: ● Privacy   Weight score: 0
I3 , 217 - 217
Created: 29-07-2024 12:29 by Analyst2, Modified: 02-09-2024 15:55 by Analyst1
Area: 266   1.46%

40.
"I'm not in some activity and didn't order something but I will get unsafe or confused when a drone staring at me or just following with me or (it) just stops there and looks at me. Definitely, every drone has a camera and because of these things, I need to know that this drone is not related to me or has some intentions to me."
Code: ● Privacy   Weight score: 0
I2 , 112 - 112
Created: 24-07-2024 14:34 by Analyst1, Modified: 02-09-2024 16:26 by Analyst1
Area: 328   1.39%

41.
"Not just stop in a weird way or for a very long time."
Code: ● Privacy   Weight score: 0
I2 , 136 - 136
Created: 24-07-2024 14:36 by Analyst1, Modified: 02-09-2024 16:26 by Analyst1
Area: 53   0.22%

42.
"If people just keep walking and the drone still stopped, they will also know that oh, he's doing some activity or is waiting for something. But this is just kind of a feeling like if you are interacting with a cat or with a dog, they just do not follow you. If you stand here for a very long time, the drone is coming (after you) and this gives a sense of (uncertain) feelings."
Code: ● Privacy   Weight score: 0
I2 , 140 - 140
Created: 24-07-2024 14:37 by Analyst1, Modified: 02-09-2024 16:26 by Analyst1
Area: 377   1.60%

43.
"what can it do? I am curious and it also concerns about privacy as drones flying above your head."
Code: ● Privacy   Weight score: 0
I1 , 158 - 158
Created: 24-07-2024 13:25 by Analyst1, Modified: 02-09-2024 16:29 by Analyst1
Area: 97   0.39%

2.4. Public space dynamics

1.

"It's especially the environment in which I picture myself to be sitting, because there is a lot going to be happening in that situation."

Code: ● Public space dynamics Weight score: 0
I6 , 41 - 41
Created: 25-07-2024 16:58 by Analyst1, Modified: 01-08-2024 13:50 by Analyst1
Area: 136 0.33%

2.4.1. Role of external agents

1.

"I'm playing some ball games with my friend on the grass"

Code: ● External agents Weight score: 0
I12 , 77 - 77
Created: 29-07-2024 12:05 by Analyst1, Modified: 02-09-2024 16:25 by Analyst1
Area: 55 0.26%

2.

"If some drone is approaching you, everyone gonna be in the park, even kids, and everyone's gonna look at you."

Code: ● External agents Weight score: 0
I7 , 19 - 19
Created: 29-07-2024 10:36 by Analyst2, Modified: 02-09-2024 16:31 by Analyst1
Area: 109 0.43%

3.

"I'm in a park, I'm imagining it's a great summer day. There's a ton of people around"

Code: ● External agents Weight score: 0
I6 , 53 - 53
Created: 25-07-2024 17:08 by Analyst1, Modified: 01-08-2024 13:50 by Analyst1
Area: 84 0.21%

4.

"have people running up to the drone if they see their own, because that's going to create chaos."

Code: ● External agents Weight score: 0
I6 , 108 - 108
Created: 25-07-2024 18:35 by Analyst1, Modified: 01-08-2024 13:50 by Analyst1
Area: 96 0.24%

5.

"I was having a nice picnic here."

Code: ● External agents Weight score: 0
I6 , 133 - 133
Created: 26-07-2024 11:22 by Analyst1, Modified: 01-08-2024 13:50 by Analyst1
Area: 32 0.08%

6.

"me and my friends are really starving and we're gonna celebrate because food is finally here."

Code: ● External agents Weight score: 0
I5 , 25 - 25
Created: 25-07-2024 16:05 by Analyst1, Modified: 02-09-2024 16:02 by Analyst1
Area: 93 0.23%

7.

"If people are like throwing Frisbees around it, around my location, I would feel a little worried too. I'm thinking, well, is the drone smart enough to dodge? Are they going to hit it? Should I talk to the people, like, wait, you guys want to pause for a second? Until I get my stuff or if there's like a dog running around. Yeah, generally these kind of things, I think

maybe my connection with the drone and the safety of it accomplishing the job. Yeah, those would be my main thoughts about uncertainty, I guess."

Code: ● External agents   Weight score: 0
I5 , 27 - 27
Created: 29-07-2024 11:43 by Analyst2, Modified: 02-09-2024 16:02 by Analyst1
Area: 515   1.26%

8.

"if there's like a dog running around."

Code: ● External agents   Weight score: 0
I5 , 27 - 27
Created: 25-07-2024 16:07 by Analyst1, Modified: 02-09-2024 16:02 by Analyst1
Area: 37   0.09%

9.

"if there's a bird flying, I think it should be able to respond to it."

Code: ● External agents   Weight score: 0
I5 , 120 - 120
Created: 25-07-2024 16:21 by Analyst1, Modified: 02-09-2024 16:02 by Analyst1
Area: 69   0.17%

10.

"there's a piece of flat land and there's no like tall grass to hurt your propellers or something. So land here and there is a crazy dog running around and there's like no dog poop on the floor that you drop my food on."

Code: ● External agents   Weight score: 0
I5 , 135 - 135
Created: 25-07-2024 16:24 by Analyst1, Modified: 02-09-2024 16:02 by Analyst1
Area: 218   0.53%

11.

"The real situation is much complex, either this person is alone or they have friends"

Code: ● External agents   Weight score: 0
I2 , 62 - 62
Created: 24-07-2024 14:23 by Analyst1, Modified: 02-09-2024 16:26 by Analyst1
Area: 84   0.36%

12.

"Because when you are building some kind of drone system in the lower sky of the city then there will be a lot of drones, and also birds, pigeons (in the sly), squirrels (on ground)."

Code: ● External agents   Weight score: 0
I2 , 64 - 64
Created: 29-07-2024 14:49 by Analyst2, Modified: 02-09-2024 16:26 by Analyst1
Area: 181   0.77%

13.

"if I'm a bystander, try to not hit me or there is a lot of people and surrounding buildings. They need to avoid all these things."

Code: ● External agents   Weight score: 0
I2 , 193 - 193
Created: 24-07-2024 14:42 by Analyst1, Modified: 02-09-2024 16:26 by Analyst1
Area: 129   0.55%

14.

"there could be so many bystanders in the park"

Code: ● External agents   Weight score: 0
I1 , 10 - 10
Created: 24-07-2024 12:22 by Analyst1, Modified: 02-09-2024 16:29 by Analyst1
Area: 45   0.18%

15.
"picnic with babies and families and like elders"
Code: ● External agents   Weight score: 0
I1 , 98 - 98
Created: 24-07-2024 13:11 by Analyst1, Modified: 02-09-2024 16:29 by Analyst1
Area: 47   0.19%

2.4.2. Environmental factors

1.
"When there's a food delivery, then you always (have someone) ring the (door) bell and then you know and you're sure that the delivery man knows where they're going, and then they deliver to you. Whereas, in this situation, the public space is open space. So when the drone approaches me, I'm still not sure if they are approaching me, or if they know they should deliver to me."
Code: ● Environmenal factors   Weight score: 0
I12 , 19 - 19
Created: 29-07-2024 11:54 by Analyst1, Modified: 02-09-2024 16:25 by Analyst1
Area: 377   1.77%

2.
"it might be a busy space. So it's like all cars on the road honking."
Code: ● Environmenal factors   Weight score: 0
I9 , 85 - 85
Created: 26-07-2024 14:36 by Analyst1, Modified: 08-08-2024 9:17 by Analyst1
Area: 68   0.26%

3.
"if we take a large, big park, and they have like different type of corners and you are stuck in one corner"
Code: ● Environmenal factors   Weight score: 0
I7 , 119 - 119
Created: 26-07-2024 12:03 by Analyst1, Modified: 02-09-2024 16:31 by Analyst1
Area: 106   0.41%

4.
"I like to sit in places where you have quite a bit of shade. And like maybe next to a bit of water or something. There's going to be needs to be some accuracy in how these drones are actually reaching me to let that go, right, because I can just imagine my snack coming up and I don't know, throwing, getting called on a branch, falling in the [names local river], and then I'm sitting there like: Well, I'm not getting that snack now."
Code: ● Environmenal factors   Weight score: 0
I6 , 86 - 86
Created: 25-07-2024 17:36 by Analyst1, Modified: 01-08-2024 13:50 by Analyst1
Area: 435   1.07%

5.
"there are trees and it's not going to bump into trees and you know drop the stuff"
Code: ● Environmenal factors   Weight score: 0
I5 , 12 - 12
Created: 25-07-2024 16:02 by Analyst1, Modified: 02-09-2024 16:02 by Analyst1
Area: 81   0.20%

6.
"I have to really put myself in this scenario, maybe there's a strong wind at the time? And I'll be thinking, who is it going to drop? Is that like the kind of uncertainty you're also looking for?"
Code: ● Environmenal factors   Weight score: 0
I5 , 26 - 26

Created: 29-07-2024 11:43 by Analyst2, Modified: 02-09-2024 16:02 by Analyst1
Area: 195 0.48%

7.
". Like, there's a piece of flat land and there's no like tall grass to hurt your propellers or something. So land here and there is a crazy dog running around and there's like no dog poop on the floor that you drop my food on. So that I feel like people definitely Should Have a say in because that's, I think, the kind of interaction that you wanna have with the drone."
Code: ● Environmenal factors Weight score: 0
I5 , 135 - 135
Created: 29-07-2024 11:51 by Analyst2, Modified: 02-09-2024 16:02 by Analyst1
Area: 370 0.91%

8.
"Because the propellers are so easily damaged to me, you know what I mean? Like, I've always seen them like, you know, a drop of rain. They're like, whoop."
Code: ● Environmenal factors Weight score: 0
I5 , 146 - 146
Created: 25-07-2024 16:26 by Analyst1, Modified: 02-09-2024 16:02 by Analyst1
Area: 154 0.38%

9.
"we can actually start looking, start preparing for their arrival because it's not at our home. If it's in our home, we can just stay chill and wait until it will arrive anyway, but it's in a public park."
Code: ● Environmenal factors Weight score: 0
I1 , 38 - 38
Created: 24-07-2024 12:51 by Analyst1, Modified: 02-09-2024 16:29 by Analyst1
Area: 203 0.82%

10.
"initially, we think of like, oh, it's better to pick the package in this location, but then in 5 minutes or nearby the time limit, we realized, oh, it's better we change the place due to changed dynamics in the park."
Code: ● Environmenal factors Weight score: 0
I1 , 138 - 138
Created: 24-07-2024 13:22 by Analyst1, Modified: 02-09-2024 16:29 by Analyst1
Area: 216 0.87%

2.4.3. Presence of multiple recipients and drones

1.
"It really depends. If there are multiple groups, then I'm like, Oh, who's it delivering to? Or if there's only one group there, I might be like, oh, they probably are delivered there. If we're talking about a scenario that everybody getting used to the delivery drone, I probably won't feel uncertain."
Code: ● Multiple groups Weight score: 0
I12 , 129 - 129
Created: 26-07-2024 13:07 by Analyst2, Modified: 02-09-2024 16:25 by Analyst1
Area: 301 1.41%

2.
"you will have multiple recipients everywhere and there is a possibility that you will receive the wrong package, if the drone identifies you to another person by mistake."
Code: ● Multiple groups Weight score: 0
I10 , 27 - 27
Created: 26-07-2024 15:08 by Analyst1, Modified: 02-09-2024 15:53 by Analyst1
Area: 170 0.77%

3.

"Even as a recipient, if I go to a scenario (where) there are many drones around, maybe just annoying or kind of weird to feel all the time that there is someone above your head. I'm telling you about the scenario that more people are expecting a delivery in a public park for example."

Code: ● Multiple groups   Weight score: 0
I8 , 30 - 30
Created: 26-07-2024 13:17 by Analyst1, Modified: 02-09-2024 16:30 by Analyst1
Area: 284   1.05%

4.

"In case there are many people ordering, I can hear many drones flying. (Imagine) we have three or four more (drones) coming right now, you (do not want to be) alerted all the time or (you want) to know that there gonna be some kind of drones flying around."

Code: ● Multiple groups   Weight score: 0
I8 , 78 - 78
Created: 26-07-2024 13:39 by Analyst1, Modified: 02-09-2024 16:30 by Analyst1
Area: 256   0.95%

5.

"If many people are gonna have deliveries in a busy day at the park, (it) is not going to be just one drone."

Code: ● Multiple groups   Weight score: 0
I8 , 150 - 150
Created: 26-07-2024 14:03 by Analyst1, Modified: 02-09-2024 16:30 by Analyst1
Area: 107   0.40%

6.

"I can imagine, however, if this is a more standard thing and there is a lot more drones flying around, then there would be a small bit of uncertainty"

Code: ● Multiple groups   Weight score: 0
I6 , 13 - 13
Created: 29-07-2024 10:56 by Analyst2, Modified: 01-08-2024 13:50 by Analyst1
Area: 149   0.37%

2.5. Differences in human roles

1.

"I will definitely feel more uncertain as compared to being a recipient."

Code: ● Human roles   Weight score: 0
I9 , 139 - 139
Created: 02-09-2024 15:45 by Analyst1, Modified: 02-09-2024 15:45 by Analyst1
Area: 71   0.27%

2.

"The main difference is in how actively they are engaging with the technology, so the bystanders is not making an active decision to engage with the technology, but it's still entering their space. Whereas a recipient made the decision to engage with that technology, so there's much more of an expected engagement in that."

Code: ● Human roles   Weight score: 0
I6 , 9 - 9
Created: 25-07-2024 16:52 by Analyst1, Modified: 01-08-2024 13:50 by Analyst1
Area: 322   0.79%

3.

"if you talk about the archetypes or possible use cases of a recipient, you can probably have like lists of possible use cases of possible archetypes. But for bystanders, you could have like, so many unpredictable variations, reactions and dynamics in the park."

Code: ● Human roles Weight score: 0
I1 , 10 - 10
Created: 24-07-2024 12:22 by Analyst1, Modified: 02-09-2024 16:29 by Analyst1
Area: 260 1.05%

2.5.1. Recipient

1.
"If the drone knows if I'm the recipient. If the drone knows where to go, basically, if there are certain about it."
Code: ● Recipient Weight score: 0
I12 , 15 - 15
Created: 29-07-2024 11:53 by Analyst1, Modified: 02-09-2024 16:25 by Analyst1
Area: 114 0.54%

2.
"As a recipient, I don't think so."
Code: ● Recipient Weight score: 0
I11 , 14 - 14
Created: 26-07-2024 13:43 by Analyst2, Modified: 08-08-2024 9:53 by Analyst1
Area: 33 0.16%

3.
"But as a recipient, I think like, okay, a drone will come so it's fine."
Code: ● Recipient Weight score: 0
I11 , 18 - 18
Created: 29-07-2024 11:26 by Analyst1, Modified: 08-08-2024 9:53 by Analyst1
Area: 71 0.35%

4.
"I wouldn't be uncertain as I am expecting it."
Code: ● Recipient Weight score: 0
I10 , 11 - 11
Created: 26-07-2024 15:06 by Analyst1, Modified: 02-09-2024 15:53 by Analyst1
Area: 45 0.20%

5.
"If the drone is not carrying anything, so I would think it is a personal drone. If it's approaching me, then probably (I am afraid that it is going to) shoot (a video of) me or it could be something from other people that I'm not expecting."
Code: ● Recipient Weight score: 0
I10 , 15 - 15
Created: 26-07-2024 15:07 by Analyst1, Modified: 02-09-2024 15:53 by Analyst1
Area: 240 1.09%

6.
"If the drone carries something then I will assume that it is like service drone, probably they also have the camera, but the purpose of the camera is not for shooting people, but just to guide his (the drone) own way. In that case, I won't feel that uncertainty."
Code: ● Recipient Weight score: 0
I10 , 19 - 19
Created: 26-07-2024 16:28 by Analyst2, Modified: 02-09-2024 15:53 by Analyst1
Area: 262 1.19%

7.
"I wouldn't feel uncertain as long as I know it's coming. I'm comparing it to an Uber driver delivery, like, I'm seeing it in the app and I know that this person is coming in five minutes. But otherwise, it could be anyone ringing the doorbell, right? It's the same way."
Code: ● Recipient Weight score: 0
I9 , 11 - 11

Created: 29-07-2024 8:15 by Analyst2, Modified: 08-08-2024 9:17 by Analyst1
Area: 269 1.02%

8.
"So I think it (uncertainty) is something related to the size of it"
Code: ● Recipient Weight score: 0
I9 , 77 - 77
Created: 29-07-2024 8:49 by Analyst2, Modified: 08-08-2024 9:17 by Analyst1
Area: 66 0.25%

9.
"If I am in a park, and just see drones flying around, maybe I'm not gonna be sure if that's going to be my package or if I have to go to stand somewhere specifically (to receive the package)."
Code: ● Recipient Weight score: 0
I8 , 18 - 18
Created: 26-07-2024 13:14 by Analyst1, Modified: 02-09-2024 16:30 by Analyst1
Area: 191 0.71%

10.
"No, because I do know in prior that I'm the recipient, so I know the info that it is approaching me, so I don't feel uncertain."
Code: ● Recipient Weight score: 0
I7 , 11 - 11
Created: 29-07-2024 10:35 by Analyst2, Modified: 02-09-2024 16:31 by Analyst1
Area: 127 0.50%

11.
"If it is a crowded area, and if it attracts huge attention. I'm a little bit introvert, it gives me uncertainty feeling if people are looking at me."
Code: ● Recipient Weight score: 0
I7 , 15 - 15
Created: 26-07-2024 11:46 by Analyst1, Modified: 02-09-2024 16:31 by Analyst1
Area: 148 0.58%

12.
"00:01:25 Participant:
Not necessarily uncertainty. Especially not considering in this story. I just made an order to be delivered by drone."
Code: ● Recipient Weight score: 0
I6 , 12 - 13
Created: 29-07-2024 10:56 by Analyst2, Modified: 01-08-2024 13:50 by Analyst1
Area: 139 0.34%

13.
"is this my delivery or is this drone heading to someone else who's also in this park somewhere in my near vicinity? So there's some uncertainty there."
Code: ● Recipient Weight score: 0
I6 , 13 - 13
Created: 01-08-2024 9:45 by Analyst1, Modified: 01-08-2024 13:50 by Analyst1
Area: 150 0.37%

14.
"I'm going to look for clues of like, it's just going to be a general box. Like, does everybody get the same size of a box? I was thinking about how much I ordered and how big of a box is"
Code: ● Recipient Weight score: 0
I5 , 21 - 21
Created: 01-08-2024 9:51 by Analyst1, Modified: 02-09-2024 16:02 by Analyst1
Area: 186 0.46%

15.
"I will feel uncertain more about my connection with the drone. Like is it for me or is it not?"

Code: ● Recipient   Weight score: 0
I5 , 21 - 21
Created: 01-08-2024 9:50 by Analyst1, Modified: 02-09-2024 16:02 by Analyst1
Area: 94   0.23%

16.
"my connection with the drone"

Code: ● Recipient   Weight score: 0
I5 , 27 - 27
Created: 25-07-2024 16:08 by Analyst1, Modified: 02-09-2024 16:02 by Analyst1
Area: 28   0.07%

17.
"Yeah, I would (feel uncertain). I'm imagining that's just a drone with a (package) box. I would then be a bit uncertain if the package is really for me, or maybe for someone else. That will make me uncertain. I think what will also play a role is the fact that, as far as I know, drones make quite a bit of noise. I think that's something more maybe emotional or more primal, but that wouldn't help with making me feel like safe or like comfortable with situation. At least not the first times (very begining stages)."

Code: ● Recipient   Weight score: 0
I4 , 11 - 11
Created: 29-07-2024 12:04 by Analyst2, Modified: 02-09-2024 15:58 by Analyst1
Area: 517   2.29%

18.
"drones make quite a bit of noise. I think that's something more maybe emotional or more primal, but that wouldn't help with making me feel like safe or like comfortable with situation."

Code: ● Recipient   Weight score: 0
I4 , 11 - 11
Created: 25-07-2024 11:34 by Analyst1, Modified: 02-09-2024 15:58 by Analyst1
Area: 184   0.81%

19.
"If the delivery goes well? Like if it approaches and drops the thing from the air, if it will put it in my hand? Those things would also be a bit uncertain for me."

Code: ● Recipient   Weight score: 0
I4 , 19 - 19
Created: 25-07-2024 11:38 by Analyst1, Modified: 02-09-2024 15:58 by Analyst1
Area: 163   0.72%

20.
"That would also make me a bit uncertain. How does it know where I am? How is it gonna go well?"

Code: ● Recipient   Weight score: 0
I4 , 23 - 23
Created: 25-07-2024 11:40 by Analyst1, Modified: 02-09-2024 15:58 by Analyst1
Area: 94   0.42%

21.
"Participant 01:44
I will feel certain, why I wouldn't feel so. I don't understand.

Analyst1 01:52
And why would you feel certain?

Participant 01:55
Because I know it will come. And it comes. So I feel certain."

Code: ● Recipient Weight score: 0
I3 , 10 - 15
Created: 29-07-2024 12:18 by Analyst2, Modified: 02-09-2024 15:55 by Analyst1
Area: 226 1.24%

22.

"It depends. So if I have this activity, and I know there is a drone coming for me, and when I saw there is drone coming to me, I might know that, Oh, it's my activity, or it's my service. So it is less uncertainty, or less unsafety.

Analyst1 00:56
ut do you still feel uncertainty? Or are you do not feel uncertainty at all?

Participant 01:01
On a relative score of one to ten, ten being high, I think the uncertainty will at least be for one or two.

Analyst1 01:12
And why, like even that one or two, what factors make you feel uncertain?

Participant 01:18
Because it is kind of a machine and yet, you know, the drones can do a lot of things. When the wings are exposed to the air, and for example, like, a small helicopter, it might create some unexpected activities. So this uncertainty could bring this unsafety."

Code: ● Recipient Weight score: 0
I2 , 10 - 18
Created: 29-07-2024 14:41 by Analyst2, Modified: 02-09-2024 16:26 by Analyst1
Area: 848 3.59%

23.

"On a relative score of one to ten, ten being high, I think the uncertainty will at least be for one or two."

Code: ● Recipient Weight score: 0
I2 , 14 - 14
Created: 24-07-2024 13:34 by Analyst1, Modified: 02-09-2024 16:26 by Analyst1
Area: 107 0.45%

24.

"the recipient is, I think, it's quite clear. Someone who expect a drone coming to the park. He or she, or they, can already expect something. Or I assume, he is already aware or know what kind of thing a drone is, like, how they get onto the ground, how they fly, that kind of thing. So I imagine that they already have some knowledge about how drone works, how they land, how they take off, that kind of thing."

Code: ● Recipient Weight score: 0
I1 , 10 - 10
Created: 24-07-2024 12:20 by Analyst1, Modified: 02-09-2024 16:29 by Analyst1
Area: 411 1.65%

25.

"they already know that drone will come. What to do when the drone actually landed? And how can I do that? Is it the right time to, like, approach the drone? Should I keep some distance to the drones? What happened when the drones failed to land? That kind of thing makes me feel uncertainty."

Code: ● Recipient Weight score: 0
I1 , 10 - 10
Created: 24-07-2024 12:22 by Analyst1, Modified: 02-09-2024 16:29 by Analyst1
Area: 291 1.17%

26.
"Yeah, I guess. As I said, maybe if I know what kind of drones will arrive, how big? Will they actually land? Do they just throw my snacks around the sky and say bye? Do they actually fly around or hover like, two meters from the ground? Do I need to like, reach the packet or do they actually need to land on the grass? That kind of uncertainty. When the drone actually approaching the recipient, is there space and where do we actually meet? Because I am wondering how it will be? Like a helicopter and we have a helipad, we already know the helicopter will land on that particular spot. But for drones in the park, where should I wait exactly? Or is it dynamic, case by case?"
Code: ● Recipient Weight score: 0
I1 , 14 - 14
Created: 29-07-2024 15:25 by Analyst2, Modified: 02-09-2024 16:29 by Analyst1
Area: 677 2.73%

27.
"is there space and where do we actually meet?"
Code: ● Recipient Weight score: 0
I1 , 14 - 14
Created: 24-07-2024 12:36 by Analyst1, Modified: 02-09-2024 16:29 by Analyst1
Area: 45 0.18%

28.
"for drones in the park, where should I wait exactly? Or is it dynamic, case by case?"
Code: ● Recipient Weight score: 0
I1 , 14 - 14
Created: 24-07-2024 12:37 by Analyst1, Modified: 02-09-2024 16:29 by Analyst1
Area: 84 0.34%

2.5.2. Bystander

1.
"Yeah. So they're sitting there, right? Yeah, then probably."
Code: ● Bystander Weight score: 0
I12 , 129 - 129
Created: 26-07-2024 13:07 by Analyst2, Modified: 02-09-2024 16:25 by Analyst1
Area: 59 0.28%

2.
"Yes. As a bystander, I would feel uncertain about a drone approaches."
Code: ● Bystander Weight score: 0
I11 , 126 - 126
Created: 26-07-2024 16:11 by Analyst2, Modified: 08-08-2024 9:53 by Analyst1
Area: 69 0.34%

3.
"It is related to the shape of the drone and the noise. The noise is like a small airplane. I don't know how it will behave: what is this? It's a machine next to me, and I don't know who is driving it. I think the noise and also the shape of the drone would make me feel a bit uncertain and scary about it."
Code: ● Bystander Weight score: 0
I11 , 130 - 130
Created: 29-07-2024 11:44 by Analyst1, Modified: 08-08-2024 9:53 by Analyst1
Area: 305 1.52%

4.
"Nobody can see it (drone) because it's something small. I don't know how the machine will behave."
Code: ● Bystander Weight score: 0
I11 , 134 - 134

Created: 29-07-2024 11:44 by Analyst1, Modified: 08-08-2024 9:53 by Analyst1
Area: 97   0.48%

5.

"know that a lot of people when they see a new thing they want to take picture and they are unconsciously getting into a dangerous area because they are getting too close."

Code: ● Bystander   Weight score: 0
I10 , 115 - 115
Created: 26-07-2024 15:30 by Analyst1, Modified: 02-09-2024 16:23 by Analyst1
Area: 170   0.77%

6.

"I'm in a scenario where the recipient is not nearby and somehow they just left their phone here, and went to their friends or bathroom for example, and the drone comes over. But nobody is confirming and everybody is wondering like who ordered this."

Code: ● Bystander   Weight score: 0
I10 , 125 - 125
Created: 26-07-2024 15:33 by Analyst1, Modified: 02-09-2024 16:23 by Analyst1
Area: 248   1.13%

7.

"Even now, when you're on a beach or someplace, you can hear a drone around you. So you are able to recognize that it's a drone. As long as it's away from you, it's okay. But if it starts coming down, then you're also looking at it more often than being like, Okay, where is it dropping by? Or, like, you know, what's happening? So that will have happened even more if there is a piece of delivery that it is holding."

Code: ● Bystander   Weight score: 0
I9 , 139 - 139
Created: 29-07-2024 9:03 by Analyst2, Modified: 02-09-2024 15:45 by Analyst1
Area: 416   1.58%

8.

"something else that also makes you are uncertain?

Participant 34:14
About the delivery location also. Where, when and how is it going to place the parcel, like drop it off."

Code: ● Bystander   Weight score: 0
I9 , 141 - 143
Created: 26-07-2024 14:53 by Analyst1, Modified: 08-08-2024 9:17 by Analyst1
Area: 174   0.66%

9.

"The bystander is that while you're enjoying your time, you could just hear the noise of the drone, or I don't know how big or small a drone could be, or what kind of packages (it) start(s) dropping, or do you know, the drone, it's coming close to the ground, or if it is just dropping something from top."

Code: ● Bystander   Weight score: 0
I8 , 10 - 10
Created: 26-07-2024 13:12 by Analyst1, Modified: 02-09-2024 16:30 by Analyst1
Area: 304   1.12%

10.

"As a bystander, most of the times, I would be annoyed from the sound or, how high something drops down."

Code: ● Bystander   Weight score: 0
I8 , 10 - 10
Created: 26-07-2024 13:12 by Analyst1, Modified: 02-09-2024 16:30 by Analyst1
Area: 103   0.38%

11.
"Yeah, I would not like that. I would have uncertainty because I don't know who's controlling it. At least the experience I have so far, just by being in a park or being on a hike and hear the sound EZZZZ around. Sometimes in the beginning, it's like, is there a mosquito? Or is there something? and then you see a drone flying around."

Code: ● Bystander   Weight score: 0
I8 , 136 - 136
Created: 29-07-2024 10:25 by Analyst2, Modified: 02-09-2024 16:30 by Analyst1
Area: 334   1.23%

12.
"every time I'm thinking about the people who want to damage things."

Code: ● Bystander   Weight score: 0
I8 , 170 - 170
Created: 26-07-2024 14:08 by Analyst1, Modified: 02-09-2024 16:30 by Analyst1
Area: 67   0.25%

13.
"if I'm like bystander, I feel like something is arriving for someone. In the current situation, delivering with a drone is not completely obvious in the mind or something."

Code: ● Bystander   Weight score: 0
I7 , 129 - 129
Created: 29-07-2024 10:47 by Analyst2, Modified: 02-09-2024 16:31 by Analyst1
Area: 171   0.67%

14.
"I'm a bystander sitting in the park and first I'm going to hear this sound, you know, because I can hear this drone approaching, and I'm going to be looking around because that's one thing we tend to do if we're sitting somewhere and we hear a sound, we try to locate the sounds. So okay, I'm already distracted."

Code: ● Bystander   Weight score: 0
I6 , 133 - 133
Created: 26-07-2024 11:22 by Analyst1, Modified: 01-08-2024 13:50 by Analyst1
Area: 312   0.76%

15.
"I'm looking around and seeing what's going on and I spot a drone. I'm like: what is it drone doing here? I'm sitting here in the park. There's no one I would check: Okay, there's no one actively controlling it, it seems because everyone is just also either in their own group, or also looking at this drone trying to figure out what's happening. And then it actually approaches a specific group of people."

Code: ● Bystander   Weight score: 0
I6 , 133 - 133
Created: 26-07-2024 11:23 by Analyst1, Modified: 01-08-2024 13:50 by Analyst1
Area: 405   0.99%

16.
"hose different companies start showing up and you're like: Oh, god, the sound I can't even focus anymore here I'm trying to enjoy a picnic."

Code: ● Bystander   Weight score: 0
I6 , 133 - 133
Created: 26-07-2024 11:24 by Analyst1, Modified: 01-08-2024 13:50 by Analyst1
Area: 139   0.34%

17.
"I'm a bystander that I know that I haven't ordered anything, then the first I think reaction is probably thinking, OK, what's the purpose of this drone?"

Code: ● Bystander   Weight score: 0
I5 , 11 - 11

Created: 25-07-2024 16:01 by Analyst1, Modified: 02-09-2024 16:02 by Analyst1
Area: 152 0.37%

18.
"Yeah. Yeah, like I said, it's just, I'm curious about this purpose."
Code: ● Bystander Weight score: 0
I5 , 196 - 196
Created: 29-07-2024 11:57 by Analyst2, Modified: 02-09-2024 16:02 by Analyst1
Area: 67 0.16%

19.
"Not really (uncertain), if it would be just the drone. If it would have a package attached to it, not really either. If it would have a live camera on it, I would feel a bit watched, a bit uneasy, maybe because I'm being filmed, or whatever. But I would feel a lot less uncertain than if I have ordered something and I want it to go well."
Code: ● Bystander Weight score: 0
I4 , 127 - 127
Created: 29-07-2024 12:14 by Analyst2, Modified: 02-09-2024 15:58 by Analyst1
Area: 338 1.49%

20.
"Analyst1 20:49
Okay, thanks. So now we complete the questions for the role of recipient. Now we go for the same kind of questions, but for the role of bystander. So before we go to this part, the question I have is, would you or would you not feel so uncertain, when a delivery drone approaches you when you're as a bystander?

Participant 21:12
No.

Analyst1 21:16
Why? Why do you not feel uncertain? Or why do you feel certain?

Participant 21:23
It's always controlled by some people. It's quiet reliable, so far the technology. The drone I have experienced is always for photo for something, it's reliable technology."
Code: ● Bystander Weight score: 0
I3 , 128 - 135
Created: 29-07-2024 12:24 by Analyst2, Modified: 02-09-2024 15:55 by Analyst1
Area: 649 3.56%

21.
"Yes, definitely because I know I'm not doing something and I know I did not order some service or require some demand. So if the drone was approaching near me, definitely, I think I will feel uncertain and unsafe.

Analyst1 31:47
On a scale of one to ten how uncertain would you feel for this? Why?

Participant 31:53
Eight

Analyst1 31:55
And what factors make you feel uncertain?

Participant 31:59
Well, because I don't know why it is approaching near me and because I know I'm not doing

something or involved in some activity or something but the drone approaches to me. I don't know what it is going to do to me."

Code: ● Bystander   Weight score: 0
I2 , 80 - 88
Created: 29-07-2024 14:51 by Analyst2, Modified: 02-09-2024 16:26 by Analyst1
Area: 646   2.74%

22.

"On a scale of one to ten how uncertain would you feel for this? Why?

Participant 31:53
Eight"

Code: ● Bystander   Weight score: 0
I2 , 82 - 84
Created: 24-07-2024 14:29 by Analyst1, Modified: 02-09-2024 16:26 by Analyst1
Area: 94   0.40%

23.

"I don't know why it is approaching near me and because I know I'm not doing something or involved in some activity or something but the drone approaches to me. I don't know what it is going to do to me."

Code: ● Bystander   Weight score: 0
I2 , 88 - 88
Created: 24-07-2024 14:30 by Analyst1, Modified: 02-09-2024 16:26 by Analyst1
Area: 202   0.86%

24.

"I think the unsafety is the first one. Second, I become confused. I'm just trying to figure out why it is coming and if I walk away, will it follow me?"

Code: ● Bystander   Weight score: 0
I2 , 92 - 92
Created: 24-07-2024 14:30 by Analyst1, Modified: 02-09-2024 16:26 by Analyst1
Area: 151   0.64%

25.

"You could think of all what is this a spy drone? Or military drone? Or what? This is my privacy. Do they record video? Take photo of my leisure time in the park? Or who places this drone in this park?"

Code: ● Bystander   Weight score: 0
I1 , 10 - 10
Created: 24-07-2024 12:22 by Analyst1, Modified: 02-09-2024 16:29 by Analyst1
Area: 200   0.81%

26.

"Why is their a drone in this park right now like without really assuming. There's a drone like any other drone, it's a flying object not UFO but it is a flying object with sounds in the park like what? What this thing is doing on the park? There could be so many reactions from me as a bystander."

Code: ● Bystander   Weight score: 0
I1 , 146 - 146
Created: 24-07-2024 13:23 by Analyst1, Modified: 02-09-2024 16:29 by Analyst1
Area: 296   1.19%

27.

"I wouldn't know why is it there and I think it shouldn't be there when you are enjoying your time and because you don't always see a delivery drone when you are laying out on the grass. Then you see whoo (imitating sound of drone) What's this? I think you'll be quite surprised, curious, irritated or feel something seems unnatural"

Code: ● Bystander   Weight score: 0

I1 , 150 - 150
Created: 24-07-2024 13:24 by Analyst1, Modified: 02-09-2024 16:29 by Analyst1
Area: 331   1.33%

3. User requirements to feel certain during HDI

1.

"I think drones are in general still quite dangerous so you want to make it as safe as possible. So I probably put some requirements"

Code: ● User requirements Weight score: 0
I10 , 59 - 59
Created: 26-07-2024 15:15 by Analyst1, Modified: 02-09-2024 16:23 by Analyst1
Area: 131 0.60%

2.

"As a person I have a lot of anxiety. I would like to know some stuff beforehand."

Code: ● User requirements Weight score: 0
I8 , 42 - 42
Created: 26-07-2024 13:20 by Analyst1, Modified: 02-09-2024 16:30 by Analyst1
Area: 80 0.30%

3.

"The first thing that I would like to know is for safety"

Code: ● User requirements Weight score: 0
I8 , 42 - 42
Created: 26-07-2024 13:21 by Analyst1, Modified: 02-09-2024 16:30 by Analyst1
Area: 55 0.20%

4.

"also comes what can you expect from it and what is actually known and said."

Code: ● User requirements Weight score: 0
I6 , 17 - 17
Created: 25-07-2024 16:56 by Analyst1, Modified: 01-08-2024 13:50 by Analyst1
Area: 75 0.18%

5.

"I can expect, right, set up my expectation."

Code: ● User requirements Weight score: 0
I3 , 36 - 36
Created: 24-07-2024 14:46 by Analyst1, Modified: 02-09-2024 15:55 by Analyst1
Area: 43 0.24%

3.1. Tracking information for the recipient

1.

"For emergency medicine, I am totally waiting. [Brainstorming.] I probably saw it's coming, it could give me a notification (Note: notification when they arrive)."

Code: ● Tracking information Weight score: 0
I12 , 105 - 105
Created: 29-07-2024 12:07 by Analyst1, Modified: 02-09-2024 16:25 by Analyst1
Area: 161 0.76%

2.

"everyone knows even our Zomato (Indian Food Delivery App) everything will be using maps and you see its gonna deliver at a certain time. The delivery person (regarding delivery method) is the same, like you get a notification when it's nearby or something."

Code: ● Tracking information Weight score: 0
I7 , 39 - 39
Created: 26-07-2024 11:51 by Analyst1, Modified: 02-09-2024 16:31 by Analyst1
Area: 256 1.00%

3.

"Those are for the expectation, and they will feel more transparent, if they have those information."

Code: ● Tracking information Weight score: 0
I3 , 44 - 44
Created: 24-07-2024 14:48 by Analyst1, Modified: 02-09-2024 15:55 by Analyst1
Area: 99 0.54%

3.1.1. Precision

1.

"This is interesting question because normally you know it (the delivery) will arrive, like in the normal delivery app. If I compare this (drone delivery) scenario to a normal delivery app, they normally will tell you like within 30 minutes or like UberEats will give you a countdown. But to be honest, it is also normal that they don't make it before the countdown. So I really want to know that there's map tracking. So I can see where the drone is, where it flies to on the map, to feel fully in control of the drone."

Code: ● Precision Weight score: 0
I10 , 91 - 91
Created: 26-07-2024 16:46 by Analyst2, Modified: 02-09-2024 16:23 by Analyst1
Area: 519 2.36%

2.

"That's the difference between a Must Have and Should Have that I do not know exactly. It should indicate some sort of a timescale on a larger context in the Must Have (Note: expected delivery duration 20-30 minutes). Like the context of being a part of a group in the garden, I know that, okay, I've ordered something, which is going to arrive in 20 minutes from now. So that is a Must Have, like how on UberEATS, you have expected time of delivery between 20 to 30 minutes. It would be nice to have a more precise one when it's really approaching (close). When you see the delivery guy like driving to your house, of course, it is not precisely real time. There is, I believe, some offset there, but it gives an indication of when and where they are (Note: real-time delivery status). That is a Should Have."

Code: ● Precision Weight score: 0
I9 , 39 - 39
Created: 29-07-2024 8:43 by Analyst2, Modified: 09-08-2024 14:49 by Analyst1
Area: 808 3.08%

3.

"if I know the time that it's arriving, if it's like precise, I'm fine"

Code: ● Precision Weight score: 0
I8 , 58 - 58
Created: 26-07-2024 13:29 by Analyst1, Modified: 02-09-2024 16:30 by Analyst1
Area: 69 0.25%

4.

"For the drone traffic (Note: drone air traffic), I'm not so sure. The real time location (Note: real time location), if I go for the scenario of I'm really sick and sometimes the estimation time, at least from the Thuisbezorgd, is always wrong. It says it can arrive (in some time) and then you listen to (update time). So, I would like to have the accuracy. If they say that I'm going to be in three minutes, I'm gonna know that in three minutes I have to leave my bed to go out and get it. I wouldn't like to have to wait. I would prefer the drone to wait for me than for me to have to wait for the drone."

Code: ● Precision Weight score: 0
I8 , 120 - 120
Created: 29-07-2024 10:00 by Analyst2, Modified: 02-09-2024 16:30 by Analyst1
Area: 607 2.24%

5.

"I want it to be like, really precise, so that if it is like an emergency, and if there are no people around me, I can hold up, okay, the drone or the medication will be arrived in this time."

Code: ● Precision Weight score: 0
I7 , 111 - 111
Created: 26-07-2024 12:00 by Analyst1, Modified: 02-09-2024 16:31 by Analyst1
Area: 190 0.74%

6.

"I don't want pop up notifications every time the drone moves a minute closer to me. Let's stay away from that. But it's nice to have a little place to do check in. For example, the time estimation or GPS (Note: time estimation), whichever one of the two would be more accurate then that case would fit better, but having some form of tracking that I can just refresh the web page and get an update in there and also feel like it is actually giving me an update, that would be nice."

Code: ● Precision Weight score: 0
I6 , 66 - 66
Created: 25-07-2024 17:26 by Analyst1, Modified: 01-08-2024 14:25 by Analyst1
Area: 481 1.18%

7.

"As long as I know that it will come and as well come between a reasonable timeframe, I wouldn't go that much to know it on a minute."

Code: ● Precision Weight score: 0
I4 , 87 - 87
Created: 25-07-2024 12:11 by Analyst1, Modified: 02-09-2024 15:58 by Analyst1
Area: 132 0.58%

8.

"As long as you know, like, oh, it will come in half an hour. I don't really mind if it's like 20% on the way or 40% on the way."

Code: ● Precision Weight score: 0
I4 , 95 - 95
Created: 25-07-2024 12:24 by Analyst1, Modified: 02-09-2024 15:58 by Analyst1
Area: 127 0.56%

9.

"If it's emergency, it changes a bit. I think it is probably crucial to know the exact delivery time"

Code: ● Precision Weight score: 0
I4 , 115 - 115
Created: 29-07-2024 12:12 by Analyst2, Modified: 02-09-2024 15:58 by Analyst1
Area: 99 0.44%

10.

"In this scenario, I think there should be a big emphasis on speed because we're dealing with emergency here. The recipient must have a sense of when it gets delivered, so they can act accordingly (Note: Exact delivery time or very small window of delivery)"

Code: ● Precision Weight score: 0
I4 , 119 - 119
Created: 29-07-2024 12:13 by Analyst2, Modified: 02-09-2024 15:58 by Analyst1
Area: 256 1.13%

11.

"This is more important package, it saves life, maybe. So I want to know very precise, how fast it can deliver."

Code: ● Precision Weight score: 0
I3 , 101 - 101
Created: 29-07-2024 12:22 by Analyst2, Modified: 02-09-2024 15:55 by Analyst1
Area: 110 0.60%

12.
"Because it's emergency and time like seconds count. So yeah, I think the precision (of delivery time and updates) is even more important. So I think this is only about the precision about time"
Code: ● Precision Weight score: 0
I1 , 130 - 130
Created: 29-07-2024 15:33 by Analyst2, Modified: 02-09-2024 16:29 by Analyst1
Area: 192 0.77%

3.1.2. Time details

1.
"There should be notification that it arrives (Note: notification when they arrive)."
Code: ● Time details Weight score: 0
I12 , 37 - 37
Created: 29-07-2024 11:57 by Analyst1, Modified: 02-09-2024 16:25 by Analyst1
Area: 83 0.39%

2.
"I can also imagine, if it is emergency medicine, when you order it, there should already be an estimated time."
Code: ● Time details Weight score: 0
I12 , 121 - 121
Created: 26-07-2024 13:06 by Analyst2, Modified: 02-09-2024 16:25 by Analyst1
Area: 110 0.52%

3.
"As it is some medicine, I would like to know if it will be faster (Note: Time to deliver) than me going to the pharmacy. It would be very interesting to have it."
Code: ● Time details Weight score: 0
I11 , 98 - 98
Created: 26-07-2024 16:09 by Analyst2, Modified: 08-08-2024 9:53 by Analyst1
Area: 161 0.80%

4.
"When it's landed, he should tell me when I can get the stuff (package) (Note: tell me when I can get the goods)."
Code: ● Time details Weight score: 0
I10 , 35 - 35
Created: 26-07-2024 15:11 by Analyst1, Modified: 02-09-2024 16:23 by Analyst1
Area: 112 0.51%

5.
"I think, one thing that is really important, is when it (the drone) will arrive (Note: when it will arrive (countdown/map tracking))"
Code: ● Time details Weight score: 0
I10 , 87 - 87
Created: 26-07-2024 16:44 by Analyst2, Modified: 02-09-2024 16:23 by Analyst1
Area: 132 0.60%

6.
"I wouldn't feel uncertain as long as I know it's coming."
Code: ● Time details Weight score: 0
I9 , 11 - 11
Created: 26-07-2024 14:23 by Analyst1, Modified: 08-08-2024 9:17 by Analyst1
Area: 56 0.21%

7.
"Mainly like some sort of a update on delivery time (Note: update on delivery time)."

Code: ● Time details   Weight score: 0
I9 , 27 - 27
Created: 29-07-2024 8:42 by Analyst2, Modified: 08-08-2024 9:17 by Analyst1
Area: 83   0.32%

8.
"I know when to expect the delivery. It is generally less to do with a drone, but more to do with the delivery time. It just ensures that I know when to expect the delivery with the drone."
Code: ● Time details   Weight score: 0
I9 , 35 - 35
Created: 29-07-2024 8:42 by Analyst2, Modified: 08-08-2024 9:17 by Analyst1
Area: 187   0.71%

9.
"I definitely need some more context on the delivery status because of this scenario like emergency medicine. I think time plays a very critical role there. So, a real time update is required."
Code: ● Time details   Weight score: 0
I9 , 115 - 115
Created: 29-07-2024 8:58 by Analyst2, Modified: 08-08-2024 9:17 by Analyst1
Area: 191   0.73%

10.
"For the rest of the things [however, does not answer the reasoning for all], in case if I was waiting for medicine, I would like for sure to know when it is arriving (Note: Notification that the drone is arriving) and where it is located. If I'm really sick and I need to do something last minute and now I have to go to the balcony or to the park whatever to receive the package for example."
Code: ● Time details   Weight score: 0
I8 , 112 - 112
Created: 29-07-2024 9:58 by Analyst2, Modified: 02-09-2024 16:30 by Analyst1
Area: 392   1.45%

11.
"Because if I need something, and it is getting delivered from nearby pharmacy or something, how long it gonna take. [Instructed to use post it]. (Note: Delivery timing)."
Code: ● Time details   Weight score: 0
I7 , 91 - 91
Created: 29-07-2024 10:42 by Analyst2, Modified: 02-09-2024 16:31 by Analyst1
Area: 169   0.66%

12.
"Analyst1 24:31
And what about delivery time?

Participant 24:34
Actually the same thing. It needs to be picked right away from the nearest one. Because as medicines are sometimes the emergency case.

Analyst1 24:46
How do you want to know this information?

Participant 24:50
Just like pointing out in the app and saying, Okay, you are in this location (and the nearest pharmacy is) 700 meters or 500 meters away and I chose the 500 meters because I found this one easier to approach you in a couple of moments.

Analyst1 25:08

How does it make you feel knowing that information compared to the delivery time kind of information in the snacks, like how precise you want it to be?

Participant 25:18
I want it to be like, really precise, so that if it is like an emergency, and if there are no people around me, I can hold up, okay, the drone or the medication will be arrived in this time. Instead, for example, if we call 911 or some emergency ambulance, it's gonna take some time. If I get the first aid in prior to the ambulance, it can make me feel ease, it can also sometimes save their lives, not just me, or any other person."

Code: ● Time details Weight score: 0
I7 , 100 - 111
Created: 29-07-2024 10:43 by Analyst2, Modified: 02-09-2024 16:31 by Analyst1
Area: 1174 4.58%

13.

"Note4: Estimated delivery time"

Code: ● Time details Weight score: 0
I4 , 81 - 81
Created: 07-08-2024 16:34 by Analyst1, Modified: 02-09-2024 15:58 by Analyst1
Area: 30 0.13%

14.

"If it's emergency, it changes a bit. I think it is probably crucial to know the exact delivery time. [Participant brainstorming]"

Code: ● Time details Weight score: 0
I4 , 115 - 115
Created: 29-07-2024 12:12 by Analyst2, Modified: 02-09-2024 15:58 by Analyst1
Area: 128 0.57%

15.

"In this scenario, I think there should be a big emphasis on speed because we're dealing with emergency here. The recipient must have a sense of when it gets delivered, so they can act accordingly (Note: Exact delivery time or very small window of delivery)"

Code: ● Time details Weight score: 0
I4 , 119 - 119
Created: 29-07-2024 12:13 by Analyst2, Modified: 02-09-2024 15:58 by Analyst1
Area: 256 1.13%

16.

"Participant 02:12
At what time it will arrive, for example."

Code: ● Time details Weight score: 0
I3 , 18 - 19
Created: 29-07-2024 12:18 by Analyst2, Modified: 02-09-2024 15:55 by Analyst1
Area: 60 0.33%

17.

"Participant 05:29
The estimated arrival time (Note: EAT (personal mobile device for all)), and the dropping location (Note: dropping location), where it's gonna land. And how the drone look like (Note: how it look like).

Analyst1 05:49
Why do you need this information?

Participant 05:54
I mean, I can expect, right, set up my expectation. Also, if I imagine we'll have a lot of drones flying through the sky, then I want to know which one is mine. And Should Have is not a

Must. I can track on my phone or the device, how far it is and where it flies, the current location of the delivery (Note: the current location track). I would expect more. It's like the delivery guy and I can say where he is now.

Analyst1 06:41
Yes. And like, what does it make you feel like when you receive this kind of information?

Participant 06:48
What do you mean?

Analyst1 06:51
So you are mentioning these different kinds of information, right? You're also explaining why you need it. So what does it make you feel if you have this information, or if you don't have this information?

Participant 07:07
Those are for the expectation, and they will feel more transparent, if they have those information."

Code: ● Time details   Weight score: 0
I3 , 31 - 44
Created: 29-07-2024 12:19 by Analyst2, Modified: 02-09-2024 15:55 by Analyst1
Area: 1233   6.76%

18.

"It's urgent, I need to know where it flies. Otherwise, I can think about the other solution. Or do I need to have an alternative plan? I can call the ambulance somewhere else, right. If the drone comes out? Yeah, so basically all the information matters. It matters more. My waiting time is more urgent than the food delivery or grocery delivery. So I can prepare the alternative plan."

Code: ● Time details   Weight score: 0
I3 , 111 - 111
Created: 24-07-2024 14:52 by Analyst1, Modified: 02-09-2024 15:55 by Analyst1
Area: 385   2.11%

19.

"And also an estimated time of arrival. So it's I think that's a Must Have in any delivery. Like, it will arrive in three minutes or 10 minutes."

Code: ● Time details   Weight score: 0
I1 , 34 - 34
Created: 29-07-2024 15:27 by Analyst2, Modified: 02-09-2024 16:29 by Analyst1
Area: 143   0.58%

20.

"I can plan my time then. There's two things. First, imagine if we try to get some delivery from the drone approaching in a public space, I think it's wise to be prepared, and know where the person or the robot or whatever thing will come. So we can actually start looking, start preparing for their arrival because it's not at our home. If it's in our home, we can just stay chill and wait until it will arrive anyway, but it's in a public park. That's one. But also the factor from the delivery methods itself, because I'm unfamiliar with drones. At least for me, it's better to be like, prepared, Okay, it's in five minutes, there'll be drones. Or maybe if it's a first time for me, maybe I just want to see a video of the drones like a first delivery with drones, just to be prepared so I can manage my time better."

Code: ● Time details   Weight score: 0
I1 , 38 - 38
Created: 29-07-2024 15:27 by Analyst2, Modified: 02-09-2024 16:29 by Analyst1
Area: 818   3.29%

21.

"the estimated time of arrival probably even more important especially during emergency. So the goal for me as a recipient is like I need to act during emergency and I'm actually actively waiting for this drone to be arrived."

Code: ● Time details   Weight score: 0
I1 , 122 - 122
Created: 29-07-2024 15:34 by Analyst2, Modified: 02-09-2024 16:29 by Analyst1
Area: 224   0.90%

3.1.3. Process tracker

1.

"When they (drones) start, they are like, okay, we see you we're gonna start delivering. It's quite difficult, I need to think about, what is the process? Imagine, I am standing there, and then the drone came from a distance. It should have something like a notification. When they arrive, I understand (drone presence) from the sound of the drone. Maybe I was talking and I was not paying attention. There should be notification that it arrives (Note: notification when they arrive). I should know the drone located me and then the moment when it delivers."

Code: ● Process tracker   Weight score: 0
I12 , 37 - 37
Created: 26-07-2024 12:43 by Analyst2, Modified: 02-09-2024 16:25 by Analyst1
Area: 557   2.62%

2.

"Yeah (Note: how are the package being delivered (flying route, gonna land or not)), again, I don't know how this drone works. It's a Must Have."

Code: ● Process tracker   Weight score: 0
I12 , 49 - 49
Created: 26-07-2024 12:46 by Analyst2, Modified: 02-09-2024 16:25 by Analyst1
Area: 143   0.67%

3.

"probably saw it's coming, it could give me a notification (Note: notification when they arrive)."

Code: ● Process tracker   Weight score: 0
I12 , 105 - 105
Created: 26-07-2024 13:03 by Analyst2, Modified: 02-09-2024 16:25 by Analyst1
Area: 96   0.45%

4.

"With the app or something, I already have an estimate of the delivery status."

Code: ● Process tracker   Weight score: 0
I9 , 85 - 85
Created: 29-07-2024 8:51 by Analyst2, Modified: 08-08-2024 9:17 by Analyst1
Area: 77   0.29%

5.

"I definitely need some more context on the delivery status"

Code: ● Process tracker   Weight score: 0
I9 , 115 - 115
Created: 26-07-2024 14:49 by Analyst1, Modified: 08-08-2024 9:17 by Analyst1
Area: 58   0.22%

6.

"I think I would like to have some kind of notification that the drone is arriving or what is happening."

Code: ● Process tracker   Weight score: 0
I8 , 14 - 14
Created: 26-07-2024 13:13 by Analyst1, Modified: 02-09-2024 16:30 by Analyst1
Area: 103   0.38%

7.

"I would like to have some notification that the drone is arriving (Note: Notification that the drone is arriving). I need to know that that's my delivery, that it's coming, in real time to see what is happening. So I think, for that, it's a must have."

Code: ● Process tracker Weight score: 0
I8 , 26 - 26
Created: 29-07-2024 9:42 by Analyst2, Modified: 02-09-2024 16:30 by Analyst1
Area: 251 0.93%

8.

"I want to know more about how the package goes (Note: the preparation of medicine process) instead of how the drone goes. So I want to know, have they already got the medicine? Have they packed the medicine? Have they load (the medicine) on the drone? I want to know all the pre-process."

Code: ● Process tracker Weight score: 0
I3 , 103 - 103
Created: 29-07-2024 12:22 by Analyst2, Modified: 02-09-2024 15:55 by Analyst1
Area: 287 1.57%

9.

"Where the medicine is right now? And how fast it's packaged? And have it got loaded on the drone? Is the drone carrying it? Is drone flying with it? So more pre-process."

Code: ● Process tracker Weight score: 0
I3 , 107 - 107
Created: 24-07-2024 14:52 by Analyst1, Modified: 02-09-2024 15:55 by Analyst1
Area: 169 0.93%

3.1.4. Live location

1.

"If it is an emergent medicine, I would be like, oh, where is it? Why is it not here yet?"

Code: ● Live location Weight score: 0
I12 , 109 - 109
Created: 29-07-2024 12:08 by Analyst1, Modified: 02-09-2024 16:25 by Analyst1
Area: 88 0.41%

2.

"This is interesting question because normally you know it (the delivery) will arrive, like in the normal delivery app. If I compare this (drone delivery) scenario to a normal delivery app, they normally will tell you like within 30 minutes or like UberEats will give you a countdown. But to be honest, it is also normal that they don't make it before the countdown. So I really want to know that there's map tracking. So I can see where the drone is, where it flies to on the map, to feel fully in control of the drone."

Code: ● Live location Weight score: 0
I10 , 91 - 91
Created: 26-07-2024 16:46 by Analyst2, Modified: 02-09-2024 16:23 by Analyst1
Area: 519 2.36%

3.

"[After some thinking] About the notification of arriving, it should have the real time location (Note: real time location)"

Code: ● Live location Weight score: 0
I8 , 34 - 34
Created: 29-07-2024 9:44 by Analyst2, Modified: 02-09-2024 16:30 by Analyst1
Area: 122 0.45%

4.

"Participant 11:11
This (Note: real time location) is a little bit about when the drone is arriving. I don't know in what way you can have only a timer or you do not know from which direction it's gonna arrive. It's like how you have with Thuisbezorgd (food delivery application in the Netherlands), with every delivery you can see. I don't know if the drone can cover the distance if I order something from within one kilometer away or from 500 meters. I don't know how it works, exactly. Maybe I would like to see the route that it's gonna take to come in."

Code: ● Live location Weight score: 0
I8 , 53 - 54
Created: 29-07-2024 9:47 by Analyst2, Modified: 02-09-2024 16:30 by Analyst1
Area: 558 2.06%

5.

"I think that it's helpful at least from what I'm used so far with the rest of the services that we're having the real time location."

Code: ● Live location Weight score: 0
I8 , 58 - 58
Created: 26-07-2024 13:35 by Analyst1, Modified: 02-09-2024 16:30 by Analyst1
Area: 132 0.49%

6.

"Not only for mine (to be idenified from the rest of drone traffic) but also to know if there are so many around. I think that can help with the real time location."

Code: ● Live location Weight score: 0
I8 , 82 - 82
Created: 29-07-2024 9:50 by Analyst2, Modified: 02-09-2024 16:30 by Analyst1
Area: 163 0.60%

7.

"For the rest of the things [however, does not answer the reasoning for all], in case if I was waiting for medicine, I would like for sure to know when it is arriving (Note: Notification that the drone is arriving) and where it is located. If I'm really sick and I need to do something last minute and now I have to go to the balcony or to the park whatever to receive the package for example."

Code: ● Live location Weight score: 0
I8 , 112 - 112
Created: 29-07-2024 9:59 by Analyst2, Modified: 02-09-2024 16:30 by Analyst1
Area: 392 1.45%

8.

"As for the Could Have, I'm going to put GPS (Note: GPS) because I think it is nice to have some form of tracking. It's not something that should happen, but it should at least have is in my whole interaction of time estimation. So the time estimation can be more specific through GPS, but at the same time, it doesn't necessarily need to have. More detailed information in that. So I think and it's also partially to do with trust and knowing what you can expect from a system, if you know that the time estimation is actually accurate, I don't really care about the GPS. But if you start thinking: Okay, but I'm not completely sure if I'm actually going to receive what I ordered, than I want more information that I'm looking at some other level of: Where can I get this confirmation that what I ordered is actually being delivered to me?"

Code: ● Live location Weight score: 0
I6 , 49 - 49
Created: 07-08-2024 16:35 by Analyst1, Modified: 07-08-2024 16:35 by Analyst1
Area: 840 2.06%

9.

"For example, the time estimation or GPS (Note: time estimation), whichever one of the two would be more accurate then that case would fit better, but having some form of tracking that I

can just refresh the web page and get an update in there and also feel like it is actually giving me an update, that would be nice."

Code: ● Live location Weight score: 0
I6 , 66 - 66
Created: 29-07-2024 11:11 by Analyst2, Modified: 01-08-2024 13:50 by Analyst1
Area: 317 0.78%

10.

"tell you the rough location of where the drone is"

Code: ● Live location Weight score: 0
I5 , 10 - 10
Created: 25-07-2024 15:49 by Analyst1, Modified: 02-09-2024 16:02 by Analyst1
Area: 49 0.12%

11.

"I thought this is probably a nice one to have: live location of the drone during the delivery (Note: Live location of the drone itself during delivery). When there's only one drone nearby, you can probably see it. But let's say there are two drones or three drones in the area, I think it's very nice to see which one is yours."

Code: ● Live location Weight score: 0
I4 , 61 - 61
Created: 29-07-2024 12:07 by Analyst2, Modified: 02-09-2024 15:58 by Analyst1
Area: 327 1.45%

12.

"The next one, could have live location of drone from warehouse to close proximity of delivery location (Note: Live location of drone from warehouse to close proximity of delivery location). Can you explain why you need that information? And how different is it from other ones?

Participant 25:13
When the drone is so close that it's time for you to get up and take action, then it's nice to know where the drone is. So you stand up, and you immediately know where you have to go or what you have to look for. With live location of the drone, from the warehouse to close proximity (in Could Have), it would be nice. If you check your phone, you can see like, oh, it's halfway. It's not that much needed. As long as you know, like, oh, it will come in half an hour. I don't really mind if it's like 20% on the way or 40% on the way. So that's why I put it in Could Have."

Code: ● Live location Weight score: 0
I4 , 93 - 95
Created: 29-07-2024 12:11 by Analyst2, Modified: 02-09-2024 15:58 by Analyst1
Area: 870 3.85%

13.

"Live location of the drone in proximity (Note: Live location of drone in proximity): I think it should have this, so you can act before hand. You know, it's coming from that way, already moved that way, or I'll get ready. So I can act as quickly as possible."

Code: ● Live location Weight score: 0
I4 , 119 - 119
Created: 25-07-2024 12:49 by Analyst1, Modified: 02-09-2024 15:58 by Analyst1
Area: 258 1.14%

14.

"I want to know more detail about how it travels and about those traveling information. So the route (Note: the travel route), where it comes from, the starting location (Note: the starting location)."

Code: ● Live location Weight score: 0
I3 , 101 - 101
Created: 29-07-2024 12:22 by Analyst2, Modified: 02-09-2024 15:55 by Analyst1

Area: 199   1.09%

3.2. Limited user intervention in drone control

3.2.1. Level of participation

1.
"What I think is too annoying is why should I know about the drone when I'm ordering nothing."
Code: ● Level of participation   Weight score: 0
I11 , 158 - 158
Created: 26-07-2024 16:16 by Analyst2, Modified: 08-08-2024 9:53 by Analyst1
Area: 92   0.46%

2.
"If I'm a bystander, I will be quite passive. So what a drone asked me to do, I will do it, just to stay safe. I would listen to the drones in general."
Code: ● Level of participation   Weight score: 0
I10 , 140 - 140
Created: 26-07-2024 15:35 by Analyst1, Modified: 02-09-2024 16:23 by Analyst1
Area: 150   0.68%

3.
"Yeah, actually I want to know it (Note: know if i need to gives space/help it (voice like ambulances/ voice of sentences))."
Code: ● Level of participation   Weight score: 0
I10 , 158 - 158
Created: 08-08-2024 9:29 by Analyst1, Modified: 02-09-2024 16:23 by Analyst1
Area: 123   0.56%

4.
"As a bystander, most of the times, I would be annoyed from the sound or, how high something drops down. Maybe also, it's gonna be interesting in case I also want something to know for the next time, but I think as a bystander, it (drone) is going to be something that will annoy."
Code: ● Level of participation   Weight score: 0
I8 , 10 - 10
Created: 29-07-2024 9:40 by Analyst2, Modified: 02-09-2024 16:30 by Analyst1
Area: 279   1.03%

5.
"if I go to a scenario (where) there are many drones around, maybe just annoying or kind of weird to feel all the time that there is someone above your head"
Code: ● Level of participation   Weight score: 0
I8 , 30 - 30
Created: 29-07-2024 9:43 by Analyst2, Modified: 02-09-2024 16:30 by Analyst1
Area: 155   0.57%

6.
"Participant 36:01
If many people are gonna have deliveries in a busy day at the park, (it) is not going to be just one drone. So if I want to have some kind of silence in and not have that sound (drone noise) all the time around me, things (packages) dropping, or, seeing drones left and right, I would like to know, in case I want to avoid that space to go."
Code: ● Level of participation   Weight score: 0
I8 , 149 - 150
Created: 29-07-2024 10:28 by Analyst2, Modified: 02-09-2024 16:30 by Analyst1
Area: 359   1.33%

7.
"To make it explode (sarcastic). I am mostly annoyed, like to say enough with the delivery. Provide some kind of feedback that what happened was not so comfortable. To have control as a bystander, is not have drones around me to be honest."

Code: ● Level of participation   Weight score: 0
I8 , 162 - 162
Created: 07-08-2024 16:36 by Analyst1, Modified: 02-09-2024 16:30 by Analyst1
Area: 238   0.88%

8.
"Analyst1 08:54
The next one (Note: A silent system)?

Participant 08:55
As I said in previous one, like when I'm the recipient, I don't want this attention grabbing or like center of attention. So, when these are going to deliver because some of the drones, when you watch it, makes quite a noise. So if it is like too noisy, and everyone knows that this drone is delivering, and this drone is moving around this part of the park. In a way I want silencer, silence."

Code: ● Level of participation   Weight score: 0
I7 , 48 - 51
Created: 29-07-2024 10:39 by Analyst2, Modified: 02-09-2024 16:31 by Analyst1
Area: 479   1.87%

9.
"I would say something not too active and something I can have some control in. So for example I ordered on the website having a little tracker on that website would be great. And then especially when that's combined with just an e-mail confirmation of my order, that would be enough information for me"

Code: ● Level of participation   Weight score: 0
I6 , 66 - 66
Created: 25-07-2024 17:26 by Analyst1, Modified: 01-08-2024 13:50 by Analyst1
Area: 301   0.74%

10.
"purely the GPS location of my phone in that moment that is being used to actually get this drone to the right location and at the moment that QR code is scanned and my package is delivered, that's the end of my active engagement with that system"

Code: ● Level of participation   Weight score: 0
I6 , 78 - 78
Created: 25-07-2024 17:31 by Analyst1, Modified: 01-08-2024 13:50 by Analyst1
Area: 245   0.60%

11.
"I would like to not be too involved with the drone."

Code: ● Level of participation   Weight score: 0
I6 , 82 - 82
Created: 25-07-2024 17:32 by Analyst1, Modified: 01-08-2024 13:50 by Analyst1
Area: 51   0.13%

12.
"I'll see my snacks show up, scan my QR code, and I'm done with this."

Code: ● Level of participation   Weight score: 0
I6 , 82 - 82
Created: 25-07-2024 17:33 by Analyst1, Modified: 01-08-2024 13:50 by Analyst1
Area: 68   0.17%

13.

"When If I'm in a bystander situation, I want to have as little active interaction with that drone as possible (Note: active interaction) because I want to allow it the space to actually fade into the background, so I don't want ---. Say for example, I'm sitting right next to someone who is actually interacting with a drone. I don't want anything to do with that as a bystander, because I don't want the decision they made to order something to influence how I'm having my day, so I wanted it to be clear signaling wise what I can expect from this drone. I've already seen a few times that people get their snacks through that. That they ordered food. Whatever. Okay, I know what to expect: It's going to show up to them and then it's going to fly away again."

Code: ● Level of participation   Weight score: 0
I6 , 157 - 157
Created: 29-07-2024 11:31 by Analyst2, Modified: 01-08-2024 13:50 by Analyst1
Area: 760   1.86%

14.

"I want it to be clear for me that I can ignore it. I want it to be clear for me that it's not expecting something of me. I'm the type of person who, when I am not expecting someone when I didn't order something and my doorbell rings, I'm not going to open because that interaction, is not an interaction that is focused on me or in any way an interaction I should have, so I'm not going to have that interaction. So especially then when I'm in the vicinity of a drone, I want to be able to ignore that as much as I can."

Code: ● Level of participation   Weight score: 0
I6 , 169 - 169
Created: 29-07-2024 11:32 by Analyst2, Modified: 01-08-2024 15:42 by Analyst1
Area: 519   1.27%

15.

"I want you to go around me so I don't hear anything"

Code: ● Level of participation   Weight score: 0
I5 , 231 - 231
Created: 29-07-2024 12:01 by Analyst2, Modified: 02-09-2024 16:02 by Analyst1
Area: 51   0.12%

16.

"No, given that it's not in my face, I would be fine."

Code: ● Level of participation   Weight score: 0
I4 , 167 - 167
Created: 29-07-2024 12:16 by Analyst2, Modified: 02-09-2024 15:58 by Analyst1
Area: 52   0.23%

17.

"I would have like to have the ability to opt out or opt in, like, please don't go near my picnic area."

Code: ● Level of participation   Weight score: 0
I1 , 174 - 174
Created: 29-07-2024 15:38 by Analyst2, Modified: 02-09-2024 16:29 by Analyst1
Area: 102   0.41%

3.2.2. Control

1.

"It really depends. If it's from the (delivery) service company, I wouldn't mind not having the control as long as it has intuitive interaction design. I don't have to control a drone and also if I think about it, if somebody else has the control over drone I wouldn't be very secure about that. So no, I wouldn't (want the control) actually."

Code: ● Control   Weight score: 0
I12 , 97 - 97
Created: 26-07-2024 13:20 by Analyst2, Modified: 02-09-2024 16:25 by Analyst1
Area: 341   1.60%

2.
"No. That would be weird. It's something that has nothing to do with me, but I want control over it? (sarcastic laugh) Yeah, I wouldn't."
Code: ● Control Weight score: 0
I12 , 165 - 165
Created: 26-07-2024 13:20 by Analyst2, Modified: 02-09-2024 16:25 by Analyst1
Area: 135 0.63%

3.
"I don't think so. You mean like to drive the drone or something like that?"
Code: ● Control Weight score: 0
I11 , 90 - 90
Created: 26-07-2024 16:08 by Analyst2, Modified: 08-08-2024 9:53 by Analyst1
Area: 74 0.37%

4.
"Okay. That will be interesting, but I think it depends on the user also. But for instance, the drone could just stop above us, and then I can control it to land in the place that I want. But I think it could be also very dangerous. If I don't know how to drive (fly) it or have no experience, I would avoid to have the service because I need to drive the drone. I think it would be interesting to open with a key or something like that."
Code: ● Control Weight score: 0
I11 , 94 - 94
Created: 26-07-2024 16:09 by Analyst2, Modified: 08-08-2024 9:53 by Analyst1
Area: 436 2.17%

5.
"I think it would be interesting to open with a key or something like that."
Code: ● Control Weight score: 0
I11 , 94 - 94
Created: 29-07-2024 11:41 by Analyst1, Modified: 08-08-2024 9:53 by Analyst1
Area: 74 0.37%

6.
"Maybe when there are bug problems (drone malfunctions). For instance, if a drone land next to me, but it's in the wrong location. But I don't know I can just catch the drone or something like that. As a bystander, I don't want to have control of the drone."
Code: ● Control Weight score: 0
I11 , 170 - 170
Created: 26-07-2024 16:17 by Analyst2, Modified: 08-08-2024 9:53 by Analyst1
Area: 256 1.28%

7.
"I want to have control over the drone in general. I don't want like, without my confirmation, he just boosts up and drop the goods and leave. I want to be in control during the interactions and I can tell him whenever I want him to leave or him to drop the goods."
Code: ● Control Weight score: 0
I10 , 47 - 47
Created: 26-07-2024 16:40 by Analyst2, Modified: 02-09-2024 16:23 by Analyst1
Area: 263 1.20%

8.
"In Won't Have, one thing is like kind of rushing me to finish some tasks (Note: rushing me (count down) to finish interaction), like finish the interaction or countdown. Basically, there are in some apps, not specifically in drones, but there are these apps that I don't like and shouldn't have is that they give you like 10 seconds to make a choice. They will automatically come down and if you don't make a choice, you go for the default one. Just to force the interaction to be finished within a certain time, so they can do the next task."
Code: ● Control Weight score: 0

I10 , 59 - 59
Created: 08-08-2024 9:25 by Analyst1, Modified: 02-09-2024 16:23 by Analyst1
Area: 542   2.46%

9.
"Um, that's a good question. I will say no. From my perspective, the drone is for someone else and someone else have the control. I wouldn't feel that I have to control the drone to feel safe. If I'm a bystander, I will be quite passive. So what a drone asked me to do, I will do it, just to stay safe. I would listen to the drones in general."
Code: ● Control   Weight score: 0
I10 , 140 - 140
Created: 26-07-2024 16:57 by Analyst2, Modified: 02-09-2024 16:23 by Analyst1
Area: 342   1.56%

10.
"Would I like to do though? Maybe not because I don't have experience flying the drones. I'm thinking of it as like someone who's interacting with it for the first time. So for the experience of it, it could be nice if I'm given the chance. But then, in this context, the use case of the drone is like delivery. So I'm expecting it to deliver something in time. In that sense, it doesn't need to give me the access to it, because I'm more concerned with what it is delivering. In that sense, I don't need to drive (fly) it. Because what if I can't do it properly? Or it could also be that there are different drones that are being used, so they all have their own ways of using it. There's going to be some constants, maybe it's inbuilt in the app. Maybe the whole landing thing could at some point be given to me as a user. Like, this is where I wanted to land. Yeah, but it's also unnecessary. I would say it (Note: control for landing) could be a Could Have. I would say control for landing could be more of a Could Have than the approaching sound."
Code: ● Control   Weight score: 0
I9 , 103 - 103
Created: 29-07-2024 8:54 by Analyst2, Modified: 08-08-2024 9:17 by Analyst1
Area: 1050   4.00%

11.
"The control does contribute to being more certain, but I don't know what that means for a bystander."
Code: ● Control   Weight score: 0
I9 , 107 - 107
Created: 26-07-2024 14:49 by Analyst1, Modified: 08-08-2024 9:17 by Analyst1
Area: 100   0.38%

12.
"Participant 25:09
But I'm also now thinking. Okay, I might be doing it more consciously and in the middle of this empty space, but that may not be the case for someone else. It's also how it's being used because they might be interacting with it for the first time."
Code: ● Control   Weight score: 0
I9 , 110 - 111
Created: 29-07-2024 8:55 by Analyst2, Modified: 08-08-2024 9:17 by Analyst1
Area: 266   1.01%

13.
"Analyst1 42:36
Do you like to have control?

Participant 42:41
No.

Analyst1 42:43
And why?

Participant 42:46
I don't know, who is getting this delivery. What for? We went through two scenarios. One was just like regular delivery versus second was for emergency care. I don't know what the purpose of delivery is, where it is headed and I don't need that information as well as a bystander. So then I also don't want control of it. So definitely I shouldn’t have control."

Code: ● Control Weight score: 0
I9 , 200 - 207
Created: 29-07-2024 9:10 by Analyst2, Modified: 08-08-2024 9:17 by Analyst1
Area: 500 1.90%

14.

"Analyst1 20:23
What about control? As a recipient, do you want to have some form of control over the drone?

Participant 20:28
On one hand, that would be nice. On the other hand, it can be done if I know how it's going to be delivered, for example. If I have to do something or if you just have to tell the drone to go more left, more right and wait for something to drop or if I have to touch something to reach and take a package. From the aspect to have control, I think I'd say it will depend. I don't know if I'm gonna trust to do the last landing. For sure, I would like to know what I will have to do in advance, like how I'm gonna receive. Maybe a validation, if I have to follow some steps and have the reassurance that I did the steps correctly."

Code: ● Control Weight score: 0
I8 , 99 - 102
Created: 29-07-2024 9:55 by Analyst2, Modified: 02-09-2024 16:30 by Analyst1
Area: 772 2.85%

15.

"From the aspect to have control, I think I'd say it will depend. I don't know if I'm gonna trust to do the last landing"

Code: ● Control Weight score: 0
I8 , 102 - 102
Created: 26-07-2024 13:47 by Analyst1, Modified: 02-09-2024 16:30 by Analyst1
Area: 119 0.44%

16.

"I said this location, but I moved from this location to this location. Can you move this? That is fine. But not like, can you do the spinning around or giving the commands to function (a certain way) or play around?"

Code: ● Control Weight score: 0
I7 , 87 - 87
Created: 26-07-2024 11:58 by Analyst1, Modified: 02-09-2024 16:31 by Analyst1
Area: 215 0.84%

17.

"Analyst1 41:28
Okay, and what about interfaces? And do you want to have control over the drone?

Participant 41:49
Control as a bystander? I have no idea on this one."

Code: ● Control Weight score: 0
I7 , 168 - 171
Created: 29-07-2024 10:51 by Analyst2, Modified: 02-09-2024 16:31 by Analyst1
Area: 180 0.70%

18.

"I can imagine I'm actually going to move away a bit from where this person is sitting. So if that happens, then it's already actually, in some ways, giving some control over me rather than the other way around."

Code: ● Control Weight score: 0
I6 , 173 - 173
Created: 26-07-2024 11:31 by Analyst1, Modified: 01-08-2024 13:50 by Analyst1
Area: 210 0.51%

19.

"I didn't order anything, then I don't want anything to do with it. I don't want any control of it, I just want to be able to ignore it."

Code: ● Control Weight score: 0
I6 , 173 - 173
Created: 26-07-2024 11:31 by Analyst1, Modified: 01-08-2024 16:37 by Analyst1
Area: 135 0.33%

20.

"it would be you just look at the map app and then pick a spot and then drop a pin and the drone will just try and land there"

Code: ● Control Weight score: 0
I5 , 142 - 142
Created: 25-07-2024 16:25 by Analyst1, Modified: 02-09-2024 16:02 by Analyst1
Area: 124 0.30%

21.

"It's not like you're going to. Like throw a rope and like, drag it down, it's not going to be physical"

Code: ● Control Weight score: 0
I5 , 142 - 142
Created: 25-07-2024 16:26 by Analyst1, Modified: 02-09-2024 16:02 by Analyst1
Area: 102 0.25%

22.

"Imagine you have a little flag and somehow the drone can detect the flag and just put it on a piece of land, say: well land here. I mark this land and this is where you going to go"

Code: ● Control Weight score: 0
I5 , 148 - 148
Created: 25-07-2024 16:27 by Analyst1, Modified: 02-09-2024 16:02 by Analyst1
Area: 180 0.44%

23.

"it could be maybe your phone. It could be a tag, whatever it is."

Code: ● Control Weight score: 0
I5 , 152 - 152
Created: 25-07-2024 16:31 by Analyst1, Modified: 02-09-2024 16:02 by Analyst1
Area: 64 0.16%

24.

"I think that would be would be fun as well as reassuring."

Code: ● Control Weight score: 0
I4 , 111 - 111
Created: 07-08-2024 16:34 by Analyst1, Modified: 02-09-2024 15:58 by Analyst1
Area: 57 0.25%

25.

"No, given that it's not in my face, I would be fine."

Code: ● Control Weight score: 0
I4 , 167 - 167
Created: 29-07-2024 12:16 by Analyst2, Modified: 02-09-2024 15:58 by Analyst1
Area: 52 0.23%

26.

"Analyst1 12:26
Okay. With regards to control, do you want to control the drone?

Participant 12:35
When I got my package then I want to say like, oh, you can fly away now and then I can press like, you can fly away and I confirm I got the package and everything set.

Participant 12:52
Okay, and why do you want to have the control?

Participant 12:56
What if I didn't finish the receiving process? If it starts to fly, then I will lose my package."

Code: ● Control Weight score: 0
I3 , 76 - 83
Created: 29-07-2024 12:21 by Analyst2, Modified: 02-09-2024 15:55 by Analyst1
Area: 464 2.54%

27.

"Analyst1 26:21
Okay. So now we go to the next part of the question, which is like: Do you like to control the drone? As a bystander?

Participant 26:33
No, I wouldn't know.

Analyst1 26:35
Why?

Participant 26:38
It's not my order. I shouldn't involve to control it.

Analyst1 26:46
What about safety? Do you like to have control or do you expect?

Participant 26:51
No. I think it's better, it's safer for me to not control."

Code: ● Control Weight score: 0
I3 , 186 - 197
Created: 29-07-2024 12:27 by Analyst2, Modified: 02-09-2024 15:55 by Analyst1
Area: 467 2.56%

28.

"it's safer for me to not control."

Code: ● Control Weight score: 0
I3 , 197 - 197
Created: 24-07-2024 15:02 by Analyst1, Modified: 02-09-2024 15:55 by Analyst1
Area: 33 0.18%

29.

"I like to have control over the drone because, in the real situation, it might be a little bit complex in the environment or surroundings. So I might need the drone to land or drop the things in a specific place. So I might need to take over the control of drone for a little bit but not many people are familiar with this drone and how to control it. For the safety, people can only control the drones using like limited control."

Code: ● Control Weight score: 0
I2 , 47 - 47
Created: 07-08-2024 16:30 by Analyst1, Modified: 02-09-2024 16:26 by Analyst1

Area: 430   1.82%

30.
"I don't think I have the neccessity to control the drone, if I'm a bystander. I don't want to control them. It is weird to control them."
Code: ● Control   Weight score: 0
I2 , 156 - 156
Created: 29-07-2024 14:57 by Analyst2, Modified: 02-09-2024 16:26 by Analyst1
Area: 136   0.58%

31.
"it's also interesting if we can also gesture to have some kind of navigation for the drone, like: move slightly towards here, because I don't like you to land where we have like picnic with babies and families and like elders, so move here. Maybe I could control how the drones will land or like moves slightly to the right or, slowly or faster."
Code: ● Control   Weight score: 0
I1 , 98 - 98
Created: 24-07-2024 13:10 by Analyst1, Modified: 02-09-2024 16:29 by Analyst1
Area: 345   1.39%

32.
"I would have like to have the ability to opt out or opt in, like, please don't go near my picnic area."
Code: ● Control   Weight score: 0
I1 , 174 - 174
Created: 29-07-2024 15:38 by Analyst2, Modified: 02-09-2024 16:29 by Analyst1
Area: 102   0.41%

3.3. Landing/take-off intention of the drone

3.3.1. Signal of landing and take-off

1.
"The moment the deliver starts (Note: the moments when the delivery starts), it's like (important) because I don't know how the drone gonna deliver. I imagine it stops somewhere and then slowly drops the package for example. So when they stop and then start delivering, so that would be the moment that I want to know when it starts."
Code: ● Signal of landing and take-off   Weight score: 0
I12 , 41 - 41
Created: 26-07-2024 12:44 by Analyst2, Modified: 02-09-2024 16:25 by Analyst1
Area: 332   1.56%

2.
"I put here, so far, was indicator of landing (Note: Indicator of landing). As someone who is just watching the situation, it will be nice to know somehow with the light or like a friendly noise that something is coming and delivering. It can make me feel scary because there's a noise."
Code: ● Signal of landing and take-off   Weight score: 0
I11 , 142 - 142
Created: 26-07-2024 16:14 by Analyst2, Modified: 08-08-2024 9:53 by Analyst1
Area: 285   1.42%

3.
"it is telling me that it is landing (Note: tell me it is landing)."
Code: ● Signal of landing and take-off   Weight score: 0
I10 , 23 - 23
Created: 26-07-2024 16:28 by Analyst2, Modified: 02-09-2024 15:53 by Analyst1
Area: 66   0.30%

4.

"it should tell everyone around that it is landing, because landing could be a quite dangerous action."

Code: ● Signal of landing and take-off   Weight score: 0
I10 , 27 - 27
Created: 26-07-2024 16:36 by Analyst2, Modified: 02-09-2024 15:53 by Analyst1
Area: 101   0.46%

5.

"have the confirmation that I will receive the goods then he will just drop automatically, leave it on the ground, and then he would just get into the air be away from me. So I would feel safe to take the stuff."

Code: ● Signal of landing and take-off   Weight score: 0
I10 , 39 - 39
Created: 26-07-2024 15:13 by Analyst1, Modified: 02-09-2024 16:23 by Analyst1
Area: 210   0.96%

6.

"But of course, before it is leaving, he should tell me that it is leaving. When it is leaving (Note: tell me it is leaving), it is dangerous, because they will turn on the helicopter thing (rotors), which could be quite dangerous and a lot of noise. Before it is leaving, I want something like a countdown from him, Hey, I'm leaving within five seconds. So get away from me and don't be nearby, things like that. I think more or less these are Must Have because these are quite safety relevant."

Code: ● Signal of landing and take-off   Weight score: 0
I10 , 39 - 39
Created: 26-07-2024 16:40 by Analyst2, Modified: 02-09-2024 16:23 by Analyst1
Area: 494   2.25%

7.

"any indication that the drone will land."

Code: ● Signal of landing and take-off   Weight score: 0
I10 , 119 - 119
Created: 26-07-2024 15:31 by Analyst1, Modified: 02-09-2024 16:23 by Analyst1
Area: 40   0.18%

8.

"If it is above you, you want to know that it is landing or not in general. So I will try to be safe as (much as) possible so I want to make sure that if it is landing I'm not nearby it. That is important, I think to anybody. For current drone technologies, I think they won't even land if people are nearby or underneath. They should always need to tell people that it is landing or where it will land."

Code: ● Signal of landing and take-off   Weight score: 0
I10 , 123 - 123
Created: 26-07-2024 16:54 by Analyst2, Modified: 02-09-2024 16:23 by Analyst1
Area: 402   1.83%

9.

"They should always need to tell people that it is landing or where it will land."

Code: ● Signal of landing and take-off   Weight score: 0
I10 , 123 - 123
Created: 26-07-2024 15:32 by Analyst1, Modified: 02-09-2024 16:23 by Analyst1
Area: 80   0.36%

10.

"When the drone landed and the recipient is getting stuff from it (the drone), it should totally stop (the rotors). When you stop, people will feel that it's safe, that they can approach it, they can touch it, or they can even pick it up, or they can look at it closely. When it (drone) boosts

up and the things are turning on, it could be suddenly quite dangerous and surprising for bystanders. It's always good to tell that, hey, I'm gonna turning on again, stay away from me."

Code: ● Signal of landing and take-off   Weight score: 0
I10 , 136 - 136
Created: 26-07-2024 16:57 by Analyst2, Modified: 02-09-2024 16:23 by Analyst1
Area: 477   2.17%

11.

"n a garden, or an open space context, if this group of people is just beside me, then this visual of where this drone is going to land is more of a Must Have"

Code: ● Signal of landing and take-off   Weight score: 0
I9 , 155 - 155
Created: 26-07-2024 14:54 by Analyst1, Modified: 08-08-2024 9:17 by Analyst1
Area: 157   0.60%

12.

"You will see some form of a descent when it's approaching delivery. It's difficult to predict what this descent looks like. All you can sense is that it's coming downwards."

Code: ● Signal of landing and take-off   Weight score: 0
I9 , 159 - 159
Created: 26-07-2024 14:55 by Analyst1, Modified: 08-08-2024 9:17 by Analyst1
Area: 172   0.66%

13.

"I want to know where it lands (Note: Landing location, signal (lights, audio, projection)). When it starts landing, I want the landing signal."

Code: ● Signal of landing and take-off   Weight score: 0
I3 , 147 - 147
Created: 29-07-2024 12:25 by Analyst2, Modified: 02-09-2024 15:55 by Analyst1
Area: 142   0.78%

3.3.2. Location

1.

"the drone located me in the start (Note: the drone located me)."

Code: ● Location   Weight score: 0
I12 , 105 - 105
Created: 26-07-2024 13:03 by Analyst2, Modified: 02-09-2024 16:25 by Analyst1
Area: 63   0.30%

2.

"I would think I need to order (the drone) to a free space to make sure that the drone will land right (on the ground)."

Code: ● Location   Weight score: 0
I11 , 22 - 22
Created: 29-07-2024 11:27 by Analyst1, Modified: 08-08-2024 9:53 by Analyst1
Area: 118   0.59%

3.

"if I need to stay in a specific location (Note: Landing location). So size is also linked with the landing location. So I was thinking about Uber, for instance, you need to stay in a specific point to get your ride."

Code: ● Location   Weight score: 0
I11 , 30 - 30
Created: 26-07-2024 13:47 by Analyst2, Modified: 08-08-2024 9:53 by Analyst1
Area: 215   1.07%

4.

"I put size in this specific context, more related to where I need to be, and to make sure that the drone will not hit anybody or something like that."

Code: ● Location Weight score: 0
I11 , 34 - 34
Created: 26-07-2024 13:48 by Analyst2, Modified: 08-08-2024 9:53 by Analyst1
Area: 149 0.74%

5.

"Also, where it lands which is also same as previous (as in Q2 answer) (Note: where it lands (projection/sound)). It need not be a projection but any indication that the drone will land. [Rethought] Yep, projection could be or it could be sound, just telling people that it is landing and so people won't get underneath."

Code: ● Location Weight score: 0
I10 , 119 - 119
Created: 26-07-2024 16:53 by Analyst2, Modified: 02-09-2024 16:23 by Analyst1
Area: 319 1.45%

6.

"Participant 34:59
Yeah, again some sort of a visual (Note: visual of landing) that should be a Must Have.

Analyst1 35:06
And how do you want it?

Participant 35:13
This is also what a bystander means, right? In a garden, or an open space context, if this group of people is just beside me, then this visual of where this drone is going to land is more of a Must Have (Note: visual of landing). So depends on the vicinity of how close you are, and how close the drone is, if it's flying from above you. So it's more to do with that.

Analyst1 35:46
Why does it depend on the proximity or vicinity? Like how close you're the delivery point or something?

Participant 35:55
You will see some form of a descent when it's approaching delivery. It's difficult to predict what this descent looks like. All you can sense is that it's coming downwards. So yeah, that's why I said that.

Analyst1 36:20
So the visual of what?

Participant 36:26
The visual of landing."

Code: ● Location Weight score: 0
I9 , 150 - 163
Created: 29-07-2024 9:05 by Analyst2, Modified: 08-08-2024 9:17 by Analyst1
Area: 1001 3.81%

7.

"in the park, if the drones are landing in a specific spot, I think it's gonna be good to get notified that this is a landing spot so you know as a bystander that, okay, if I go there, there are gonna be drones around."

Code: ● Location Weight score: 0
I8 , 146 - 146
Created: 29-07-2024 10:28 by Analyst2, Modified: 02-09-2024 16:30 by Analyst1
Area: 217 0.80%

8.
"I said this location, but I moved from this location to this location. Can you move this?"
Code: ● Location Weight score: 0
I7 , 87 - 87
Created: 29-07-2024 10:41 by Analyst2, Modified: 02-09-2024 16:31 by Analyst1
Area: 89 0.35%

9.
"maybe there's a pick up location that I should already make my way there."
Code: ● Location Weight score: 0
I5 , 10 - 10
Created: 25-07-2024 15:50 by Analyst1, Modified: 02-09-2024 16:02 by Analyst1
Area: 73 0.18%

10.
"I guess you do walk towards a specific spot where it drops, and then you basically pick it up."
Code: ● Location Weight score: 0
I4 , 19 - 19
Created: 25-07-2024 11:39 by Analyst1, Modified: 02-09-2024 15:58 by Analyst1
Area: 94 0.42%

11.
"Yeah, sure. So for the first one, the delivery location (Note: Delivery location). I think this one is probably the most crucial for me. When you order something from a drone, it's something new and never done before and knowing where you can be to receive it, either by picking it up somewhere or waiting somewhere for the drone to come to you. I think that is the most crucial and you should definitely know where to be to get it."
Code: ● Location Weight score: 0
I4 , 33 - 33
Created: 29-07-2024 12:06 by Analyst2, Modified: 02-09-2024 15:58 by Analyst1
Area: 432 1.91%

12.
"I would be a bit uncertain whether they will drop it in front of the door, in the back garden, or, maybe the delivery location will say: I will be at the park. Then it's doesn't have enough indication for me."
Code: ● Location Weight score: 0
I4 , 41 - 41
Created: 25-07-2024 11:43 by Analyst1, Modified: 02-09-2024 15:58 by Analyst1
Area: 208 0.92%

13.
"I think those are absolutely crucial (Note1: Delivery location; Note2: Some signal that it knows how to find me - location sharing, face detection). I think without those, it will probably be hard to get people to feel happy and give them a safe feeling about the process"
Code: ● Location Weight score: 0
I4 , 81 - 81
Created: 29-07-2024 12:09 by Analyst2, Modified: 02-09-2024 15:58 by Analyst1
Area: 271 1.20%

14.
"The same goes for the delivery location, they have to know where they can wait or where they can receive the emergency medicines (Note: Delivery location)."
Code: ● Location Weight score: 0
I4 , 119 - 119
Created: 29-07-2024 12:13 by Analyst2, Modified: 02-09-2024 15:58 by Analyst1
Area: 155 0.69%

15.

"I think something that probably should have given the age that we live in, the ability to adapt the delivery location (Note: Ability to adapt delivery location). So say that time is really of the essence. Maybe you know, from which way the drone is coming, and you can move into that direction. Or maybe you are driving somewhere, but then the road is blocked, or you have to move somewhere else, then you don't really have the time to get to the original delivery location. I think in that case, it should be able to adapt."

Code: ● Location Weight score: 0
I4 , 119 - 119
Created: 25-07-2024 12:48 by Analyst1, Modified: 02-09-2024 15:58 by Analyst1
Area: 524 2.32%

16.

"So the landing location, I want to know where it lands"

Code: ● Location Weight score: 0
I3 , 147 - 147
Created: 29-07-2024 12:24 by Analyst2, Modified: 02-09-2024 15:55 by Analyst1
Area: 54 0.30%

17.

"it decided to land somewhere where the older (recipient) cannot reach, or they're dying, I can help to deliver it to them."

Code: ● Location Weight score: 0
I3 , 205 - 205
Created: 29-07-2024 12:29 by Analyst2, Modified: 02-09-2024 15:55 by Analyst1
Area: 122 0.67%

18.

"Yeah. Where to pick up and how to pick up? Because we don't have a dedicated space in the park, assuming there is no dedicated space in the park. So yeah, I think it needs to be clear. Should I just stay in my area of sitting or should I walk into like less crowded space or empty space?"

Code: ● Location Weight score: 0
I1 , 34 - 34
Created: 29-07-2024 15:27 by Analyst2, Modified: 02-09-2024 16:29 by Analyst1
Area: 287 1.16%

19.

"realize when we change the scenario, there is also a change in terms of priority in the Must Have, like where to pick up suddenly becomes less relevant."

Code: ● Location Weight score: 0
I1 , 122 - 122
Created: 29-07-2024 15:35 by Analyst2, Modified: 02-09-2024 16:29 by Analyst1
Area: 152 0.61%

3.3.3. Delivery method

1.

"I don't know how they deliver it. I don't know if the drone would land or how are the package being delivered. Is it gonna land or not, like the more detailed behavior."

Code: ● Delivery method Weight score: 0
I12 , 33 - 33
Created: 29-07-2024 11:54 by Analyst1, Modified: 02-09-2024 16:25 by Analyst1
Area: 168 0.79%

2.

"I don't know how the drone gonna deliver. I imagine it stops somewhere and then slowly drops the package for example."

Code: ● Delivery method Weight score: 0
I12 , 41 - 41

Created: 29-07-2024 11:58 by Analyst1, Modified: 02-09-2024 16:25 by Analyst1
Area: 117   0.55%

3.
"how it will be delivered. If it's the first time, I want to have more instructions."
Code: ● Delivery method   Weight score: 0
I12 , 53 - 53
Created: 29-07-2024 12:01 by Analyst1, Modified: 02-09-2024 16:25 by Analyst1
Area: 83   0.39%

4.
"Maybe I can approach the drone to give me access to the thing (to deliver the package), or the drone can just drop the thing (package) for me. I think the idea of drop things is not good."
Code: ● Delivery method   Weight score: 0
I11 , 74 - 74
Created: 29-07-2024 11:38 by Analyst1, Modified: 08-08-2024 9:53 by Analyst1
Area: 187   0.93%

5.
"I imagine is that he won't drop the stuff on the ground automatically, there's something that is catching the goods and I have to take it out by myself. It could also be that the drone can drop the goods on the ground or automatically and then leave it."
Code: ● Delivery method   Weight score: 0
I10 , 35 - 35
Created: 26-07-2024 15:12 by Analyst1, Modified: 02-09-2024 16:23 by Analyst1
Area: 253   1.15%

6.
"I will prefer that it can drop on the ground automatically."
Code: ● Delivery method   Weight score: 0
I10 , 39 - 39
Created: 26-07-2024 15:12 by Analyst1, Modified: 02-09-2024 16:23 by Analyst1
Area: 59   0.27%

7.
"It depends on how it is being delivered. Is the drone landing down all the way to the ground and putting the delivery or giving it to my hand? If it is given to my hand, then I don't know, how close to me it will come, especially if it's my first interaction."
Code: ● Delivery method   Weight score: 0
I9 , 15 - 15
Created: 26-07-2024 14:23 by Analyst1, Modified: 08-08-2024 9:17 by Analyst1
Area: 259   0.99%

8.
"lso as a Must Have, can be like, how I'm gonna receive the package (Note: how am I going to receive the package? (ex wait for the drone to land). Are they gonna drop it or is it gonna land? Also for safety reasons to know how close I can approach or not."
Code: ● Delivery method   Weight score: 0
I8 , 34 - 34
Created: 29-07-2024 9:54 by Analyst2, Modified: 02-09-2024 16:30 by Analyst1
Area: 292   1.08%

9.
"Another is about how I'm gonna receive the package. I have to wait, for example, (for) the drone to land. If I have to read the drone in a specific way and to wait for further instructions."
Code: ● Delivery method   Weight score: 0
I8 , 38 - 38
Created: 29-07-2024 9:53 by Analyst2, Modified: 02-09-2024 16:30 by Analyst1
Area: 189   0.70%

10.
"I believe it's important, especially if there are different ways to deliver a package, to know beforehand what I have to do."
Code: ● Delivery method   Weight score: 0
I8 , 38 - 38
Created: 26-07-2024 13:20 by Analyst1, Modified: 02-09-2024 16:30 by Analyst1
Area: 124   0.46%

11.
"if you have to wait for the drone to land, maybe it's not good to approach a lot, you do not want to have an injury, maybe someone else is around and I don't know what side or anything. I do not know how automating the landing (procedure) can be"
Code: ● Delivery method   Weight score: 0
I8 , 42 - 42
Created: 26-07-2024 13:21 by Analyst1, Modified: 02-09-2024 16:30 by Analyst1
Area: 245   0.90%

12.
"Analyst1 21:35
Okay, and why do you want to know this information?

Participant 21:40
It (drones delivering packages) is something completely new and for safety. If I'm waiting for a bag of chips, and they just throw me a bag of chips, it's fine (and there is not much scope for package damage), but if I want a Coca Cola and it's a bit heavier, I need to know a little bit what I have to do. If I ordered and the delivery (method) is different. If I delivered and it (drone) comes where I am, or if I have to walk to receive something somewhere else."
Code: ● Delivery method   Weight score: 0
I8 , 103 - 106
Created: 29-07-2024 9:55 by Analyst2, Modified: 02-09-2024 16:30 by Analyst1
Area: 565   2.09%

13.
"Will they actually land? Do they just throw my snacks around the sky and say bye?"
Code: ● Delivery method   Weight score: 0
I1 , 14 - 14
Created: 07-08-2024 16:29 by Analyst1, Modified: 02-09-2024 16:29 by Analyst1
Area: 81   0.33%

14.
"Because I am wondering how it will be? Like a helicopter and we have a helipad, we already know the helicopter will land on that particular spot."
Code: ● Delivery method   Weight score: 0
I1 , 14 - 14
Created: 24-07-2024 12:35 by Analyst1, Modified: 02-09-2024 16:29 by Analyst1
Area: 145   0.58%

15.
"Should I just stay in my area of sitting or should I walk into like less crowded space or empty space? So I can pick up the package and how to pick up? Will the drone land or shall I grab the package from the drone while it's floating in the air?"
Code: ● Delivery method   Weight score: 0
I1 , 34 - 34
Created: 24-07-2024 12:45 by Analyst1, Modified: 02-09-2024 16:29 by Analyst1
Area: 246   0.99%

3.4. Recognition of the drone and recipient

3.4.1. Details of recipient

1.
"Yeah, I put here in Must Have recipient information (Note: Recipient information). As a bystander in the scenario of emergency medicine, it could be interesting to have the recipient information. For instance, if the person is not able to pick the medicine, I can deliver (it) to the person. If I am taking care of someone that ordered this, I can go there and pick. So I think it could be interesting."
Code: ● Details of recipient   Weight score: 0
I11 , 174 - 174
Created: 26-07-2024 16:18 by Analyst2, Modified: 08-08-2024 9:53 by Analyst1
Area: 402   2.00%

2.
"If it is for emergency, the case could be a little bit different. I first want to know, the medicine is for who. It should be in more definitive way, I would say. It should be a visual way like a printed receipt. I was just thinking about if there are any ways that help us to identify the person that needs a medicine, probably by sky guidance."
Code: ● Details of recipient   Weight score: 0
I10 , 144 - 144
Created: 26-07-2024 16:57 by Analyst2, Modified: 02-09-2024 16:23 by Analyst1
Area: 345   1.57%

3.
"If someone ordered emergence medicine in public area, he might still be conscious, when they receive the medicine so he can do it by himself. But if he lost his consciousness, it will be like they have diabetes and they already fainted and but they need insulin (immediately). In that case, the bystander will be the one who actually becomes the recipient. But then they will need some guidance to find out where the patient is."
Code: ● Details of recipient   Weight score: 0
I10 , 148 - 148
Created: 26-07-2024 16:58 by Analyst2, Modified: 02-09-2024 16:23 by Analyst1
Area: 428   1.95%

4.
"I want the drone easy to identify because I want to know where it goes to and to whom (Note: for who (visual receipt/voice guidance)). I know that it is for emergency because of its colors and then I want to know for who it is going for, so I can give space for it or help it to get to the person."
Code: ● Details of recipient   Weight score: 0
I10 , 154 - 154
Created: 26-07-2024 16:59 by Analyst2, Modified: 02-09-2024 16:23 by Analyst1
Area: 297   1.35%

5.
"For example, if we take a large, big park, and they have like different type of corners and you are stuck in one corner and the drone is approaching you, for example. And if it has an audio saying that I'm delivering medicine to this person in this zone and has this medicine, people might approach the recipient."
Code: ● Details of recipient   Weight score: 0
I7 , 119 - 119
Created: 29-07-2024 10:46 by Analyst2, Modified: 02-09-2024 16:31 by Analyst1
Area: 313   1.22%

6.

"The name or something of the recipient (Note: name of the recipient), if the drone lost its way or moved from one place to other, as we discussed in the first case of the recipient. The drone didn't understand as it has no Google Maps or something. If it is possible, and the name is displayed in a way, we can just go around and ask. It would be nice."

Code: ● Details of recipient   Weight score: 0
I7 , 175 - 175
Created: 29-07-2024 10:51 by Analyst2, Modified: 02-09-2024 16:31 by Analyst1
Area: 352   1.37%

7.

"Something like that would have some relevance because say something actually did happen to the recipients. Who ordered these emergency medication? Maybe they were really in need of that emergency medication. So there's some relevance of bystanders there."

Code: ● Details of recipient   Weight score: 0
I6 , 181 - 181
Created: 29-07-2024 11:34 by Analyst2, Modified: 02-08-2024 10:33 by Analyst1
Area: 254   0.62%

8.

"A screen on that drone saying: Hey, I have an emergency delivery for so and so."

Code: ● Details of recipient   Weight score: 0
I6 , 197 - 197
Created: 29-07-2024 11:36 by Analyst2, Modified: 01-08-2024 13:50 by Analyst1
Area: 79   0.19%

9.

"in an emergency situation, I just somehow imagine people would be like, well, it's a good idea to put the people's name and their condition on the voice announcement or something."

Code: ● Details of recipient   Weight score: 0
I5 , 190 - 190
Created: 25-07-2024 16:36 by Analyst1, Modified: 02-09-2024 16:02 by Analyst1
Area: 179   0.44%

3.4.2. Tutorial

1.

"This could be some kind of introductory instruction (Note: instruction). One of the possibilities like how it will be delivered. If it's the first time, I want to have more instructions."

Code: ● Tutorial   Weight score: 0
I12 , 53 - 53
Created: 29-07-2024 12:01 by Analyst1, Modified: 02-09-2024 16:25 by Analyst1
Area: 186   0.87%

2.

"Instructions (Note: instruction) are a Should Have. I would consider when or where to give it. If it's the first time I use it, it should have an instruction somewhere, in case it is needed. But I would expect the whole interaction design to be very smooth, very intuitive, so I don't have to go read through or follow the instruction."

Code: ● Tutorial   Weight score: 0
I12 , 109 - 109
Created: 26-07-2024 13:04 by Analyst2, Modified: 02-09-2024 16:25 by Analyst1
Area: 335   1.57%

3.

"Yes, size of the drone. For instance, when I asked for a car on Uber, usually you can see the brand and the type of the car. So you can imagine like I have three people and they are very tall, and it will be difficult to be comfortable in this car. So you can have an idea about it. I put

size in this specific context, more related to where I need to be, and to make sure that the drone will not hit anybody or something like that."

Code: ● Tutorial   Weight score: 0
I11 , 34 - 34
Created: 26-07-2024 13:47 by Analyst2, Modified: 08-08-2024 9:53 by Analyst1
Area: 432   2.15%

4.

"Assuming that this is my first time doing a delivery like this, I don't know where this drone is going to land, and how is this delivery going to happen. It must have an indicator of what is expected of me, like some sort of visual of scenario of drone and me."

Code: ● Tutorial   Weight score: 0
I9 , 41 - 41
Created: 29-07-2024 8:44 by Analyst2, Modified: 08-08-2024 9:17 by Analyst1
Area: 260   0.99%

5.

"Yes. This is before the drone arrives."

Code: ● Tutorial   Weight score: 0
I9 , 45 - 45
Created: 29-07-2024 8:44 by Analyst2, Modified: 08-08-2024 9:17 by Analyst1
Area: 38   0.14%

6.

"For the first instance, I need a visual of the scenario that establishes the relationship of the drone and me, and how this delivery is going to take place. For the later onwards, I don't need a visual per se, but, okay, drone is going to arrive in two minutes, just that is enough. I can think of the Must Have and the Should Have. But for later, it could be the Could Have or Won't Have. After some point when it becomes more mainstream, then you don't need this anymore."

Code: ● Tutorial   Weight score: 0
I9 , 61 - 61
Created: 29-07-2024 8:46 by Analyst2, Modified: 08-08-2024 9:17 by Analyst1
Area: 473   1.80%

7.

"That (appearance) should really match with what is coming. That makes a difference from the UberEATS delivery because this person is really coming to your doorstep without the vehicle. So a very generic image of the vehicle would work. But because I'm seeing the drone, at least at the start, when it's my first interaction, it will be weird. If I'm expecting something smaller, and it turns out to be way bigger, but I think if it is way bigger in my head, and it's smaller in reality, then I wouldn't be as intimidated. So yeah, a comparable visual of the drone (Note: comparable visual of drone) would be a Should Have."

Code: ● Tutorial   Weight score: 0
I9 , 73 - 73
Created: 29-07-2024 8:47 by Analyst2, Modified: 08-08-2024 9:17 by Analyst1
Area: 622   2.37%

8.

"What I mean with the comparable visual of the drone is, I was trying to make two points. One is with the size of it. This drone, as a piece of technology, is coming close to me or is delivering object in my vicinity. So I think it (uncertainty) is something related to the size of it. If it is larger than what I expect, then I might be taken by surprise. I wouldn't be surprised, I would be surprised either ways (smaller or bigger than I imagined the drone to be). So let's say I'm expecting, if it (the drone) is really large, I will be surprised. If it's really small, I will also be surprised. But if it's somewhere close by (to my expectation of size) then I will be lesser surprised. It will be more of oh, is it coming near to me and it's bigger than what I expect, then

I might want to go away from it. So that's why I said it might be more intimidating if it's bigger and approaching closer."

Code: ● Tutorial Weight score: 0
I9 , 77 - 77
Created: 29-07-2024 8:48 by Analyst2, Modified: 08-08-2024 9:17 by Analyst1
Area: 902 3.44%

9.

"That's why a visual of the drone and me in the scenario, even if that is more generic, but some indication of what to expect, will be nicer. The drone and me, I think, like you know what an average human (size) is (relative to the drone). You can get an estimate of the size with this visual."

Code: ● Tutorial Weight score: 0
I9 , 81 - 81
Created: 29-07-2024 8:48 by Analyst2, Modified: 08-08-2024 9:17 by Analyst1
Area: 292 1.11%

10.

"Analyst1 28:21
What about comparable visual of the drone?

Participant 28:28
It remains. It will be nice if the compatible visual is somehow made a part of the of this as well (Note: visual of scenario drone and me). So that's also true for the previous one (for recipient), I would say. If it's like somehow made a part of this visual."

Code: ● Tutorial Weight score: 0
I9 , 116 - 119
Created: 29-07-2024 8:59 by Analyst2, Modified: 08-08-2024 9:17 by Analyst1
Area: 350 1.33%

11.

"If I think as a first-time user, I will have no idea what every sound or every color is gonna mean. (If) I see something red, maybe it's something to not approach. If you put under consideration that (some) people are color blind, or especially in the Netherlands, where people from different countries have different views about how to approach or what not to do. If there are a lot of cues on a drone, maybe we'll want to have some general instructions before, to know a little bit how the service is working. Some kind of education before you interact, you can be completely comfortable using it."

Code: ● Tutorial Weight score: 0
I8 , 50 - 50
Created: 29-07-2024 9:46 by Analyst2, Modified: 02-09-2024 16:30 by Analyst1
Area: 599 2.21%

12.

"That's why I said that maybe the education about how these (drone delivery) services working can be helpful to understand a bit what can happen and what to expect."

Code: ● Tutorial Weight score: 0
I8 , 170 - 170
Created: 26-07-2024 14:08 by Analyst1, Modified: 02-09-2024 16:30 by Analyst1
Area: 163 0.60%

13.

"if it's a first time for me, maybe I just want to see a video of the drones like a first delivery with drones, just to be prepared so I can manage my time better."

Code: ● Tutorial Weight score: 0
I1 , 38 - 38
Created: 24-07-2024 12:52 by Analyst1, Modified: 02-09-2024 16:29 by Analyst1
Area: 162 0.65%

14.

"we need to have some kind of educational awareness for the public and with the governmental logos."

Code: ● Tutorial Weight score: 0
I1 , 166 - 166
Created: 24-07-2024 13:26 by Analyst1, Modified: 02-09-2024 16:29 by Analyst1
Area: 98 0.39%

3.4.3. Presence

1.

"When they arrive, I understand (drone presence) from the sound of the drone. Maybe I was talking and I was not paying attention."

Code: ● Presence Weight score: 0
I12 , 37 - 37
Created: 29-07-2024 11:56 by Analyst1, Modified: 02-09-2024 16:25 by Analyst1
Area: 129 0.61%

2.

"Not specific to what is delivering but just to know that there is a drone delivering something around. It must be a very soft song, otherwise it will be like annoying like, oh my god (irritated expression)."

Code: ● Presence Weight score: 0
I11 , 150 - 150
Created: 26-07-2024 16:15 by Analyst2, Modified: 08-08-2024 9:53 by Analyst1
Area: 206 1.03%

3.

"If drone delivery is a normal service in the future. You want it to be identified from far away."

Code: ● Presence Weight score: 0
I10 , 71 - 71
Created: 26-07-2024 15:17 by Analyst1, Modified: 02-09-2024 16:23 by Analyst1
Area: 96 0.44%

4.

"It is always better that you are ready for a drone coming to you and you see it coming rather than it just pops out near you, unknowingly."

Code: ● Presence Weight score: 0
I10 , 71 - 71
Created: 26-07-2024 15:18 by Analyst1, Modified: 02-09-2024 16:23 by Analyst1
Area: 138 0.63%

5.

"The additional factor is I know that there is something or someone approaching me. Some sort of an update on that."

Code: ● Presence Weight score: 0
I9 , 35 - 35
Created: 29-07-2024 8:43 by Analyst2, Modified: 08-08-2024 9:17 by Analyst1
Area: 114 0.43%

6.

"The sound (Note: approaching sound) would be a nice, Could Have. I think it's more valuable for a bystander."

Code: ● Presence Weight score: 0
I9 , 85 - 85
Created: 08-08-2024 9:09 by Analyst1, Modified: 08-08-2024 9:17 by Analyst1
Area: 108 0.41%

7.

"approaching sound (communicates) kind of make way for it (the drone)."

Code: ● Presence Weight score: 0
I9 , 123 - 123
Created: 29-07-2024 8:59 by Analyst2, Modified: 08-08-2024 9:17 by Analyst1
Area: 69 0.26%

8.

"So the first thing that I think of is the presence. The presence of the drone I'm thinking about: Should the drone announce itself to the people that is around it? And of course, you know, it's a big piece of flying object. Of course people are going to see it. But maybe if people are preoccupied in a way that they won't be able to see it. I think it's also nice. Drone to announce itself and also I don't know how quiet they are, I guess the fans are going to be a little loud, but the part that I'm doubting is that is it a Must Have or is it Should Have so that's when I'm just speaking out loud, of course. That's what you want me to do right? I'm just trying to decide if it's a Must Have or Should Have.
I think I'll give it a must because people seeing the drone themselves is one thing, but I feel like as something that is going to enter a public space and also people's space it's also nice for the drones--- Oh wait, by the way, are you recording and just because we restarted it, I don't want you to forget..."

Code: ● Presence Weight score: 0
I5 , 56 - 57
Created: 29-07-2024 11:44 by Analyst2, Modified: 02-09-2024 16:02 by Analyst1
Area: 1023 2.50%

9.

"I don't want it to be noise. It's just that you need to---. I hope there is a more ambient way for stating the purpose."

Code: ● Presence Weight score: 0
I5 , 73 - 73
Created: 25-07-2024 16:11 by Analyst1, Modified: 02-09-2024 16:02 by Analyst1
Area: 119 0.29%

10.

"I'm assuming that it should announce its announce itself to the public space"

Code: ● Presence Weight score: 0
I5 , 77 - 77
Created: 29-07-2024 11:45 by Analyst2, Modified: 02-09-2024 16:02 by Analyst1
Area: 76 0.19%

11.

"I think it's for an object. It should be announcing itself to the environment that is in. It's like human walk in and you see them, you know, it doesn't matter if you acknowledge its presence or not. You know that there's a human being. It's for the person that is within the space to know what is around them. I think that is a basic--- It's a safety thing. It's a social thing, and in general, you know what I mean, I find it kind of important.
It's kind of like somebody entered a room and you need to, make eye contact or something somehow sort of make them know that. Okay, I see you. You know what I mean? Like you have to just announce yourself. But I think for a piece of machine it should do the same because it's entering a social, public human space well, not just human, but, you know. But social is between human, right? So I think it should make it known that it's entering the space so other people can watch out if it's flying too close or if it's going to do something within the space. People should know. So that's for presence for me"

Code: ● Presence Weight score: 0
I5 , 85 - 86
Created: 29-07-2024 11:46 by Analyst2, Modified: 02-09-2024 16:02 by Analyst1
Area: 1053 2.58%

12.
"This is not just for the recipient, but this is more for just pretty much everybody."
Code: ● Presence   Weight score: 0
I5 , 87 - 87
Created: 25-07-2024 16:14 by Analyst1, Modified: 02-09-2024 16:02 by Analyst1
Area: 84   0.21%

13.
"magine if we try to get some delivery from the drone approaching in a public space, I think it's wise to be prepared, and know where the person or the robot or whatever thing will come."
Code: ● Presence   Weight score: 0
I1 , 38 - 38
Created: 24-07-2024 12:50 by Analyst1, Modified: 02-09-2024 16:29 by Analyst1
Area: 185   0.74%

3.4.4. Verification

1.
"I think this (Note: the drone located me) is related to the intention I just mentioned. So it's like, they see me."
Code: ● Verification   Weight score: 0
I12 , 31 - 31
Created: 08-08-2024 10:02 by Analyst1, Modified: 02-09-2024 16:25 by Analyst1
Area: 114   0.54%

2.
"I should know the drone located me"
Code: ● Verification   Weight score: 0
I12 , 37 - 37
Created: 29-07-2024 11:57 by Analyst1, Modified: 02-09-2024 16:25 by Analyst1
Area: 34   0.16%

3.
"a Should Have is deliver finish (Note: deliver finish)."
Code: ● Verification   Weight score: 0
I12 , 41 - 41
Created: 29-07-2024 11:59 by Analyst1, Modified: 02-09-2024 16:25 by Analyst1
Area: 55   0.26%

4.
"Have, I put if I have a key to open the drone (Note: Key to open) to receive my package. I think this is nice, it depends on the location, for instance. I think it's very important to have it to avoid that somebody else will not take your package."
Code: ● Verification   Weight score: 0
I11 , 38 - 38
Created: 26-07-2024 15:59 by Analyst2, Modified: 08-08-2024 9:53 by Analyst1
Area: 247   1.23%

5.
"I like to type the key to open the package."
Code: ● Verification   Weight score: 0
I11 , 74 - 74
Created: 29-07-2024 11:38 by Analyst1, Modified: 08-08-2024 9:53 by Analyst1
Area: 43   0.21%

6.
"This should also have the key to open as it is a medicine. I think it's something very important. So it Should Have."
Code: ● Verification   Weight score: 0

I11 , 102 - 102
Created: 26-07-2024 16:10 by Analyst2, Modified: 08-08-2024 9:53 by Analyst1
Area: 116 0.58%

7.
"it will first identify that I'm the recipient (Note: identification with me)"
Code: ● Verification Weight score: 0
I10 , 23 - 23
Created: 26-07-2024 15:08 by Analyst1, Modified: 02-09-2024 15:53 by Analyst1
Area: 76 0.35%

8.
"I would imagine if this service really exist, you will have multiple recipients everywhere and there is a possibility that you will receive the wrong package, if the drone identifies you to another person by mistake. For this type of delivery service, you can always compare it to the current ones like supermarket deliveries. For instance, it is not like after three days of your order but within like one hour. Normally, when they (delivery personal) try to approach you, they will first check your indication if you are the right person. I'm not sure if the identification should happen during when it is on the air or when it is landing."
Code: ● Verification Weight score: 0
I10 , 27 - 27
Created: 26-07-2024 16:35 by Analyst2, Modified: 02-09-2024 15:53 by Analyst1
Area: 641 2.92%

9.
"After identified, I should be able to confirm with the drone that I already get the goods. It can then leave a general confirmation that I got the stuff and a confirmation that it can leave."
Code: ● Verification Weight score: 0
I10 , 43 - 43
Created: 26-07-2024 15:14 by Analyst1, Modified: 02-09-2024 16:23 by Analyst1
Area: 190 0.86%

10.
"Let's think. I guess also, yeah, it's maybe a bit in between a must have and it should have. Some form of how do I put it ---? Identification control, like when I order something at home, I know that it's meant for me because, well, they're ringing my doorbell (Note: check-in that I am the recipient). I would hope it's for me, but when I'm in a park, I'm imagining it's a great summer day. There's a ton of people around. I, for one, would like to know that it's indeed meant for me, but also the other way around if it happens to end up with someone else it would also be great if there's some confirmation, having a bit of a check in there because when you need to ---. In that park, you see, you see a drone going down like 50 meters away from you, and someone just taking your items being like: Oh well, I could use a snack during my park visit that I'm missing something there. So it's not necessarily a Must Have, but it's a bit on a Should Have."
Code: ● Verification Weight score: 0
I6 , 53 - 53
Created: 29-07-2024 11:10 by Analyst2, Modified: 01-08-2024 13:50 by Analyst1
Area: 954 2.34%

11.
"if I could know there was as little as possible being saved because especially when I'm thinking that I could have a QR code to throw a scan before it's delivered, that would mean that I don't need personal data to be saved in any way"
Code: ● Verification Weight score: 0
I6 , 78 - 78
Created: 29-07-2024 11:12 by Analyst2, Modified: 02-08-2024 11:08 by Analyst1
Area: 234 0.57%

12.

"just having that extra check in when the device is there already, I think that is extremely valuable."

Code: ● Verification Weight score: 0
I6 , 91 - 91
Created: 25-07-2024 18:20 by Analyst1, Modified: 01-08-2024 13:50 by Analyst1
Area: 101 0.25%

13.

"when I scan my QR code there I have the opportunity to just go through the questions and these questions can also be very confirming like: Hey, you just all this medication. Are you OK? Did you receive the right medication? Are you helped enough with this? Do you need further assistance?"

Code: ● Verification Weight score: 0
I6 , 100 - 100
Created: 25-07-2024 18:30 by Analyst1, Modified: 01-08-2024 13:50 by Analyst1
Area: 288 0.71%

14.

"I'm just thinking of maybe there could have like an extra confirmation (Note: extra check-in) of being the right person that can be done a bit more accurately than would be necessary or nice to have"

Code: ● Verification Weight score: 0
I6 , 100 - 100
Created: 25-07-2024 18:33 by Analyst1, Modified: 01-08-2024 13:50 by Analyst1
Area: 198 0.49%

15.

"Maybe I should stand up for it to sense me easily."

Code: ● Verification Weight score: 0
I5 , 10 - 10
Created: 25-07-2024 15:50 by Analyst1, Modified: 02-09-2024 16:02 by Analyst1
Area: 50 0.12%

16.

"I want to have some sort of a secret code. Like, let me just write it here like a secret code that both of us know."

Code: ● Verification Weight score: 0
I5 , 90 - 90
Created: 25-07-2024 16:16 by Analyst1, Modified: 02-09-2024 16:02 by Analyst1
Area: 115 0.28%

17.

"If you confirm from your side like: Hey this is me, then that also gives that signal."

Code: ● Verification Weight score: 0
I4 , 49 - 49
Created: 25-07-2024 11:44 by Analyst1, Modified: 02-09-2024 15:58 by Analyst1
Area: 85 0.38%

18.

"Hey, I've received it. If that doesn't happen, then at least they know, like, hey it's not finished yet."

Code: ● Verification Weight score: 0
I4 , 53 - 53
Created: 25-07-2024 11:45 by Analyst1, Modified: 02-09-2024 15:58 by Analyst1
Area: 104 0.46%

19.

"If the delivery person delivers the food, they have to ask you the code. Otherwise, they cannot confirm they gave it. I think that just adds an extra element of safety. Especially as the recipient, it gives you this feeling like oh, they will not fuck me over because I have the code."

Code: ● Verification Weight score: 0
I4 , 57 - 57
Created: 25-07-2024 11:46 by Analyst1, Modified: 02-09-2024 15:58 by Analyst1
Area: 284 1.26%

20.

"Some signal that it knows how to find me - location sharing, face detection). I think without those, it will probably be hard to get people to feel happy and give them a safe feeling about the process."

Code: ● Verification Weight score: 0
I4 , 81 - 81
Created: 29-07-2024 12:09 by Analyst2, Modified: 02-09-2024 15:58 by Analyst1
Area: 201 0.89%

21.

"Analyst1 12:26
Okay. With regards to control, do you want to control the drone?

Participant 12:35
When I got my package then I want to say like, oh, you can fly away now and then I can press like, you can fly away and I confirm I got the package and everything set.

Participant 12:52
Okay, and why do you want to have the control?

Participant 12:56
What if I didn't finish the receiving process? If it starts to fly, then I will lose my package."

Code: ● Verification Weight score: 0
I3 , 76 - 83
Created: 29-07-2024 12:22 by Analyst2, Modified: 02-09-2024 15:55 by Analyst1
Area: 464 2.54%

22.

"I think this is the Must Have for any delivery. Just to check whether, okay, this is my order, already complete, or something missing. That kind of thing. It will be very bad if the drones comes and I took the package. The drone flies away but, oh no, this is not my package. It's not fun then."

Code: ● Verification Weight score: 0
I1 , 38 - 38
Created: 29-07-2024 15:28 by Analyst2, Modified: 02-09-2024 16:29 by Analyst1
Area: 294 1.18%

23.

"Well, nice. All right, cool. I think they are related, where to pick up and how to pick up. I could imagine there could be multiple ways to get this information at least for me. An app on my phone, for example, where to pick up, like how to pick up some kind of like, just typical user interface on the screen. But it could be interesting if we could have some kind of projection from the drones, like, the area that it will land on the park. Projection from the sky, highlighting the area. For example, target color, this is where you will get your package. And when I approach the area, it will change the color the shape, like, Oh, this is right or not. Let's say I'm on the right position or right angle to get the package, it will somehow change the light or sound or whatever. And how to pick up: just wave. It's like, hello, hello (showing hand waving). The basic idea is to just communicate simply through the app, the other is through projection. There should be a way for me to indicate this is where I am in the park. It could be a gesture or maybe I need to perform some unique gesture so the drone can somehow detect,

oh, this the recipient that I need to approach and I need to fly closer to the person and that kind of thing."

Code: ● Verification   Weight score: 0
I1 , 50 - 50
Created: 29-07-2024 15:30 by Analyst2, Modified: 02-09-2024 16:29 by Analyst1
Area: 1240   4.99%

24.

"There should be a way for me to indicate this is where I am in the park. It could be a gesture or maybe I need to perform some unique gesture so the drone can somehow detect,"

Code: ● Verification   Weight score: 0
I1 , 50 - 50
Created: 24-07-2024 13:00 by Analyst1, Modified: 02-09-2024 16:29 by Analyst1
Area: 174   0.70%

25.

"Wave (my hand) to turn to indicate where am I."

Code: ● Verification   Weight score: 0
I1 , 98 - 98
Created: 24-07-2024 13:10 by Analyst1, Modified: 02-09-2024 16:29 by Analyst1
Area: 46   0.19%

3.4.5. Recognise own drone

1.

"I'm more certain about: this (drone) is not for me, it's not for this group but it is for the other group."

Code: ● Recognise own drone   Weight score: 0
I12 , 153 - 153
Created: 26-07-2024 13:15 by Analyst2, Modified: 02-09-2024 16:25 by Analyst1
Area: 106   0.50%

2.

"There is a number on the drones body, also showing number 27, for you to identify, and then see if it is the right one. You can confirm things like that. In any way, I think there's a process of identification with each other, between the user and the drone."

Code: ● Recognise own drone   Weight score: 0
I10 , 35 - 35
Created: 26-07-2024 16:38 by Analyst2, Modified: 02-09-2024 16:23 by Analyst1
Area: 258   1.17%

3.

"I can be a little bit more sure about what it's flying around, is it for me. Even as a recipient, if I go to a scenario (where) there are many drones around, maybe just annoying or kind of weird to feel all the time that there is someone above your head. I'm telling you about the scenario that more people are expecting a delivery in a public park for example."

Code: ● Recognise own drone   Weight score: 0
I8 , 30 - 30
Created: 29-07-2024 9:43 by Analyst2, Modified: 02-09-2024 16:30 by Analyst1
Area: 361   1.33%

4.

"I(there) some signaling or sign on the drone that I actually know it's from the company (Note: some signaling on the drone to confirm that this is the company I ordered something from) that I that I ordered something from so that I actually have that visual recognition that it is something that I ordered from.
00:05:56 Analyst1:
And why do you want to have this visual recognition?
00:06:02 Participant:

It's especially the environment in which I picture myself to be sitting, because there is a lot going to be happening in that situation. So I want to have that certainty and taking away a bit of that uncertainty that I was just describing earlier. I want to know, have that extra layer of information: What is actually happening? Ohh right. I see it's just the same as for ordering when I order Domino's and I see someone with dominoes packages walking up there's that, there's that certainty in there that I know what to expect."

Code: ● Recognise own drone   Weight score: 0
I6 , 37 - 41
Created: 29-07-2024 11:08 by Analyst2, Modified: 01-08-2024 13:50 by Analyst1
Area: 947   2.32%

5.

"I'm imagining this, it's quite important to have. You're thinking onto the recipient. As a recipient, I would like to know if the drone is here for me."

Code: ● Recognise own drone   Weight score: 0
I5 , 89 - 89
Created: 29-07-2024 11:47 by Analyst2, Modified: 02-09-2024 16:02 by Analyst1
Area: 151   0.37%

6.

"Oh wow yeah. I mean it's different because while you're in the emergency situation, right? So you want to know if help is there or not and this is more important in emergency situation. You have to know if it's here or if it's just someone else's snack because you're going to be ready for it then. I think it's important for people to know that help is already there. Than if it's a snack, I think it's less important for people to know, like is it my snack or not like it could be good, but it's not extremely necessary. But for emergencies, I think it is."

Code: ● Recognise own drone   Weight score: 0
I5 , 179 - 179
Created: 29-07-2024 11:55 by Analyst2, Modified: 02-09-2024 16:02 by Analyst1
Area: 558   1.37%

7.

"Wait, I think I put it here (Note: Visual indication that the drone is my order - e.g. number or color that matches something in my app) as well. Some other way to do is to, for example, label them in some way, by giving them like a color. Then you know, like, oh, the one with the green lights is mine or maybe it has a normal plate on it or name."

Code: ● Recognise own drone   Weight score: 0
I4 , 73 - 73
Created: 29-07-2024 12:08 by Analyst2, Modified: 02-09-2024 15:58 by Analyst1
Area: 348   1.54%

8.

"Because speed is of the essence, a clear indicator is also crucial (Note: Clear outside indicator that this drone is 'your' drone). (When) you see a drone come your way, you immediately know like, Oh, this is mine, or this is not mine and you can act quickly. Yeah, I think those three are crucial."

Code: ● Recognise own drone   Weight score: 0
I4 , 119 - 119
Created: 29-07-2024 12:13 by Analyst2, Modified: 02-09-2024 15:58 by Analyst1
Area: 298   1.32%

9.

"I imagine we'll have a lot of drones flying through the sky, then I want to know which one is mine."

Code: ● Recognise own drone   Weight score: 0
I3 , 36 - 36
Created: 24-07-2024 14:47 by Analyst1, Modified: 02-09-2024 15:55 by Analyst1
Area: 99   0.54%

10.
"think it would be good if I know what kind of drone will arrive as there could be multiple types of drones. Colors. That is the Should Have. With Could Have: assuming if the drones are able to deliver more than one packages at the same time, so which one is mine? Let's say in the future, it's becoming a reality and normal that there are so many drones, then you are like, which one is my drone?"

Code: ● Recognise own drone   Weight score: 0
I1 , 38 - 38
Created: 29-07-2024 15:29 by Analyst2, Modified: 02-09-2024 16:29 by Analyst1
Area: 396   1.59%

3.4.6. Purpose

1.
"I think it's important to show the emergency purpose of the drone (Note: showing the emergency purpose). So basically, the last question you ask, what is this drone for? It's important (for emergency) because I (as a bystander) could potentially avoid any (collision). It's flying in the sky, at least people are aware of this as emergency or if the drone needs help, they could respond faster."

Code: ● Purpose   Weight score: 0
I12 , 175 - 175
Created: 29-07-2024 12:14 by Analyst1, Modified: 02-09-2024 16:25 by Analyst1
Area: 394   1.85%

2.
"If the emergency is too critical, it's like life or death, it could have a sign or something on the drone."

Code: ● Purpose   Weight score: 0
I11 , 178 - 178
Created: 29-07-2024 11:49 by Analyst1, Modified: 08-08-2024 9:53 by Analyst1
Area: 106   0.53%

3.
"First I need to know its purpose (Note: its purpose why it comes here (voice/visual logos/lights)). Why did it come here?"

Code: ● Purpose   Weight score: 0
I10 , 107 - 107
Created: 26-07-2024 15:29 by Analyst1, Modified: 02-09-2024 16:23 by Analyst1
Area: 121   0.55%

4.
"If they could already tell what's inside the package and just help the bystanders to identify that the drone is not delivering something emergency or something dangerous."

Code: ● Purpose   Weight score: 0
I10 , 125 - 125
Created: 26-07-2024 15:33 by Analyst1, Modified: 02-09-2024 16:23 by Analyst1
Area: 170   0.77%

5.
"For a bystander, it will still be a drone. If it's carrying a delivery package, then there might be curiosity for the first visual interaction, like, oh, it's delivery drone (Note: purpose of the drone as delivery)."

Code: ● Purpose   Weight score: 0
I9 , 175 - 175
Created: 29-07-2024 9:07 by Analyst2, Modified: 08-08-2024 9:17 by Analyst1
Area: 215   0.82%

6.

"I think it will see the delivery in some way. There's going to be some package attached to the drone. So that's how you will know that it's a drone for delivery. There should be a clear marking of like a delivery drone versus a drone for personal use, because this drone could have done the delivery and is now returning back. It would be nice to know that okay, this is a delivery drone, rather than someone's just flying the drone."

Code: ● Purpose   Weight score: 0
I9 , 179 - 179
Created: 29-07-2024 9:07 by Analyst2, Modified: 08-08-2024 9:17 by Analyst1
Area: 433   1.65%

7.

"You wouldn't understand why this drone is just randomly like flying. Is someone recording the area or like, what's happening? Is the drone trying to do something? There will be no context other than a drone flying."

Code: ● Purpose   Weight score: 0
I9 , 183 - 183
Created: 26-07-2024 14:58 by Analyst1, Modified: 08-08-2024 9:17 by Analyst1
Area: 214   0.82%

8.

"As a bystander, it's just doing delivery. The purpose of the delivery doesn't affect the bystander."

Code: ● Purpose   Weight score: 0
I9 , 215 - 215
Created: 26-07-2024 15:00 by Analyst1, Modified: 08-08-2024 9:17 by Analyst1
Area: 99   0.38%

9.

"the purpose of the drone was more to do with the distinction between a delivery drone versus a personnel. In this sense, it should still indicate that it's a delivery drone. But then, does there need to be a further distinction of like an emergency delivery versus a regular delivery? That is unnecessary."

Code: ● Purpose   Weight score: 0
I9 , 223 - 223
Created: 26-07-2024 15:00 by Analyst1, Modified: 08-08-2024 9:17 by Analyst1
Area: 305   1.16%

10.

"The moment it (the drone) approaches, maybe they can have some color like, these (delivery) drones of every company can have or like (how) the (vehicle design of) Thuisbezorgd has. When they (drones) are reaching (the park), maybe they can have some colors or some kind of specific logo that I can know (the purpose). For example, like drone Uber. At least you know, that maybe it's not an individual (private drone) taking photos, playing with the people in the park, but someone knows that (drone) comes, lands and leaves."

Code: ● Purpose   Weight score: 0
I8 , 158 - 158
Created: 29-07-2024 10:29 by Analyst2, Modified: 02-09-2024 16:30 by Analyst1
Area: 524   1.93%

11.

"some signaling or sign on the drone that I actually know it's from the company (Note: some signaling on the drone to confirm that this is the company I ordered something from) that I that I ordered something from so that I actually have that visual recognition that it is something that I ordered from."

Code: ● Purpose   Weight score: 0
I6 , 37 - 37
Created: 25-07-2024 16:57 by Analyst1, Modified: 01-08-2024 13:50 by Analyst1
Area: 302   0.74%

12.
"I suppose what would, help I mentioned of course the signaling visually already on the snacks drone, I think having some information only that it is a medical thing in some way. I think that would already help a lot. Also in signaling to other people as they're going into the bystander element there but also to other people if they know: Oh right, that is a medicine service delivery, versus that also gives a certain sense of urgency (Note: urgency/medical signaling) in how they interact with that drone. If it would accidentally stop at their place."

Code: ● Purpose Weight score: 0
I6 , 112 - 112
Created: 29-07-2024 11:25 by Analyst2, Modified: 01-08-2024 13:50 by Analyst1
Area: 554 1.36%

13.
"there is a certain recognizability in that pattern of what you see and what you can expect from what you're seeing."

Code: ● Purpose Weight score: 0
I6 , 153 - 153
Created: 26-07-2024 11:28 by Analyst1, Modified: 01-08-2024 13:50 by Analyst1
Area: 115 0.28%

14.
"if I'm a bystander that I know that I haven't ordered anything, then the first I think reaction is probably thinking, OK, what's the purpose of this drone?"

Code: ● Purpose Weight score: 0
I5 , 11 - 11
Created: 25-07-2024 16:00 by Analyst1, Modified: 02-09-2024 16:02 by Analyst1
Area: 155 0.38%

15.
"So I feel like the purpose should be less---. The announcement should be made less intrusive. Oh, I wonder how. I mean. I think it should be visual. Instead of audio, which is more ambient. It all depends on if you're going to like, put a flashlight. It's not going to be that ambient, but visual---. So this will be more for people are curious about what the drone is there for. So then if they're looking for the purpose of the drone, they are able to access it. And for the ones that don't care, they're also not bothered by the drone just screaming. I'm here to deliver here, you know, that's going to be annoying."

Code: ● Purpose Weight score: 0
I5 , 88 - 88
Created: 29-07-2024 11:46 by Analyst2, Modified: 02-09-2024 16:02 by Analyst1
Area: 618 1.51%

16.
"Yeah. I'm going to move these down (Note: presence - beeping with a low frequency, Note: purpose - Less intrusive, visual, and ambient). And I think I'm also going to have this. But I'm going to put it in Must Have, because if people is having allergic to---."

Code: ● Purpose Weight score: 0
I5 , 157 - 157
Created: 29-07-2024 11:53 by Analyst2, Modified: 02-09-2024 16:02 by Analyst1
Area: 259 0.63%

17.
"presence and purpose which are similar to what a recipient wants to know, but also for bystander so that they are informed about the intentions.
00:42:07 Participant:
Yeah. Exactly, so that they are informed about the intentions"

Code: ● Purpose Weight score: 0
I5 , 208 - 210
Created: 29-07-2024 11:59 by Analyst2, Modified: 02-09-2024 16:02 by Analyst1
Area: 228 0.56%

18.
"I think these two just goes for general for everybody like it should, you know, of course the drone should let you know that it's in your vicinity and then its purpose is also really important because I want to know if everybody will be recorded and whatnot like this. Just for like privacy and safety concerns."

Code: ● Purpose Weight score: 0
I5 , 223 - 223
Created: 29-07-2024 12:00 by Analyst2, Modified: 02-09-2024 16:02 by Analyst1
Area: 311 0.76%

19.
"I'm more just thinking of from a bystander perspective, I feel like I do not need to participate in this in whatever way, so I don't really care. I just need to know what it's here for and that's it."

Code: ● Purpose Weight score: 0
I5 , 243 - 243
Created: 29-07-2024 12:01 by Analyst2, Modified: 02-09-2024 16:02 by Analyst1
Area: 199 0.49%

20.
"Maybe I can explain them as I go. Something it should have is some clear indication that it is a delivery drone (Note: Some clear indication that its a delivery drone). Maybe for myself aside like: Oh, it's this is just an Uber Delivery drone."

Code: ● Purpose Weight score: 0
I4 , 135 - 135
Created: 29-07-2024 12:14 by Analyst2, Modified: 02-09-2024 15:58 by Analyst1
Area: 243 1.07%

21.
"I think maybe on a broader level, some people might get aggressive with drones. It may be good if it has some indication for them."

Code: ● Purpose Weight score: 0
I4 , 163 - 163
Created: 25-07-2024 13:04 by Analyst1, Modified: 02-09-2024 15:58 by Analyst1
Area: 130 0.57%

22.
"I want to know if it's emergency. So if it's not, also let me know. It has to distinguish non emergency and emergency.

Analyst1 25:18
Yeah. How would you like to know this information?

Participant 25:22
I would keep it to non emergency just like normal, and the emergency one has some alarm."

Code: ● Purpose Weight score: 0
I3 , 173 - 177
Created: 29-07-2024 12:25 by Analyst2, Modified: 02-09-2024 15:55 by Analyst1
Area: 307 1.68%

23.
"Yes, I must want to know the purpose of the drone (Note: commercial bands) because the drone can do a lot of things either the commercial activity or shoot videos during the travels or even engaging in the war. So, if I was doing the picnic in the park, I know it has to be like a commercial drone flying to me, not some of the other things. The commercial brand drones are expected to not harm the others as it has been (issued) certificates by the government. So this is the most important things. The others like friendly, light (coloured) looking (Note: Friendly light outlook) or the voice output (Note: Voice output) also tell me like what the drone is

doing and what activities he's going to do. For example, drone could have a voice that says like your delivery or your pizza or something else and if it also emits some friendly lights or have good looking then I will be sure like, okay this is a safe drone flying to me. So it is also linked to this part which is Won't Have, like related to each other, so it won't have like a weapon like outlook (Note: Weapon outlook) or there is no info on the body of drone (Note: No info on the body of drone). So, (lets say), there is a drone flying to me and I have no idea what the drone is like and it's all black, I'm like a recipient, I will be frightened with what it is."

Code: ● Purpose   Weight score: 0
I2 , 31 - 31
Created: 29-07-2024 14:43 by Analyst2, Modified: 02-09-2024 16:26 by Analyst1
Area: 1327   5.62%

24.

"I think we only need to know whether it is delivering a package or emergency or involving in some of the other activities. I don't think it needs to go further to give any specific information because it raises questions and problems."

Code: ● Purpose   Weight score: 0
I2 , 100 - 100
Created: 29-07-2024 14:53 by Analyst2, Modified: 02-09-2024 16:26 by Analyst1
Area: 234   0.99%

25.

"I'm relaxing on the park and suddenly (I see) flying object (and think) like what is this thing and what can it do?"

Code: ● Purpose   Weight score: 0
I1 , 158 - 158
Created: 24-07-2024 13:25 by Analyst1, Modified: 02-09-2024 16:29 by Analyst1
Area: 115   0.46%

4. Drone design solutions to address uncertainty in HDI

4.1. Use case dependent

1.
"it's a scenario where it is actually nice to have some involvement from someone else who is there, but at the same time you don't want everyone around there to be involved"
Code: ● Use case dependent Weight score: 0
I6 , 177 - 177
Created: 26-07-2024 11:33 by Analyst1, Modified: 01-08-2024 13:50 by Analyst1
Area: 171 0.42%

2.
"if it's a snack, I think it's less important for people to know, like is it my snack or not like it could be good, but it's not extremely necessary. But for emergencies, I think it is."
Code: ● Use case dependent Weight score: 0
I5 , 179 - 179
Created: 25-07-2024 16:35 by Analyst1, Modified: 02-09-2024 16:02 by Analyst1
Area: 184 0.45%

3.
"I want to know if it's emergency. So if it's not, also let me know. It has to distinguish non emergency and emergency."
Code: ● Use case dependent Weight score: 0
I3 , 173 - 173
Created: 24-07-2024 15:02 by Analyst1, Modified: 02-09-2024 15:55 by Analyst1
Area: 118 0.65%

4.
"when you switch the use case then the information (required) actually changes with the role."
Code: ● Use case dependent Weight score: 0
I1 , 194 - 194
Created: 24-07-2024 13:30 by Analyst1, Modified: 02-09-2024 16:29 by Analyst1
Area: 92 0.37%

4.1.1. Appearance - non-emergency

1.
"The appearance can be different based on what is being delivered."
Code: ● Appearance - non-emergency Weight score: 0
I12 , 89 - 89
Created: 26-07-2024 12:54 by Analyst2, Modified: 02-09-2024 16:25 by Analyst1
Area: 65 0.31%

2.
"I think I would have the same as I mentioned before. I don't think I would have different. Yeah, but if I don't know what is this drone for then I don't have an expectation basically."
Code: ● Appearance - non-emergency Weight score: 0
I12 , 161 - 161
Created: 26-07-2024 13:19 by Analyst2, Modified: 02-09-2024 16:25 by Analyst1
Area: 183 0.86%

3.
"We should have (the drone) more distinguishable, so people around also know that this is for a medical reason and it's not like a bag of chips or my Amazon (package)."
Code: ● Appearance - non-emergency Weight score: 0
I8 , 132 - 132
Created: 26-07-2024 13:52 by Analyst1, Modified: 02-09-2024 16:30 by Analyst1

Area: 166 0.61%

4.

"The moment it (the drone) approaches, maybe they can have some color like, these (delivery) drones of every company can have or like (how) the (vehicle design of) Thuisbezorgd has."

Code: ● Appearance - non-emergency Weight score: 0
I8 , 158 - 158
Created: 26-07-2024 14:04 by Analyst1, Modified: 02-09-2024 16:30 by Analyst1
Area: 180 0.66%

5.

"When they (drones) are reaching (the park), maybe they can have some colors or some kind of specific logo that I can know (the purpose). For example, like drone Uber. At least you know, that maybe it's not an individual (private drone) taking photos, playing with the people in the park, but someone knows that (drone) comes, lands and leaves."

Code: ● Appearance - non-emergency Weight score: 0
I8 , 158 - 158
Created: 26-07-2024 14:04 by Analyst1, Modified: 02-09-2024 16:30 by Analyst1
Area: 343 1.27%

6.

"I think appearance is already a large part. There's, of course, most delivery services have a certain color, like when you see pink, when you see orange cycling around that already signals a lot on what's actually happening. So having that level of signaling is already some form that would fit in this Must Have. That's also why I put it in as a Must Have because I think it is a very low threshold thing to actually include, but it does make a huge difference being the recipients."

Code: ● Appearance - non-emergency Weight score: 0
I6 , 45 - 45
Created: 29-07-2024 11:09 by Analyst2, Modified: 01-08-2024 13:50 by Analyst1
Area: 483 1.18%

7.

"Same thing now happens when I see a new company pop up (Note: some signaling on the drone to confirm that this is the company I ordered something from). You see a new bright color that somehow enters enters your space. But the moment you start associating a color, a name to a certain action happening. Then that, and certainly as bystander, is way lower when I actually have that consistency of seeing that color related to these actions, then, if a different colored drone would show up, I would be confused. There would be an uncertainty there. If, for example, I would see after being used to this seeing one of those emergency medicines drones for the first time, I would be like, wait, what is going on here? So this is a different situation that would bring quite a bit of uncertainty, because that doesn't fade to the background because it's not something we're used to.
00:38:41 Analyst1:
Yeah, and so you suggest, like all the grocery drones, to have a similar color template?
00:38:48 Participant:
Yes and no. Like I said before, there's always going to be a ton of different brands trying to enter this space. But they always have like waves, where you see a few first ones sprinkled around, next thing you know, that's all you see and there is a certain recognizability in that pattern of what you see and what you can expect from what you're seeing. And then sometimes a new color and enters a space, a new brand shows up. And then you have that curve again and then it steadies up and sometimes perhaps the color will disappear. First, we'll start seeing a few less of them, the advertisements will stop disappearing, and then they'll be off."

Code: ● Appearance - non-emergency Weight score: 0
I6 , 149 - 153
Created: 29-07-2024 11:29 by Analyst2, Modified: 02-08-2024 15:48 by Analyst1
Area: 1669 4.09%

8.
"Analyst1 39:23
And how do you want to see this indication?

Participant 39:31
There's multiple ways, either through a display, maybe it has some costume, maybe it's on the box (package attached).

Analyst1 39:38
Display you mean by attached to the drone or on the ground or on your phone?

Participant 39:44
I think attached to the drone.

Analyst1 39:49
[Revised the three ways: display, costume, box]

Participant 40:00
Yeah, maybe a flag on it or something. Just any visual cue, basically."
Code: ● Appearance - non-emergency Weight score: 0
I4 , 136 - 147
Created: 29-07-2024 12:15 by Analyst2, Modified: 02-09-2024 15:58 by Analyst1
Area: 534 2.36%

9.
"Yeah. It could have unique shapes or aesthetics in the drone that represent the purpose. We know how the ambulance looks like, we know how the brandweerauto (fire fighter truck) looks like. If we have those kinds of universally accepted or emergency aesthetics of the delivery drones, more into the physical features, then we don't need app on the phone.

Analyst1 49:48
Do you want to have an app to know the information or do you want it to the way the drone looks?

Participant 50:02
Ideally, only from the looks of the drones. But initially, we need to have some kind of educational awareness for the public and with the governmental logos."
Code: ● Appearance - non-emergency Weight score: 0
I1 , 162 - 166
Created: 29-07-2024 15:38 by Analyst2, Modified: 02-09-2024 16:29 by Analyst1
Area: 658 2.65%

4.1.2. HMIs - emergency

1.
"The drone could have a specific light, like the ambulances. The drone could have a specific color showing that it's critical package. So as to call attention, that it's important."
Code: ● HMIs - emergency Weight score: 0
I11 , 182 - 182
Created: 29-07-2024 11:50 by Analyst1, Modified: 08-08-2024 9:53 by Analyst1
Area: 179 0.89%

2.
"Yeah, actually I want to know it (Note: know if i need to gives space/help it (voice like ambulances/ voice of sentences)).

Analyst1 46:25

And how do you want to know it?

Participant 46:34
The most instant one will be voice, like ambulance.

Analyst1 46:43
Like a siren you mean?

Participant 46:45
Yeah

Analyst1 46:49
If there is siren, does it mean you want to help or does it mean you need to give space?

Participant 46:57
I need to give space. If I want to help, probably another voice. If it needs help then I would expect voice of sentences rather than just sounds."

Code: ● HMIs - emergency   Weight score: 0
I10 , 158 - 170
Created: 26-07-2024 17:00 by Analyst2, Modified: 02-09-2024 16:23 by Analyst1
Area: 617   2.81%

3.

"if it's like completely emergency, having sound also for people to notify that something is happening."

Code: ● HMIs - emergency   Weight score: 0
I8 , 116 - 116
Created: 26-07-2024 13:50 by Analyst1, Modified: 02-09-2024 16:30 by Analyst1
Area: 102   0.38%

4.

"Participant 30:49
Could have, it's more like a siren, kind of a noise, just like the audio (Note: siren to move people). Obviously, how the emergency vehicles when they are the ambulance siren or something saying that it's an emergency."

Code: ● HMIs - emergency   Weight score: 0
I7 , 118 - 121
Created: 29-07-2024 10:46 by Analyst2, Modified: 02-09-2024 16:31 by Analyst1
Area: 237   0.92%

5.

"Participant 44:41
Is there any way I can help and if it is like announcing or something? (Note: Announcement must be loud and clear, so that i can know if i am of any help) In this case, it can be like an audio announcement saying that, hey, this person is stuck in this place, this person needs help. The audio announcement should be loud and clear saying that this person is in an emergency and if you're in the same zone, can you go and check it out something. Because, if in the case given, not everyone can be in a medical emergency that they can be helpful. They can do something themselves. If I'm nearby and but I am not aware of the situation and I'm in the other part of the park or somewhere and if I get to know with the announcement I can rush to the place and I can help him. Being helpful."

Code: ● HMIs - emergency   Weight score: 0
I7 , 182 - 183
Created: 29-07-2024 10:52 by Analyst2, Modified: 02-09-2024 16:31 by Analyst1
Area: 805   3.14%

6.

"For the Could Have, the siren (Note: Siren).

Analyst1 49:23
What does it tell you, the siren?

Participant 49:26
It's more like an ambulance siren saying that there is this person located in this particular position because even the drone announcing that there is someone needing the help but no one knows where. But the siren approaching him, is easier."

Code: ● HMIs - emergency Weight score: 0
I7 , 193 - 197
Created: 29-07-2024 10:54 by Analyst2, Modified: 02-09-2024 16:31 by Analyst1
Area: 369 1.44%

7.

"thinking about that trauma helicopter a bit further, perhaps I would even prefer if in the second scenario, if it would land in an approximate area in which I am, so it doesn't just show up to me individually, but it lands in the area gives you a pop-up: Hey, I'm close to you, please walk over so that you don't have the personal interactions happening before I can actually get to the drone."

Code: ● HMIs - emergency Weight score: 0
I6 , 125 - 125
Created: 26-07-2024 11:20 by Analyst1, Modified: 01-08-2024 13:50 by Analyst1
Area: 393 0.96%

8.

"I have just been thinking about mobile devices is, that drone is landing in your vicinity, that you get a pop up like, hey, an emergency delivery drone is landing in your vicinity. Please stand by, someone is need of medication. If no one approaches the drone, please check around if there is something going on there. Something like that would have some relevance because say something actually did happen to the recipients. Who ordered these emergency medication? Maybe they were really in need of that emergency medication. So there's some relevance of bystanders there. But at the same time, how broad would that be sent out? What is the relevance relevant parameter of actually getting that notification?"

Code: ● HMIs - emergency Weight score: 0
I6 , 181 - 181
Created: 26-07-2024 11:35 by Analyst1, Modified: 01-08-2024 13:50 by Analyst1
Area: 709 1.74%

9.

"Yeah. So I have the pop up, I have that here (Note: pop-up of someone in vicinity in need of help). That's a bit more specific because the pop up is specifically linked to a mobile device that's a bit more should have than a must have because there's also other ways of communicating that. There could also indeed be for example, I don't know. A screen on that drone saying: Hey, I have an emergency delivery for so and so. So there's also other ways of having that interaction, but in the relevance and the urgency, especially if you consider that code of conduct and that way of getting that familiarity with that, then it would also make sense to get a pop up like that and it wouldn't surprise you to see a pop up like that when you see an emergency drone land. So you also would have this clarity of the expectancy for you?"

Code: ● HMIs - emergency Weight score: 0
I6 , 197 - 197
Created: 29-07-2024 11:35 by Analyst2, Modified: 02-08-2024 13:54 by Analyst1
Area: 828 2.03%

10.

"I think they're all going to stay the same, but I'm just going to change all these expressions to be more intrusive."

Code: ● HMIs - emergency Weight score: 0

I5 , 165 - 165
Created: 25-07-2024 16:32 by Analyst1, Modified: 02-09-2024 16:02 by Analyst1
Area: 116   0.28%

11.
"I think it's OK to keep them the same. I'll probably. Yeah. Now I take back the intrusive bit. It's the same. Because, the way that I understand ambulance is. That I feel like they have all these loud noises are because the other cars should dodge them, but I think it's not necessary for drones. Drones don't need it, there is no busy traffic in the air, I'm guessing. So that wouldn't be necessary. And also I think it is a good thing to do to protect people's privacy even though they are having an emergency medical situation in the public space. They should also be given the choice of if they want to announce it or not. So I think by a drone announcing it for them is not necessarily always a good thing."
Code: ● HMIs - emergency   Weight score: 0
I5 , 171 - 171
Created: 29-07-2024 11:54 by Analyst2, Modified: 02-09-2024 16:02 by Analyst1
Area: 711   1.74%

12.
"I'm thinking if there's a real difference between these two. For example, flashing lights or something would be would be nice. I think that is a clear indication, but it would maybe signal just the extra bit of emergency towards bystanders."
Code: ● HMIs - emergency   Weight score: 0
I4 , 177 - 177
Created: 25-07-2024 13:05 by Analyst1, Modified: 02-09-2024 15:58 by Analyst1
Area: 240   1.06%

13.
"Participant 20:01
If it's emergency, I would like if it has alarm. I think drone itself, it should have an alarm like those for the ambulance or on the (police) car. This is for emergency thing so the other people will be also aware of it, will be more conscious about it. For example, give the space and something like that."
Code: ● HMIs - emergency   Weight score: 0
I3 , 126 - 127
Created: 29-07-2024 12:23 by Analyst2, Modified: 02-09-2024 15:55 by Analyst1
Area: 326   1.79%

14.
"I would keep it to non emergency just like normal, and the emergency one has some alarm."
Code: ● HMIs - emergency   Weight score: 0
I3 , 177 - 177
Created: 24-07-2024 15:02 by Analyst1, Modified: 02-09-2024 15:55 by Analyst1
Area: 88   0.48%

15.
"No, the alarm has to be the old way (Note: ambulance alarm), not only when it is landing or something. When it is flying and also carrying the alarm, the landing location and signal (Note: Landing location, signal (lights, audio, projection)) just for us to provide help if it's necessary. For example, it decided to land somewhere where the older (recipient) cannot reach, or they're dying, I can help to deliver it to them."
Code: ● HMIs - emergency   Weight score: 0
I3 , 205 - 205
Created: 29-07-2024 12:28 by Analyst2, Modified: 02-09-2024 15:55 by Analyst1
Area: 425   2.33%

16.

"If it is with alarm, I already know it is emergency. Then I will start to look around who is exactly the person (who) ordered or it's like any person who is, for example, not capable of receiving the package. So I can quickly assist them."

Code: ● HMIs - emergency   Weight score: 0
I3 , 213 - 213
Created: 24-07-2024 15:04 by Analyst1, Modified: 02-09-2024 15:55 by Analyst1
Area: 238   1.31%

17.

"For the Must Have, I think the drone needs to have the obvious signs for medical (Note: Obvious signs for medical) because it needs to distinguish this drone (from the rest) to the others and also it needs to have shining lights for the emergency (Note: Shining light for the emergency) just like, for example, the ambulance, the police cars. Because it's for the emergency, it needs to make the others be aware of what this drone is doing, i.e., is for the emergency activities. So, people will also understand this; if it's going to land and provide this stuff, the other people can take care of or they can move away or they can just understand what the drone is doing and they can provide more support."

Code: ● HMIs - emergency   Weight score: 0
I2 , 62 - 62
Created: 29-07-2024 14:48 by Analyst2, Modified: 02-09-2024 16:26 by Analyst1
Area: 706   2.99%

18.

"the emergency signal (Note: emergency signal (alert to phone) could be sent to everyone's phones."

Code: ● HMIs - emergency   Weight score: 0
I2 , 172 - 172
Created: 24-07-2024 14:39 by Analyst1, Modified: 02-09-2024 16:26 by Analyst1
Area: 97   0.41%

19.

"it's ordered, it needs to pass through and send some alert and also it emits some lights or audios (Note: audio output/light (make people aware this drone)) that remind people that it is like emergency drone."

Code: ● HMIs - emergency   Weight score: 0
I2 , 172 - 172
Created: 24-07-2024 14:39 by Analyst1, Modified: 02-09-2024 16:26 by Analyst1
Area: 208   0.88%

20.

"If they (drones) do not send some audio, people will ignore it because drone when flies, it might not have too much noise sometime. So people who do not notice this and people may get in their (drone) way."

Code: ● HMIs - emergency   Weight score: 0
I2 , 172 - 172
Created: 24-07-2024 14:40 by Analyst1, Modified: 02-09-2024 16:26 by Analyst1
Area: 205   0.87%

21.

"The drone have some voice output and say, oh, we have like the emergency way and just tell that to the surrounding people."

Code: ● HMIs - emergency   Weight score: 0
I2 , 176 - 176
Created: 24-07-2024 14:41 by Analyst1, Modified: 02-09-2024 16:26 by Analyst1
Area: 122   0.52%

22.

"The lights are also on the drone?

Participant 46:44
Also on the drone because people can easily notice these and easily understand what it is says."

Code: ● HMIs - emergency Weight score: 0
I2 , 182 - 184
Created: 24-07-2024 14:41 by Analyst1, Modified: 02-09-2024 16:26 by Analyst1
Area: 149 0.63%

23.

"For the emergency signal, sometimes we could send like some alert to the surrounding people, to their phones. Because, we also receive some alert from the government that oh, there is like some kind of earthquake or some kind of fire and I need to prepare so I can receive these in my phone."

Code: ● HMIs - emergency Weight score: 0
I2 , 188 - 188
Created: 24-07-2024 14:41 by Analyst1, Modified: 02-09-2024 16:26 by Analyst1
Area: 291 1.23%

24.

"So if you're not there, identification of emergency drones, so people go and help, basically, do you expect that?

Participant 53:59
Yeah.

Analyst1 54:01
And how do you want to know this information?

Participant 54:08
Appearance or through its sound or trough lights. I think something that relates to the drones, the thing itself."

Code: ● HMIs - emergency Weight score: 0
I1 , 180 - 186
Created: 29-07-2024 15:39 by Analyst2, Modified: 02-09-2024 16:29 by Analyst1
Area: 348 1.40%

4.1.3. Appearance - emergency

1.

"I would imagine if the drone would also serve as some kind of a sign, for people to know something's happening, to ask for help."

Code: ● Appearance - emergency Weight score: 0
I12 , 175 - 175
Created: 29-07-2024 12:15 by Analyst1, Modified: 02-09-2024 16:25 by Analyst1
Area: 128 0.60%

2.

"If you are sick, you just want to have something very functional. I'm not happy, I'm sick and I need a medicine. But also could be interesting to have something colorful. But in my case, I would say no, I just need my medicine please."

Code: ● Appearance - emergency Weight score: 0
I11 , 122 - 122
Created: 29-07-2024 11:43 by Analyst1, Modified: 08-08-2024 9:53 by Analyst1
Area: 234 1.17%

3.

"The drone could have a specific light, like the ambulances. The drone could have a specific color showing that it's critical package. So as to call attention, that it's important."

Code: ● Appearance - emergency   Weight score: 0
I11 , 182 - 182
Created: 29-07-2024 11:50 by Analyst1, Modified: 08-08-2024 9:53 by Analyst1
Area: 179   0.89%

4.

"If it is for emergency, it should look similar like for medical emergency (Note: looks similar to ambulances (identify emergency)). It should be similar to ambulance. If they look similar, people will consider them that they (drones) are under the same service for emergency. If there are cars or people that are in their way, people will just automatically avoid it and leave space for the drone to fly."
Code: ● Appearance - emergency   Weight score: 0
I10 , 95 - 95
Created: 26-07-2024 16:47 by Analyst2, Modified: 02-09-2024 16:23 by Analyst1
Area: 404   1.84%

5.

"Appearance should be also brilliant, similar to ambulances (Note: bright colors- easy to identify its location, similar to amublances). Brilliant, I mean, it is easy to identify its location (in the sky) with bright colors."
Code: ● Appearance - emergency   Weight score: 0
I10 , 152 - 152
Created: 26-07-2024 15:36 by Analyst1, Modified: 02-09-2024 16:23 by Analyst1
Area: 223   1.01%

6.

"I know that it is for emergency because of its colors and then I want to know for who it is going for, so I can give space for it or help it to get to the person."
Code: ● Appearance - emergency   Weight score: 0
I10 , 154 - 154
Created: 26-07-2024 15:37 by Analyst1, Modified: 02-09-2024 16:23 by Analyst1
Area: 162   0.74%

7.

"Participant 29:16
For the medical delivery, I'm telling again, maybe from far away you cannot really distinguish. You could have the typical kind of like ambulances or the color of the ambulance of the country or the cross. I don't know if the medical things can come to your balcony or to your window, I don't know how if you can even see the floors or anything. We should have (the drone) more distinguishable, so people around also know that this is for a medical reason and it's not like a bag of chips or my Amazon (package)."
Code: ● Appearance - emergency   Weight score: 0
I8 , 131 - 132
Created: 29-07-2024 10:02 by Analyst2, Modified: 02-09-2024 16:30 by Analyst1
Area: 531   1.96%

8.

"We should have (the drone) more distinguishable, so people around also know that this is for a medical reason and it's not like a bag of chips or my Amazon (package)."
Code: ● Appearance - emergency   Weight score: 0
I8 , 132 - 132
Created: 26-07-2024 13:52 by Analyst1, Modified: 02-09-2024 16:30 by Analyst1
Area: 166   0.61%

9.

"The most important thing for a bystander is to have the drone appear in a way to reflect that it delivers medicines (Note: appearance of medical delivery). (It helps me) to not block (the course of the drone) or to know not to engage, or maybe someone wants to steal. Not everyone is thinking of the same. But for me, if it's truly emergency that you have to help by giving way

for an ambulance to pass by, or even if you see it landing, to be more patient or whatever can happen until the person receives it."

Code: ● Appearance - emergency   Weight score: 0
I8 , 166 - 166
Created: 29-07-2024 10:30 by Analyst2, Modified: 02-09-2024 16:30 by Analyst1
Area: 509   1.88%

10.

"Participant 25:58
The white and the red one, like you get on the ambulance (Note: The (+) symbol indicating Pharmacy). It is not a Must Have but the Should Have is like, it's okay, even if the drone doesn't have this white thing and the pharmacy symbol or something. If a drone only is delivering for me, it's fine. If it has, maybe for people, it's easier to recognize in the future when there are many drones. For example, when we are on a highway or something, if there is an ambulance or something going on and if it is with siren, everyone gives the away. For now, anything is fine. If in the case of like traffic increase of the drones, then for the emergency cases, if the drones are like painted in a different manner using white or something, it will be good."

Code: ● Appearance - emergency   Weight score: 0
I7 , 114 - 115
Created: 29-07-2024 10:44 by Analyst2, Modified: 02-09-2024 16:31 by Analyst1
Area: 769   3.00%

11.

"t's okay, even if the drone doesn't have this white thing and the pharmacy symbol or something. If a drone only is delivering for me, it's fine. If it has, maybe for people, it's easier to recognize in the future when there are many drones."

Code: ● Appearance - emergency   Weight score: 0
I7 , 115 - 115
Created: 26-07-2024 12:01 by Analyst1, Modified: 02-09-2024 16:31 by Analyst1
Area: 240   0.94%

12.

"If in the case of like traffic increase of the drones, then for the emergency cases, if the drones are like painted in a different manner using white or something, it will be good."

Code: ● Appearance - emergency   Weight score: 0
I7 , 115 - 115
Created: 26-07-2024 12:02 by Analyst1, Modified: 02-09-2024 16:31 by Analyst1
Area: 180   0.70%

13.

"Analyst1 47:03
What about the appearance of the drone? Is it something similar to what you have seen as a recipient?

Participant 47:09
I absolutely think that is also important now and in future. I don't think we have heavy traffic of drones now. In the future if we have like heavy traffic of drones, if we announce loud and clear and appears in an appealing way, especially this emergency symbol (Note: The (+) symbol indicating Pharmacy), everyone get an insight that there is a certain person (suffering) in the given zone. We can go and look after if we can help him or something."

Code: ● Appearance - emergency   Weight score: 0
I7 , 186 - 189
Created: 29-07-2024 10:53 by Analyst2, Modified: 02-09-2024 16:31 by Analyst1
Area: 600   2.34%

14.

"I suppose what would, help I mentioned of course the signaling visually already on the snacks drone, I think having some information only that it is a medical thing in some way. I think that would already help a lot. Also in signaling to other people as they're going into the bystander element there but also to other people if they know: Oh right, that is a medicine service delivery, versus that also gives a certain sense of urgency (Note: urgency/medical signaling) in how they interact with that drone. If it would accidentally stop at their place."

Code: ● Appearance - emergency   Weight score: 0
I6 , 112 - 112
Created: 29-07-2024 11:25 by Analyst2, Modified: 01-08-2024 13:50 by Analyst1
Area: 554   1.36%

15.

"Yeah, definitely color wise because when you. Oh god, I'm hearing an echo (likely in the online call)
When you imagine helicopters right now. You have, of course your trauma helicopters, which are bright yellow. Everyone knows if they see one of those flying, that is trauma helicopter. It doesn't really matter for our interactions because we're not interacting with those helicopters. But still it's very clear to everyone, that it's a trauma helicopter so that bears a medical situation in some way going on. So with that, simply color is already a huge signaler of what's going on, even if it doesn't necessarily have the same urgency of getting priority. For example, on the roads as ambulances have, then still that element of color gives, I think, enough information to people. So perhaps even with, that thinking about that trauma helicopter a bit further, perhaps I would even prefer if in the second scenario, if it would land in an approximate area in which I am, so it doesn't just show up to me individually, but it lands in the area gives you a pop-up: Hey, I'm close to you, please walk over so that you don't have the personal interactions happening before I can actually get to the drone."

Code: ● Appearance - emergency   Weight score: 0
I6 , 124 - 125
Created: 29-07-2024 11:26 by Analyst2, Modified: 01-08-2024 13:50 by Analyst1
Area: 1205   2.95%

16.

"Yes. There [refers to having written it down]. There's also the ---. And of course, with the signaling of it being an emergency drone (Note: signaling of emergency drone0 does also come a certain: What can you expect from it? So it does mean that culture-wise as well, you need to know, right that's an emergency drone, when one lands, there is someone around looking for medication. Do the same way as right now. If you hear sirens going and you're on the road, you know you make way for them. There's a certain---. It's not necessarily a rule, but it is something you know that you do and there is certain code of conduct in that that does need to happen to actually get into the interactions that I'm describing. Especially with for example, the pop up of: Hey, someone around you is in need of help and signaling of---."

Code: ● Appearance - emergency   Weight score: 0
I6 , 185 - 185
Created: 29-07-2024 11:35 by Analyst2, Modified: 01-08-2024 13:50 by Analyst1
Area: 823   2.02%

17.

"Because speed is of the essence, a clear indicator is also crucial (Note: Clear outside indicator that this drone is 'your' drone). (When) you see a drone come your way, you immediately know like, Oh, this is mine, or this is not mine and you can act quickly."

Code: ● Appearance - emergency   Weight score: 0
I4 , 119 - 119
Created: 25-07-2024 12:47 by Analyst1, Modified: 02-09-2024 15:58 by Analyst1
Area: 259   1.14%

18.

"I think this case is pretty crucial. We're still dealing with emergency and speed. It should clearly indicate towards bystanders that (the drone) is delivering emergency supplies, which

someone is in dire need of (Note: Clearly indicate that its a emergency delivery drone (color of the drone - eg bright yellow, red stripes, maybe a red cross on it)). Bystanders should in some way respect it. It should have a clear indication that is emergency delivery. I think that is probably the most crucial thing. Also, then people won't throw like rocks at it or sticks at it or something worse.

Participant 43:09
Yeah, something that should or could have? I'm thinking maybe some flashing lights (Note: lights to emphasize emergency).

Analyst1 43:22
That again, comes part of indication, right? That it's an emergency,

Participant 43:28
Yeah, I'm thinking if there's a real difference between these two. For example, flashing lights or something would be would be nice. I think that is a clear indication, but it would maybe signal just the extra bit of emergency towards bystanders.

Analyst1 43:47
In the Must Have, how do you think it should clearly indicate? So, in Should Have, it can use like light.

Participant 44:02
Just by the general color or shape of it. Paint that, the reflecting bright yellow, maybe put some red stripes on it.
[Asked participant to add it to the sticky notes]
Maybe red cross on the box beneath it. So it's very clear that it's like serious stuff."

Code: ● Appearance - emergency Weight score: 0
I4 , 171 - 183
Created: 29-07-2024 12:17 by Analyst2, Modified: 02-09-2024 15:58 by Analyst1
Area: 1508 6.67%

19.

"Just by the general color or shape of it. Paint that, the reflecting bright yellow, maybe put some red stripes on it.
[Asked participant to add it to the sticky notes]
Maybe red cross on the box beneath it. So it's very clear that it's like serious stuff."

Code: ● Appearance - emergency Weight score: 0
I4 , 181 - 183
Created: 25-07-2024 13:06 by Analyst1, Modified: 02-09-2024 15:58 by Analyst1
Area: 257 1.14%

20.

"Participant 27:56
No, the alarm has to be the old way (Note: ambulance alarm), not only when it is landing or something. When it is flying and also carrying the alarm, the landing location and signal (Note: Landing location, signal (lights, audio, projection)) just for us to provide help if it's necessary. For example, it decided to land somewhere where the older (recipient) cannot reach, or they're dying, I can help to deliver it to them.

Analyst1 28:35
Okay, so you want to help but how? Should it give you like an alarm you mean?

Participant 28:41
No. For example, this is the person dying and she ordered the medicine but she is not able to receive the package. It landed here but what if this person is not capable to reach, so I can receive the package and give it to her.

Analyst1 29:07
Do you want to know this through an alarm?

Participant 29:09
If it is with alarm, I already know it is emergency. Then I will start to look around who is exactly the person (who) ordered or it's like any person who is, for example, not capable of receiving the package. So I can quickly assist them."

Code: ● Appearance - emergency Weight score: 0
I3 , 204 - 213
Created: 29-07-2024 12:29 by Analyst2, Modified: 02-09-2024 15:55 by Analyst1
Area: 1130 6.20%

21.

"I think the drone needs to have the obvious signs for medical (Note: Obvious signs for medical) because it needs to distinguish this drone (from the rest) to the others"

Code: ● Appearance - emergency Weight score: 0
I2 , 62 - 62
Created: 24-07-2024 14:21 by Analyst1, Modified: 02-09-2024 16:26 by Analyst1
Area: 168 0.71%

22.

"Yeah. So the emergency signal (Note: emergency signal (alert to phone) could be sent to everyone's phones. Well, it's ordered, it needs to pass through and send some alert and also it emits some lights or audios (Note: audio output/light (make people aware this drone)) that remind people that it is like emergency drone."

Code: ● Appearance - emergency Weight score: 0
I2 , 172 - 172
Created: 29-07-2024 14:58 by Analyst2, Modified: 02-09-2024 16:26 by Analyst1
Area: 321 1.36%

23.

"It could have unique shapes or aesthetics in the drone that represent the purpose. We know how the ambulance looks like, we know how the brandweerauto (fire fighter truck) looks like. If we have those kinds of universally accepted or emergency aesthetics of the delivery drones, more into the physical features, then we don't need app on the phone."

Code: ● Appearance - emergency Weight score: 0
I1 , 162 - 162
Created: 24-07-2024 13:26 by Analyst1, Modified: 02-09-2024 16:29 by Analyst1
Area: 348 1.40%

24.

"you're driving on the road, you see ambulance, you need to stop. If you don't, then you will know that you'll crash the ambulance. As long as I know, this is the thing, I can already learn to adjust my action. They're related."

Code: ● Appearance - emergency Weight score: 0
I1 , 170 - 170
Created: 24-07-2024 13:27 by Analyst1, Modified: 02-09-2024 16:29 by Analyst1
Area: 226 0.91%

25.

"I think this will be very similar to ambulance"

Code: ● Appearance - emergency Weight score: 0
I1 , 178 - 178
Created: 24-07-2024 13:29 by Analyst1, Modified: 02-09-2024 16:29 by Analyst1
Area: 46 0.19%

4.2. Human-machine interfaces to communicate drone intentions

1.
"so you already see the green that can we do anything with the light so we don't add on another visual cue onto this already lighting thing because I don't want to make the drone like extremely busy."
Code: ● HMIs Weight score: 0
I5 , 102 - 102
Created: 25-07-2024 16:17 by Analyst1, Modified: 02-09-2024 16:02 by Analyst1
Area: 198 0.48%

2.
"I feel like it's already busy enough. Like their lights, there are sounds like."
Code: ● HMIs Weight score: 0
I5 , 112 - 112
Created: 25-07-2024 16:19 by Analyst1, Modified: 02-09-2024 16:02 by Analyst1
Area: 79 0.19%

3.
"Considering disability people. I would rather have all of them (interfaces). Some people are deaf, some people are blind, I think it's more friendly for them."
Code: ● HMIs Weight score: 0
I3 , 155 - 155
Created: 24-07-2024 15:01 by Analyst1, Modified: 02-09-2024 15:55 by Analyst1
Area: 158 0.87%

4.
"Analyst1 25:30
Okay. I think you also answered the next question like, how do you expect to receive this information and why? You just explained the signals. So I'm now curious about, what about the appearance and interfaces for the drone? How do you want to see the drone to be like, is there any preference there?

Participant 25:54
No, I don't mind about how it looks like, the interface. I mean, as long as, it has some combination of signal. It's fine."
Code: ● HMIs Weight score: 0
I3 , 178 - 181
Created: 29-07-2024 12:26 by Analyst2, Modified: 02-09-2024 15:55 by Analyst1
Area: 471 2.58%

5.
"The good thing is if you have like (information) on both app and drones, you will have redundancy for every interaction that you could think of."
Code: ● HMIs Weight score: 0
I1 , 90 - 90
Created: 24-07-2024 13:05 by Analyst1, Modified: 02-09-2024 16:29 by Analyst1
Area: 144 0.58%

4.2.1. Phone

1.
"I think it could be in the app."
Code: ● Phone Weight score: 0
I12 , 57 - 57
Created: 26-07-2024 12:46 by Analyst2, Modified: 02-09-2024 16:25 by Analyst1
Area: 31 0.15%

2.

"Some of them. Everything (instruction) could be integrated into app, like you're showing all the process that could be very easy and straightforward because it's in my phone. But things like, it (the drone) located me (Note: the drone located me), the notification should be on the phone. I'm not paying attention and I don't see it. It (notification) could be on the phone and when the drone located me it can be on the phone"

Code: ● Phone Weight score: 0
I12 , 61 - 61
Created: 26-07-2024 12:47 by Analyst2, Modified: 02-09-2024 16:25 by Analyst1
Area: 426 2.00%

3.

"But you need to be really careful and I wouldn't go for vocal like somebody saying like, Hey, your delivery is here. I'm not really sure about that. I think those detailed information can be shown on the service app."

Code: ● Phone Weight score: 0
I12 , 69 - 69
Created: 26-07-2024 12:52 by Analyst2, Modified: 02-09-2024 16:25 by Analyst1
Area: 216 1.01%

4.

"I think instruction can be definitely on the app. You can give really nice detailed instructions."

Code: ● Phone Weight score: 0
I12 , 73 - 73
Created: 29-07-2024 12:04 by Analyst1, Modified: 02-09-2024 16:25 by Analyst1
Area: 97 0.46%

5.

"Maybe could be in the app or something. I am relating a lot with Uber, but maybe something like that."

Code: ● Phone Weight score: 0
I11 , 38 - 38
Created: 26-07-2024 13:48 by Analyst2, Modified: 08-08-2024 9:53 by Analyst1
Area: 101 0.50%

6.

"You usually need to say the last four numbers of your cell phone to the delivery guy to receive your package. It's just to make sure that you are the right person receiving your package. You just click on the drone or in your cell phone to open the key and then you can have your package."

Code: ● Phone Weight score: 0
I11 , 42 - 42
Created: 29-07-2024 11:32 by Analyst1, Modified: 08-08-2024 9:53 by Analyst1
Area: 288 1.44%

7.

"I think the recipient can have an app, in the cell phone."

Code: ● Phone Weight score: 0
I11 , 70 - 70
Created: 29-07-2024 11:37 by Analyst1, Modified: 08-08-2024 9:53 by Analyst1
Area: 57 0.28%

8.

"Maybe I can approach the drone to give me access to the thing (to deliver the package), or the drone can just drop the thing (package) for me. I think the idea of drop things is not good. So I think what could be interesting, is an app on my cell phone (to input code), I think it could be cheaper. You then don't need to have a screen on the drone."

Code: ● Phone Weight score: 0

I11 , 74 - 74
Created: 26-07-2024 16:05 by Analyst2, Modified: 08-08-2024 9:53 by Analyst1
Area: 349 1.74%

9.
"As a recipient, I think only in the app would be enough for me"
Code: ● Phone Weight score: 0
I11 , 78 - 78
Created: 26-07-2024 16:06 by Analyst2, Modified: 08-08-2024 9:53 by Analyst1
Area: 62 0.31%

10.
"By default, you create an app, it's something functional. Everyone knows how to deal with an app. I don't know exactly what is your target group, young people or seniors. If it's seniors, it could be different but I think an app is very usual and cheap."
Code: ● Phone Weight score: 0
I11 , 110 - 110
Created: 29-07-2024 11:42 by Analyst1, Modified: 08-08-2024 9:53 by Analyst1
Area: 253 1.26%

11.
"Only the song could be interesting. I was thinking maybe I receive a notification on my cellphone that it's a drone and it's around. What I think is too annoying is why should I know about the drone when I'm ordering nothing. It's more related to not be afraid that the drone is around me with this song. The song is just to know that it's there and someone is receiving the delivery. As a bystander, I don't want to have any information about the drone or interfaces."
Code: ● Phone Weight score: 0
I11 , 158 - 158
Created: 26-07-2024 16:16 by Analyst2, Modified: 08-08-2024 9:53 by Analyst1
Area: 468 2.33%

12.
"The apps will give you a notification, tell you your drone is approaching and it wants to identify with you. Either you have like a button on your app that you can confirm, or you have to check if it is the right drone (for the recipient)."
Code: ● Phone Weight score: 0
I10 , 35 - 35
Created: 26-07-2024 15:10 by Analyst1, Modified: 02-09-2024 16:23 by Analyst1
Area: 239 1.09%

13.
"The app will tell you that the drone is like number 27. There is a number on the drones body, also showing number 27, for you to identify, and then see if it is the right one."
Code: ● Phone Weight score: 0
I10 , 35 - 35
Created: 26-07-2024 15:11 by Analyst1, Modified: 02-09-2024 16:23 by Analyst1
Area: 175 0.80%

14.
"I will say either from app. App is a more definite way because you have to click something or slide something to give instruction to the drone."
Code: ● Phone Weight score: 0
I10 , 51 - 51
Created: 26-07-2024 16:41 by Analyst2, Modified: 02-09-2024 16:23 by Analyst1
Area: 143 0.65%

15.
"As a bystander you wont have app to know about the drones."
Code: ● Phone Weight score: 0

I10 , 111 - 111
Created: 26-07-2024 15:29 by Analyst1, Modified: 02-09-2024 16:23 by Analyst1
Area: 58   0.26%

16.
"I think a visual will be nicer on the screen of my phone, I guess. I'm just drawing parallels between an Uber Eats delivery and drone delivery. That's how I'm thinking. For now, in my head, it's like an UberEATS app that I've ordered this delivery on. I don't know how, but the drone is coming and then it is like the visual you have seen before of something (Note: before arrival on phone; visual of scenario drone and me). It's like a line drawing of a person (stick animation) and then expecting where this delivery is and person like holding the handout and saying, Okay, this is what's expected of you or like, this is what the drone will do. Like leave it five meters from your location, like a pin drop, a visual of that."
Code: ● Phone   Weight score: 0
I9 , 49 - 49
Created: 29-07-2024 8:45 by Analyst2, Modified: 08-08-2024 9:17 by Analyst1
Area: 728   2.77%

17.
"It's like a line drawing of a person (stick animation) and then expecting where this delivery is and person like holding the handout and saying, Okay, this is what's expected of you or like, this is what the drone will do. Like leave it five meters from your location, like a pin drop, a visual of that."
Code: ● Phone   Weight score: 0
I9 , 49 - 49
Created: 26-07-2024 14:28 by Analyst1, Modified: 08-08-2024 9:17 by Analyst1
Area: 303   1.15%

18.
"Analyst1 10:05
Also update on delivery time, where do you want to receive this information?

Participant 10:10
On the same platform (app on phone). I'm assuming now that it is the phone, but it should be one platform. You've ordered the drone delivery on that using some platform, so it should be on the same platform. So that's the Must Have and Should Have."
Code: ● Phone   Weight score: 0
I9 , 50 - 53
Created: 29-07-2024 8:45 by Analyst2, Modified: 08-08-2024 9:17 by Analyst1
Area: 373   1.42%

19.
"With the app or something, I already have an estimate of the delivery status."
Code: ● Phone   Weight score: 0
I9 , 85 - 85
Created: 26-07-2024 14:35 by Analyst1, Modified: 08-08-2024 9:17 by Analyst1
Area: 77   0.29%

20.
"Analyst1 03:43
Yeah. It can include application notifications. And also beyond that, like something you want to see on the drone or from the drone. [Miro board instructions] I will request you to explain me why you think this information is needed and why is it that important?

Participant 04:10
I would like to have some notification that the drone is arriving (Note: Notification that the drone is arriving). I need to know that that's my delivery, that it's coming, in real time to see what is happening. So I think, for that, it's a must have."

Code: ● Phone   Weight score: 0
I8 , 23 - 26
Created: 29-07-2024 9:43 by Analyst2, Modified: 02-09-2024 16:30 by Analyst1
Area: 562   2.08%

21.

"The first thing that I would like to know is for safety that you know: if you have to wait for the drone to land, maybe it's not good to approach a lot, you do not want to have an injury, maybe someone else is around and I don't know what side or anything. I do not know how automating the landing (procedure) can be or how clever the drone can be and be little bit cautious about the surroundings.

Analyst1 09:19
Okay, and how do you want to receive this information? Through what form of communication?

Participant 09:27
By an app or like on my phone because everywhere I go, I have my phone. I think it's the easiest way to receive some kind of additional information."

Code: ● Phone   Weight score: 0
I8 , 42 - 46
Created: 29-07-2024 9:45 by Analyst2, Modified: 02-09-2024 16:30 by Analyst1
Area: 687   2.54%

22.

"Don't know if it's gonna be on app or if it's going to be on the website. To order somewhere out and to order something with the drone, you should have access to the internet or to an application. I think they're gonna be ways before you start using, to see for what you're gonna use."

Code: ● Phone   Weight score: 0
I8 , 74 - 74
Created: 29-07-2024 9:48 by Analyst2, Modified: 02-09-2024 16:30 by Analyst1
Area: 284   1.05%

23.

"Participant 15:25
Yeah. Not only for mine (to be idenified from the rest of drone traffic) but also to know if there are so many around. I think that can help with the real time location. If there are in a park, three more people waiting and then you see or you hear from far away, to expect which one (is yours and which are not), like, Okay, three more drones are gonna pass through me, and to be prepared a bit.

Analyst1 15:52
How do you want to know this information? How do you want to know which is your drone if there are multiple of them?

Participant 16:06
If it's from far away, even if it's like a different color, we cannot really distinguish. I don't know how big the drones are gonna be or how close to me they can approach. I think (I would want to know the information) through an app."

Code: ● Phone   Weight score: 0
I8 , 81 - 86
Created: 29-07-2024 9:51 by Analyst2, Modified: 02-09-2024 16:30 by Analyst1
Area: 817   3.02%

24.

"Maybe people can have an application to see (drones), (like) how you can see the traffic with airplanes, to know if you go somewhere. Maybe you can have other issues like privacy and

private drones, among others. As a bystander, I would like to know, if I go somewhere and see that there are many deliveries about to happen where I am and I don't want to have the sound, I would like to go somewhere else."

Code: ● Phone   Weight score: 0
I8 , 146 - 146
Created: 13-08-2024 16:46 by Analyst1, Modified: 02-09-2024 16:30 by Analyst1
Area: 405   1.50%

25.

"maybe kind of an application within the mobile. Nowadays, everyone knows even our Zomato (Indian Food Delivery App) everything will be using maps and you see its gonna deliver at a certain time. The delivery person (regarding delivery method) is the same, like you get a notification when it's nearby or something."

Code: ● Phone   Weight score: 0
I7 , 39 - 39
Created: 26-07-2024 11:51 by Analyst1, Modified: 02-09-2024 16:31 by Analyst1
Area: 314   1.23%

26.

"for example I ordered on the website having a little tracker on that website would be great. And then especially when that's combined with just an e-mail confirmation of my order, that would be enough information for me because I don't want to get all---. Let's put it like this. I don't want pop up notifications every time the drone moves a minute closer to me. Let's stay away from that. But it's nice to have a little place to do check in. For example, the time estimation or GPS (Note: time estimation), whichever one of the two would be more accurate then that case would fit better, but having some form of tracking that I can just refresh the web page and get an update in there and also feel like it is actually giving me an update, that would be nice.
00:13:49 Analyst1:
And where do you want to access this website? Like on what kind of device?
00:13:56 Participant:
Logically speaking, I would just say my phone because I don't expect to have another device on me when I'm in the park, hanging out with friends looking for a snack."

Code: ● Phone   Weight score: 0
I6 , 66 - 70
Created: 29-07-2024 11:11 by Analyst2, Modified: 02-08-2024 13:51 by Analyst1
Area: 1055   2.59%

27.

"00:14:06 Analyst1:
Yeah. And what about checking in that I'm the recipient? How do you want to know, whether the drone just checked you if you are the recipient?
00:14:21 Participant:
Yeah, I'm not too sure about that. Because that's also something that happens in quite a few different ways. Like sometimes you use an ID card, sometimes they make you do a signature. It's a bit tricky because it's the balance of you want to know that you're the right recipientbut, I don't want to have to go through (the signature processes), so maybe literally just on the website where you ordered it, you get a QR code that appears once your drone is actually where you are. So you stay within the system that you already have like that. That is one thing. It would be nice if you're limiting the amount of systems that are actually involved in it, and it's not happening. Spread out over too many different types of platforms and too many different types of devices, and I already need that singular device to make the order. I would then also prefer using that same device to confirm that this is indeed the person that ordered it."

Code: ● Phone   Weight score: 0
I6 , 71 - 74
Created: 29-07-2024 11:12 by Analyst2, Modified: 01-08-2024 13:50 by Analyst1
Area: 1134   2.78%

28.
"00:15:32 Analyst1:
Yeah. And the last one data collection of who, what, where, like, how do you want to know how much of your data is being collected or how? Yeah, things like that.
00:15:47 Participant:
Yeah, well, it would be great if I could know there was as little as possible being saved because especially when I'm thinking that I could have a QR code to throw a scan before it's delivered, that would mean that I don't need personal data to be saved in any way, shape or form, so that is something I would also want to read back in the terms and conditions of this website when I make an order that does data that's not being collected. So that's purely the GPS location of my phone in that moment that is being used to actually get this drone to the right location and at the moment that QR code is scanned and my package is delivered, that's the end of my active engagement with that system like I might get a review e-mail at one point, but other than that there shouldn't be any active communication or collaboration in there anymore."

Code: ● Phone   Weight score: 0
I6 , 75 - 78
Created: 29-07-2024 11:12 by Analyst2, Modified: 01-08-2024 13:50 by Analyst1
Area: 1058   2.59%

29.
"Yeah. So for now, I'm just still sticking with it with the same web interaction, but rather than the system, the interaction ending when I scan my QR code there I have the opportunity to just go through the questions and these questions can also be very confirming like: Hey, you just all this medication. Are you OK? Did you receive the right medication? Are you helped enough with this? Do you need further assistance? You know, those are the kind of questions you can then get in there."

Code: ● Phone   Weight score: 0
I6 , 100 - 100
Created: 29-07-2024 11:17 by Analyst2, Modified: 01-08-2024 13:50 by Analyst1
Area: 489   1.20%

30.
"I'm pretty sure it's the app is going to tell you it's going to be delivered by drone."

Code: ● Phone   Weight score: 0
I5 , 10 - 10
Created: 25-07-2024 15:48 by Analyst1, Modified: 02-09-2024 16:02 by Analyst1
Area: 86   0.21%

31.
"is looking at this piece of land right, you probably will have a live footage on your phone and then you can just maybe drag the footage around and pick a location on that like just fix the spot."

Code: ● Phone   Weight score: 0
I5 , 141 - 141
Created: 25-07-2024 16:25 by Analyst1, Modified: 02-09-2024 16:02 by Analyst1
Area: 195   0.48%

32.
"So it could be maybe your phone. It could be a tag, whatever it is."

Code: ● Phone   Weight score: 0
I5 , 152 - 152
Created: 29-07-2024 11:52 by Analyst2, Modified: 02-09-2024 16:02 by Analyst1
Area: 67   0.16%

33.
"something like an app on your phone and like a location tracker would create this connection of where the drone is or where you are."

Code: ● Phone   Weight score: 0
I4 , 15 - 15
Created: 25-07-2024 11:36 by Analyst1, Modified: 02-09-2024 15:58 by Analyst1
Area: 132   0.58%

34.

"Participant 14:27
I think probably something on the app. I imagine just a map with like a stamp on it. If you can somehow manage it, something with like a picture would be nice. It will be more like a Google Maps kind of way, where you do the street view thing. That will be amazing, but I think that will be a pain to implement. Just having a delivery location, I think that (would be fine).

Analyst1 15:06
Why do you want to know this in an app?

Participant 15:08
I think that's probably the easiest. You should have a map then you exactly know where you should be. So say, it will give you an address, then I would be a bit uncertain whether they will drop it in front of the door, in the back garden, or, maybe the delivery location will say: I will be at the park. Then it's doesn't have enough indication for me."

Code: ● Phone   Weight score: 0
I4 , 36 - 41
Created: 29-07-2024 12:06 by Analyst2, Modified: 02-09-2024 15:58 by Analyst1
Area: 835   3.69%

35.

"Participant 16:00
Yeah, I put some signal that it knows how to find me (Note: Some signal that it knows how to find me - location sharing, face detection). Actually, I think these two (Note1: Some signal that it knows how to find me - location sharing, face detection; Note2: Some confirmation button/process from my side) are a bit connected for me.

Analyst1 16:24
So you meant signaling that it knows how to find me and the confirmation button/process from your side? So you don't want both of them to be connected? But they're placed in must have and should have, right?

Participant 16:40
Yeah. But I think they go like hand in hand together, I would say. If you confirm from your side like: Hey this is me, then that also gives that signal.

Analyst1 16:57
How do you call it in technical terms? Is it like two way authentication?

Participant 17:04
I think so. Yeah, maybe it's authentication two-way. I think would be nice, if I can say like: Hey, I've received it. If that doesn't happen, then at least they know, like, hey it's not finished yet.

Analyst1 17:28
You want to happen this through? Face detection, or through mobile or, some form of other kind of interface?

Participant 17:41
I think the easiest would be the app. The first thing I thought of was like, Uber Eats, what they will do is they will give you a code. If the delivery person delivers the food, they have to ask

you the code. Otherwise, they cannot confirm they gave it. I think that just adds an extra element of safety. Especially as the recipient, it gives you this feeling like oh, they will not fuck me over because I have the code."

Code: ● Phone Weight score: 0
I4 , 44 - 57
Created: 29-07-2024 12:07 by Analyst2, Modified: 02-09-2024 15:58 by Analyst1
Area: 1663 7.35%

36.

"I thought this is probably a nice one to have: live location of the drone during the delivery (Note: Live location of the drone itself during delivery). When there's only one drone nearby, you can probably see it. But let's say there are two drones or three drones in the area, I think it's very nice to see which one is yours. I don't think it's as crucial because as long as you know, where it's gonna deliver at, it will be okay, so I'd say. If you have this location, then it's so easy and you can follow it. The technology should be there to deliver this.

Analyst1 19:19
The live location? How do you want to know this information? What kind of interface?

Participant 19:27
I would say a map again, I think on the phone.

Analyst1 19:32
With delivery location, did you mean it more like, okay, it's going to drop at this certain address or these coordinates?

Participant 19:40
Yeah"

Code: ● Phone Weight score: 0
I4 , 61 - 69
Created: 29-07-2024 12:07 by Analyst2, Modified: 02-09-2024 15:58 by Analyst1
Area: 918 4.06%

37.

"Wait, I think I put it here (Note: Visual indication that the drone is my order - e.g. number or color that matches something in my app) as well. Some other way to do is to, for example, label them in some way, by giving them like a color. Then you know, like, oh, the one with the green lights is mine or maybe it has a normal plate on it or name. That would also be a nice way. Most people have phones nowadays, should probably be viable on a laptop as well. Although I think if you have a laptop, you're probably not ordering drone service.

Participant 20:48
Why?

Participant 20:50
Yeah, maybe there's a few instances where you are. But I think in general, if you are outside with a laptop, you probably have a phone as well. That's actually not that good of argument [participant thinking]. Most food delivery apps, for example, they work both on the laptop and the phone. I think it should work on a laptop as well. Maybe you are in the park, you are working on your laptop, then it should work on there as well."

Code: ● Phone Weight score: 0
I4 , 73 - 77
Created: 29-07-2024 12:08 by Analyst2, Modified: 02-09-2024 15:58 by Analyst1
Area: 1021 4.51%

38.

"I can track on my phone or the device, how far it is and where it flies, the current location of the delivery (Note: the current location track). I would expect more. It's like the delivery guy and I can say where he is now."

Code: ● Phone   Weight score: 0
I3 , 36 - 36
Created: 24-07-2024 14:47 by Analyst1, Modified: 02-09-2024 15:55 by Analyst1
Area: 224   1.23%

39.

"Participant 09:06
On the device.

Analyst1 09:23
What kind of device you mean?

Participant 09:25
Like my phone.

Participant 09:39
I think all of them are same (Puts the comment on ETA note but it applies to all Notes in the matrix).

Analyst1 10:05
Okay. With regards to dropping location, how precisely you want to know this?

Participant 10:19
For example, I can see myself on my phone (location), and I want to know where the drone gonna land: next to me, like on the current map on my device, or give me a spot on the device where I can expect."

Code: ● Phone   Weight score: 0
I3 , 52 - 63
Created: 29-07-2024 12:20 by Analyst2, Modified: 02-09-2024 15:55 by Analyst1
Area: 580   3.18%

40.

"I can see myself on my phone (location), and I want to know where the drone gonna land: next to me, like on the current map on my device, or give me a spot on the device where I can expect."

Code: ● Phone   Weight score: 0
I3 , 63 - 63
Created: 24-07-2024 14:49 by Analyst1, Modified: 02-09-2024 15:55 by Analyst1
Area: 189   1.04%

41.

"Analyst1 11:33
Okay, do you think having some interfaces on the drone? Like visual or audio or something else helps or what if a drone wants to communicate some messages?

Participant 11:48
I would like to receive it on my phone.

Analyst1 11:57
Why?

Participant 11:58
It's easier. It's like a remote control. I can control everything on my phone. It's convenient.

Sufficient. I don't need to switch device to device like I look at my phone and I interact with him. It's a bit not neutral interaction."

Code: ● Phone Weight score: 0
I3 , 68 - 75
Created: 29-07-2024 12:21 by Analyst2, Modified: 02-09-2024 15:55 by Analyst1
Area: 530 2.91%

42.

"When I got my package then I want to say like, oh, you can fly away now and then I can press like, you can fly away and I confirm I got the package and everything set."

Code: ● Phone Weight score: 0
I3 , 79 - 79
Created: 24-07-2024 14:50 by Analyst1, Modified: 02-09-2024 15:55 by Analyst1
Area: 167 0.92%

43.

"Is it something like so you want to know more information, in the sense I can interpret it as, so you want to be more aware of what the drone is doing? Where the package is? How it's being prepared? Is it?

Participant 19:23
Yeah

Analyst1 19:24
What does it make you feel knowing more information?

Participant 19:28
I can have an alternative plan, basically. If it's not gonna arrive or if any errors gonna happen and I can have a second backup plan.

Analyst1 19:41
Yeah, great. Okay, thank you and how do you like to receive this information?

Participant 19:49
Also on personal device."

Code: ● Phone Weight score: 0
I3 , 113 - 123
Created: 29-07-2024 12:23 by Analyst2, Modified: 02-09-2024 15:55 by Analyst1
Area: 619 3.39%

44.

"Well, I think in this way both are fine. I think if it is like an interactive interface then I can see where is the drone? What is drone doing? On my phone."

Code: ● Phone Weight score: 0
I2 , 43 - 43
Created: 07-08-2024 16:30 by Analyst1, Modified: 02-09-2024 16:26 by Analyst1
Area: 156 0.66%

45.

"you can try to control the drones in the phone by pressing left or up (directional control) and to slightly control like in few centimeters. It might be more safe."

Code: ● Phone Weight score: 0
I2 , 47 - 47
Created: 24-07-2024 14:20 by Analyst1, Modified: 02-09-2024 16:26 by Analyst1
Area: 163 0.69%

46.

"Personally I think the audio is good but I think if in the open environments the audio might have a lot of disturbance and can't hear the voice clearly even if we use the Siri outside. So I think in this situation it might be more feasible if you would like to control this with your phone either by using Bluetooth or either like internet control, and the press (of the button) could be precise. One press they can go left to few centimeters and so on. This is more accurate."

Code: ● Phone   Weight score: 0
I2 , 51 - 51
Created: 29-07-2024 14:46 by Analyst2, Modified: 02-09-2024 16:26 by Analyst1
Area: 476   2.02%

47.

"it's also from the drone. It could be more directive because people could not have this attention to read the things or like to try operate in the phones. In that situation, they can direct and maybe they can directly interact with the drones. It saves time and shorten the procedures beforehand."

Code: ● Phone   Weight score: 0
I2 , 76 - 76
Created: 24-07-2024 14:29 by Analyst1, Modified: 02-09-2024 16:26 by Analyst1
Area: 296   1.25%

48.

"Well, nice. All right, cool. I think they are related, where to pick up and how to pick up. I could imagine there could be multiple ways to get this information at least for me. An app on my phone, for example, where to pick up, like how to pick up some kind of like, just typical user interface on the screen."

Code: ● Phone   Weight score: 0
I1 , 50 - 50
Created: 29-07-2024 15:29 by Analyst2, Modified: 02-09-2024 16:29 by Analyst1
Area: 310   1.25%

49.

"I'm thinking how can I order in the first place, but I assume it was from the app"

Code: ● Phone   Weight score: 0
I1 , 62 - 62
Created: 24-07-2024 13:01 by Analyst1, Modified: 02-09-2024 16:29 by Analyst1
Area: 81   0.33%

50.

"The types of drone should be through the photo of the drone. The payment, I think, simply putting the QR code or signal logo. Maybe not at that time, but maybe for the next time I know that I can like pay on demand.

Analyst1 30:32
How do you want to do this, like on the drone, you scan it or in the app?

Participant 30:41
On the drone, directly on the drone. Basically, I think it will be interesting if we can shift most of the interaction to the drone and move far from the app.

Analyst1 31:03
Why do you think it is a good way?

Participant 31:11
The good thing is if you have like (information) on both app and drones, you will have redundancy for every interaction that you could think of. I don't have a strong reason. Just trying to push towards the inclusivity. Like what if there is no app? Just trying to entertain that idea. If we don't have an app at all for this thing to function. That will be, I think, interesting."

Code: ● Phone   Weight score: 0
I1 , 82 - 90
Created: 24-07-2024 13:05 by Analyst1, Modified: 02-09-2024 16:29 by Analyst1
Area: 962   3.87%

4.2.2. Non-semantic audio

1.
"Subtle sound, (however), could work. Not vocal sound, not voice. For the moment they deliver or for delivery finish (notification), it can be easily done, with simple light or sound patterns, with electronic devices."
Code: ● Non-semantic audio   Weight score: 0
I12 , 61 - 61
Created: 29-07-2024 12:02 by Analyst1, Modified: 02-09-2024 16:25 by Analyst1
Area: 216   1.01%

2.
"It is the same with the sound. But I would be a little bit careful with the sound because we're in a park and I don't know how close the drone is to the person. Maybe when it (drone) is far, it can have lights and when it is close by, it can be sound. When it's a little bit closer, it can be more intimate. With the sound, it is the same way like you turn on your laptop, you have the classic sound then you know, oh, the laptop is awake. You can really play with the character of sound as well."
Code: ● Non-semantic audio   Weight score: 0
I12 , 65 - 65
Created: 26-07-2024 12:50 by Analyst2, Modified: 02-09-2024 16:25 by Analyst1
Area: 496   2.33%

3.
"I'm not sure because I imagine, in public space, sound would be too intrusive. But it depends on what kind of sound. If it's just like bling [participant imitated 'bling' sound], that should be fine. But you need to be really careful and I wouldn't go for vocal like somebody saying like, Hey, your delivery is here. I'm not really sure about that. I think those detailed information can be shown on the service app"
Code: ● Non-semantic audio   Weight score: 0
I12 , 69 - 69
Created: 26-07-2024 12:50 by Analyst2, Modified: 02-09-2024 16:25 by Analyst1
Area: 415   1.95%

4.
"There should also be some kind of sound to say, I delivered. The sound should be very minimal."
Code: ● Non-semantic audio   Weight score: 0
I12 , 77 - 77
Created: 26-07-2024 12:53 by Analyst2, Modified: 02-09-2024 16:25 by Analyst1
Area: 94   0.44%

5.
"They just leave with a turning off laptop sound. You would hear it in your peripheral, but you wouldn't pay attention to it."
Code: ● Non-semantic audio   Weight score: 0
I12 , 113 - 113
Created: 29-07-2024 12:09 by Analyst1, Modified: 02-09-2024 16:25 by Analyst1
Area: 124   0.58%

6.
"Sound is very intrusive in public space. I might want to know this, but how about the other people (they don't want to hear)."
Code: ● Non-semantic audio   Weight score: 0

I12 , 149 - 149
Created: 29-07-2024 12:12 by Analyst1, Modified: 02-09-2024 16:25 by Analyst1
Area: 125   0.59%

7.
"nice to know somehow with the light or like a friendly noise that something is coming and delivering."
Code: ● Non-semantic audio   Weight score: 0
I11 , 142 - 142
Created: 29-07-2024 11:45 by Analyst1, Modified: 08-08-2024 9:53 by Analyst1
Area: 101   0.50%

8.
"Yes. It could have a specific song (Note: Nice song of delivering (tell it is deliverig)) like a noise that washing machine does sometimes, when it's finished. I am trying to imagine that I am in the park with my eyes closed just enjoying the weather and the Sun and then when I hear this, I know there is a drone around me. It's okay because it's a nice song and I know that someone is receiving a package but it's oka"
Code: ● Non-semantic audio   Weight score: 0
I11 , 146 - 146
Created: 26-07-2024 16:15 by Analyst2, Modified: 08-08-2024 9:53 by Analyst1
Area: 419   2.09%

9.
"Not specific to what is delivering but just to know that there is a drone delivering something around. It must be a very soft song, otherwise it will be like annoying like, oh my god (irritated expression)."
Code: ● Non-semantic audio   Weight score: 0
I11 , 150 - 150
Created: 29-07-2024 11:46 by Analyst1, Modified: 08-08-2024 9:53 by Analyst1
Area: 206   1.03%

10.
"Only the song could be interesting. I was thinking maybe I receive a notification on my cellphone that it's a drone and it's around. What I think is too annoying is why should I know about the drone when I'm ordering nothing. It's more related to not be afraid that the drone is around me with this song. The song is just to know that it's there and someone is receiving the delivery. As a bystander, I don't want to have any information about the drone or interfaces."
Code: ● Non-semantic audio   Weight score: 0
I11 , 158 - 158
Created: 26-07-2024 16:16 by Analyst2, Modified: 08-08-2024 9:53 by Analyst1
Area: 468   2.33%

11.
"With the song, something with sound. I was thinking maybe have a light blinking but I think its not important. Maybe the noise is enough."
Code: ● Non-semantic audio   Weight score: 0
I11 , 162 - 162
Created: 29-07-2024 11:48 by Analyst1, Modified: 08-08-2024 9:53 by Analyst1
Area: 137   0.68%

12.
"Either just by language, tell everybody: I'm landing, I'm landing, and with some cues, project some light on the ground directly. People know that the drone will landing on these, like, within this area of projection. Or with some noise like beeps."
Code: ● Non-semantic audio   Weight score: 0
I10 , 31 - 31
Created: 26-07-2024 16:36 by Analyst2, Modified: 02-09-2024 16:23 by Analyst1
Area: 248   1.13%

13.
"the drone should also tell people when it will stop again and leave and which is important (Note: when it boost up again and leave (sound and light)). I will say this (information) also with sound and light."

Code: ● Non-semantic audio   Weight score: 0
I10 , 129 - 129
Created: 26-07-2024 15:34 by Analyst1, Modified: 02-09-2024 16:23 by Analyst1
Area: 207   0.94%

14.
"The sound (Note: approaching sound) would be a nice, Could Have. I think it's more valuable for a bystander."

Code: ● Non-semantic audio   Weight score: 0
I9 , 85 - 85
Created: 29-07-2024 8:50 by Analyst2, Modified: 08-08-2024 9:17 by Analyst1
Area: 108   0.41%

15.
"Of course, the sound will be useful. But it will be even more useful for a bystander than me (as a recipient). This is for the first delivery. But eventually if it becomes more mainstream, then you don't really need a visual indicator of status, but you can already make out from the sound. But then it might be a busy space. So it's like all cars on the road honking. So there should be a balance."

Code: ● Non-semantic audio   Weight score: 0
I9 , 85 - 85
Created: 29-07-2024 8:51 by Analyst2, Modified: 08-08-2024 9:17 by Analyst1
Area: 398   1.52%

16.
"I did visualize it that way. But, is it adding value to the interaction? Or is it just perceived as unnecessary? Well, I think it is a Should Have though. Like what I said with the the sound, it might be too much if everyone's making the sound. But even as a bystander, it'll add value because you will know where this drone is going to drop the parcel, or where is it going to descend. In that sense, it should be somewhere between a Should Have and a Must Have. [Brainstorming on Miro]. This (Note: approaching sound) is more of the approaching sound (of the drone when delivering)."

Code: ● Non-semantic audio   Weight score: 0
I9 , 99 - 99
Created: 29-07-2024 8:54 by Analyst2, Modified: 08-08-2024 9:17 by Analyst1
Area: 584   2.22%

17.
"Yeah, I did mention sound earlier (Note: sound (identify drone)). But I do think eventually if it does become mainstream, it will be too much. Maybe at the start, a sound with the visual might work. But I'm not so sure about the sound. It's difficult to imagine what sound will be okay, in this outdoor space."

Code: ● Non-semantic audio   Weight score: 0
I9 , 199 - 199
Created: 29-07-2024 9:09 by Analyst2, Modified: 08-08-2024 9:17 by Analyst1
Area: 309   1.18%

18.
"Analyst1 09:39
Okay. Just that you know, the phone application is one form and there are other forms as well. There could be interfaces on the drone, like lights or audio signals from the drone. Or you can also think of the way the drone flies or the way the drone appears, as in the colors, the stickers or the shape of the drone. I'm just saying this information, so you know.

Participant 10:10
If I think as a first-time user, I will have no idea what every sound or every color is gonna mean. (If) I see something red, maybe it's something to not approach. If you put under consideration that (some) people are color blind, or especially in the Netherlands, where people from different countries have different views about how to approach or what not to do. If there are a lot of cues on a drone, maybe we'll want to have some general instructions before, to know a little bit how the service is working. Some kind of education before you interact, you can be completely comfortable using it."

Code: ● Non-semantic audio Weight score: 0
I8 , 47 - 50
Created: 29-07-2024 9:46 by Analyst2, Modified: 02-09-2024 16:30 by Analyst1
Area: 1011 3.73%

19.

"For sure, I would like also on the drone to have some kind of sound or maybe some more intense light or more rapid blinking to know they're gonna land or to drop the packet. If I think of a bad scenario, if it's really bright sun and in a park, I don't know how easy is it to see on the drone what is happening. If you don't have sunglasses, so I don't know how easy it is to see from the drone. For sure, some kind of sound or more intense light can be helpful. I'm gonna start to trust that (drones communicating with interfaces) after I'm going to start having a bit of interaction with these kinds of deliveries."

Code: ● Non-semantic audio Weight score: 0
I8 , 94 - 94
Created: 29-07-2024 9:52 by Analyst2, Modified: 02-09-2024 16:30 by Analyst1
Area: 616 2.27%

20.

"It could always be on the app. But if it's like completely emergency, having sound also for people to notify that something is happening. But I don't know about the privacy problem. For example, someone does not want (others) to know that they need medicines. Maybe you can select if you're in a good situation. So that is something of additional feature, (like) you know how much you are in need or the privacy."

Code: ● Non-semantic audio Weight score: 0
I8 , 116 - 116
Created: 29-07-2024 9:59 by Analyst2, Modified: 02-09-2024 16:30 by Analyst1
Area: 412 1.52%

21.

"Yeah, I think I cannot really think of something more. For the privacy (Note: privacy), if I don't want to order anything with a drone and you know just have a drone next to me or flying on my head, I would expect to have some kind of privacy and have my data to get deleted and not have some kind of archive with my face or with my location as a bystander. It is a Should Have. I am not completely sure about the indication for landing (Note: indication of landing), whether it is Should Have or Must Have. For example, in the park, if the drones are landing in a specific spot, I think it's gonna be good to get notified that this is a landing spot so you know as a bystander that, okay, if I go there, there are gonna be drones around. If the drones don't have a specific spot and can be everywhere, then maybe that could be only with more sound, that I'm (drone is) arriving. Not also something (audio signal) really annoying, because already it's annoying the sound (of rotors). (Note: drone air traffic) Maybe people can have an application to see (drones), (like) how you can see the traffic with airplanes, to know if you go somewhere. Maybe you can have other issues like privacy and private drones, among others. As a bystander, I would like to know, if I go somewhere and see that there are many deliveries about to happen where I am and I don't want to have the sound, I would like to go somewhere else."

Code: ● Non-semantic audio Weight score: 0
I8 , 146 - 146
Created: 29-07-2024 10:28 by Analyst2, Modified: 02-09-2024 16:30 by Analyst1
Area: 1415 5.22%

22.
"when I'm the recipient, I don't want this attention grabbing or like center of attention. So, when these are going to deliver because some of the drones, when you watch it, makes quite a noise. So if it is like too noisy, and everyone knows that this drone is delivering, and this drone is moving around this part of the park. In a way I want silencer, silence."

Code: ● Non-semantic audio Weight score: 0
I7 , 51 - 51
Created: 26-07-2024 11:52 by Analyst1, Modified: 02-09-2024 16:31 by Analyst1
Area: 361 1.41%

23.
"Oh, right. Yeah, that's important. Well, I guess it's just not. This is not just for recipient. This is for anyone that is---.
I don't know how much meter should be like 10 meter radius, 5m radius. There should be some sort of a measurement for that, but then pretty much within that range I feel like people all around it related or not related should know the presence of it and maybe the purpose too. But I don't want it to be noise. It's just that you need to---. I hope there is a more ambient way for stating the purpose.
00:15:39 Analyst1:
So first is the presence, right? And how do you want the drone to inform its presence? Is it through some interfaces or the way it appears or? Yeah, like something on your mobile or on road?
00:15:59 Participant:
I think the drone should announce it not just for ---, because this is not just for the recipient. So I'm assuming that it should announce its announce itself to the public space and the---.
Like for this one, I think it could be a bit more intrusive. So I'm just going to say beeping with a low frequency."

Code: ● Non-semantic audio Weight score: 0
I5 , 71 - 78
Created: 29-07-2024 11:45 by Analyst2, Modified: 02-09-2024 16:02 by Analyst1
Area: 1079 2.64%

24.
"I'm imagining just beep beep beep"

Code: ● Non-semantic audio Weight score: 0
I5 , 81 - 81
Created: 25-07-2024 16:12 by Analyst1, Modified: 02-09-2024 16:02 by Analyst1
Area: 33 0.08%

25.
"Ambient is when things are not loud. That is not grabbing your attention that it's kind of. It's a piece of information but it's in the background."

Code: ● Non-semantic audio Weight score: 0
I5 , 94 - 94
Created: 25-07-2024 16:16 by Analyst1, Modified: 02-09-2024 16:02 by Analyst1
Area: 147 0.36%

26.
"Yeah, it's kind of the same (Note: presence - beeping with a low frequency, Note: purpose - Less intrusive, visual, and ambient).
00:41:52 Analyst1:
So you mentioned, yeah, just for audio purpose I'm mentioning. So you mentioned presence and purpose which are similar to what a recipient wants to know, but also for bystander so that they are informed about the intentions.
00:42:07 Participant:
Yeah. Exactly, so that they are informed about the intentions"

Code: ● Non-semantic audio Weight score: 0
I5 , 206 - 210

Created: 29-07-2024 11:59 by Analyst2, Modified: 02-09-2024 16:02 by Analyst1
Area: 469   1.15%

27.

"Exactly the same with bystanders for snacks (Note: presence - beeping with a low frequency, Note: purpose - Less intrusive, visual, and ambient, Note: Visible camera).
00:45:53 Analyst1:
So nothing different and why?
00:45:59 Participant:
I'm more just thinking of from a bystander perspective, I feel like I do not need to participate in this in whatever way, so I don't really care. I just need to know what it's here for and that's it. If it's here for, say, to save people, if it's here to deliver a snack, I feel like as a bystander, it doesn't really ,make too much of a difference for me."
Code: ● Non-semantic audio   Weight score: 0
I5 , 239 - 243
Created: 29-07-2024 12:01 by Analyst2, Modified: 02-09-2024 16:02 by Analyst1
Area: 610   1.49%

28.

"it has very loud sounds like a helicopter landing just to deliver a bottle of water."
Code: ● Non-semantic audio   Weight score: 0
I4 , 99 - 99
Created: 25-07-2024 12:28 by Analyst1, Modified: 02-09-2024 15:58 by Analyst1
Area: 84   0.37%

29.

"Participant 23:13
So the landing location, I want to know where it lands (Note: Landing location, signal (lights, audio, projection)). When it starts landing, I want the landing signal.

Analyst1 23:23
What do you mean by landing signal?

Participant 23:25
Like some sound or a motion of landing or by lights. Something like that.

Analyst1 23:41
And which one do you prefer, lights?

Participant 23:44
All are fine. I don't mind. Considering disability people. I would rather have all of them (interfaces). Some people are deaf, some people are blind, I think it's more friendly for them.

Analyst1 24:05
With regards to signal, you mean the moment it's going to land, should it notify you, or where exactly?

Participant 24:11
The location and landing. The location they can do a spot of lights right.

Analyst1 24:19
Like projection you mean?

Participant 24:21
Yeah, like on the ground, like where exactly, spot on the ground, it is gonna land. When it spotted, say for example, give some clear indication for (when) it's going down."
Code: ● Non-semantic audio   Weight score: 0

I3 , 146 - 163
Created: 29-07-2024 12:26 by Analyst2, Modified: 02-09-2024 15:55 by Analyst1
Area: 1094 6.00%

30.

"I'm on the right position or right angle to get the package, it will somehow change the light or sound"

Code: ● Non-semantic audio Weight score: 0
I1 , 50 - 50
Created: 24-07-2024 12:58 by Analyst1, Modified: 02-09-2024 16:29 by Analyst1
Area: 102 0.41%

31.

"So if you're not there, identification of emergency drones, so people go and help, basically, do you expect that?

Participant 53:59
Yeah.

Analyst1 54:01
And how do you want to know this information?

Participant 54:08
Appearance or through its sound or trough lights. I think something that relates to the drones, the thing itself."

Code: ● Non-semantic audio Weight score: 0
I1 , 180 - 186
Created: 29-07-2024 15:40 by Analyst2, Modified: 02-09-2024 16:29 by Analyst1
Area: 348 1.40%

4.2.3. Semantic audio

1.

"you need to be really careful and I wouldn't go for vocal like somebody saying like, Hey, your delivery is here. I'm not really sure about that. I think those detailed information can be shown on the service app."

Code: ● Semantic audio Weight score: 0
I12 , 69 - 69
Created: 29-07-2024 12:04 by Analyst1, Modified: 02-09-2024 16:25 by Analyst1
Area: 212 1.00%

2.

"Either just by language, tell everybody: I'm landing, I'm landing, and with some cues, project some light on the ground directly. People know that the drone will landing on these, like, within this area of projection. Or with some noise like beeps."

Code: ● Semantic audio Weight score: 0
I10 , 31 - 31
Created: 26-07-2024 16:36 by Analyst2, Modified: 02-09-2024 16:23 by Analyst1
Area: 248 1.13%

3.

"Before it is leaving, I want something like a countdown from him, Hey, I'm leaving within five seconds. So get away from me and don't be nearby, things like that. I think more or less these are Must Have because these are quite safety relevant."

Code: ● Semantic audio Weight score: 0
I10 , 39 - 39
Created: 26-07-2024 15:13 by Analyst1, Modified: 02-09-2024 16:23 by Analyst1
Area: 244 1.11%

4.
"As a bystander you wont have app to know about the drones. Either voice, it tells me that the drone is looking for someone or delivering something, or just visual ones, which are like logos on their (drone) body or if there's light indication. Logos on their body in general, I am also comparing to the normal ones, like Thuisbezorgd (an online food delivery service)."

Code: ● Semantic audio   Weight score: 0
I10 , 111 - 111
Created: 26-07-2024 16:51 by Analyst2, Modified: 02-09-2024 16:23 by Analyst1
Area: 368   1.67%

5.
"Also, where it lands which is also same as previous (as in Q2 answer) (Note: where it lands (projection/sound)). It need not be a projection but any indication that the drone will land. [Rethought] Yep, projection could be or it could be sound, just telling people that it is landing and so people won't get underneath."

Code: ● Semantic audio   Weight score: 0
I10 , 119 - 119
Created: 26-07-2024 16:54 by Analyst2, Modified: 02-09-2024 16:23 by Analyst1
Area: 319   1.45%

6.
"I need to give space. If I want to help, probably another voice. If it needs help then I would expect voice of sentences rather than just sounds."

Code: ● Semantic audio   Weight score: 0
I10 , 170 - 170
Created: 26-07-2024 17:00 by Analyst2, Modified: 02-09-2024 16:23 by Analyst1
Area: 145   0.66%

7.
"(The drone) says it's (delivering) happening, in the language that they can understand. If I share the same balcony with someone, I need to know that something is coming towards where I live or anything."

Code: ● Semantic audio   Weight score: 0
I8 , 166 - 166
Created: 29-07-2024 10:31 by Analyst2, Modified: 02-09-2024 16:30 by Analyst1
Area: 203   0.75%

8.
"It gives you an extra happiness in the interaction."

Code: ● Semantic audio   Weight score: 0
I7 , 79 - 79
Created: 26-07-2024 11:55 by Analyst1, Modified: 02-09-2024 16:31 by Analyst1
Area: 51   0.20%

9.
"Analyst1 43:11
Yeah, and you want to know this as an announcement or displays?

Participant 43:43
Display is fine. I think if I'm the bystander and if it is announcing in a way that because if it lost its way, it's gonna announce like three to four times it gets annoyed by the other, not just to me but the rest of the people in the zone. So if it is just a name display, everyone can understand okay, this person or this drone has lost and looking for this person. So it can just check by, instead of getting annoyed. For example, if someone is playing speaker, when you are like working in the library or something, it annoys you."

Code: ● Semantic audio   Weight score: 0
I7 , 176 - 179
Created: 29-07-2024 10:51 by Analyst2, Modified: 02-09-2024 16:31 by Analyst1
Area: 647   2.52%

10.
"the drone just screaming. I'm here to deliver here, you know, that's going to be annoying."
Code: ● Semantic audio   Weight score: 0
I5 , 88 - 88
Created: 25-07-2024 16:14 by Analyst1, Modified: 02-09-2024 16:02 by Analyst1
Area: 90   0.22%

11.
"The others like friendly, light (coloured) looking (Note: Friendly light outlook) or the voice output (Note: Voice output) also tell me like what the drone is doing and what activities he's going to do. For example, drone could have a voice that says like your delivery or your pizza or something else and if it also emits some friendly lights or have good looking then I will be sure like, okay this is a safe drone flying to me. So it is also linked to this part which is Won't Have, like related to each other, so it won't have like a weapon like outlook (Note: Weapon outlook) or there is no info on the body of drone (Note: No info on the body of drone). So, (lets say), there is a drone flying to me and I have no idea what the drone is like and it's all black, I'm like a recipient, I will be frightened with what it is."
Code: ● Semantic audio   Weight score: 0
I2 , 31 - 31
Created: 29-07-2024 14:44 by Analyst2, Modified: 02-09-2024 16:26 by Analyst1
Area: 827   3.51%

12.
"Participant 13:12
Well, I think in this way both are fine. I think if it is like an interactive interface then I can see where is the drone? What is drone doing? On my phone. But in the real scenarios, it might be better if the drone uses the audio because it helps me to be alert and I know there is a drone and I know that drone is going to do what. So they can just simply say by using the audio sounds and it will be very clear (to express intention of drone) in the physical environment."
Code: ● Semantic audio   Weight score: 0
I2 , 42 - 43
Created: 29-07-2024 14:45 by Analyst2, Modified: 02-09-2024 16:26 by Analyst1
Area: 493   2.09%

13.
"Personally I think the audio is good but I think if in the open environments the audio might have a lot of disturbance and can't hear the voice clearly even if we use the Siri outside. So I think in this situation it might be more feasible if you would like to control this with your phone either by using Bluetooth or either like internet control, and the press (of the button) could be precise. One press they can go left to few centimeters and so on. This is more accurate."
Code: ● Semantic audio   Weight score: 0
I2 , 51 - 51
Created: 29-07-2024 14:46 by Analyst2, Modified: 02-09-2024 16:26 by Analyst1
Area: 476   2.02%

14.
"For bystander, the most important thing is that drones could broadcast what it is doing (Note: Broadcast what it is doing). I think if it is in the cities or urban area and it is like daylight so a little bit voice, sounds will not have much influence on the others. So it is okay to broadcast what it is doing with more sound or at least the drone is like five meters and people within five meters can hear it. I think it is the most important thing."
Code: ● Semantic audio   Weight score: 0
I2 , 96 - 96
Created: 29-07-2024 14:53 by Analyst2, Modified: 02-09-2024 16:26 by Analyst1
Area: 451   1.91%

15.

"I think it is audio. Because I am bystander and my phone does not link to this drone. I do not have some service or something. They (drones are) just passing by and audio is the most effective way, I think."

Code: ● Semantic audio   Weight score: 0
I2 , 124 - 124
Created: 24-07-2024 14:35 by Analyst1, Modified: 02-09-2024 16:26 by Analyst1
Area: 206   0.87%

4.2.4. Display

1.

"I wouldn't expect a big screen on it (drone) that would be too much."

Code: ● Display   Weight score: 0
I12 , 157 - 157
Created: 02-08-2024 15:21 by Analyst1, Modified: 02-09-2024 16:25 by Analyst1
Area: 68   0.32%

2.

"Also, the drone could have a screen or something. For instance, if I can type my key to open the package."

Code: ● Display   Weight score: 0
I11 , 70 - 70
Created: 26-07-2024 16:04 by Analyst2, Modified: 08-08-2024 9:53 by Analyst1
Area: 105   0.52%

3.

"Maybe I can approach the drone to give me access to the thing (to deliver the package), or the drone can just drop the thing (package) for me. I think the idea of drop things is not good. So I think what could be interesting, is an app on my cell phone (to input code), I think it could be cheaper. You then don't need to have a screen on the drone."

Code: ● Display   Weight score: 0
I11 , 74 - 74
Created: 26-07-2024 16:05 by Analyst2, Modified: 08-08-2024 9:53 by Analyst1
Area: 349   1.74%

4.

"It could be on the screen, very subtle. Not audio. Just like on the package that we usually receive. It could be on the screen with the name of the person."

Code: ● Display   Weight score: 0
I11 , 198 - 198
Created: 26-07-2024 16:26 by Analyst2, Modified: 08-08-2024 9:53 by Analyst1
Area: 155   0.77%

5.

"It's easy to change on the drone, different recipients, different names, so I think it's okay."

Code: ● Display   Weight score: 0
I11 , 202 - 202
Created: 29-07-2024 11:51 by Analyst1, Modified: 08-08-2024 9:53 by Analyst1
Area: 94   0.47%

6.

"Participant 16:19
Not with any (visual) interfaces. [Rethought] Maybe visually is also good. For example, as you see in a mobile, the (advertisement) scrolling happens. In the same way, maybe if it is delivering, if it has a keynote, it will be nice. It is not a Must Have though. If it is also displayed in like scrolling, but it's nice. But the audio one, I actually prefer that.

Analyst1 16:57
Why audio?

Participant 17:01
It gives you an extra happiness in the interaction."

Code: ● Display Weight score: 0
I7 , 74 - 79
Created: 29-07-2024 10:41 by Analyst2, Modified: 02-09-2024 16:31 by Analyst1
Area: 494 1.93%

7.

"the name is displayed in a way, we can just go around and ask. It would be nice. It can also be different with different case, if in the future there are more drones to be delivered in certain spot and you're gonna know which one is for which one."

Code: ● Display Weight score: 0
I7 , 175 - 175
Created: 26-07-2024 12:36 by Analyst1, Modified: 02-09-2024 16:31 by Analyst1
Area: 247 0.96%

8.

"Analyst1 43:11
Yeah, and you want to know this as an announcement or displays?

Participant 43:43
Display is fine. I think if I'm the bystander and if it is announcing in a way that because if it lost its way, it's gonna announce like three to four times it gets annoyed by the other, not just to me but the rest of the people in the zone. So if it is just a name display, everyone can understand okay, this person or this drone has lost and looking for this person. So it can just check by, instead of getting annoyed. For example, if someone is playing speaker, when you are like working in the library or something, it annoys you."

Code: ● Display Weight score: 0
I7 , 176 - 179
Created: 29-07-2024 10:51 by Analyst2, Modified: 02-09-2024 16:31 by Analyst1
Area: 647 2.52%

9.

"A screen on that drone saying: Hey, I have an emergency delivery for so and so."

Code: ● Display Weight score: 0
I6 , 197 - 197
Created: 29-07-2024 11:36 by Analyst2, Modified: 01-08-2024 13:50 by Analyst1
Area: 79 0.19%

10.

"Maybe for myself aside like: Oh, it's this is just an Uber Delivery drone.

Analyst1 39:23
And how do you want to see this indication?

Participant 39:31
There's multiple ways, either through a display"

Code: ● Display Weight score: 0
I4 , 135 - 139
Created: 25-07-2024 13:03 by Analyst1, Modified: 02-09-2024 15:58 by Analyst1
Area: 215 0.95%

4.2.5. Ground projection

1.

"Either just by language, tell everybody: I'm landing, I'm landing, and with some cues, project some light on the ground directly. People know that the drone will landing on these, like, within this area of projection."

Code: ● Ground projection   Weight score: 0
I10 , 31 - 31
Created: 26-07-2024 16:36 by Analyst2, Modified: 02-09-2024 16:23 by Analyst1
Area: 217   0.99%

2.

"At the point of delivery, it will be nice to have a visual on the ground (Note: Visual projection on ground), indicating that, okay, this is where I'm going to land. A (projected) circle is reducing (in size) when it's coming down."

Code: ● Ground projection   Weight score: 0
I9 , 85 - 85
Created: 29-07-2024 8:50 by Analyst2, Modified: 08-08-2024 9:17 by Analyst1
Area: 231   0.88%

3.

"I had this visual of the drone is flying, like horizontally and vertically. So when it's arrived at a spot, it is going to descend. So that's when I'm visualizing this marking (projection) on the ground, when it's like coming down, so I have an idea of where it is going to land."

Code: ● Ground projection   Weight score: 0
I9 , 89 - 89
Created: 26-07-2024 14:36 by Analyst1, Modified: 08-08-2024 9:17 by Analyst1
Area: 279   1.06%

4.

"it's more of like, going this way (horizontally above a certain height like a straight line) and then when it's almost above the area of delivery, then it starts coming downwards. That would make more (sense). It'll add a lot of practicality for the people around rather than like a projectile (curve) situation. That way (straight-line trajectory), it will give more context, Oh, that's what going to happen. Maybe it's also nice that it stops there for a couple seconds, and then starts descending."

Code: ● Ground projection   Weight score: 0
I9 , 171 - 171
Created: 26-07-2024 14:56 by Analyst1, Modified: 08-08-2024 9:17 by Analyst1
Area: 500   1.90%

5.

"Participant 36:39
If there is a specific place for landing, everyone knows that, okay, this square meter is for landing drones, like to know don't be really close (to the drones). On the other hand, maybe (there) can be direct light beams (projected) down (on the ground) to know that this is the surface area that the drone is needing, but I don't know how strong that light can be in a daylight or whatever."

Code: ● Ground projection   Weight score: 0
I8 , 153 - 154
Created: 29-07-2024 10:29 by Analyst2, Modified: 02-09-2024 16:30 by Analyst1
Area: 410   1.51%

6.

"Analyst1 24:19
Like projection you mean?

Participant 24:21
Yeah, like on the ground, like where exactly, spot on the ground, it is gonna land. When it spotted, say for example, give some clear indication for (when) it's going down."

Code: ● Ground projection   Weight score: 0
I3 , 160 - 163

Created: 24-07-2024 15:01 by Analyst1, Modified: 02-09-2024 15:55 by Analyst1
Area: 246   1.35%

7.
"it could be interesting if we could have some kind of projection from the drones, like, the area that it will land on the park. Projection from the sky, highlighting the area. For example, target color, this is where you will get your package. And when I approach the area, it will change the color the shape, like, Oh, this is right or not."
Code: ● Ground projection   Weight score: 0
I1 , 50 - 50
Created: 29-07-2024 15:30 by Analyst2, Modified: 02-09-2024 16:29 by Analyst1
Area: 341   1.37%

4.2.6. Lights

1.
"when the drone located me it can be on the phone but it can also be on the drone. I would consider more like light because sound would be too weird here in a public space to call your name."
Code: ● Lights   Weight score: 0
I12 , 61 - 61
Created: 26-07-2024 12:48 by Analyst2, Modified: 02-09-2024 16:25 by Analyst1
Area: 189   0.89%

2.
"If it's just LED lights, it's simple and it can be seen from far away. It's not like complicated pattern you need to recognize."
Code: ● Lights   Weight score: 0
I12 , 65 - 65
Created: 29-07-2024 12:03 by Analyst1, Modified: 02-09-2024 16:25 by Analyst1
Area: 127   0.60%

3.
"Maybe when it (drone) is far, it can have lights and when it is close by, it can be sound."
Code: ● Lights   Weight score: 0
I12 , 65 - 65
Created: 26-07-2024 12:50 by Analyst2, Modified: 02-09-2024 16:25 by Analyst1
Area: 90   0.42%

4.
"It could be the tilting angle of the drone but it could be visual, as in light. I wouldn't expect a big screen on it (drone) that would be too much."
Code: ● Lights   Weight score: 0
I12 , 157 - 157
Created: 26-07-2024 13:15 by Analyst2, Modified: 02-09-2024 16:25 by Analyst1
Area: 148   0.70%

5.
"nice to know somehow with the light or like a friendly noise that something is coming and delivering."
Code: ● Lights   Weight score: 0
I11 , 142 - 142
Created: 29-07-2024 11:45 by Analyst1, Modified: 08-08-2024 9:53 by Analyst1
Area: 101   0.50%

6.
"if there's light indication."
Code: ● Lights   Weight score: 0
I10 , 111 - 111

Created: 26-07-2024 15:30 by Analyst1, Modified: 02-09-2024 16:23 by Analyst1
Area: 28   0.13%

7.
"Lights are to indicate that it is working. So I won't like approach it for no reason, like to just have fun, or to just take a picture. I know that a lot of people when they see a new thing they want to take picture and they are unconsciously getting into a dangerous area because they are getting too close. Some lights could be a way to avoid that. I want to know the purpose of why it comes near me."
Code: ● Lights   Weight score: 0
I10 , 115 - 115
Created: 26-07-2024 16:52 by Analyst2, Modified: 02-09-2024 16:23 by Analyst1
Area: 402   1.83%

8.
"the drone should also tell people when it will stop again and leave and which is important (Note: when it boost up again and leave (sound and light)). I will say this (information) also with sound and light."
Code: ● Lights   Weight score: 0
I10 , 129 - 129
Created: 26-07-2024 15:34 by Analyst1, Modified: 02-09-2024 16:23 by Analyst1
Area: 207   0.94%

9.
"Participant 18:02
If I can have visual to see that something is happening. I know that many drones also have red light with blink sometimes, like not to cross or whatever. So, red light can notify for not (only) danger but also to be careful. On the other hand, if it's something that blinks constantly, I cannot know if this is for me, or just because the drone is on the air."
Code: ● Lights   Weight score: 0
I8 , 93 - 94
Created: 29-07-2024 9:52 by Analyst2, Modified: 02-09-2024 16:30 by Analyst1
Area: 378   1.40%

10.
"if it's something that blinks constantly, I cannot know if this is for me, or just because the drone is on the air."
Code: ● Lights   Weight score: 0
I8 , 94 - 94
Created: 26-07-2024 13:43 by Analyst1, Modified: 02-09-2024 16:30 by Analyst1
Area: 115   0.42%

11.
"For sure, I would like also on the drone to have some kind of sound or maybe some more intense light or more rapid blinking to know they're gonna land or to drop the packet. If I think of a bad scenario, if it's really bright sun and in a park, I don't know how easy is it to see on the drone what is happening. If you don't have sunglasses, so I don't know how easy it is to see from the drone. For sure, some kind of sound or more intense light can be helpful. I'm gonna start to trust that (drones communicating with interfaces) after I'm going to start having a bit of interaction with these kinds of deliveries."
Code: ● Lights   Weight score: 0
I8 , 94 - 94
Created: 29-07-2024 9:52 by Analyst2, Modified: 02-09-2024 16:30 by Analyst1
Area: 616   2.27%

12.

"if there is a camera, I think it would be good that letting people know that it's green or red (visual interface on the drone). If it is green, and it's recording, people will know that they are recorded."

Code: ● Lights Weight score: 0
I7 , 147 - 147
Created: 29-07-2024 10:49 by Analyst2, Modified: 02-09-2024 16:31 by Analyst1
Area: 204 0.80%

13.

"So I'm imagining this, it's quite important to have. You're thinking onto the recipient. As a recipient, I would like to know if the drone is here for me.
So, if there's a word for it. I'm just going to describe it in a sentence. Are you here for me? This should be done with. Ambience and with some privacy. Like I don't want my name to be on the drone. It's like you should not say I'm here for [paticipant says their name]. Like do everybody in the park know [name]. Like I don't want it to be like that. But I want to have some sort of a secret code. Like, let me just write it here like a secret code that both of us know. Like maybe it's flashing green and then I know. So then I know it's my drone so I can react against it like I can walk towards it."

Code: ● Lights Weight score: 0
I5 , 89 - 90
Created: 29-07-2024 11:47 by Analyst2, Modified: 02-09-2024 16:02 by Analyst1
Area: 758 1.86%

14.

"it's maybe on your app you will say dot dot dash and then the light will go blink, blink and then. Like, keep on. Keep on for like 2 seconds and then blink. Blink keep on to and that's how you would have like a secret code with the drone and then you know all is mine."

Code: ● Lights Weight score: 0
I5 , 102 - 102
Created: 25-07-2024 16:18 by Analyst1, Modified: 02-09-2024 16:02 by Analyst1
Area: 268 0.66%

15.

"oh, the one with the green lights is mine"

Code: ● Lights Weight score: 0
I4 , 73 - 73
Created: 29-07-2024 12:08 by Analyst2, Modified: 02-09-2024 15:58 by Analyst1
Area: 41 0.18%

16.

"landing signal?

Participant 23:25
Like some sound or a motion of landing or by lights."

Code: ● Lights Weight score: 0
I3 , 149 - 151
Created: 24-07-2024 15:00 by Analyst1, Modified: 02-09-2024 15:55 by Analyst1
Area: 88 0.48%

17.

"if it also emits some friendly lights or have good looking then I will be sure like, okay this is a safe drone flying to me."

Code: ● Lights Weight score: 0
I2 , 31 - 31
Created: 24-07-2024 13:36 by Analyst1, Modified: 02-09-2024 16:26 by Analyst1
Area: 124 0.53%

18.

"Yeah. So the emergency signal (Note: emergency signal (alert to phone) could be sent to everyone's phones. Well, it's ordered, it needs to pass through and send some alert and also it emits some lights or audios (Note: audio output/light (make people aware this drone)) that remind people that it is like emergency drone."

Code: ● Lights   Weight score: 0
I2 , 172 - 172
Created: 29-07-2024 14:58 by Analyst2, Modified: 02-09-2024 16:26 by Analyst1
Area: 321   1.36%

19.

"The lights are also on the drone?

Participant 46:44
Also on the drone because people can easily notice these and easily understand what it is says."

Code: ● Lights   Weight score: 0
I2 , 182 - 184
Created: 24-07-2024 14:41 by Analyst1, Modified: 02-09-2024 16:26 by Analyst1
Area: 149   0.63%

20.

"So if you're not there, identification of emergency drones, so people go and help, basically, do you expect that?

Participant 53:59
Yeah.

Analyst1 54:01
And how do you want to know this information?

Participant 54:08
Appearance or through its sound or trough lights. I think something that relates to the drones, the thing itself."

Code: ● Lights   Weight score: 0
I1 , 180 - 186
Created: 29-07-2024 15:40 by Analyst2, Modified: 02-09-2024 16:29 by Analyst1
Area: 348   1.40%

4.3. Drone appearance

1.

"If the drone is not carrying anything, so I would think it is a personal drone."

Code: ● Appearance   Weight score: 0
I10 , 15 - 15
Created: 26-07-2024 15:06 by Analyst1, Modified: 02-09-2024 15:53 by Analyst1
Area: 79   0.36%

2.

"If the drone carries something then I will assume that it is like service drone, probably they also have the camera, but the purpose of the camera is not for shooting people, but just to guide his (the drone) own way."

Code: ● Appearance   Weight score: 0
I10 , 19 - 19
Created: 26-07-2024 15:07 by Analyst1, Modified: 02-09-2024 15:53 by Analyst1
Area: 217   0.99%

3.

"That (appearance) should really match with what is coming. That makes a difference from the UberEATS delivery because this person is really coming to your doorstep without the vehicle. So a very generic image of the vehicle would work."

Code: ● Appearance   Weight score: 0
I9 , 73 - 73
Created: 26-07-2024 14:32 by Analyst1, Modified: 08-08-2024 9:17 by Analyst1
Area: 235   0.90%

4.
"There's going to be some package attached to the drone. So that's how you will know that it's a drone for delivery. There should be a clear marking of like a delivery drone versus a drone for personal use"

Code: ● Appearance   Weight score: 0
I9 , 179 - 179
Created: 26-07-2024 14:57 by Analyst1, Modified: 08-08-2024 9:17 by Analyst1
Area: 204   0.78%

5.
"a visual that I have in mind is of a drone with a parcel of some kind attached to it."

Code: ● Appearance   Weight score: 0
I9 , 187 - 187
Created: 26-07-2024 14:58 by Analyst1, Modified: 08-08-2024 9:17 by Analyst1
Area: 85   0.32%

6.
"think it's (Note: Friendly outlook) also like designs, and appearance if it is friendly. Outlook is also linked to all these activities like normal (package) delivery or something else, not just trying to shoot some pictures or drop some dirty bomb or something."

Code: ● Appearance   Weight score: 0
I2 , 120 - 120
Created: 24-07-2024 14:35 by Analyst1, Modified: 02-09-2024 16:26 by Analyst1
Area: 262   1.11%

7.
"Appearance, and then you know, how to act and react."

Code: ● Appearance   Weight score: 0
I1 , 170 - 170
Created: 24-07-2024 13:27 by Analyst1, Modified: 02-09-2024 16:29 by Analyst1
Area: 52   0.21%

4.3.1. Colours/stickers

1.
"I think color is very tricky because usually the drones are black, or white. I don't know if it needs to be colorful. Maybe if it's next to leave or something like that could be interesting. Like something fun."

Code: ● Colours/stickers   Weight score: 0
I11 , 86 - 86
Created: 26-07-2024 16:08 by Analyst2, Modified: 08-08-2024 9:53 by Analyst1
Area: 210   1.05%

2.
"In my imagination, (the drone) it is a black one. For delivery, it could be like more brilliant colour like yellow, orange, but you might also mistaking it as like for other service but not for delivery. Yellow and orange looks more like an emergency service. So probably more like blue like low key ones, but not black. Black and green give me an impression that it is a weapon. You see a lot of in TV that the drones that you use as weapon are either black or green. So probably colors other than that. If you use black in general, I will say, it's also fine because every drone is black now."

Code: ● Colours/stickers   Weight score: 0
I10 , 67 - 67
Created: 26-07-2024 16:42 by Analyst2, Modified: 02-09-2024 16:23 by Analyst1
Area: 594   2.70%

3.
"If drone delivery is a normal service in the future. You want it to be identified from far away. You want to know that it is coming for you. It is always better that you are ready for a drone coming to you and you see it coming rather than it just pops out near you, unknowingly. I guess with more distinguished color and little bit bright, it will catch people's attention."
Code: ● Colours/stickers   Weight score: 0
I10 , 71 - 71
Created: 26-07-2024 16:42 by Analyst2, Modified: 02-09-2024 16:23 by Analyst1
Area: 374   1.70%

4.
"just visual ones, which are like logos on their (drone) body or if there's light indication. Logos on their body in general, I am also comparing to the normal ones, like Thuisbezorgd (an online food delivery service). They (delivery bikes) are always orange, so you expect them to deliver something and if you're not ordering, you know that it is for someone else. So you won't approach."
Code: ● Colours/stickers   Weight score: 0
I10 , 111 - 111
Created: 26-07-2024 16:52 by Analyst2, Modified: 02-09-2024 16:23 by Analyst1
Area: 387   1.76%

5.
"It's just like a DHL box or like a pizza carton thing. Something already familiar."
Code: ● Colours/stickers   Weight score: 0
I9 , 191 - 191
Created: 29-07-2024 9:09 by Analyst2, Modified: 08-08-2024 9:17 by Analyst1
Area: 82   0.31%

6.
"If I think as a first-time user, I will have no idea what every sound or every color is gonna mean. (If) I see something red, maybe it's something to not approach. If you put under consideration that (some) people are color blind"
Code: ● Colours/stickers   Weight score: 0
I8 , 50 - 50
Created: 26-07-2024 13:27 by Analyst1, Modified: 02-09-2024 16:30 by Analyst1
Area: 229   0.85%

7.
"If it's from far away, even if it's like a different color, we cannot really distinguish. I don't know how big the drones are gonna be or how close to me they can approach."
Code: ● Colours/stickers   Weight score: 0
I8 , 86 - 86
Created: 29-07-2024 9:51 by Analyst2, Modified: 02-09-2024 16:30 by Analyst1
Area: 172   0.64%

8.
"Analyst1 19:19
What about the appearance like the colors or shape? Is there any certain preference that you have?

Participant 19:32
From far away, I'm not gonna really manage to look what is happening. I mean, if it's about to land in a park, it's gonna be preferable to not be green for example. I think it has maybe to do a bit with geomorphology or where it goes. If I am waiting in snow, to white wouldn't be easy

(to identify). It's a little bit where you're going to use it. For the shape, I don't think it's important for me. If something gets a bit more sphere shape, you can be a bit more safe to not hit. No matter what, you need to have the Helix (quadcopter) to fly around."

Code: ● Colours/stickers   Weight score: 0
I8 , 95 - 98
Created: 29-07-2024 9:54 by Analyst2, Modified: 02-09-2024 16:30 by Analyst1
Area: 700   2.58%

9.

"The moment it (the drone) approaches, maybe they can have some color like, these (delivery) drones of every company can have or like (how) the (vehicle design of) Thuisbezorgd has. When they (drones) are reaching (the park), maybe they can have some colors or some kind of specific logo that I can know (the purpose). For example, like drone Uber. At least you know, that maybe it's not an individual (private drone) taking photos, playing with the people in the park, but someone knows that (drone) comes, lands and leaves."

Code: ● Colours/stickers   Weight score: 0
I8 , 158 - 158
Created: 29-07-2024 10:29 by Analyst2, Modified: 02-09-2024 16:30 by Analyst1
Area: 524   1.93%

10.

"when I order Domino's and I see someone with dominoes packages walking up there's that, there's that certainty in there that I know what to expect."

Code: ● Colours/stickers   Weight score: 0
I6 , 41 - 41
Created: 25-07-2024 16:58 by Analyst1, Modified: 01-08-2024 13:50 by Analyst1
Area: 147   0.36%

11.

"I think appearance is already a large part. There's, of course, most delivery services have a certain color, like when you see pink, when you see orange cycling around that already signals a lot on what's actually happening. So having that level of signaling is already some form that would fit in this Must Have."

Code: ● Colours/stickers   Weight score: 0
I6 , 45 - 45
Created: 29-07-2024 11:09 by Analyst2, Modified: 02-08-2024 11:56 by Analyst1
Area: 313   0.77%

12.

"I think it is a very low threshold thing to actually include, but it does make a huge difference being the recipients."

Code: ● Colours/stickers   Weight score: 0
I6 , 45 - 45
Created: 25-07-2024 16:59 by Analyst1, Modified: 01-08-2024 13:50 by Analyst1
Area: 118   0.29%

13.

"hen you look at delivery bikes and stuff. They kind of blend into the environment and you just notice the color and you already know what is happening."

Code: ● Colours/stickers   Weight score: 0
I6 , 129 - 129
Created: 26-07-2024 11:21 by Analyst1, Modified: 02-08-2024 11:57 by Analyst1
Area: 151   0.37%

14.

"you're going to look for the logo of the app to see: Oh is it for me? Like if you see it is the same logo then you probably think, OK, well."

Code: ● Colours/stickers   Weight score: 0
I5 , 21 - 21

Created: 25-07-2024 16:04 by Analyst1, Modified: 02-09-2024 16:02 by Analyst1
Area: 141   0.35%

15.
"They're going to print it all over the box. It's going to be free commercial."
Code: ● Colours/stickers   Weight score: 0
I5 , 21 - 21
Created: 25-07-2024 16:04 by Analyst1, Modified: 02-09-2024 16:02 by Analyst1
Area: 77   0.19%

16.
"maybe it's on the box (package attached)."
Code: ● Colours/stickers   Weight score: 0
I4 , 139 - 139
Created: 29-07-2024 12:15 by Analyst2, Modified: 02-09-2024 15:58 by Analyst1
Area: 41   0.18%

17.
"ither as a recipient or bystander, knowing the commercial brand, I know this is safe."
Code: ● Colours/stickers   Weight score: 0
I2 , 116 - 116
Created: 24-07-2024 14:34 by Analyst1, Modified: 02-09-2024 16:26 by Analyst1
Area: 85   0.36%

18.
"I think the brand is just like the visual how the drone looks like and like some brand. I think that's enough and you do not need like an audio, phones or something."
Code: ● Colours/stickers   Weight score: 0
I2 , 148 - 148
Created: 29-07-2024 14:56 by Analyst2, Modified: 02-09-2024 16:26 by Analyst1
Area: 165   0.70%

19.
"there are maybe its colors"
Code: ● Colours/stickers   Weight score: 0
I2 , 152 - 152
Created: 24-07-2024 14:38 by Analyst1, Modified: 02-09-2024 16:26 by Analyst1
Area: 26   0.11%

20.
"Ideally, only from the looks of the drones. But initially, we need to have some kind of educational awareness for the public and with the governmental logos."
Code: ● Colours/stickers   Weight score: 0
I1 , 166 - 166
Created: 29-07-2024 15:37 by Analyst2, Modified: 02-09-2024 16:29 by Analyst1
Area: 157   0.63%

4.3.2. Friendly/rounded looks

1.
"If it's delivering food or snacks, I wouldn't expect something that's very mechanical, or masculine. I would expect something more, I wouldn't say futuristic, but more clean, more friendly and more (like it fits into) daily lifestyle."
Code: ● Friendly/rounded looks   Weight score: 0
I12 , 89 - 89
Created: 26-07-2024 12:55 by Analyst2, Modified: 02-09-2024 16:25 by Analyst1
Area: 234   1.10%

2.

"In my opinion, the classical drones we have now are very ugly for me, because it seems like a spider and I don't like spiders. It's like a huge spider for me. But I understand that it is important to have that design. It could be more rounded, for me it's more friendly, without pointed (edges). Your package is closed (protected) and the drone can fly during the rain or something like that. I (should) know that my deliver will be safe."

Code: ● Friendly/rounded looks Weight score: 0
I11 , 82 - 82
Created: 26-07-2024 16:06 by Analyst2, Modified: 08-08-2024 9:53 by Analyst1
Area: 438 2.18%

3.

"could be more rounded, for me it's more friendly, without pointed (edges). Your package is closed (protected) and the drone can fly during the rain or something like that. I (should) know that my deliver will be safe."

Code: ● Friendly/rounded looks Weight score: 0
I11 , 82 - 82
Created: 29-07-2024 11:39 by Analyst1, Modified: 08-08-2024 9:53 by Analyst1
Area: 217 1.08%

4.

"As a bystander, I think, the same design as to communicate that someone will receive something, so very rounded. If possible not to see the helices (rotors) and something more friendly."

Code: ● Friendly/rounded looks Weight score: 0
I11 , 166 - 166
Created: 29-07-2024 11:48 by Analyst1, Modified: 08-08-2024 9:53 by Analyst1
Area: 185 0.92%

5.

"No, when I'm thinking of a delivery drone, a visual that I have in mind is of a drone with a parcel of some kind attached to it."

Code: ● Friendly/rounded looks Weight score: 0
I9 , 187 - 187
Created: 29-07-2024 9:08 by Analyst2, Modified: 08-08-2024 9:17 by Analyst1
Area: 128 0.49%

6.

"For the shape, I don't think it's important for me. If something gets a bit more sphere shape, you can be a bit more safe to not hit. No matter what, you need to have the Helix (quadcopter) to fly around."

Code: ● Friendly/rounded looks Weight score: 0
I8 , 98 - 98
Created: 29-07-2024 9:55 by Analyst2, Modified: 02-09-2024 16:30 by Analyst1
Area: 204 0.75%

7.

"It should be appealing good, not like a kind of animated one, and also some kind of pretty solid body."

Code: ● Friendly/rounded looks Weight score: 0
I7 , 71 - 71
Created: 26-07-2024 11:55 by Analyst1, Modified: 02-09-2024 16:31 by Analyst1
Area: 102 0.40%

8.

"It should be appealing and it makes me happy."

Code: ● Friendly/rounded looks Weight score: 0
I7 , 163 - 163
Created: 26-07-2024 12:34 by Analyst1, Modified: 02-09-2024 16:31 by Analyst1
Area: 45 0.18%

9.

"Not just rustic, it should be like aesthetic. It makes me appealing that yeah, this drone is gonna deliver and it is not like rustic like an experimental piece."

Code: ● Friendly/rounded looks Weight score: 0
I7 , 167 - 167
Created: 26-07-2024 12:34 by Analyst1, Modified: 02-09-2024 16:31 by Analyst1
Area: 160 0.62%

10.

"I want to say sharp looking edges or just anything that makes people alert (Note: Alarming visuals, sharp looknig exterior). I'm going to say alarming visuals. But I will give examples.
00:42:37 Analyst1:
So it's more inclusive, you mean?
00:42:41 Participant:
Yeah. Just I wanted (it) to look safe.
00:42:45 Analyst1:
And how do you define looking safe?
00:42:45 Participant:
Maybe with softer edges and like the turns of the corner is not like a very acute angle instead of ----. and also just not like really polished metal looking reflective, you know, like the kind of thing if it drops on you or cut the *******[said: f*ing] like wound on you, that kind of stuff."

Code: ● Friendly/rounded looks Weight score: 0
I5 , 210 - 218
Created: 29-07-2024 12:00 by Analyst2, Modified: 02-09-2024 16:02 by Analyst1
Area: 694 1.70%

11.

"Just I wanted (it) to look safe."

Code: ● Friendly/rounded looks Weight score: 0
I5 , 214 - 214
Created: 25-07-2024 16:38 by Analyst1, Modified: 02-09-2024 16:02 by Analyst1
Area: 32 0.08%

12.

"Maybe with softer edges and like the turns of the corner is not like a very acute angle instead of ----. and also just not like really polished metal looking reflective, you know, like the kind of thing if it drops on you or cut the *******[said: f*ing] like wound on you"

Code: ● Friendly/rounded looks Weight score: 0
I5 , 218 - 218
Created: 25-07-2024 16:38 by Analyst1, Modified: 02-09-2024 16:02 by Analyst1
Area: 271 0.66%

13.

"Yeah, with this one (Note: Pleasant drone - no loud sounds, smooth operations, maybe some visually pleasing looks, a name for the drone). These are basically like nice extras for me to enhance the experience. Sometimes in restaurants, they will have robots that serve the food. They make it like a cute robot, like a cat robot, or that has some interface showing a cute face or that will talk funny. I think those things are functionally not really needed. But it will be fun. So I think about maybe a nice look for it. Maybe it has some angry bird costume or whatever. I think it will be fun for some people to get the food delivered by an angry bird or something that looks funny or beautiful. The same for the loud sounds. If it has a very loud sound, maybe you would feel a bit constrained. I think it's not that nice. If it's, for example, it has very loud sounds like a helicopter landing just to deliver a bottle of water. If it would, for example, be pretty quiet and it would like be very smooth that just enhances the experience. I don't think it's crucial to get right to make (the interaction) work."

Code: ● Friendly/rounded looks Weight score: 0
I4 , 99 - 99

Created: 29-07-2024 12:11 by Analyst2, Modified: 02-09-2024 15:58 by Analyst1
Area: 1111 4.91%

14.
"If there are cameras on it, maybe it's wise to hide them or close them in some way and they only open when they need to. I feel this is a pretty sensitive point, you should think about getting it right."
Code: ● Friendly/rounded looks Weight score: 0
I4 , 103 - 103
Created: 25-07-2024 12:45 by Analyst1, Modified: 02-09-2024 15:58 by Analyst1
Area: 202 0.89%

15.
"maybe it has some costume, maybe it's on the box (package attached)."
Code: ● Friendly/rounded looks Weight score: 0
I4 , 139 - 139
Created: 25-07-2024 13:02 by Analyst1, Modified: 02-09-2024 15:58 by Analyst1
Area: 68 0.30%

16.
"If they put something over, it could be fun"
Code: ● Friendly/rounded looks Weight score: 0
I3 , 67 - 67
Created: 24-07-2024 14:50 by Analyst1, Modified: 02-09-2024 15:55 by Analyst1
Area: 43 0.24%

17.
"The others like friendly, light (coloured) looking (Note: Friendly light outlook) or the voice output (Note: Voice output) also tell me like what the drone is doing and what activities he's going to do. For example, drone could have a voice that says like your delivery or your pizza or something else and if it also emits some friendly lights or have good looking then I will be sure like, okay this is a safe drone flying to me. So it is also linked to this part which is Won't Have, like related to each other, so it won't have like a weapon like outlook (Note: Weapon outlook) or there is no info on the body of drone (Note: No info on the body of drone). So, (lets say), there is a drone flying to me and I have no idea what the drone is like and it's all black, I'm like a recipient, I will be frightened with what it is."
Code: ● Friendly/rounded looks Weight score: 0
I2 , 31 - 31
Created: 29-07-2024 14:43 by Analyst2, Modified: 02-09-2024 16:26 by Analyst1
Area: 827 3.51%

18.
"If the drone wings are exposed, like the helicopter, it might cut the hands or might cut the head and people may get hurt. If the rotors are well protected by a lot of circles of plastic or some soft metals, like fencing our home, so we are not going to be hurt by the wings. It can be frightening if the drone flies very close to us and gives us delivery. But if he is not, so it might be like to drop something in front of us or they can just drop it from the air and maybe from like 10 centimeters or 20 centimeters from the ground, but it has a distance with people."
Code: ● Friendly/rounded looks Weight score: 0
I2 , 35 - 35
Created: 29-07-2024 14:45 by Analyst2, Modified: 02-09-2024 16:26 by Analyst1
Area: 570 2.42%

19.
"I think it's (Note: Friendly outlook) also like designs, and appearance if it is friendly. Outlook is also linked to all these activities like normal (package) delivery or something else, not just trying to shoot some pictures or drop some dirty bomb or something."
Code: ● Friendly/rounded looks Weight score: 0
I2 , 120 - 120

Created: 29-07-2024 14:55 by Analyst2, Modified: 02-09-2024 16:26 by Analyst1
Area: 264   1.12%

20.

"Analyst1 42:16
Yeah. Okay. And the friendly outlook, is it through its appearance, or are you want to hear that it is friendly?

Participant 42:25
Yeah, I didn't want to need to be sharp. I think that is the kind of way of friendly and there are maybe its colors. And I think this is good to define friendly. I think it's like designers work."

Code: ● Friendly/rounded looks   Weight score: 0
I2 , 149 - 152
Created: 29-07-2024 14:57 by Analyst2, Modified: 02-09-2024 16:26 by Analyst1
Area: 356   1.51%
