# Supplementary material for: Human interactions with delivery drones in public spaces: design recommendations from recipient and bystander perspectives
Source: Front Robot AI. 2025 May 30;12:1580289. doi: 10.3389/frobt.2025.1580289 (PMC12162322; doi:10.3389/frobt.2025.1580289)
Supplement: Supplementary file 1 [file DataSheet1.zip › Methods/Instructions.pdf]

Participant Information

Hi,

Thank you for being a part of our research. This sheet briefs you about the study and the steps you need to take. So, please read it carefully.

About the study

The purpose of this study is to understand user requirements regarding the design of and interaction with delivery drones in public spaces. In this study, you will be personally asked to answer interview questions about delivery drones and later participate in focus groups where you will sketch drones and storyboard interaction spaces.

Duration

This study will take a total of 2.5 hours, divided into two parts: an interview and a focus group. The interview will take about 1 hour, and the focus group will take about 1.5 hours. These sessions will not be held back-to-back but will be scheduled on different days or weeks.

The information below helps you to prepare and understand the context of the study.

Delivery scenario

This study investigates the interaction with drones that deliver a package to a user in a public space such as a park (see Figure 1). In the scenario, the drone delivers daily utilities (e.g. snacks) as requested by you or another person present in the park.

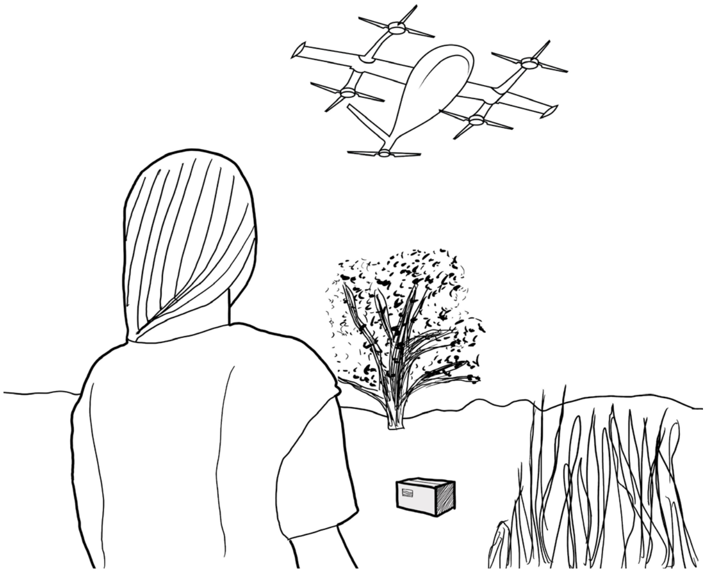

Supplementary Figure 1: An illustration of a user watching a delivery drone arrive in a park.

Human roles

Please imagine yourself in two roles: recipient and bystander. People interact with drones in two main ways: actively as recipients of a package, or passively as bystanders near the drone. While both can be present during delivery, their roles differ based on their awareness and engagement with the drone.

Further descriptions of the human roles are provided below in the section below titled "Story."

Story
After reading the stories below, imagine yourself in the roles of recipient and bystander, separately.

Recipient role

On a quiet afternoon in a public park, you are having a picnic with your friends. Realising you need more snacks, you turn to your mobile and visit the local drone delivery service website. You select the items you need and finalise your order. As you hit "confirm," you imagine a drone flying through the sky, heading to your location in the park.

Bystander role
On a quiet afternoon in a public park, you are having a picnic with your friends. As you relax, you hear a distant hum growing louder. Looking up, you see a sleek drone descending into the park. Although you aren't expecting anything, you notice the presence of the drone in your vicinity.

Interview tasks

Once you read the information above, you are ready to start the interview. Please respond to the interview questions (see below) from the perspectives of both bystander and recipient roles, separately. Use the Miro board (link will be shared;) to categorise information using the MoSCoW prioritisation method[1] for question numbers 2, 4, 6 and 8.

****

Start of interview

****

Interview questions:

Recipient role -

1. Would/wouldn't you imagine feeling uncertainty when a drone approaches you? If so, what factors might make you feel uncertain, and why?
2. What information do you expect to receive based on the recipient role? Why?
3. How do you expect to receive this information and why? (Follow1: What characteristics [(related to the appearance, HMIs and control)] do you expect the delivery drone to possess and why?; Follow2: What if the drone communicates some messages? Interfaces?)
4. Consider a scenario like the delivery of emergency medicines. What information do you expect to receive, and how do you expect to receive it? Why?

Bystander role -

5. Would/wouldn't you imagine feeling uncertainty when a drone approaches in proximity? If so, what factors might make you feel uncertain, and why?
6. What information do you expect to receive based on the bystander role? Why?
7. How do you expect to receive this information and why? (Follow1: What characteristics [(related to the appearance, HMIs and control)] do you expect the delivery drone to possess and why?; Follow2: What if the drone communicates some messages? Interfaces?)
8. Consider a scenario like the delivery of emergency medicines. What information do you expect to receive, and how do you expect to receive it? Why?

General remark

9. Are there any other general remarks or aspects you would like to mention?

****

End of interview

****

[1] Participants categorise the provided information or characteristics using the MoSCoW prioritization method, sorting them into four categories: 1) "Must Have", 2) "Should Have", 3) "Could Have", and 4) "Will not Have" (Clegg & Barker, 1994).

Focus group tasks

Please storyboard an interaction scenario and the drone that delivers a package in the interaction, imagining yourself in the role of the recipient. Please explain your design choices at every stage.

A few moments later….

Please storyboard an interaction scenario and the drone that delivers a package in the interaction, imagining yourself in the role of the bystander. Please explain your design choices at every stage.

A few moments later….

Please watch the videos of existing drone models and reflect on your thoughts and preferences. In addition, you are allowed to adjust your sketches based on the features observed in the existing drone models if found beneficial.

Note: Please follow design thinking principles such as empathise, define, ideate, and be constructive with your arguments while respecting each other's opinions.
