# Supplementary material for: Human interactions with delivery drones in public spaces: design recommendations from recipient and bystander perspectives
Source: Front Robot AI. 2025 May 30;12:1580289. doi: 10.3389/frobt.2025.1580289 (PMC12162322; doi:10.3389/frobt.2025.1580289)
Supplement: Supplementary file 1 [file DataSheet1.zip › Methods/Videos_on_existing_drone_models.pdf]

Videos on existing drone models

Videos showcasing the drone models, which differed in terms of wing type, delivery method and aesthetics, were extracted from YouTube (https://www.youtube.com/). These videos were edited using Adobe Premiere Pro to emphasize drone features and interactions, while removing textual overlays, explanations, and audio. The edited materials were used under fair use provisions, strictly for the purposes of criticism, commentary, and reporting. The video sources are listed below:

1. Amazon Air (Octacopter hybrid with drop):
    a. https://www.youtube.com/watch?v=ahkeyw-FUlQ (News)
    b. https://www.youtube.com/watch?v=WtK1QJyfAnc (CNET)
2. Manna aero (Quadcopter with drop):
   https://www.youtube.com/watch?v=oTJKo15rqtc
3. Wing (Hybrid VTOL with cable):
   https://youtu.be/d51c9CpSNqw?si=phs9xtgbhaBdbrNq
4. Zipline (Hybrid VTOL with cable and mini-droid):
   https://www.youtube.com/watch?v=o7_X9vVQlAg

Inclusion criteria for the videos:

1. Drones have the capability to deliver to the end (public) user in a small interaction space (e.g., backyard, park, public space).
2. The drones do not need a dedicated landing station.
3. The drone models are available from the company pages on the internet or social media.
