# Supplementary material for: Human interactions with delivery drones in public spaces: design recommendations from recipient and bystander perspectives
Source: Front Robot AI. 2025 May 30;12:1580289. doi: 10.3389/frobt.2025.1580289 (PMC12162322; doi:10.3389/frobt.2025.1580289)
Supplement: Supplementary file 1 [file DataSheet1.zip › README.pdf]

Readme for Supplementary Material

Folders:

1. **Methods:**
   - **Instructions.pdf:** Includes instructions, interview questions, and fictional stories to help participants imagine the two roles.
   - **Videos_on_existing_drone_models.pdf:** Contains source video links and criteria for selecting drone models.
2. **Data_&_results:**
   - **Interviews:**
     - **Interview_codes_MaxQDA.pdf:** Contains codes and themes (with participant quotes) from the thematic analysis of interview transcriptions.
     - **All_sticky_notes.xlsx:** Contains all post-it notes collected from the Miro boards during the interviews, sorted by user requirement themes and sub-themes (Tracking information for the recipient, Recognition of the drone and recipient, Landing/take-off intentions of the drone, and Limited user intervention in drone control). The notes are colour-coded using the MoSCoW prioritisation method.
   - **Focus_groups:**
     - Contains PDF files of the storyboards (including interface cards), drone sketches (folder name: Storyboards_sketches), and participant reflections on existing drone models (folder name: Reflections_on_existing_drone_models -> file name: FG_reflection_codes.pdf), all subjected to thematic analysis.
     - **File Organisation of folder Storyboards_sketches:**
       - **PDF Naming:** Each file is named "FG" followed by the group number (e.g., FG1.pdf for Group 1).
       - **PDF Content Order:** Storyboard (recipient role) → Interface cards (recipient role) → Drone sketch (recipient role) → Storyboard (bystander role) → Drone sketch (bystander role).
       - **Note:** No interface cards were used for the bystander role.
     - **Inconsistencies:**
       - Some sketches and storyboards for a given role and group might be inconsistent, with certain design features mentioned in the storyboard but not in the drone sketch (e.g., FG1 included a projection feature in the storyboard but did not sketch it). These inconsistencies were addressed during analysis by the focus group moderators, using transcriptions for reference.
